# Collagen VI is a fibrosis-associated signal disrupting muscle regeneration across distinct human myopathies

**DOI:** 10.1038/s44319-026-00834-0

**Published:** 2026-06-19

**Authors:** Laura Muraine, Mona Bensalah, Stephen Gargan, Paul Dowling, Anne Bigot, Valérie Allamand, Jamila Dhiab, Maria Kondili, Sophie Perié, Jean Lacau St-Guily, Gillian Butler-Browne, Vincent Mouly, Kay Ohlendieck, Capucine Trollet, Elisa Negroni

**Affiliations:** 1 Center for Research in Myology U974, Sorbonne Université, INSERM, Myology Institute, Paris, France; 2 Department of Biology, Maynooth University, National University of Ireland, Maynooth W23F2H6, Co, Kildare, Ireland; 3 Kathleen Lonsdale Institute for Human Health Research, Maynooth University, Maynooth W23F2H6, Co, Kildare, Ireland; 4 Department of Otolaryngology Head and Neck Surgery, Com Maillot-Hartmann Clinic, Neuilly Sur Seine, France; 5 Department of Otolaryngology-Head and Neck surgery, Rothschild Foundation Hospital and Sorbonne University, Paris, France

**Keywords:** Molecular Biology of Disease, Musculoskeletal System, Signal Transduction

## Abstract

Muscle fibrosis is a major driver of progression in diverse myopathies, yet the conserved molecular mediators of this process in humans remain poorly defined. Here, we identify collagen VI as a common regeneration-impairing extracellular matrix (ECM) component across three distinct human myopathies: Duchenne Muscular Dystrophy (DMD), Oculopharyngeal Muscular Dystrophy (OPMD), and Inclusion Body Myositis (IBM). Proteomic profiling of fibrotic biopsies reveals consistent upregulation of collagen VI and laminin γ1, alongside disease-specific alterations. Fibroadipogenic progenitors (FAPs) are the predominant source of these ECM components, including collagen VI and laminin γ1. Functionally, xenotransplantation of patient-derived FAPs into regenerating mouse muscle induces localized collagen deposition, myofiber atrophy, and depletion of Pax7⁺ muscle stem cells. Mechanistic assays demonstrate that FAP-derived collagen VI is sufficient to impair myogenic fusion, while silencing COL6 in patient FAPs restores fusion capacity, directly linking pathological collagen VI deposition to regeneration failure. Our findings uncover collagen VI as a conserved effector of fibrosis and stem cell niche disruption in human myopathies, positioning it as a potential therapeutic target across genetically and clinically distinct muscle diseases.

## Synopsis

Proteomic analysis reveals that collagen VI is a conserved effector of fibrosis and stem cell niche disruption in human myopathies, positioning it as a potential therapeutic target across genetically and clinically distinct muscle diseases.

- Proteomic analysis reveals ECM disease-specific and shared changes in three human myopathies.
- Collagen VI is found upregulated across three distinct human myopathies.
- FAPs are the predominant source of collagen VI within fibrotic muscle.
- Collagen VI impairs myogenic fusion and disrupts stem cell niche during regeneration.

## Abstract (web summary)

Proteomic analysis reveals that collagen VI is a conserved effector of fibrosis and stem cell niche disruption in human myopathies, positioning it as a potential therapeutic target across genetically and clinically distinct muscle diseases.

## Graphical abstract

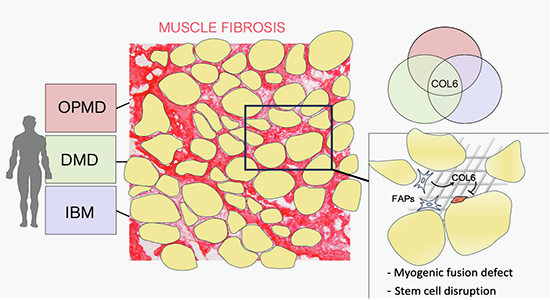

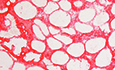

## Introduction

Muscular disorders encompass a diverse group of inherited and acquired diseases characterized by progressive muscle weakness and degeneration. These conditions differ in age of onset, affected muscle groups, and severity (Mercuri et al, 2019). Duchenne muscular dystrophy (DMD), a severe genetic myopathy caused by mutations in the X-linked chromosomal *DMD* gene, leads to widespread muscle degeneration from early childhood (Duan et al, 2021). In contrast, oculopharyngeal muscular dystrophy (OPMD) and inclusion body myositis (IBM) are late-onset myopathies typically manifesting after the age of 50, with more localized muscle involvement. A common feature of OPMD and IBM is pharyngeal muscle weakness, leading to dysphagia (Trollet et al, 2010; Greenberg, 2019). OPMD is caused by a triplet repeat expansion mutation, whereas IBM is an acquired inflammatory myopathy.

A hallmark of these myopathies is muscle fibrosis, characterized by an excessive deposition of extracellular matrix (ECM) components (Smith and Barton, 2018). Over time, ECM replaces muscle fibers, compromising tissue integrity and limiting the efficacy of therapeutic interventions.

Recent single-cell omics studies have provided insights into the cellular heterogeneity underlying dystrophic progression (Scripture-Adams et al, 2022; Saleh et al, 2022). While intercellular communication is recognized as a key factor in fibrosis, comprehensive analyses of ECM composition in human myopathies remain limited.

The ECM is a dynamic scaffold that regulates tissue development, structural integrity, and key cellular functions, such as proliferation, survival, and stem cell maintenance (Baghdadi et al, 2018; Osses and Brandan, 2002; Chrysostomou and Mourikis, 2024; Thorsteinsdóttir et al, 2011). In skeletal muscle, the ECM surrounding each muscle fiber is called the endomysium. This connective tissue can be divided into two distinct but interconnected compartments: the basal lamina and the interstitial ECM. The basal lamina is directly associated with the sarcolemma of each fiber and surrounds sublaminar muscle stem cells (MuSCs). It is connected, through a collagen VI network, to a reticular scaffold of interstitial ECM rich in collagen I and III (Sanes, 2003). The endomysium is highly deformable, and its composition undergoes dynamic remodeling during the regeneration process. Remodeling of the matrisome—the ensemble of ECM proteins and associated regulators, including structural components (core matrisome) and regulatory proteins (Hynes and Naba, 2012)—plays a central role in the apparition and maintenance of fibrosis. ECM proteins are secreted by a variety of cell types within muscle: MuSCs, macrophages, endothelial cells, and fibroadipogenic progenitors (FAPs) (Baghdadi et al, 2018; Schüler et al, 2022; Gratchev et al, 2001; Schnoor et al, 2008; Urciuolo et al, 2013). Among these, FAPs are the most prominent source of ECM proteins during homeostasis and regeneration (Zou et al, 2008; Uezumi et al, 2011; Chapman et al, 2017; Archile-Contreras et al, 2010; Kühl et al, 1984; Gatchalian et al, 1989). FAPs are involved in both physiological and pathological processes. However, their role in human skeletal muscle fibrosis remains poorly defined. Understanding quantitative and qualitative changes in the matrisome and the specific contribution of FAPs to the interstitial matrix composition is essential for deciphering the mechanisms driving skeletal muscle fibrosis and developing corrective strategies.

In this study, we performed a comparative proteomic analysis using liquid chromatography coupled to mass spectrometry (LC-MS/MS) of three distinct human myopathies involving fibrosis: DMD, OPMD, and IBM. By comparing affected fibrotic muscles from patients to those from healthy age-matched subjects, we identified matrisome alterations in each pathology. We defined different ECM remodeling signatures across myopathies, but a shared over-abundance of collagen VI. Furthermore, we isolated FAPs from patient biopsies and defined their secretion of key ECM proteins in vitro and in vivo. Beyond ECM remodeling, our results highlight the impact of FAP-driven ECM alterations on muscle regeneration. Indeed, in vivo experiments demonstrated that FAP-secreted ECM components, including Collagen VI and basal lamina proteins, influence muscle stem cell behavior and muscle tissue regenerative capacity. Silencing COL6 in patient FAPs was able to restore fusion capacity, directly linking pathological collagen VI deposition to regeneration failure. This work advances our understanding of ECM regulation in skeletal muscle diseases and uncovers collagen VI as a conserved effector of fibrosis and a potential therapeutic target across genetically and clinically distinct muscle diseases.

## Results

### Fibrotic FAPs share similar features across DMD, OPMD, and IBM

**Figure 1:**
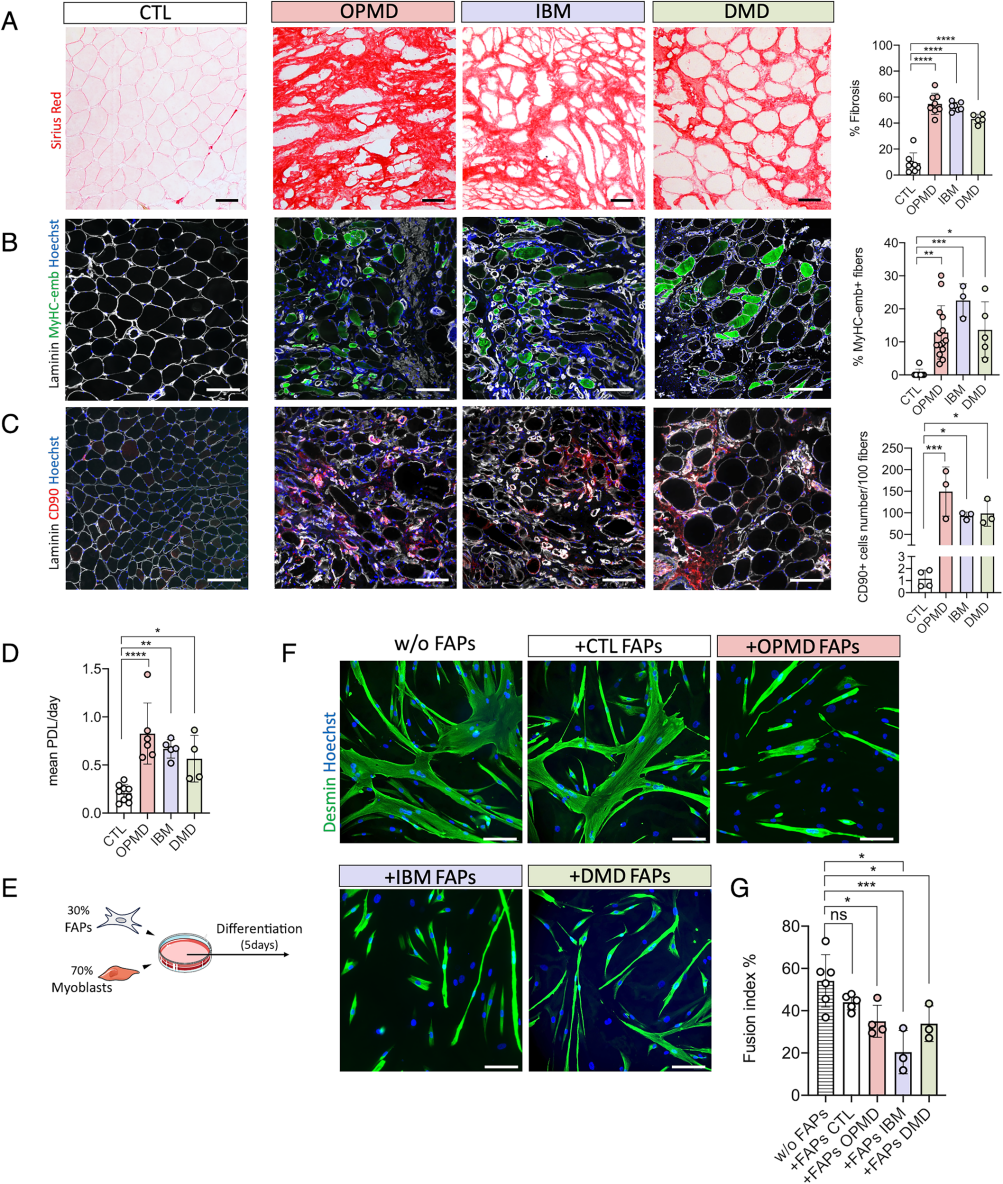
FAPs are abundant in affected muscles of OPMD, IBM, and DMD patients and impair myotubes fusion in vitro. (**A**) Sirius red coloration of human skeletal muscle biopsies from healthy CTL, OPMD, IBM, and DMD patients. Scale bar: 50 μm - Quantification of the percentage of fibrosis by Sirius red coloration (biological replicates *n* = 5–8 biopsies per condition). OPMD *P* < 0.0001, IBM *P* < 0.0001, DMD *P* < 0.0001. (**B**) Immunostaining of laminin (white), Hoechst (blue), and embryonic myosin heavy chain (MyHC-emb (MYH3)) (green). Scale bar: 50 μm. Quantification of the percentage of MyHC-emb positive fibers for each condition (biological replicates *n* = 3–14 biopsies per condition) OPMD *P *= 0.0018, IBM *P* = 0.0003, DMD *P* = 0.0107. (**C**) Immunostaining of laminin (white), Hoechst (blue), and CD90 (red) on human biopsies. Quantification of the number of interstitial cells positive for CD90 per 100 fibers (biological replicates *n* = 3–4 biopsies per condition). Scale bar: 50 μm. OPMD *P* = 0.0006, IBM *P* = 0.0148, DMD *P* = 0.0106. (**D**) In vitro proliferation rate expressed as the mean population doubling (PDL) /day of FAPs isolated from healthy CTL, OPMD, IBM, and DMD muscle biopsies (biological replicates *n* = 4–9 per condition). OPMD *P* < 0,0001, IBM *P* = 0.0024, DMD *P* = 0.0315. (**E**) Schematic representation of co-culture protocol; FAPs are co-cultured with myoblasts at a 30%/70% ratio for 5 days in differentiation medium. (**F**) Immunostaining of myotubes/FAPs co-cultures (Desmin, green; Hoechst, blue) after 5 days of differentiation, scale bar: 50 μm. (**G**) Quantification of the fusion index of the myoblasts, with or without FAPs (biological replicates *n* = 3–5 per condition). CTL ns; *P* = 0.3959, OPMD *P* = 0.0358, IBM *P* = 0.0007, DMD P = 0,0442. Data are presented as mean ± SD, with *P* values obtained by ordinary one-way ANOVA test followed by Tukey’s multiple comparisons test (*P < 0.1, **P < 0.01, ***P < 0.001, ****P < 0.0001). Source data are available online for this figure.

Muscle fibrosis is a hallmark of many myopathies, yet it remains unclear whether its cellular and molecular drivers are conserved across diseases or if each myopathy presents a distinct fibrotic signature. To investigate this, we analyzed three myopathies characterized by the presence of fibrosis: Duchenne Muscular Dystrophy (DMD), Oculopharyngeal Muscular Dystrophy (OPMD), and Inclusion Body Myositis (IBM). Biopsies from affected muscles exhibited severe fibrosis covering 38–69% of the total muscle section, compared to a median of 9% in healthy muscles (CTL) (Fig. 1A; Appendix Fig. S1). They also showed 13–23% of regenerating fibers positive for embryonic myosin heavy chain (MyHC-emb) (Fig. 1B), along with a high abundance of fibroadipogenic progenitors (FAPs), identified as interstitial cells expressing the CD90 marker (Fig. 1C; Appendix Fig. S2). To characterize the cellular actors of fibrosis and assess potential disease-specific differences, we isolated FAPs from fibrotic muscles of all three myopathies and analyzed their properties in vitro. FAPs isolated from affected muscles all displayed increased proliferation rates compared to those from non-fibrotic control muscles (Fig. 1D). Moreover, when co-cultured with myotubes (Fig. 1E), disease-derived FAPs impaired myogenic fusion, resulting in a reduced fusion index, an effect not observed in the presence of FAPs from control muscles (Fig. 1F,G).

### Mass spectrometry analysis reveals disease-specific and shared changes in the muscle proteome

**Figure 2:**
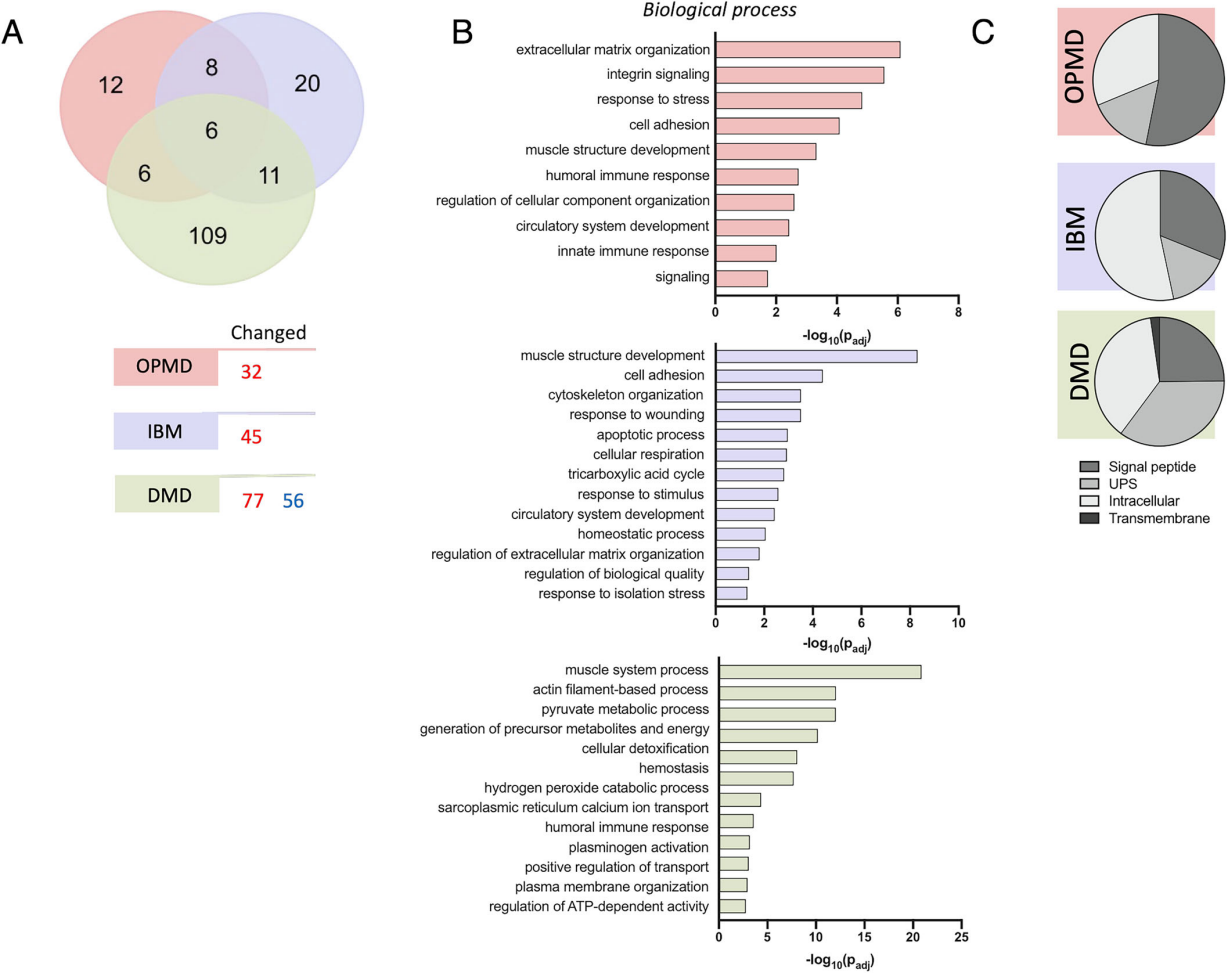
Proteomic analysis highlights different disease profiles and a shared ECM signature. (**A**) Venn diagram representing differentially expressed proteins shared between myopathies- Number of differentially expressed proteins identified among pathologies when compared to their age-matched control muscles (increased in red, decreased in blue). (**B**) Significantly altered proteins (Appendix Tables S1–S4) were subjected to a GO term analysis to identify associated biological processes using gProfiler. Bar charts of the top enriched terms for each comparison. (**C**) Representation of the repartition of proteins on their predicted secretion potential using the Outcyte prediction tool (UPS unconventional protein secretion). Source data are available online for this figure.

To investigate fibrosis-associated ECM remodeling in DMD, OPMD, and IBM, we analyzed the proteome of fibrotic muscle biopsies of each pathology by LC-MS/MS. Each patient’s muscles were compared to age-matched control muscles. In total, 172 proteins exhibited a significantly different abundance compared to control, with 32 proteins increased in OPMD, 45 in IBM, and 133 (77 increased, 56 decreased) in DMD samples (Fig. 2A; Appendix Tables S1–S4), suggesting more pronounced proteomic alterations in DMD when compared to age-matched healthy muscles. Of these, 25 proteins were shared between two of the three myopathies, while only six proteins were commonly differentially expressed across all three diseases (Fig. 2A; Appendix Table S5), highlighting both shared and disease-specific signatures. To characterize disease-specific proteomes, we performed gene ontology biological process enrichment analyses.

Among the most enriched terms, several were related to ECM organization, highlighting the expected increase in ECM protein abundance in pathological biopsies. Additional pathway-level analysis revealed molecular features reflecting the distinct pathophysiology of each disorder.

OPMD biopsies were mainly characterized by cytoskeletal and structural remodeling, with increased levels of proteins such as FLNA, SPTAN1, and ACTG1, whereas IBM showed enrichment in mitochondrial enzymes (ACO2, CS) and UQCRC1, together with chaperones (HSPA9, HSPA1A), indicating mitochondrial dysfunction and impaired proteostasis. DMD biopsies were characterized by alterations in cytoskeletal organization with changed levels of structural and contractile proteins such as vimentin (VIM), desmin (DES), and multiple myosin isoforms (MYH1, MYH7, MYH2), along with associated cytoskeletal components (TPM1, TPM2, ACTB). These changes were accompanied by chronic inflammatory and immune signaling, as reflected by the upregulation of complement and acute phase proteins (C3, SERPINA1, SERPINC1, ORM1) and immunoglobulins (IGKC, IGHG1, IGHM, IGHA1). In parallel, a shift toward glycolytic metabolism was observed, with increased abundance of key glycolytic enzymes (GPI, LDHA, PKM) pointing to ongoing regeneration and immune activation (Fig. 2B). Using Outcyte prediction tool (Zhao et al, 2019), we determined that among differentially expressed proteins, more than 68% for OPMD, 46% for IBM and 60% for DMD were annotated as secreted, conventionally or unconventionally (Fig. 2C; Appendix Tables S1–S4).

### FAPs drive core matrisome remodeling across myopathies

**Figure 3:**
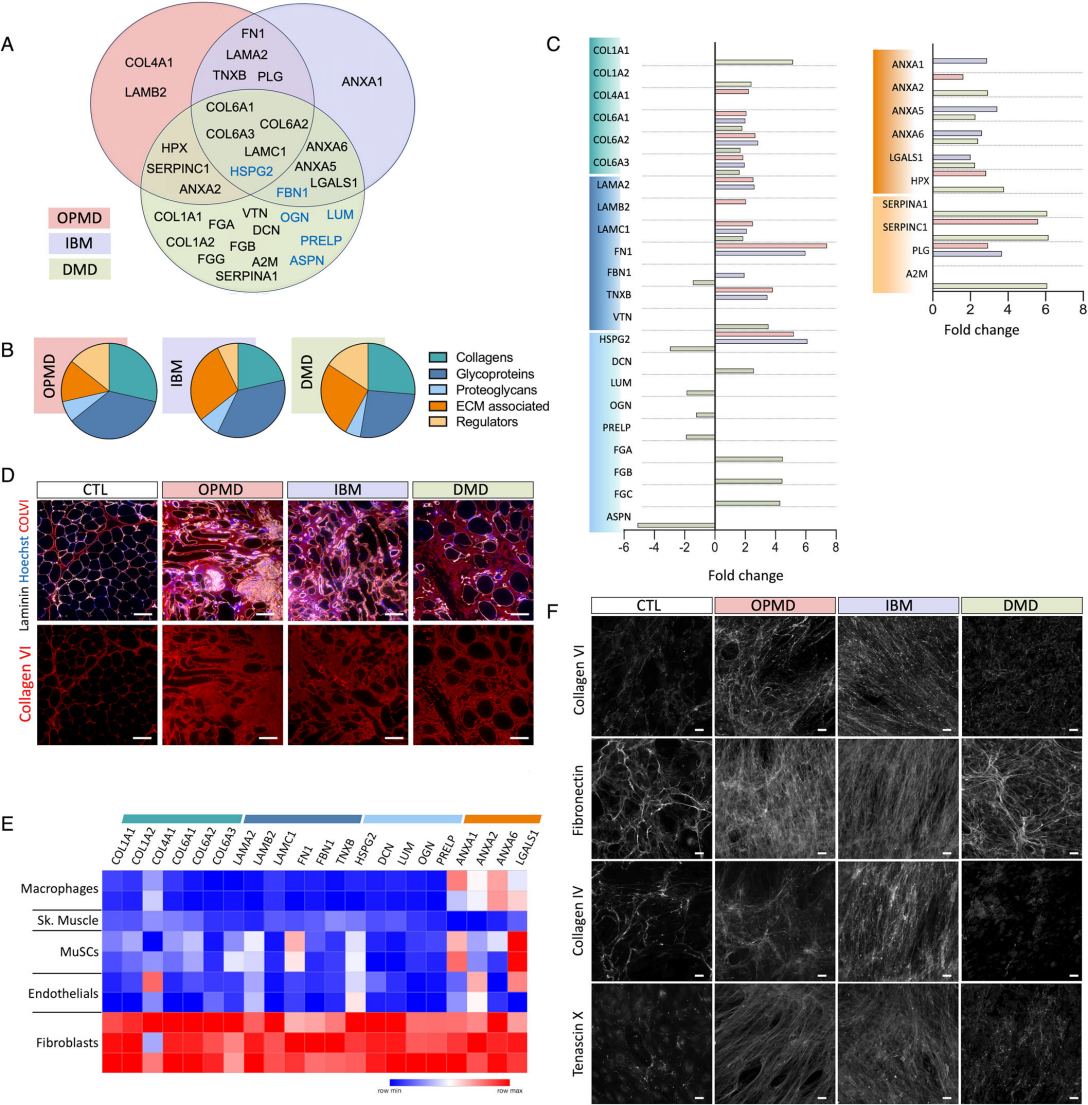
FAPs are the main actors responsible for matrisome alterations in myopathies. (**A**) Venn diagram representing matrisome proteins (from the matrisome database matrisomeproject.mit.edu) differentially expressed between pathologies and their age-matched control muscles. In blue, proteins decreased in DMD biopsies. (**B**) Representation of the repartition of the differentially expressed proteins for each group of ECM-related proteins, in each pathology. (**C**) Representation of the fold changes in abundance of differentially expressed ECM-associated proteins for each pathology (same color code as (**A**)) compared to their controls. Color code for proteins corresponds to the matrisome database protein groups shown in (**B**). (**D**) Immunostaining of laminin (white), Hoechst (blue), and Collagen alpha-3(VI) chain (red). Scale bar: 50 μm. (**E**) Heatmap of the expression of ECM proteins in several resident cell types within skeletal muscle on sc-RNA-seq dataset extracted from De Micheli et al 2020 (data ref: GSE143704). MuSCs muscle stem cells, Sk muscle skeletal muscle. (**F**) Immunostaining of Collagen alpha-3(VI) chain, fibronectin, Collagen IV, and Tenascin X on ECM produced by FAPs in vitro. Scale bar: 50 μm Source data are available online for this figure.

To further dissect fibrosis-associated ECM remodeling, we filtered the identified proteins using the human matrisome database (Naba et al, 2016), which categorizes ECM components into the “core matrisome” (collagens, glycoproteins, and proteoglycans) and “matrisome-associated” proteins (ECM regulators, ECM-affiliated proteins, and secreted factors). Between 18% and 43% of the differentially expressed proteins were ECM-related, with the majority belonging to the core matrisome (Fig. 3A,B; Appendix Tables S6–S9). We identified five core matrisome proteins consistently altered across all three myopathies: Collagen VI (COL6A1, COL6A2, and COL6A3), laminin subunit gamma 1 (LAMC1), and perlecan (HSPG2) (Fig. 3A,C). Notably, HSPG2 levels diverged showing decreased expression in DMD but increased expression in OPMD and IBM (Fig. 3C). Disease-specific ECM alterations included upregulation of collagen alpha-1(I) chain (COLIA1) and fibronectin 1 (FN1), the latter being significantly upregulated in both OPMD and IBM and exhibiting the highest fold change (Fig. 3C; Appendix Tables S6–S9). The major component of microfibrils, FBN1 was decreased in DMD while increased in IBM biopsies. ECM remodeling proteins were also dysregulated, with alpha-2-macroglobulin (A2M) and alpha-1-antitrypsin (SERPINA1) increased in DMD, antithrombin-III (SERPINC1) altered in both DMD and OPMD or plasminogen activation system (PLG) altered in both OPMD and IBM. Shared dysregulation of ECM degradation pathways suggests a common impairment in ECM turnover across diseases. Changes in annexins, which are involved in membrane repair, included ANXA1 increased in IBM, ANXA2 in DMD and OPMD, and ANXA5 and ANXA6 in DMD and IBM. DMD displayed a distinct ECM remodeling profile, particularly marked by the accumulation of small leucine-rich proteoglycans (SLRPs) such as decorin (DCN) and lumican (LUM). Notably, DMD samples showed reduced levels of ASPN, LUM, OGN, and PRELP, with DCN increased and biglycan (BGN) uniquely enriched in DMD samples. Basal membrane proteins such as COL4A1 and laminin subunit beta-2 (LAMB2) were specifically upregulated in OPMD (Fig. 3A,C). Immunofluorescence analysis (Fig. 3D) further validated the presence of a high level of Collagen VI across all three myopathies. Altogether, these findings reveal a combination of shared ECM remodeling features and distinct disease-specific signatures across myopathies. To further delineate the cellular sources of ECM proteins, we analyzed a publicly available human single-cell RNA sequencing (scRNA-seq) dataset De Micheli et al (Data ref: (De Micheli et al, 2020). This analysis confirmed that FAPs are the predominant source of the identified ECM proteins, including collagens, glycoproteins and basal lamina proteins (Fig. 3E). To investigate their role in ECM production, FAPs isolated from control, DMD, OPMD, and IBM muscle samples were cultured in vitro and found to actively secrete key ECM components, forming networks of collagen VI fibrils, glycoproteins (Tenascin X, Fibronectin) and basal lamina components (Collagen IV) (Fig. 3F).

### FAPs-driven ECM modifications impact muscle regeneration and stem cell niche maintenance

**Figure 4:**
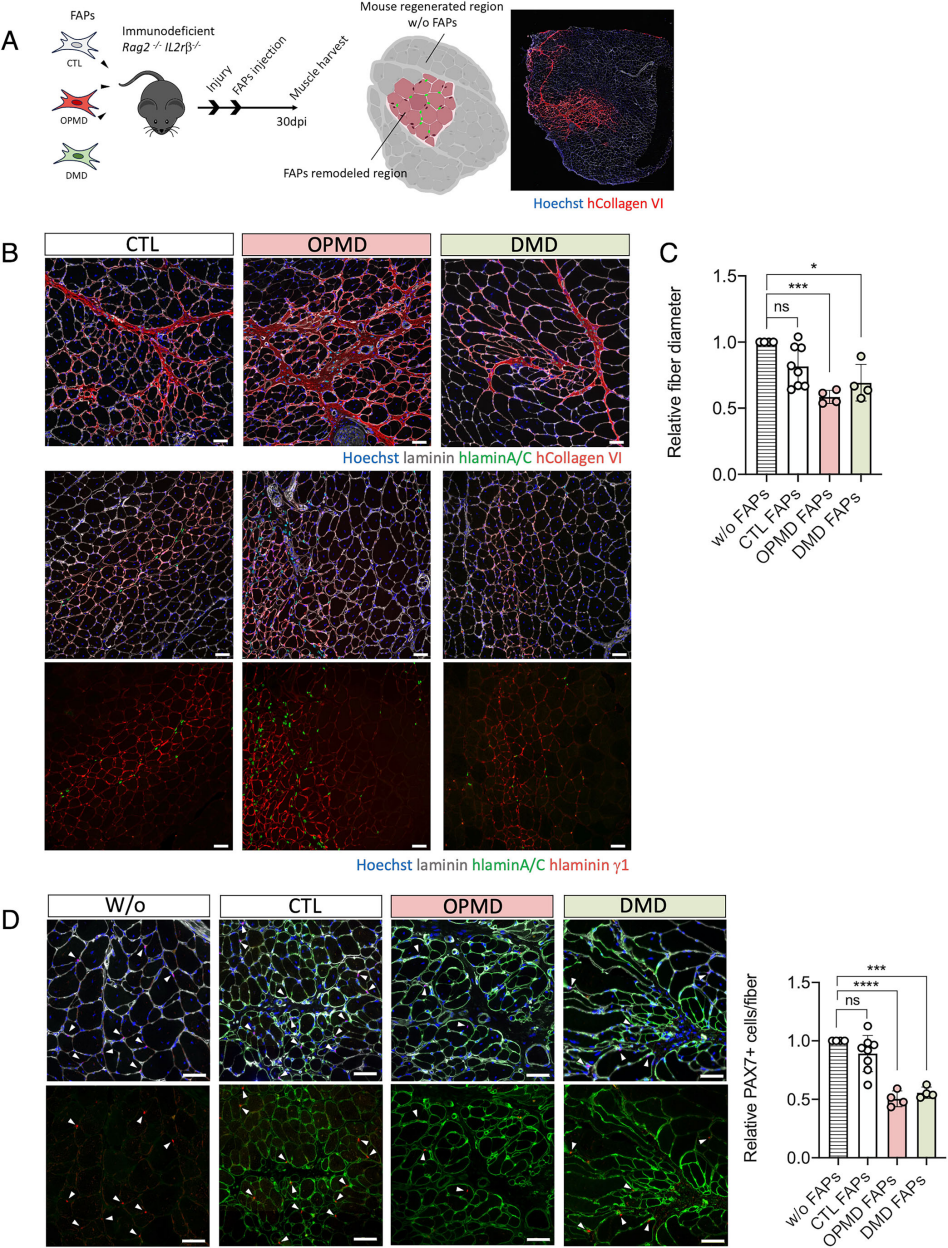
FAPs-driven ECM modifications impact muscle regeneration and stem cell niche maintenance. (**A**) Experimental setup of FAPs transplantation. FAPs from healthy CTL, OPMD, or DMD were injected into the TA of immunodeficient mice after injury. TA were retrieved and analyzed 30 days post injections. For each analysis, the portion of the TA containing human cells (FAPs-remodeled regions) was compared to the regenerated mouse region without any human FAPs. (**B**) Immunostaining of laminin (white), Hoechst (blue), and human-specific Collagen alpha-3(VI) chain (red) or human-specific laminin gamma 1 (red). Scale bar: 50 μm. (**C**) Quantification of minimal feret fiber diameter in the muscle region remodeled by FAPs normalized to the non-injected muscle area. (biological replicates *n* = 4–8 per condition) CTL ns; *P* = 0.1008, OPMD *P* = 0.0009, DMD *P* = 0.0115. (**D**) Immunostaining of laminin (white), Hoechst (blue), Pax7 (red), and human-specific laminin gamma 1 (green). Arrows point to Pax7-positive cells. Scale bar: 50 μm. Quantification of Pax7+ cells per fiber normalized to the non-injected muscle area (biological replicates *n* = 4–8) CTL ns; *P* = 0.3960, OPMD *P* < 0.0001, DMD *P* = 0.0002. Data are presented as mean ± SD, with *P* values obtained by ordinary one-way ANOVA test followed by Tukey’s multiple comparisons test (*P < 0.1, **P < 0.01, ***P < 0.001, ****P < 0.0001). Source data are available online for this figure.

To assess the functional impact of disease-derived FAPs on muscle regeneration, we injected human FAPs isolated from fibrotic muscle biopsies (from OPMD and DMD muscles) into the regenerating *tibialis anterior* (TA) muscle of Rag2^−/−^ Il2rb^−/−^ immunodeficient mice (Fig. 4A). Four weeks post-injection, following complete regeneration, we detected human FAPs in the interstitial space between newly formed muscle fibers using human-specific antibodies (Fig. 4B). All FAPs actively secreted ECM proteins, including collagen VI within the interstitial matrix surrounding muscle fibers, and laminin γ1 in the basal lamina of newly formed fibers (Fig. 4B). In the injected muscle, we specifically analyzed regions where human FAPs were present and compared them to adjacent regions lacking FAPs. Fiber diameter analysis revealed that muscle fibers in fibrotic FAP-rich regions were significantly smaller than those in the rest of the TA (Fig. 4C). Furthermore, quantification of endogenous Pax7+ cells showed a reduced number of muscle stem cells (MuSCs) in these fibrotic FAP-remodeled regions, suggesting that FAP-derived ECM may impair MuSCs quiescence and niche maintenance during regeneration (Fig. 4D).

### Collagen VI plays a key role in myogenic fusion

**Figure 5:**
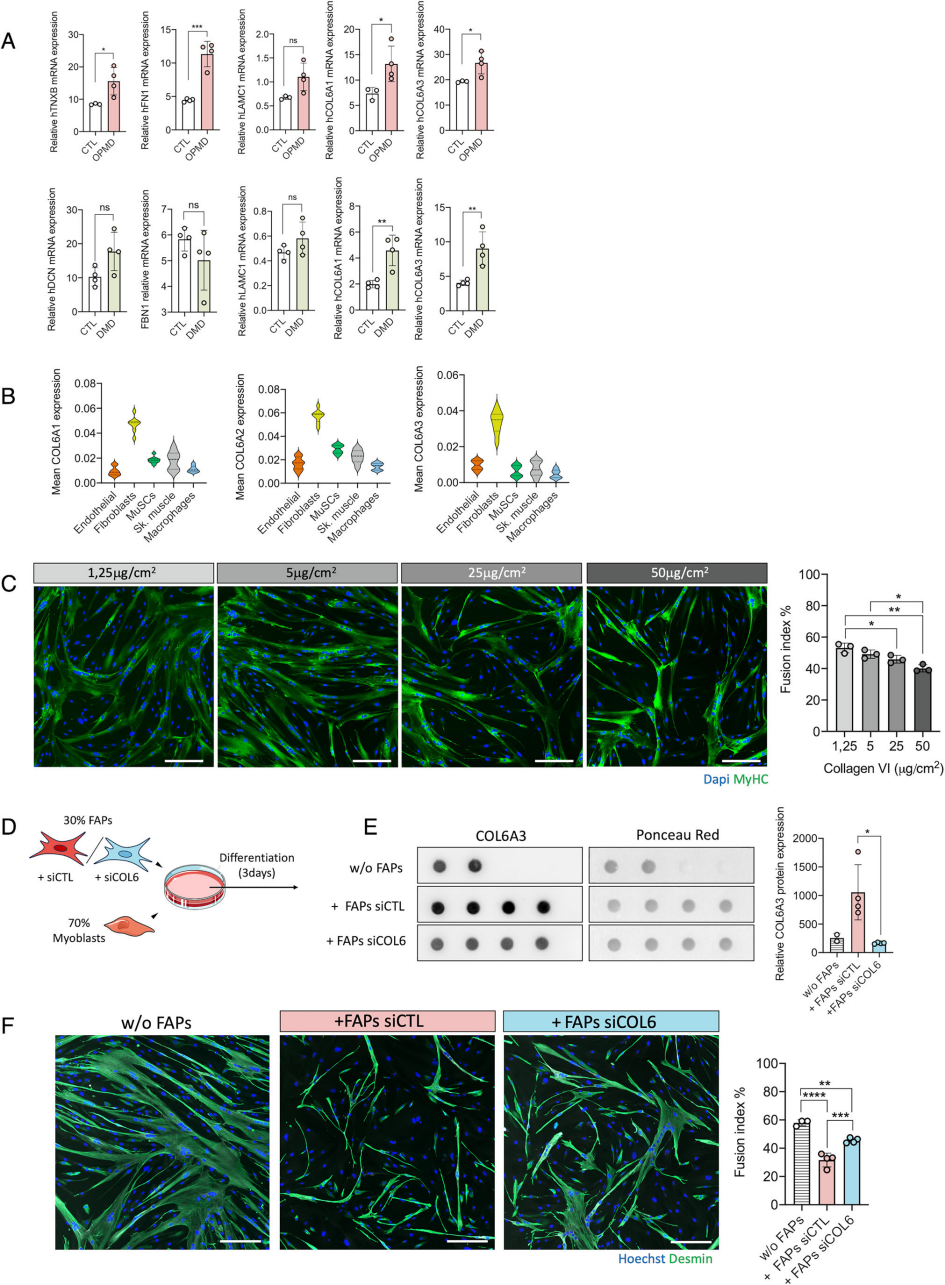
Collagen VI is involved in the impairment of myoblast fusion in vitro. (**A**) RT-qPCR quantification of ECM genes normalized to the expression of human-specific B2M (biological replicates *n* = 3–4) OPMD - hTNXB *P* = 0.0366, hFN1 *P* = 0.0004; hLAMC1 ns *P* = 0.0519, hCOL6A1 *P* = 0.0421 hCOL6A3 *P* = 0.0357. DMD - hDCN ns *P* = 0.0551, hFBN1 ns *P* = 0.240 hLAMC1 ns *P* = 0.1594 hCOL6A1 *P* = 0.0051 hCOL6A3 *P* = 0.0067. Data are presented as mean ± SD, with *P* values obtained by *t* test. (**B**) Expression of COL6A1, COL6A2, and COL6A3 in several resident cell types within skeletal muscle on sc-RNA-seq dataset extracted from De Micheli et al 2020 (Data ref: GSE143704) (biological replicates *n* = 8). (**C**) Immunostaining of myotubes (Myosin Heavy Chain, green; Hoechst, blue) after 5 days of differentiation on different concentrations of collagen type VI coatings. Quantification of the fusion index (technical replicates *n* = 3 per condition). Scale bar: 200 μm. 1.25 vs 5 ns *P* = 0.3288; 1.25 vs 25 *P* = 0.0418; 1.25 vs 50 *P* = 0,0014; 5 vs 50 *P* = 0.0129. Data are presented as mean ± SD, with *P* values obtained by ordinary one-way ANOVA test followed by Tukey’s multiple comparisons test (*P < 0.1, **P < 0.01, ***P < 0.001, ****P < 0.0001). (**D**) Schematic representation of the co-culture experiment. Myoblasts were seeded with OPMD FAPs previously transfected for 24 h with a siRNA against COL6A3 (siCOL6) or a non-targeting siRNA (siCTL) and differentiated for 3 days. (**E**) Quantification of Collagen VI alpha-3protein by dot blot on supernatants. The collagen signal was normalized on Ponceau red staining. (technical replicates *n* = 2–4) FAPs siCOL6 *P *= 0.0132. Data are presented as mean ± SD, with *P* values obtained by ordinary one-way ANOVA test followed by Tukey’s multiple comparisons test (*P < 0.1, **P < 0.01, ***P < 0.001, ****P < 0.0001). (**F**) Immunostaining of myoblasts/FAPs co-cultures (Desmin, green; Hoechst, blue) after 3 days of differentiation. Scale bar: 200 μm. Quantification of the fusion index of myoblasts alone, or with FAPs transfected with COL6A3 siRNA or a non-targeting siRNA (technical replicates *n* = 4 per condition). FAPs siCTL *P* < 0.0001; siCOL6 *P* = 0.0032; siCTL vs siCOL6 *P* = 0.0010. Data are presented as mean ± SD, with *P* values obtained by ordinary one-way ANOVA test followed by Tukey’s multiple comparisons test (*P < 0.1, **P < 0.01, ***P < 0.001, ****P < 0.0001). Source data are available online for this figure.

Collagen VI was consistently upregulated in all three pathologies (Fig. 3A) and actively secreted by FAPs in vitro (Fig. 3F). Using human-specific primer sequences, we analyzed the expression of several ECM candidates in FAPs injected into regenerating TA muscles of mice. Some mRNAs were significantly more highly expressed by disease-derived FAPs compared to controls. Among these, *Col6a1* and *Col6a3* were upregulated in FAPs from both pathologies in this regenerative context (Fig. 5A). Moreover, public single-cell transcriptomic data show that *Col6a1, Col6a2* and *Col6a3* expressions are markedly higher in FAPs compared to other muscle-resident cell types (Fig. 5B). To further investigate the impact of FAP-derived ECM on myogenic potential, we coated culture wells with increasing concentrations of collagen VI and assessed its effect on myogenic differentiation, as measured by fusion index in vitro. We found that higher concentrations of collagen VI significantly reduced the fusion ability of human muscle cells in a dose-dependent manner (Fig. 5C). To further directly assess the role of COLVI secreted by FAPs in the regulation of the fusion index, we silenced *COL6* expression specifically in FAPs isolated from OPMD patients, using siRNA (Fig. 5D,E). As FAPs isolated from the three pathologies present similar in vitro characteristics, we decided as a proof of principle to use FAPs from OPMD patients. These siRNA-treated-FAPs were then co-cultured with myotubes for 3 days, and myogenic fusion was evaluated. The level of secreted COLVI expression, assessed by dot blot, was restored to the baseline level observed in wells without FAPs (Fig. 5E). Notably, knockdown of *COL6* in FAPs (siCOL6) significantly improved the fusion index compared to co-cultures with FAPs transfected with a control siRNA (siCTL), indicating that FAP-derived COLVI contributes to the impaired myogenic fusion observed in disease conditions (Fig. 5F).

## Discussion

The ECM plays a central role in maintaining muscle architecture and regulating regeneration, yet its composition and remodeling dynamics in human myopathies remain poorly defined. While fibrosis, defined as an excessive deposition of ECM, is a common feature across many muscular dystrophies, most studies have focused on inflammatory or myogenic aspects, leaving the matrisome relatively understudied. How ECM remodeling differs—or converges—across distinct fibrotic muscle diseases has not been systematically addressed. Here, we provide a comparative analysis of ECM composition in DMD, OPMD, and IBM. By integrating proteomic profiling with functional assays, we identify both shared and disease-specific ECM signatures and demonstrate that FAP-mediated ECM remodeling contributes to impaired muscle regeneration through disruption of the stem cell niche. To minimize muscle-specific variability, each condition was compared to the corresponding muscle type from age-matched unaffected individuals, enabling the identification of robust disease-associated proteomic changes within the fibrotic environment.

A specific characteristic of the ECM is its capacity to undergo dynamic remodeling, balancing synthesis and degradation in a tightly regulated manner (Hynes, 2009). In all three muscle diseases—DMD, OPMD, and IBM—we observed a common shift toward impaired ECM turnover, primarily driven by increased expression of protease inhibitors, as illustrated by the examples described below. Protease inhibitors such as A2M and SERPINA1 have been highlighted in DMD (Capitanio et al, 2020), and our data also reveal elevated levels of other serine protease inhibitors like SERPINC1 in both DMD and OPMD. This pattern of broad protease inhibition suggests a common mechanism limiting ECM degradation in these muscle diseases. Additionally, the plasminogen (PLG) system, a key player in ECM remodeling through fibrin and fibronectin degradation and activation of protease (Rahman and Krause, 2020), was elevated in OPMD and IBM, and detected in DMD compared to controls. Together, these changes likely contribute to pathological ECM accumulation and fibrosis, reflecting a shared disruption of ECM homeostasis across all three diseases.

This impaired ECM turnover also intersects with inflammatory processes, which are a hallmark of DMD, IBM, and OPMD. In these diseases, the accumulation of ECM-derived DAMPs (damage-associated molecular patterns) and inflammatory mediators contributes to chronic inflammation and fibrosis. Notably, increased annexins (ANXA1, ANXA2) point to active membrane repair mechanisms and a potential role in sustaining fibrotic signaling. Moreover, the presence of pro-inflammatory molecules such as fibrinogen—already elevated in the ECM of DMD and OPMD—can activate macrophages and promote TGFβ signaling, further exacerbating tissue fibrosis (Vidal et al, 2008).

Both OPMD and IBM are late-onset disorders characterized by mitochondrial dysfunction (Gambelli et al, 2004; Greenberg, 2019) and protein inclusions. Although OPMD and IBM differ in their genetic and pathological origins—PABPN1 mutations in OPMD versus the inflammatory and degenerative processes in IBM—they share some overlapping clinical features, including dysphagia due to preferential involvement of the cricopharyngeal muscle. Following matrisome-specific filtering, our analysis revealed that OPMD and IBM display distinct ECM remodeling signatures, including differences in collagen composition and in the expression of glycoproteins such as fibrillin-1 (FBN1). Given its role as a structural scaffold regulating the sequestration and activation of growth factors, including TGFβ (Li et al, 2024), differential FBN1 levels may contribute to disease-specific modulation of this pathway. While FBN1 levels remained unchanged in OPMD, they were reduced in DMD and increased in IBM, suggesting a distinct effect on TGFβ bioavailability and signaling. These observations reinforce the idea that ECM composition can differentially influence profibrotic signaling across myopathies and thereby contribute to disease progression.

In contrast to OPMD and IBM, DMD is primarily driven by dystrophin deficiency, leading to sarcolemma destabilization, repeated fiber damage, and chronic inflammation (Petrof et al, 1993). Consistent with this, our proteomic data showed increased expression of cytoskeletal proteins (such as vimentin and various myosin isoforms) and glycolytic enzymes (GPI, LDHA, PKM), reflecting cytoskeletal reorganization and metabolic adaptation to ongoing muscle regeneration. This is further supported by elevated levels of pro-inflammatory mediators like fibrinogen (Murphy and Ohlendieck, 2016; Capitanio et al, 2020), which may contribute to the amplification of fibrotic signaling and tissue scarring. ECM structure and stiffness are also shaped by its proteoglycan content. Several small leucine-rich proteoglycans (SLRPs)—including ASPN, BGN, DCN, LUM, OGN, and PRELP—bind collagen fibrils to protect them from collagenase cleavage (Imai et al, 1997; Geng et al, 2006; Kalamajski and Oldberg Å, 2009). Notably, DMD samples showed reduced levels of ASPN, LUM, OGN, and PRELP, with DCN increased and BGN uniquely enriched in DMD samples. These imbalances could destabilize collagen architecture and alter matrix stiffness, which directly influences muscle cell fate (Gilbert et al, 2010; Moyle et al, 2020) and modulates FAP differentiation (Loomis et al, 2022). Thus, shared ECM remodeling defects in DMD, OPMD, and IBM may converge on a common mechanism: reduced matrix turnover coupled with altered stiffness, reinforcing fibrosis, impairing stem cell activation, and disrupting regenerative signaling.

Beyond its impact on fibrosis, ECM composition and stiffness also directly affect muscle stem cell (MuSC) behavior, particularly within the specialized microenvironment of the basal lamina, which is rich in collagens and laminins (Relaix and Zammit, 2012). This niche governs MuSC quiescence, activation, and self-renewal, making it essential for muscle homeostasis and regeneration (Schüler et al, 2022). Specific ECM components have been shown to exert opposing effects on MuSC fate: some, such as laminin 211, promote quiescence (Baghdadi et al, 2018), while others, like laminins containing the α5 chain, support proliferation (Rayagiri et al, 2018). FAPs are often considered the major producers of ECM components in skeletal muscle, although they are not the only ECM-secreting cells within muscle (Zou et al, 2008; Uezumi et al, 2011). During regeneration, other cell types like MuSCs, macrophages, or endothelial cells have been shown to secrete ECM components (Baghdadi et al, 2018; Schüler et al, 2022; Gratchev et al, 2001; Schnoor et al, 2008; Urciuolo et al, 2013).

Our results show a deleterious impact from disease-derived FAPs on MuSCs, revealed by a decrease in myoblast fusion index in vitro, while no effect is visible in the presence of control FAPs. FAPs are essential to muscle, and their absence leads to altered regeneration in mice (Wosczyna et al, 2019). In mice, FAPs have been shown to promote myogenesis (Joe et al, 2010; Yu et al, 2024). However, some studies do not observe a positive effect of FAPs on myoblasts differentiation and fusion (Mázala et al, 2023). Interestingly, we did not observe a positive effect on myoblasts fusion when co-cultured with FAPs isolated from control muscles. These discrepancies between studies could be explained by species differences, variations in culture conditions including the medium used, the duration of differentiation, the ratio of each cell type in co-culture, or the activation state of the FAPs.

Our findings demonstrate that disease-derived FAPs significantly remodel the ECM during regeneration, with consequences for MuSC maintenance. When human FAPs from fibrotic muscle biopsies were transplanted into regenerating mouse muscle, they integrated into the interstitial space and actively secreted ECM proteins, including collagen VI and laminin subunits. Importantly, regions enriched in fibrotic FAPs displayed smaller muscle fibers and a reduced number of Pax7+ MuSCs, suggesting that excessive or specific ECM deposition impairs MuSC quiescence, niche integrity, and ongoing regeneration. In the proteomic data from all three diseases, we observed elevated levels of basal lamina components such as LAMA2 (merosin), LAMB2, LAMC1, and COL4A1. These alterations may disrupt MuSC positioning and niche integrity, contributing to impaired MuSC behavior and defective regeneration observed in muscle biopsies from OPMD (Gidaro et al, 2013), DMD (Cardone et al, 2023), and IBM patients (Wanschitz et al, 2013).

Among the ECM components identified in our study, five were consistently increased across all disease conditions. Laminin γ1, a subunit of several heterotrimeric laminin isoforms, such as laminin-211, is a key component of the basal lamina. In contrast, perlecan displayed a distinct profile, with decreased levels in DMD biopsies, possibly reflecting its role as an anchor to cell surface receptors like dystroglycan (Peng et al, 1998), which is destabilized in the absence of dystrophin. Among these shared alterations identified in our study, collagen VI emerged as a key player for further investigation, as all three α chains composing the final beaded microfilaments of the collagen VI protein were found to be increased across all pathological conditions. Collagen VI was secreted by FAPs both in vitro and in vivo, and functionally, its accumulation impaired myogenic differentiation, as shown by reduced fusion indices in myoblast cultures exposed to high COLVI levels. Previous studies carried out in mice highlight the finely regulated role of collagen VI in modulating the behavior of satellite cells. A complete deficiency of collagen VI impairs MuSC self-renewal following injury (Urciuolo et al, 2013), emphasizing its importance in maintaining a functional niche and promoting proper muscle regeneration. Indeed, supplementation with collagen VI—via mesenchymal stem cells expressing collagen VI—enhances muscle regeneration and maturation in Col6ko mouse models (Takenaka-Ninagawa et al, 2021). Elevated levels of collagen VI, by inhibiting the canonical Wnt pathway (which acts as a soluble ligand), delay early myogenic differentiation (Metti et al, 2024). Our experiments using cultured human myoblasts appear to reflect a similar pattern. Recently, Mohassel et al (Mohassel et al, 2025) showed that during murine muscle regeneration, collagen VI-deficient matrix is not able to regulate the dynamic TGF-β bioavailability, impairing this regulatory mechanism.

These findings raise important considerations regarding the therapeutic targeting of collagen VI. Notably, previous work in γ-sarcoglycan-deficient mice showed that reducing collagen VI ameliorates muscle pathology without improving muscle function (De Greef et al, 2016). Together with our data, this suggests that modulating collagen VI levels may not readily translate into functional benefit. Importantly, our identification of collagen VI as a shared alteration across the three myopathies studied here supports its relevance as a common disease-associated ECM component. However, therapeutic strategies will likely require precise tuning rather than simple inhibition, and their efficacy may still depend on disease context and stage.

Taken together, our data reveal that FAP-mediated ECM remodeling—particularly the aberrant deposition of collagens and laminins—profoundly alters the MuSC niche, impairing stem cell positioning, quiescence, and regenerative potential. These findings reposition the ECM not merely as a passive structural scaffold but as an active, disease-modified microenvironmental cue with instructive roles in stem cell fate decisions. By delineating both shared and disease-specific ECM alterations, our study provides a conceptual framework for understanding how niche dysfunction contributes to regeneration failure in fibrotic myopathies. We uncovered Collagen VI as a conserved fibrotic-associated signal disrupting muscle regeneration across distinct human myopathies. Our findings further underscore the complexity of ECM remodeling in fibrotic muscle diseases: while mechanisms such as increased collagen deposition and protease inhibition are commonly observed, distinct disease-specific ECM signatures reveal divergent pathogenic processes. Importantly, these signatures may offer novel targets for therapeutic strategies aimed at restoring MuSC function and muscle homeostasis. Future studies should now focus on mechanistically linking specific ECM alterations to MuSC dysfunction and evaluating targeted interventions in relevant disease models.

## Methods

**Table:** Reagents and tools table

Reagent/resource	Reference or source	Identifier or catalog number
**Experimental models**		
Rag2-/-Il2rb−/− C57BL/6 (*M.* *musculus*)	In House	N/A
**Antibodies**		
Laminin (Rabbit polyclonal) 1:400	Dako	Z0097
Embryonic Myosin Heavy Chain (Mouse IgG1) 1:4	Developmental Studies Hybridoma Bank (DSHB)	F1.652
CD90 (Mouse IgG1) 1:50	BD Pharmingen	555593
Desmin(Mouse IgG1, clone D33) 1:50	Dako	M0760
Collagen alpha-3(VI) (Mouse IgG1, clone 3C4) 1:500	Sigma-Aldrich	MAB1944
Lamin A/C (Mouse IgG2b, clone 636) 1:400	Santa Cruz Biotechnology	sc-7292
Myosin Heavy Chain (Mouse IgG2b, clone MF20) 1:20	Developmental Studies Hybridoma Bank (DSHB)	
Laminin gamma 1 (Mouse IgG2a, clone 2E8) 1:20	Developmental Studies Hybridoma Bank (DSHB)	
Pax7 (Mouse IgG1) 1:20	Developmental Studies Hybridoma Bank (DSHB)	
Collagen IV (Mouse IgG1, clone M3F7) 1:50	Developmental Studies Hybridoma Bank (DSHB)	
Tenascin X (Sheep, polyclonal) 1:15	BioTechne	AF6999
Fibronectin (Mouse IgG1) 1:100	Novocastra	NCL-Fib
Sheep anti-mouse HRP	Jackson IR	515-035-062
**Oligonucleotides and other sequence-based reagents**		
TNXB	Eurogentec	F:CTGGAATCCGACCACAGATAC R:TGGGAGGTTCTCAAGGCTTCC
FN1	Eurogentec	F:CTTGCCTGGGAGAAGGCAG R:TCGATGTGGTCTGCACAGAG
LAMC1	Eurogentec	F:CCAGCCCGGCATCACTGGT R:TCTGCATTCACAGCGACCATC
DCN	Eurogentec	F:GCTCTCCTACATCCGCATTGCT R:GTCCTTTCAGGCTAGCTGCAT
FBN1	Eurogentec	F: CCGGTGTGAGTGCAACAAAG R: ATGTCTCGGCATTCTGTCCG
COL6A1	Eurogentec	F:GCCTTCCTGAAGAATGTCACCG R: CACGCTGGCGGAGCCGTCCA
COL6A3	Eurogentec	F: GTGTTGCAGCCTCTACCGAG R: CGTCCAGGTAGTCAACCAGC
B2M	Eurogentec	F: CTCTCTTTCTGGCCTGGAGG R:TGCTGGATGACGTGAGTAAACC
**Chemicals, enzymes, and other reagents**		
SYBR Green Master Mix	Applied Biosystems	4309155
Red Ponceau	Sigma-Aldrich	09189
Notexin	Latoxan	L8104
Tragacanth gum	Sigma-Aldrich	G1128
Sirius red Direct red 80	Sigma-Aldrich	365548
DMEM	Life Technologies	61965-026
Fetal bovine serum	Serana	S-FBS-CO-015
199 medium	Life Technologies	41150-020
DPBS	Life Technologies	20012-019
Fetuin	Life Technologies	10344-026
bFGF	Invitrogen	PHG0026
EGF	Life Technologies	PHG0311
Insulin	Life Technologies	91077 C
Gentamicin	Life Technologies	15750-037
Clarity max ECL substrate	Bio-Rad	1705062
Paraformaldehyde 32%	Electron Microscopy Science	15714
Collagen VI	Rockland	009-001-108
Lipofectamine RNAiMAX	Invitrogen	10601435
ON-TARGETplus siRNA human COL6A3 SMARTPool	Dharmacon	L-003646-00
MACs CD56 microbeads	Miltenyi Biotec	130-050-401
ON-TARGETplus Non-targeting pool	Dharmacon	D-001810-10
**Software**		
GraphPad Prism	GraphPad	Version 8.4.0
Zen Blue 2.0 software	Zeiss	Version 2.0
ImageJ software		Version 1.0
**Other**		
Cryostat	Leica	CM1850
Leica DMR microscope equipped with a Nikon DS-Ri1 camera	Leica	
Chemidoc MP imaging system	Bio-Rad	
Axio Observer7 microscope	Zeiss	

### Muscle biopsy samples

Human muscle samples from healthy controls (CTL), oculopharyngeal muscular dystrophy (OPMD), Duchenne muscular dystrophy (DMD), and inclusion body myositis (IBM) patients (see Table 1) were obtained anonymously after informed consent, via the Myobank-AFM, affiliated with EurobioBank, in accordance with European recommendations and French legislation (authorization AC-2019-3502).

**Table 1:** Human biopsies used in the study.

Status	Muscle	Number
CTL	Cricopharyngeal	4
	Dorsal	1
	Deltoid	1
	Paraspinal	5
	Quadriceps	6
	Tensor fascia lata	1
	Sternocleidomastoid	9
OPMD	Cricopharyngeal	13
IBM	Cricopharyngeal	11
DMD	Paraspinal	8
	Quadriceps	1
	Spinal	1

### Muscle histology

Fresh human muscle biopsy samples (OPMD, IBM, DMD, and CTL) were mounted on tragacanth gum (6% in water; Sigma-Aldrich) placed on a cork support and snap frozen in liquid nitrogen-cooled isopentane. Transverse serial frozen cryosections (5μm thick) obtained with a cryostat (Leica CM1850) were processed for staining. Sirius red (SR) coloration was obtained by first fixing the sections with 4% paraformaldehyde (PFA), followed by an incubation in a 0.3% solution of Sirius red at room temperature (RT) for 1 h. Sections were imaged by light microscopy (Leica DMR microscope equipped with a Nikon DS-Ri1 camera). The percentage of fibrosis within each biopsy was assessed on SR-stained cryosections from 3 to 5 fields per sample, using ImageJ software, and expressed as a percentage of the total area analyzed. Cryosections were used for subsequent proteomic and immunofluorescence analyses.

### Proteomic sample preparation

The preparation of tissue specimens and subsequent analysis of extracted proteins by bottom-up proteomics was performed as previously described (Dowling et al, 2020). Frozen cryosections (for a total amount of 250 μm) from human muscle biopsy samples (OPMD, IBM, DMD, and CTL) were homogenized using lysis buffer containing 4% (w/v) sodium dodecyl sulfate, 0.1 M dithiothreitol, and 100 mM Tris-Cl, pH 7.6, and a protease inhibitor cocktail (Thermo Scientific #A32953). Homogenization was carried out using a handheld homogenizer (Kimble Chase, Rockwood, TN, USA), followed by a brief water bath sonication. Samples were then heated for 3 min (min) at 95 °C and centrifuged at 16,000 ×  *g* for 5 min. The protein-containing supernatant was extracted and used for subsequent proteomic studies. The Pierce 660 nm Protein Assay system was used to determine protein concentration. Extracted proteins were further processed for mass spectrometric analysis. Samples were mixed with 8 M urea, 0.1 M Tris pH 8.9 in Vivacon 500 spin filter units and centrifuged at 14,000× *g* for 15 min. Samples were processed with the Filter-aided sample preparation (FASP) method (Wiśniewski et al, 2009), buffer switching, and protein trypsination prior to mass spectrometric peptide analysis.

### Mass spectrometry and proteomic data analysis

Protein samples were analyzed using a Q-Exactive mass spectrometer (Thermo Scientific) coupled to a Dionex RSLCnano (Thermo Scientific). Peptides were separated using a 2–40% gradient of acetonitrile (A: 0.1% FA, B: 80% acetonitrile, 0.1% FA) over 65 min at a flow rate of 250 nl/min. The Q Exactive was operated in the data-dependent mode, collecting a full MS scan from 300 to 1650 *m/z* at 70 K resolution and an AGC target of 1e6. The 10 most abundant ions per scan were selected for MS/MS at 17.5 K resolution and AGC target of 1e5 and intensity threshold of 1 K. Maximum fill times were 10 ms and 100 ms for MS and MS/MS scans, respectively, with a dynamic exclusion of 60 s. Samples were analyzed using 25 NCE (normalized collisional energy) with 20% stepped energy (Murphy et al, 2019). For quantitative analysis, samples were evaluated using MaxQuant (v1.5.2.8) and the Andromeda search engine to identify the detected features against the UniProtKB/SwissProt database (Homo sapiens). The following search parameters were used: (i) first search peptide tolerance of 20 ppm, (ii) main search peptide tolerance of 4.5 ppm, (iii) cysteine carbamidomethylation set as a fixed modification, (iv) methionine oxidation set as a variable modification, (v) a maximum of two missed cleavage sites, and (vi) a minimum peptide length of seven amino acids. The false discovery rate (FDR) was set to 1% for both peptides and proteins using a target-decoy approach. Perseus v.1.5.6.0 was used for data analysis, processing, and visualization (Gargan et al, 2020).

### Proteomic data analysis

Cricopharyngeal (CP) muscles from OPMD (*n* = 3) and IBM patients (*n* = 4) were compared to CP muscle from age-matched healthy subjects (*n* = 4). DMD paraspinal (PS) muscles (*n* = 4), were compared to PS from age-matched healthy subjects (see Table 2). Enriched terms for biological processes from the list of differentially expressed proteins were obtained using g: Profiler (Kolberg et al, 2023). Differentially expressed genes were analyzed with the Retrieve/ID mapping tool from the UniProt database to obtain the corresponding reviewed protein sequences. These sequences were then used to obtain information about the presence of signal peptides, transmembrane domains, and unconventional protein secretion using OutCyte v1.022 prediction tools. Lists of proteins have been filtered using the list from the Matrisome project (http://matrisomeproject.mit.edu) (Naba et al, 2016) to annotate them into matrisome protein groups.

**Table 2:** Human biopsies used for the LC-MS/MS analysis.

Status	Muscle	Age	Sex
Healthy (CTL)	Cricopharyngeal (CP)	78	F
		86	M
		70	M
		83	F
	Paraspinal (PS)	19	M
		17	M
		18	M
	Dorsal (D)	17	M
OPMD	Cricopharyngeal (CP)	56	M
		52	F
		64	M
IBM	Cricopharyngeal (CP)	76	F
		70	ND
		60	M
		63	M
DMD	Paraspinal (PS)	15	M
		12	M
		15	M
		15	M

### Cell isolation, culture, and ECM production

Human cells from muscle samples were isolated as described previously (Bigot et al, 2009): briefly, muscle biopsy samples were minced, and explants were plated onto dishes coated with fetal bovine serum (FBS) (Invitrogen). Isolated cells were cultured at 37 °C in growth medium (GM) consisting of 199 medium (Life Technologies) and Dulbecco’s modified Eagle’s medium (DMEM, Life Technologies) at a 1:4 volume ratio supplemented with 20% FBS, 25 μg/mL fetuin (Life Technologies), 0.5 ng/ mL bFGF (Life Technologies), 5 ng/mL EGF (Life Technologies), 5 μg/mL insulin (Sigma-Aldrich), and 50 μg/mL gentamycin (Life Technologies) in a humid atmosphere containing 5% CO_2_ and tested for mycoplasma contamination. Myoblasts and FAPs were isolated by using magnetic activated cell sorting (MACS) with CD56 antibody-coupled microbeads (130-050-401, Miltenyi Biotec) according to the manufacturer’s instructions. After separation of the two populations, CD56+ myoblasts and CD56- FAPs, the purity of each population was monitored by immunocytochemistry after fixation for 10 min with pure ethanol using an antibody against desmin (clone D33, Dako), which is exclusively expressed in myoblasts. At least 500 cells per condition were counted. FAPs used in subsequent analysis were always <1.5% desmin-positive and myoblasts >90% desmin-positive. Lifespan and proliferative status of cells used in this study have been carefully monitored to make sure cells do not reach pre-senescence for any of the conditions. For the production of ECM, FAPs were plated at 15.10^e^3/cm^2^ on non-coated glass coverslips in four-well plates, and the GM was changed every 3 days for 14 days.

### In vivo FAPs transplantation

This study was carried out in strict accordance with the legal regulations in France and according to the ethical guidelines for animal research of the European Union. The protocol was approved and delivered by the French Ministry of Higher Education and Scientific Research (number: 2021072217421004_v4).

Mice were housed in groups of 4–5 per cage in individually ventilated cages with an automatic watering system and ad libitum access to food. Temperature and humidity were controlled, and a 12-h light/dark cycle was maintained. Cages were enriched with nesting material and tunnels. Mice were monitored daily for general health. 3-month-old male and female Rag2−/− Il2rb−/− immunodeficient mice were used as recipients of transplanted human FAPs. Mice were anesthetized by an intraperitoneal injection of 80 mg/kg ketamine hydrochloride and 10 mg/kg xylazine. The *tibialis anterior* (TA) of the mice was subjected to damage in order to trigger regeneration; for OPMD FAPs injections, TAs were subjected to three freeze lesion cycles as previously described (Negroni et al, 2009), then 15 μL of 1.4 × 10^5^ FAPs in PBS were injected immediately after cryodamage and 4 and 8 days later. For DMD FAPs injections, TAs were injected with 20 μl of notexin 20 μg/mL, and 1 × 10^5^ cells in 15 μl of PBS were injected the next day. Mice were euthanized by cervical dislocation 4 weeks after the first injection, and the TAs were collected, snap frozen in isopentane, and stored at −80 °C for further analyses. Human-specific antibodies were used to distinguish human cells within mouse TAs. For fiber diameter and Pax7-positive cells number analysis, areas containing human cells were compared to areas without human cells in the same mouse TA.

### Co-culture and collagen coating experiments

For co-culture experiments, myoblasts and FAPs were seeded together at a 70%/30% ratio and a final density of 21,000 cells/cm^2^ in μ-Slide 8 Well (80826, Ibidi) plates. Cells were differentiated by shifting the growth medium to a serum-free medium consisting of DMEM and 50 μg/mL gentamycin. After 3 or 5 days of differentiation, cells were fixed with PFA 4% and used for subsequent immunostaining experiments.

For collagen treatment, collagen 6 (Rockland, 009-001-108) was diluted in 0.1% acetic acid and coated on μ-Slide 8 Well (80826, Ibidi) plates from 1.25 to 50 μg/cm^2^. The coating was left at 37 °C for 1 h before washing with PBS and seeding human myoblasts from healthy individuals. Cells were differentiated the next day and fixed with PFA 4% after 5 days of differentiation.

### siRNA transfection

Primary FAPs from OPMD patients were seeded at a confluence of 80% in a 48-well plate. The next day, cells were transfected with COL6A3 siRNAs (ON-TARGETplus siRNA human COL6A3 SMARTPool, L-003646-00, Dharmacon) at a final concentration of 30 nM, using Lipofectamine RNAiMAX reagent (10601435, Invitrogen) according to the manufacturer’s instructions, in growth medium. ON-TARGETplus Non-targeting pool (D-001810-10) was used as a negative control. The next day, FAPs were detached, counted, and seeded in co-culture with myoblasts as described above. Three days after differentiation, the supernatant was collected, and cells were fixed with PFA 4% for subsequent analysis.

### Dot blot analysis

Supernatant from co-culture experiments were collected, and centrifuged at 300 G for 10 min to remove dead cells. Dot blot apparatus (Bio-Dot 1706545, Bio-Rad) was used following manufacturer instructions: 40 μl of each supernatant was transferred onto a nitrocellulose membrane by micro-filtration. The membrane was stained with Ponceau red for total protein quantification. After rinsing, the membrane was blocked in Tris-buffered saline with Tween 20 (TBST) containing 5% milk for 30 min before incubation with COL6A3 antibody (MAB 1944) overnight. Membrane was incubated with appropriate secondary antibody in TBST for 45 min before revelation using the Clarity Max ECL substrate (1705062, Bio-Rad) on a ChemiDoc MP imaging system (Bio-Rad). Protein quantification was performed by normalizing Collagen VI signal to total protein using an ImageJ macro (Ohgane and Yoshioka, 2019).

### Quantitative PCR

RNA from frozen mouse muscle sections was extracted using Trizol reagent (Invitrogen) according to the manufacturer’s instructions. RNA was reverse transcribed using M-MLV (Invitrogen) according to the manufacturer’s instructions. Quantitative polymerase chain reaction (qPCR) was carried out using SYBR Green Mastermix (Roche Applied Science) in a LightCycler 480 Real-Time PCR System (Roche, Applied Science) or a Applied Biosystems QuantStudio 6 Pro (Thermofisher scientific) with the following cycling protocol: 8 min at 95 °C; followed by 50 cycles at 95 °C for 15 s (s), 60 °C for 15 s and 72 °C for 15 s, and a final step consisting of 5 s at 95 °C and 1 min at 65 °C. The specificity of the PCR product was evaluated by melting curve analysis using the following program: 65 °C to 97 °C with a 0.11 °C/s increase. To analyze the gene expression of injected human cells, only human-specific primers (see Reagents and Tools table) were used, and gene expression levels were normalized to human RPLP0 or human B2M expression and quantified with the 2–ΔΔCt method. The human specificity of the primers was attested using a non-injected TA muscle as a negative control for each qPCR. The primer sequences used in this study are listed in the Reagents and Tools table.

### Immunofluorescence imaging

Immunostaining was performed either on 5-μm-thick muscle biopsy sections or on FAPs in vitro, fixed in 4% paraformaldehyde for 10 min at room temperature (RT) and incubated with PBS containing 2% FBS and 0,2% Triton for 30 min at RT. Incubation with primary antibodies (see Reagents and Tools table) was performed at RT for 1 h (h) then cryosections or fixed cells were incubated with appropriate fluorescent secondary antibodies (Life Technologies) at 1/400 at RT for 45 min. Nuclei were stained with Hoechst. Widefield fluorescence images were taken with an Axio Observer 7 microscope (Zeiss) equipped with a motorized stage coupled to an Orca Flash 4 Camera (Hamamatsu) and driven by the Zen software (Zeiss).

### Statistics

Data are expressed as the mean ± SD. Statistical significance was assessed by ordinary one-way ANOVA followed by Tukey’s multiple comparisons test or Student's *T* tests. All statistical analyses were performed using GraphPad Prism (version 8.0, GraphPad Software Inc.). Differences were considered to be significant at *P < 0.05, **P < 0.01, ***P < 0.001, and ****P < 0.0001. The number of animals per group and the number of primary cultures were determined based on prior studies in our laboratory and established practices in the field. Animals and primary cultures were randomly assigned to experimental groups, and data acquisition and analysis were performed blinded to these group assignments. No animals or primary cultures were excluded, and all samples collected were included in the analysis.

## Supplementary information

Appendix

Peer Review File

Source data Fig. 1

Source data Fig. 2

Source data Fig. 3

Source data Fig. 4

Source data Fig. 5

## Data Availability

The mass spectrometry proteomics data have been deposited to the OSF website: https://osf.io/f6wc7/overview?view_only=d817b9d425c8460ebc3d50d3ebb2d5d0. The source data of this paper are collected in the following database record: biostudies:S-SCDT-10_1038-S44319-026-00834-0.
