# Publisher Correction: Collagen VI is a fibrosis-associated signal disrupting muscle regeneration across distinct human myopathies

**DOI:** 10.1038/s44319-026-00857-7

**Published:** 2026-07-01

**Authors:** Laura Muraine, Mona Bensalah, Stephen Gargan, Paul Dowling, Anne Bigot, Valérie Allamand, Jamila Dhiab, Maria Kondili, Sophie Perié, Jean Lacau St-Guily, Gillian Butler-Browne, Vincent Mouly, Kay Ohlendieck, Capucine Trollet, Elisa Negroni

**Affiliations:** 1 Center for Research in Myology U974, Sorbonne Université, INSERM, Myology Institute, Paris, France; 2 Department of Biology, Maynooth University, National University of Ireland, Maynooth W23F2H6, Co, Kildare, Ireland; 3 Kathleen Lonsdale Institute for Human Health Research, Maynooth University, Maynooth W23F2H6, Co, Kildare, Ireland; 4 Department of Otolaryngology Head and Neck Surgery, Com Maillot-Hartmann Clinic, Neuilly Sur Seine, France; 5 Department of Otolaryngology-Head and Neck surgery, Rothschild Foundation Hospital and Sorbonne University, Paris, France

**Correction to:** *EMBO Reports*. 10.1038/s44319-026-00834-0 | Published online 19 June 2026

In this article, the author’s name Gillian Butler-Browne was incorrectly written as Gillian Butler-oBrowne. The original article has been corrected.

