# Supplementary material for: Collagen VI is a fibrosis-associated signal disrupting muscle regeneration across distinct human myopathies
Source: EMBO Rep. 2026 Jun 19;27(14):4124–40. doi: 10.1038/s44319-026-00834-0 (PMC13400756; doi:10.1038/s44319-026-00834-0)
Supplement: Supplementary file 1 — Appendix [file 44319_2026_834_MOESM1_ESM.pdf]

Appendix

Collagen VI is a fibrosis-associated signal disrupting muscle regeneration across distinct human myopathies

Laura Muraine[1], Mona Bensalah[1], Stephen Gargan[2,3], Paul Dowling[2,3], Anne Bigot[1], Valérie Allamand[1], Jamila Dhiab[1], Maria Kondili[1], Sophie Perié[1,4], Jean Lacau St-Guily[1,5], Gillian Butler-Browne[1], Vincent Mouly[1], Kay Ohlendieck[2,3], Capucine Trollet[1 †*], Elisa Negroni[1 †*]

Table of content

Appendix Figure S1 2
Appendix Figure S2 2
Appendix Table S1 3
Appendix Table S2 8
Appendix Table S3 12
Appendix Table S4 14
Appendix Table S5 17
Appendix Table S6 19
Appendix Table S7 20
Appendix Table S8 21
Appendix Table S9 22

Appendix Figure S1

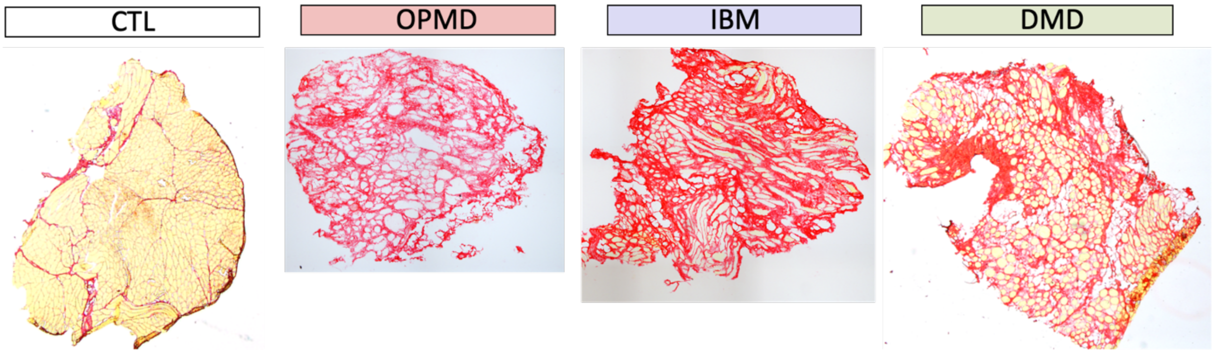

Sirius red coloration of whole human skeletal muscle biopsies from healthy control (CTL), OPMD, IBM and DMD patients.

Appendix Figure S2

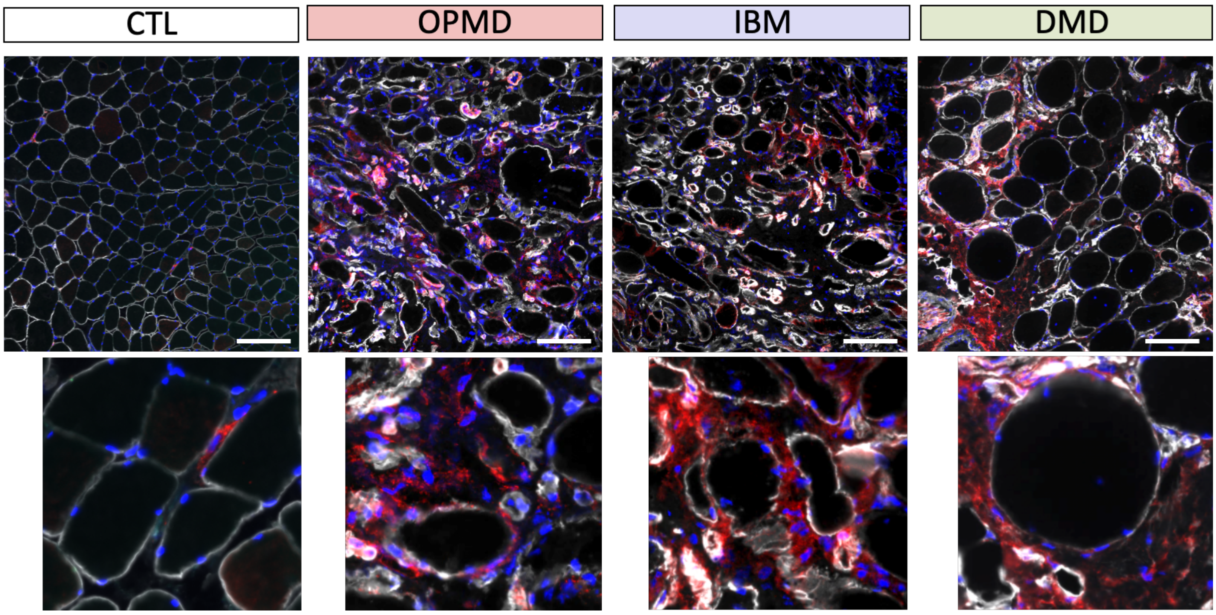

Panel from Figure 1A and their magnification images. Immunostaining of laminin (white), Hoechst (blue) and CD90 (red) on human biopsies. Scale bar: 50μm

Appendix Table S1

List of identified proteins that exhibit an **increased** abundance in tissue specimens from patients afflicted with **Duchenne muscular dystrophy**. Protein samples were analysed by label-free LC-MS/MS.

Protein	Gene	Peptides	ANOVA p-value	Molecular mass (kDa)	Fold change	Outcyte prediction
Phosphoglycerate kinase 1	PGK1	20	0.000577	44.6	23.81	Intracellular
Aspartate aminotransferase, cytoplasmic	GOT1	18	0.000117	46.2	15.33	Intracellular
Histone H2A type 3	Hist3h2a	3	0.00042	14.1	12.01	Intracellular
Myc box-dependent-interacting protein 1	BIN1	17	0.045572	64.6	8.74	UPS
Calsequestrin-2	CASQ2	7	0.001895	46.4	8.22	signal peptide
SERCA1 Calcium ATPase	ATP2A1	45	2.33E-06	110.2	7.33	Transmembrane
LIM domain-binding protein 3	LDB3	21	0.025152	77.1	7.24	Intracellular
L-lactate dehydrogenase A chain	LDHA	18	7.82E-08	36.6	7.09	UPS
Retinal dehydrogenase 1	ALDH1A1	13	0.00344	54.8	7.05	UPS
Ceruloplasmin	CP	17	5.57E-09	122.2	6.99	signal peptide
Aspartate aminotransferase, mitochondrial	GOT2	18	0.002905	47.5	6.59	Intracellular

Adenylate kinase isoenzyme 1	AK1	12	5.76E-05	21.6	6.42	UPS
Haptoglobin	HP	21	1.11E-07	45.2	6.18	signal peptide
Carbonic anhydrase 1	CA1	13	0.002464	28.8	6.18	UPS
Antithrombin-III	SERPINC1	10	7.00E-06	52.6	6.15	signal peptide
Alpha-1-antitrypsin	SERPINA1	20	1.12E-08	46.7	6.09	signal peptide
Alpha-2-macroglobulin	A2M	46	4.19E-05	163.2	6.09	signal peptide
Complement C3	C3	56	8.12E-07	187.1	5.77	signal peptide
Ig kappa chain C region	IGKC	7	2.17E-05	11.7	5.76	Intracellular
Ig gamma-1 chain C region	IGHG1	14	1.20E-06	36.1	5.66	UPS
Ig mu chain C region	IGHM	14	0.003589	49.4	5.31	Intracellular
Ig alpha-1 chain C region	IGHA1	8	0.001251	37.6	5.31	UPS
Hemoglobin subunit delta	HBD	15	0.005782	16.1	5.23	UPS
Myoglobin	MB	13	0.00545	17.2	5.21	UPS
Collagen alpha-1(I) chain	COL1A1	10	0.000217	138.9	5.15	signal peptide
Lamin-A/C	LMNA	33	4.35E-05	74.1	4.98	Intracellular
Troponin C, skeletal muscle	TNNC2	10	9.44E-06	18.1	4.78	UPS
Apolipoprotein A-I	APOA1	19	9.56E-09	30.7	4.77	signal peptide
Apolipoprotein A-II	APOA2	4	5.80E-07	11.2	4.77	signal peptide
Fibrinogen alpha chain	FGA	18	0.002479	94.9	4.48	signal peptide
Fibrinogen beta chain	FGB	28	0.000298	55.9	4.45	signal peptide

Fibrinogen gamma chain	FGG	19	0.000157	51.5	4.31	signal peptide
Band 3 anion transport protein	SLC4A1	22	0.016178	101.7	4.12	Intracellular
Alpha-1-acid glycoprotein 1	ORM1	6	0.004218	23.5	4.08	signal peptide
Serotransferrin	TF	34	4.98E-10	77.1	3.93	signal peptide
Hemopexin	HPX	14	4.64E-06	51.6	3.78	signal peptide
Vitronectin	VTN	9	0.000339	54.3	3.54	signal peptide
Catalase	CAT	19	0.016932	59.7	3.51	Intracellular
Fructose-bisphosphate aldolase A	ALDOA	26	7.14E-05	39.4	3.47	Intracellular
Glyceraldehyde-3-phosphate dehydrogenase	GAPDH	17	7.01E-06	36.1	3.33	UPS
Myosin light chain 1/3, skeletal muscle isoform	MYL1	20	0.000146	21.1	3.32	UPS
Creatine kinase M-type	CKM	29	0.000136	43.1	3.18	Intracellular
Glucose-6-phosphate isomerase	GPI	13	4.31E-06	63.1	3.08	Intracellular
L-lactate dehydrogenase B chain	LDHB	12	0.000287	36.6	3.04	UPS
Protein disulfide-isomerase	P4HB	12	1.40E-06	57.1	3.02	signal peptide
Annexin A2	ANXA2	23	2.49E-07	38.6	2.93	UPS
Tubulin beta chain	TUBB	15	0.012759	49.6	2.67	Intracellular
Decorin	DCN	9	2.99E-09	39.7	2.57	signal peptide

Profilin-1	PFN1	7	0.045603	15.1	2.43	UPS
Tropomyosin beta chain	TPM2	31	0.004073	32.8	2.42	UPS
Collagen alpha-2(I) chain	COL1A2	9	1.51E-05	129.3	2.41	signal peptide
Annexin A6	ANXA6	28	0.000152	75.8	2.40	Intracellular
Heat shock protein HSP 90-beta	HSP90AB1	22	0.008443	83.2	2.39	Intracellular
Pyruvate dehydrogenase E1 component subunit alpha, mitochondrial	PDHA1	10	0.038441	43.3	2.36	Intracellular
Vimentin	VIM	29	5.39E-05	53.6	2.34	Intracellular
Annexin A5	ANXA5	11	4.99E-05	35.9	2.26	UPS
Galectin-1	LGALS1	6	0.002512	14.7	2.24	signal peptide
Tropomyosin alpha-1 chain	TPM1	35	5.26E-06	32.7	2.23	UPS
Fructose-bisphosphate aldolase C	ALDOC	4	0.000776	39.4	2.21	Intracellular
Immunoglobulin kappa light chain	IGKC	7	3.94E-05	23.3	2.15	Intracellular
Calmodulin	calm1a	8	0.016161	16.8	1.97	UPS
Myosin regulatory light chain 2, cardiac	MYL2	15	0.000423	18.7	1.89	UPS
Laminin subunit gamma-1	LAMC1	25	0.030443	177.6	1.86	signal peptide

Glycogen phosphorylase, muscle form	PYGM	55	3.74E-08	97.1	1.85	Intracellular
Spectrin beta chain, erythrocytic	SPTB	60	0.046824	246.4	1.84	Intracellular
Collagen alpha-1(VI) chain	COL6A1	27	1.18E-10	108.5	1.81	signal peptide
Collagen alpha-2(VI) chain	COL6A2	22	2.55E-09	108.5	1.69	signal peptide
Collagen alpha-3(VI) chain	COL6A3	83	4.67E-09	343.6	1.63	signal peptide
Creatine kinase B-type	CKB	9	0.01345	42.6	1.62	Intracellular
Myosin-1	MYH1	180	2.83E-08	223.1	1.51	Intracellular
Myosin-7	MYH7	186	0.000775	223.1	1.50	Intracellular
Beta-enolase	ENO3	24	2.84E-05	46.9	1.48	UPS
Pyruvate kinase PKM	PKM	34	3.15E-05	57.9	1.45	Intracellular
Myosin light chain 6B	MYL6B	17	0.028465	22.7	1.43	UPS
Aldose reductase	AKR1B1	12	2.87E-05	35.8	1.39	UPS
Phosphoglycerate mutase 2	PGAM2	14	0.006858	28.7	1.37	UPS
SERCA2 Calcium ATPase	ATP2A2	46	0.021393	114.7	1.36	Transmembrane

APPENDIX TABLE S2

List of identified proteins that exhibit a **decreased** abundance in tissue specimens from patients afflicted with **Duchenne muscular dystrophy**. Protein samples were analysed by label-free LC-MS/MS.

Protein	Gene	Peptides	ANOVA p-value	Molecular mass (kDa)	Fold Change	Outcyte prediction
Desmin	DES	29	4.53E-05	53.5	1.18	Intracellular
Mimecan	OGN	11	6.64E-06	33.9	1.23	signal peptide
Nebulin	NEB	334	0.000753	772.9	1.26	Intracellular
Cytochrome b-c1 complex subunit 2, mitochondrial	UQCRC2	13	0.018903	48.4	1.27	Intracellular
AMP deaminase 1	AMPD1	17	0.000296	90.2	1.29	Intracellular
Calnexin	CANX	9	0.00929	67.5	1.30	signal peptide
Glutathione S-transferase Mu 2	GSTM2	12	0.014107	25.7	1.33	UPS
Flavin reductase (NADPH)	BLVRB	8	0.000992	22.1	1.33	UPS
Phosphatidylethanolamine-binding protein 1	PEBP1	12	5.81E-07	21.1	1.38	UPS
Calsequestrin-1	CASQ1	10	3.56E-06	45.2	1.40	signal peptide
Peroxiredoxin-2	PRDX2	11	0.000485	21.8	1.45	UPS
Fibrillin-1	FBN1	50	0.00057	312.3	1.45	signal peptide
Glycogen debranching enzyme	AGL	54	0.000188	174.7	1.47	Intracellular

Myosin-9	MYH9	37	0.000247	226.5	1.50	Intracellular
Phosphoglucomutase-1	PGM1	24	0.000182	61.4	1.51	Intracellular
Malate dehydrogenase, cytoplasmic	MDH1	11	0.03641	36.4	1.57	UPS
Troponin T, fast skeletal muscle	TNNT3	17	8.59E-06	31.8	1.82	Intracellular
Glycogenin-1	GYG1	8	0.034523	39.3	1.84	UPS
F-actin-capping protein subunit alpha-2	CAPZA2	14	0.016389	32.9	1.87	UPS
Lumican	LUM	10	4.22E-10	38.4	1.87	signal peptide
Prolargin	PRELP	14	1.97E-06	43.8	1.90	signal peptide
Myomesin-1	MYOM1	89	0.015736	187.6	1.95	Intracellular
Myomesin-2	MYOM2	77	0.00033	164.8	1.96	Intracellular
Triosephosphate isomerase	TPI1	17	7.79E-05	26.6	2.01	UPS
Actin, cytoplasmic 1	ACTB	29	1.45E-06	41.7	2.04	Intracellular
Histone H4	HIST1H4A	5	0.000161	11.3	2.05	Intracellular
Peptidyl-prolyl cis-trans isomerase A	PPIA	8	0.001389	18.0	2.19	UPS
14-3-3 protein zeta/delta	YWHAZ	10	0.001528	27.7	2.21	UPS
Troponin C, slow skeletal and cardiac muscles	TNNC1	14	0.008861	18.4	2.27	UPS
Tubulin alpha-1B chain	TUBA1B	12	0.001781	50.1	2.40	Intracellular

Hemoglobin subunit beta	HBB	17	0.000278	15.9	2.70	UPS
Hemoglobin subunit alpha	HBA1	14	0.00128	15.2	2.71	UPS
Glutathione S-transferase omega-1	GSTO1	7	0.047125	27.5	2.74	UPS
Basement membrane-specific heparan sulfate proteoglycan core protein	HSPG2	54	0.000918	468.8	2.96	signal peptide
Myosin-binding protein C, slow-type	MYBPC1	70	0.037722	128.2	3.06	Intracellular
Selenium-binding protein 1	SELENBP1	16	0.030134	52.4	3.08	UPS
UTP-glucose-1-phosphate uridylyltransferase	UGP2	18	0.04067	56.9	3.35	UPS
PDZ and LIM domain protein 3	PDLIM3	17	0.015789	39.2	3.56	Intracellular
Polymerase I and transcript release factor	PTRF	8	0.00299	43.4	3.67	Intracellular
Tripartite motif-containing protein 72	TRIM72	19	0.016553	52.7	3.81	Intracellular
Titin	TTN	1601	0.00128	3816	3.84	Intracellular
Myosin regulatory light chain 2, skeletal muscle isoform	MYLPF	18	5.98E-06	19.0	4.29	UPS
PDZ and LIM domain protein 5	PDLIM5	9	0.007655	63.9	4.71	UPS
Protein deglycase DJ-1	PARK7	7	2.50E-07	19.8	4.74	UPS

Asporin	ASPN	9	6.83E-06	43.4	5.11	signal peptide
Myozenin-1	MYOZ1	12	0.009786	31.7	5.12	UPS
Reticulon-4	RTN4	5	0.041574	129.9	5.16	Intracellular
EH domain-containing protein 2	EHD2	11	0.013169	61.1	5.18	Intracellular
LIM and cysteine-rich domains protein 1	LMCD1	21	0.028637	40.8	5.21	Intracellular
Vesicle-associated membrane protein-associated protein A	VAPA	3	0.034867	27.8	5.41	UPS
NADH-cytochrome b5 reductase 1	CYB5R1	13	0.005189	34.1	8.11	Transmembrane
Myosin-2	MYH2	177	6.60E-05	223.0	10.55	Intracellular
Probable C->U-editing enzyme APOBEC-2	APOBEC2	9	0.007095	25.7	21.69	UPS
Voltage-dependent anion-selective channel protein 3	VDAC3	6	0.00721	30.6	39.17	UPS
Protein-arginine deiminase type-2	PADI2	16	0.007478	75.5	41.52	Intracellular
Myosin-4	MYH4	104	0.000617	223.1	87.80	Intracellular

Appendix Table S3

List of identified proteins that exhibit an **increased** abundance in cricopharyngeal muscle specimens from patients afflicted with **oculopharyngeal muscular dystrophy**. Protein samples were analysed by label-free LCMS/MS.

**Protein**	**Gene**	**Peptides**	**ANOVA p-value**	**Molecular mass (kDa)**	**Fold change**	**Outcyte prediction**
Filamin-A	FLNA	77	0.004287	280.7	7.53	Intracellular
Fibronectin	FN1	51	0.003843	272.3	7.40	signal peptide
Complement factor H	CFH	28	0.004987	139.1	6.62	signal peptide
Ig mu chain C region	IGHM	14	0.019406	49.4	6.32	Intracellular
Antithrombin-III	SERPINC1	20	0.018219	52.6	5.59	signal peptide
Basement membrane-specific heparan sulfate proteoglycan core protein	HSPG2	49	0.000406	468.8	5.21	signal peptide
Histone H4	HIST1H4A	8	0.003623	11.4	4.01	Intracellular
Tenascin-X	TNXB	61	0.010999	458.3	3.82	signal peptide
Spectrin alpha chain, non-erythrocytic 1	SPTAN1	54	0.000714	284.5	3.58	Intracellular
Myosin-11	MYH11	69	0.007319	227.3	3.33	Intracellular
Clusterin	CLU	13	0.042211	52.4	3.31	signal peptide
Plasminogen	PLG	30	0.036482	90.5	2.94	signal peptide

Hemopexin	HPX	14	0.042217	51.6	2.83	signal peptide
Collagen alpha-2(VI) chain	COL6A2	24	0.005593	108.5	2.67	signal peptide
Laminin subunit alpha-2	LAMA2	20	0.011041	343.9	2.54	signal peptide
Laminin subunit gamma-1	LAMC1	27	0.00967	177.6	2.50	signal peptide
Heat shock 70 kDa protein 1A	HSPA1	21	0.005859	70.1	2.43	Intracellular
Collagen alpha-1(IV) chain	COL4A1	3	0.04153	160.6	2.25	signal peptide
Mast cell carboxypeptidase A	CPA3	10	0.033325	48.6	2.23	signal peptide
Collagen alpha-1(VI) chain	COL6A1	25	0.010699	108.5	2.08	signal peptide
Laminin subunit beta-2	LAMB2	32	0.041314	195.9	2.05	signal peptide
Complement factor B	CFB	17	0.010158	85.5	2.04	signal peptide
14-3-3 protein zeta/delta	YWHAZ	15	0.007801	27.7	2.01	UPS
Lamin-A/C	LMNA	39	0.032851	74.1	1.91	Intracellular
Glutathione S-transferase P	GSTP1	8	0.016469	23.4	1.89	UPS
14-3-3 protein epsilon	YWHAE	17	0.042265	29.2	1.89	UPS
Collagen alpha-3(VI) chain	COL6A3	85	0.020197	343.6	1.87	signal peptide
Actin, cytoplasmic 2	ACTG1	22	0.018436	41.7	1.72	Intracellular
Tubulin beta-4B chain	TUBB4B	15	0.000324	49.8	1.63	Intracellular
Annexin A2	ANXA2	25	0.027693	38.6	1.62	UPS
Aldehyde dehydrogenase, mitochondrial	ALDH2	14	0.025178	56.3	1.61	Intracellular

Alpha-enolase	ENO1	15	0.044302	47.2	1.51	UPS

Appendix Table S4

List of identified proteins that exhibit an **increased** abundance in cricopharyngeal muscle specimens from patients afflicted with **inclusion body myositis**. Protein samples were analysed by label-free LC-MS/MS.

**Protein**	**Gene**	**Peptides**	**ANOVA p-value**	**Molecular mass (kDa)**	**Fold change**	**Outcyte prediction**
Actin, alpha cardiac muscle 1	ACTC1	31	0.000355	42.0	6.89	Intracellular
Basement membrane-specific heparan sulfate proteoglycan core protein	HSPG2	68	0.000751	468.8	6.11	signal peptide
Fibronectin	FN1	45	0.017906	272.3	5.98	signal peptide
Aconitate hydratase, mitochondrial	ACO2	36	0.018057	85.4	5.26	Intracellular
Citrate synthase, mitochondrial	CS	12	0.02578	51.7	4.60	UPS
Cytochrome b-c1 complex subunit 1, mitochondrial	UQCRC1	19	0.026717	52.6	4.34	Intracellular
Elongation factor Tu, mitochondrial	TUFM	19	0.017793	49.5	4.31	Intracellular

Histone H4	HIST1H4A	9	6.71E-05	11.3	3.75	Intracellular
Plasminogen	PLG	25	0.006887	90.5	3.67	signal peptide
Mast cell carboxypeptidase A	CPA3	9	0.009916	48.6	3.64	signal peptide
Tenascin-X	TNXB	61	0.02395	458.3	3.46	signal peptide
Annexin A5	ANXA5	14	0.009982	35.9	3.43	UPS
Calsequestrin-2	CASQ2	11	0.013957	46.4	3.31	signal peptide
Titin	TTN	645	0.022355	3906.4	3.18	Intracellular
NAD(P) transhydrogenase, mitochondrial	NNT	24	0.041736	113.8	3.17	Intracellular
Superoxide dismutase [Mn], mitochondrial	SOD2	7	0.026648	24.7	3.15	UPS
Calreticulin	CALR	6	0.011898	48.1	3.03	signal peptide
Annexin A1	ANXA1	21	0.040818	38.7	2.88	UPS
Collagen alpha-2(VI) chain	COL6A2	23	7.24E-05	108.5	2.849	signal peptide
3-ketoacyl-CoA thiolase, mitochondrial	ACAA2	15	0.038653	41.9	2.83	Intracellular
Plectin	PLEC	180	0.013036	531.7	2.63	Intracellular
Annexin A6	ANXA6	30	0.034283	75.8	2.61	Intracellular
Laminin subunit alpha-2	LAMA2	48	0.001802	343.9	2.61	signal peptide

Stress-70 protein, mitochondrial	HSPA9	19	0.032871	73.6	2.56	Intracellular
Tubulin alpha-1B chain	TUBA1B	16	0.026025	50.1	2.54	Intracellular
Obscurin	OBSCN	99	0.036813	868.4	2.45	Intracellular
Polymerase I and transcript release factor	PTRF	9	0.025267	43.4	2.44	Intracellular
Heat shock protein HSP 90-beta	HSP90AB1	25	0.040796	83.2	2.31	Intracellular
Elongation factor 2	EEF2	21	0.001045	95.3	2.28	Intracellular
Heat shock 70 kDa protein 1A	HSPA1A	23	0.039811	70.1	2.28	Intracellular
Desmin	DES	33	0.015993	53.5	2.21	Intracellular
Tubulin beta-4B chain	TUBB4B	20	0.000329	49.8	2.17	Intracellular
Laminin subunit gamma-1	LAMC1	39	0.013587	177.6	2.10	signal peptide
Glutathione S-transferase P	GSTP1	8	0.010106	23.3	2.09	UPS
Tubulin alpha-4A chain	TUBA4A	16	0.028034	49.9	2.05	UPS
Myozenin-2	MYOZ2	13	0.012721	29.8	2.05	Intracellular
Galectin-1	LGALS1	6	0.004436	14.7	2.01	signal peptide
Collagen alpha-1(VI) chain	COL6A1	29	0.024197	108.5	2.01	signal peptide
Elongation factor 1-alpha 1	EEF1A1	14	0.036258	50.1	1.99	UPS
Myotilin	MYOT	24	0.047863	55.3	1.96	Intracellular

Collagen alpha-3(VI) chain	COL6A3	84	0.006678	343.6	1.96	signal peptide
Fibrillin-1	FBN1	80	0.001306	312.3	1.95	signal peptide
Vinculin	VCL	36	0.012835	123.8	1.83	Intracellular
Spectrin alpha chain, non-erythrocytic 1	SPTAN1	66	0.011121	284.5	1.70	Intracellular
Myosin-9	MYH9	70	0.02321	226.5	1.67	Intracellular

APPENDIX TABLE S5

Lists of differentially expressed proteins identified among pathologies when compared to age-matched control muscles. Proteins decreased in DMD biopsies are marked with asterisks.

OPMD	IBM	DMD		OPMD-DMD	IBM-DMD	OPMD-IBM	OPMD-IBM-DMD
FLNA	ACTC1	PGK1	MYH7	IGHM	ANXA5	FN1	*HSPG2
CFH	ACO2	GOT1	ENO3	SERPINC1	CASQ2	TNXB	*HIST1H4A
MYH11	CS	Hist3h2a	PKM	HPX	*TTN	SPTAN1	COL6A2
CLU	UQCRC1	BIN1	MYL6B	*YWHAZ	ANXA6	PLG	LAMC1
HSPA1	TUFM	ATP2A1	AKR1B1	LMNA	*TUBA1B	LAMA2	COL6A1
COL4A1	NNT	LDB3	PGAM2	ANXA2	*PTRF	CPA3	COL6A3
LAMB2	SOD2	LDHA	ATP2A2		HSP90AB1	GSTP1	
CFB	CALR	ALDH1A1	*OGN		*DES	TUBB4B	
YWHAE	ANXA1	CP	*NEB		LGALS1		
ACTG1	ACAA2	GOT2	*UQCRC2		*FBN1		
ALDH2	PLEC	AK1	*AMPD1		*MYH9		
ENO1	HSPA9	HP	*CANX				
	OBSCN	CA1	*GSTM2				
	EEF2	SERPINA1	*BLVRB				
	HSPA1A	A2M	*PEBP1				
	TUBA4A	C3	*CASQ1				
	MYOZ2	IGKC	*PRDX2				
	EEF1A1	IGHG1	*AGL				
	MYOT	IGHA1	*PGM1				
	VCL	HBD	*MDH1				

		MB	*TNNT3				
		COL1A1	*GYG1				
		TNNC2	*CAPZA2				
		APOA1	*LUM				
		APOA2	*PRELP				
		FGA	*MYOM1				
		FGB	*MYOM2				
		FGG	*TPI1				
		SLC4A1	*ACTB				
		ORM1	*PPIA				
		TF	*TNNC1				
		VTN	*HBB				
		CAT	*HBA1				
		ALDOA	*GSTO1				
		GAPDH	*MYBPC1				
		MYL1	*SELENBP1				
		CKM	*UGP2				
		GPI	*PDLIM3				
		LDHB	*TRIM72				
		P4HB	*MYLPF				
		TUBB	*PDLIM5				
		DCN	*PARK7				
		PFN1	*ASPN				
		TPM2	*MYOZ1				
		COL1A2	*RTN4				
		PDHA1	*EHD2				
		VIM	*LMCD1				
		TPM1	*VAPA				
		ALDOC	*CYB5R1				
		calm1a	*MYH2				
		MYL2	*APOBEC2				
		PYGM	*VDAC3				
		SPTB	*PADI2				
		CKB	*MYH4				
		MYH1					

APPENDIX TABLE S6

List of matrisome proteins **increased** in cricopharyngeal muscle from patients with **OPMD** as compared to **CTL**

**Protein**	**Gene**	**Fold change**	**Matrisome database**
Collagen alpha-1(VI) chain	COL6A1	2.08	collagens
Collagen alpha-2(VI) chain	COL6A2	2.67	collagens
Collagen alpha-3(VI) chain	COL6A3	1.87	collagens
Collagen alpha-1(IV) chain	COL4A1	2.25	collagens
Laminin subunit alpha-2	LAMA2	2.54	glycoproteins
Laminin subunit beta-2	LAMB2	2.05	glycoproteins
Laminin subunit gamma-1	LAMC1	2.50	glycoproteins
Fibronectin	FN1	7.40	glycoproteins
Tenascin-X	TNXB	3.82	glycoproteins
Basement membrane-specific heparan sulfate proteoglycan core protein	HSPG2	5.21	proteoglycans
Annexin A2	ANXA2	1.62	ecm affiliated
Hemopexin	HPX	2.83	ecm affiliated
Antithrombin-III	SERPINC1	5.59	regulators
Plasminogen	PLG	2.94	regulators

APPENDIX TABLE S7

List of matrisome proteins **increased** in cricopharyngeal muscle from patients with **IBM** as compared to **CTL**.

**Protein**	**Gene**	**Fold change**	**Matrisome database**
Collagen alpha-1(VI) chain	COL6A1	2.01	collagens
Collagen alpha-2(VI) chain	COL6A2	2.849	collagens
Collagen alpha-3(VI) chain	COL6A3	1.96	collagens
Laminin subunit alpha-2	LAMA2	2.61	glycoproteins
Laminin subunit gamma-1	LAMC1	2.10	glycoproteins
Tenascin-X	TNXB	3.46	glycoproteins
Fibronectin	FN1	5.98	glycoproteins
Fibrillin-1	FBN1	1.95	glycoproteins
Basement membrane-specific heparan sulfate proteoglycan core protein	HSPG2	6.11	proteoglycans
Galectin-1	LGALS1	2.01	ecm affiliated
Annexin A1	ANXA1	2.88	ecm affiliated
Annexin A5	ANXA5	3.43	ecm affiliated
Annexin A6	ANXA6	2.61	ecm affiliated
Plasminogen	PLG	3.67	regulators

APPENDIX TABLE S8

List of matrisome proteins **increased** in paraspinal muscle from patients with **DMD** as compared to **CTL.**

**Protein**	**Gene**	**Fold change**	**Matrisome database**
Collagen alpha-1(I) chain	COL1A1	5.15	collagens
Collagen alpha-2(I) chain	COL1A2	2.41	collagens
Collagen alpha-1(VI) chain	COL6A1	1.81	collagens
Collagen alpha-2(VI) chain	COL6A2	1.69	collagens
Collagen alpha-3(VI) chain	COL6A3	1.63	collagens
Laminin subunit gamma-1	LAMC1	1.86	glycoproteins
Vitronectin	VTN	3.54	glycoproteins
Fibrinogen alpha chain	FGA	4.48	glycoproteins
Fibrinogen beta chain	FGB	4.45	glycoproteins
Fibrinogen gamma chain	FGG	4.31	glycoproteins
Decorin	DCN	2.57	proteoglycans
Annexin A6	ANXA6	2.40	ecm affiliated
Annexin A5	ANXA5	2.26	ecm affiliated
Annexin A2	ANXA2	2.93	ecm affiliated
Galectin-1	LGALS1	2.24	ecm affiliated

Hemopexin	HPX	3.78	ecm affiliated
Alpha-2-macroglobulin	A2M	6.09	regulators
Alpha-1-antitrypsin	SERPINA1	6.09	regulators
Antithrombin-III	SERPINC1	6.15	regulators

APPENDIX TABLE S9

List of matrisome proteins **decreased** in paraspinal muscle from patients with **DMD** as compared to **CTL.**

**Protein**	**Gene**	**Fold change**	**Matrisome database**
Fibrillin-1	FBN1	1.45	glycoproteins
Asporin	ASPN	5.11	proteoglycans
Lumican	LUM	1.87	proteoglycans
Mimecan	OGN	1.23	proteoglycans
Prolargin	PRELP	1.90	proteoglycans
Basement membrane-specific heparan sulfate proteoglycan core protein	HSPG2	2.96	proteoglycans
