# Supplementary figures and images for: Collagen VI is a fibrosis-associated signal disrupting muscle regeneration across distinct human myopathies
Source: EMBO Rep. 2026 Jun 19;27(14):4124–40. doi: 10.1038/s44319-026-00834-0 (PMC13400756; doi:10.1038/s44319-026-00834-0)

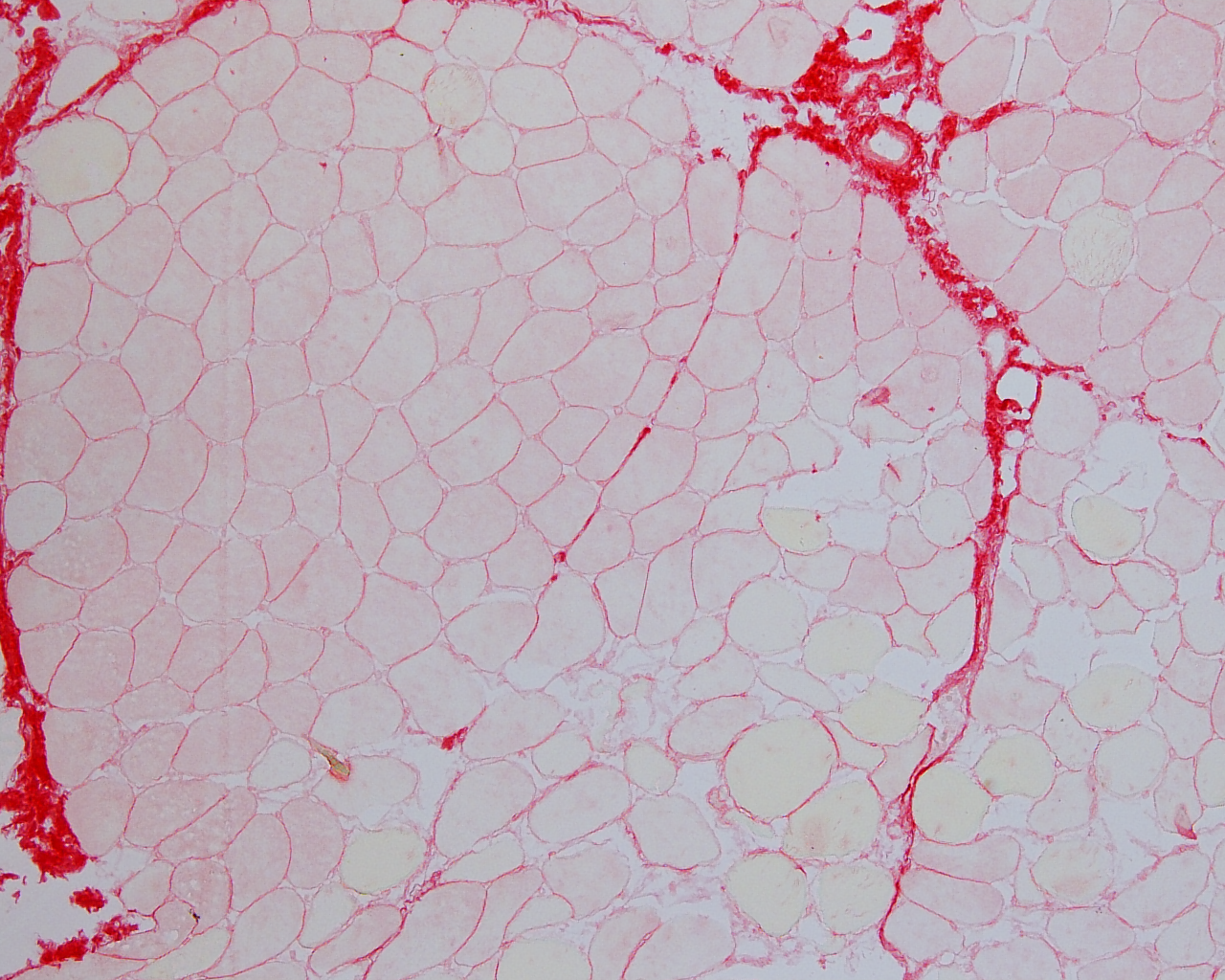

Supplement: Supplementary file 3 — Source data Fig. 1 [file 44319_2026_834_MOESM3_ESM.zip › Figure 1/1A/CTL.tif]

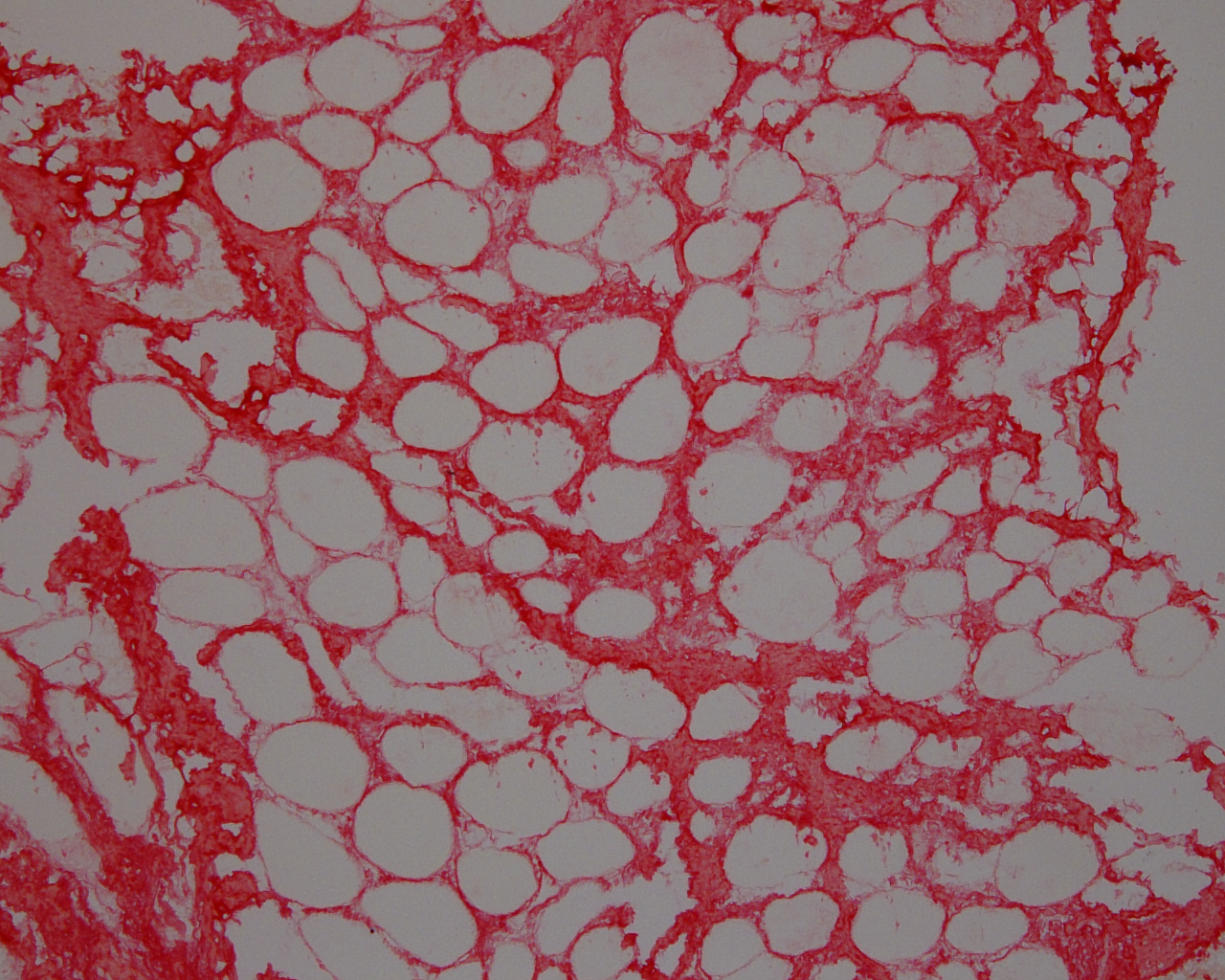

Supplement: Supplementary file 3 — Source data Fig. 1 [file 44319_2026_834_MOESM3_ESM.zip › Figure 1/1A/DMD.tif]

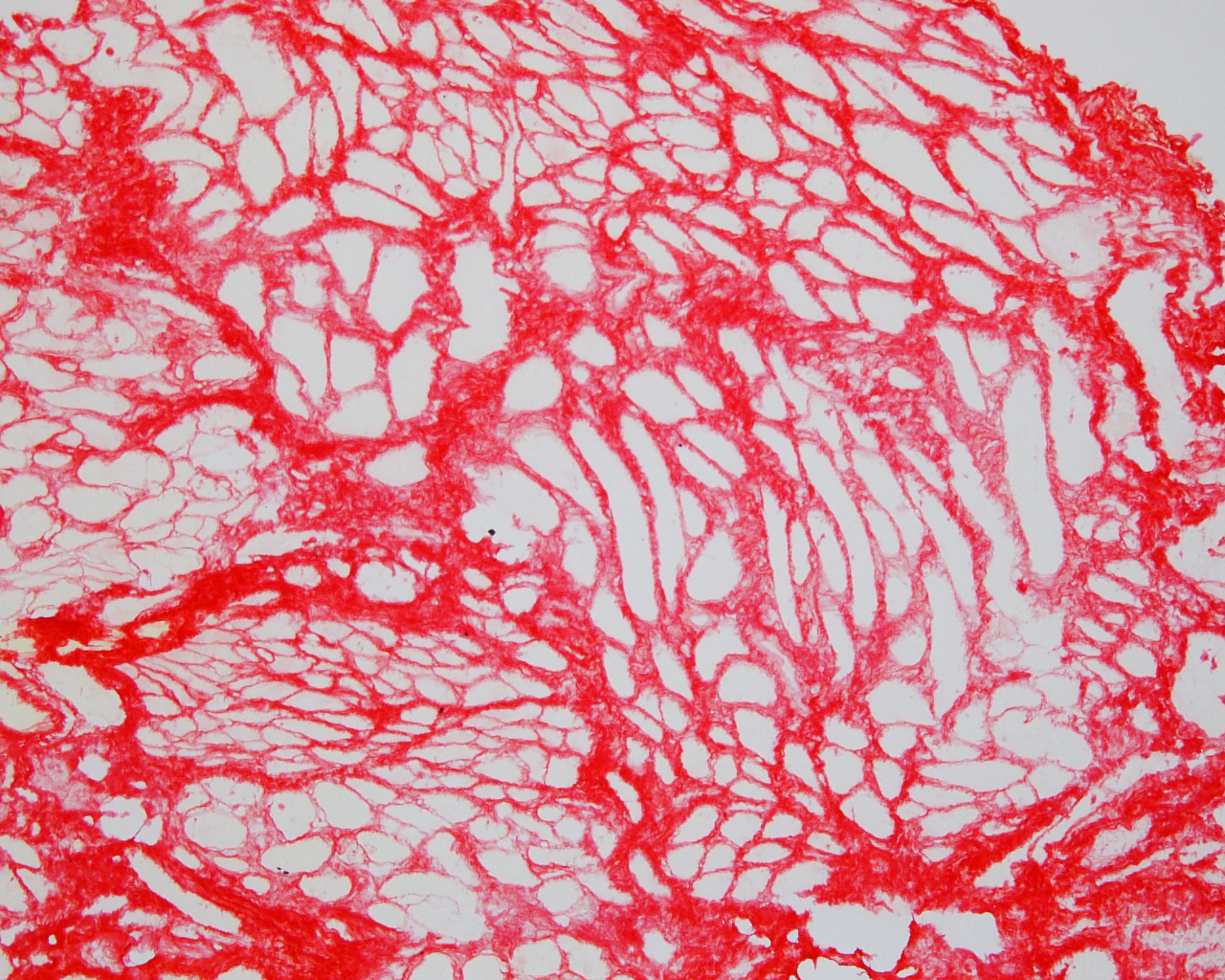

Supplement: Supplementary file 3 — Source data Fig. 1 [file 44319_2026_834_MOESM3_ESM.zip › Figure 1/1A/IBM.tif]

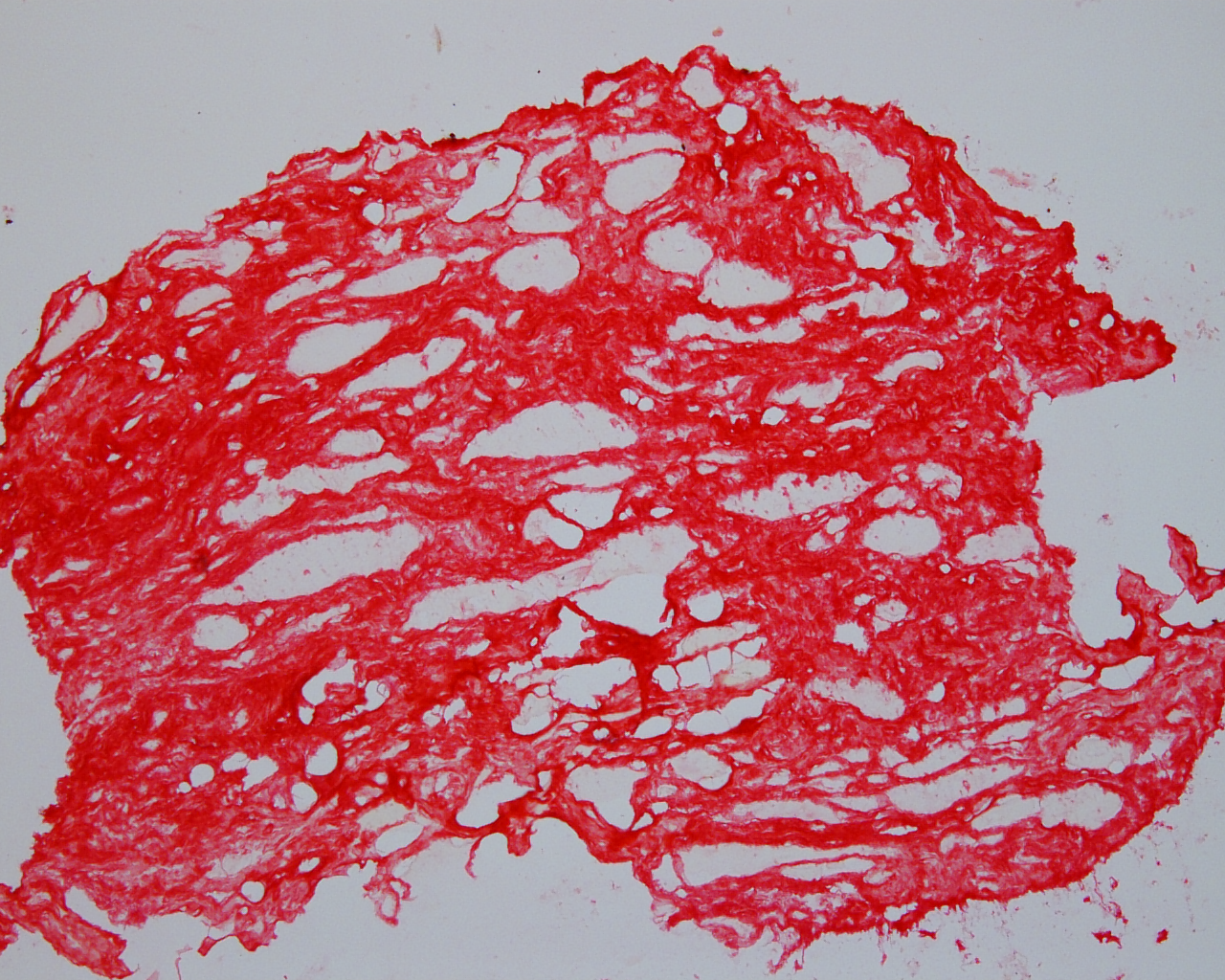

Supplement: Supplementary file 3 — Source data Fig. 1 [file 44319_2026_834_MOESM3_ESM.zip › Figure 1/1A/OPMD.tif]

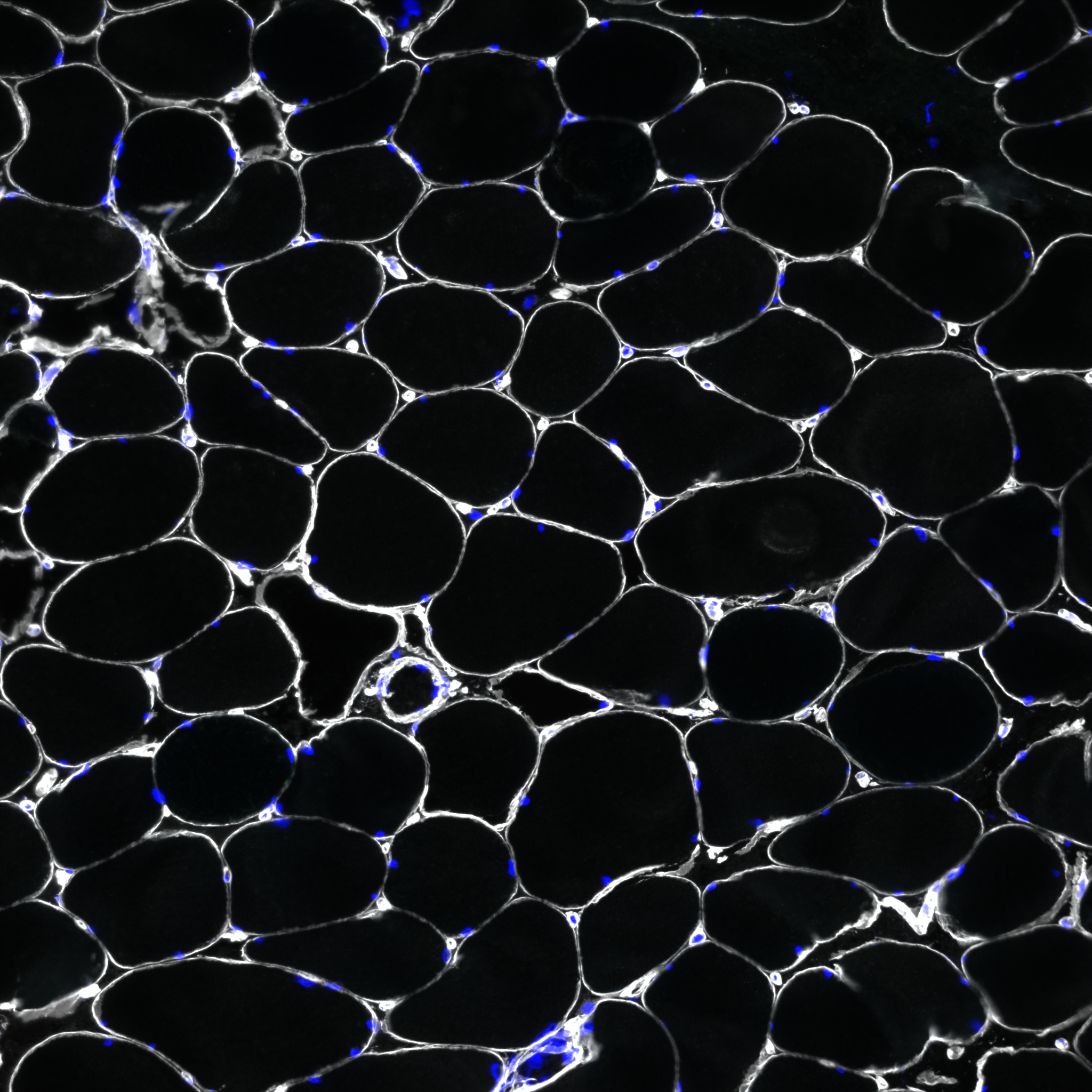

Supplement: Supplementary file 3 — Source data Fig. 1 [file 44319_2026_834_MOESM3_ESM.zip › Figure 1/1B/CTL.tif]

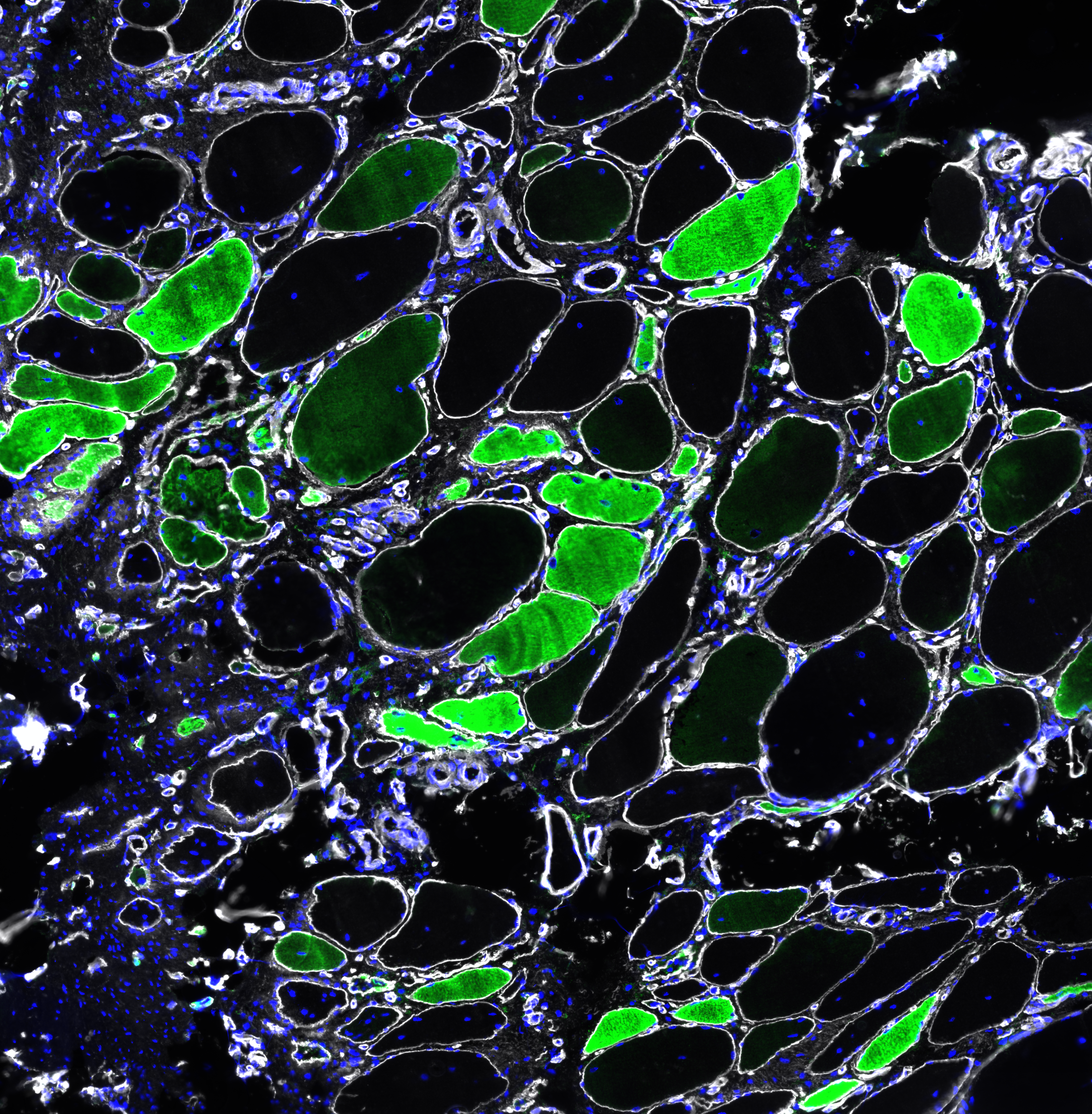

Supplement: Supplementary file 3 — Source data Fig. 1 [file 44319_2026_834_MOESM3_ESM.zip › Figure 1/1B/DMD.tif]

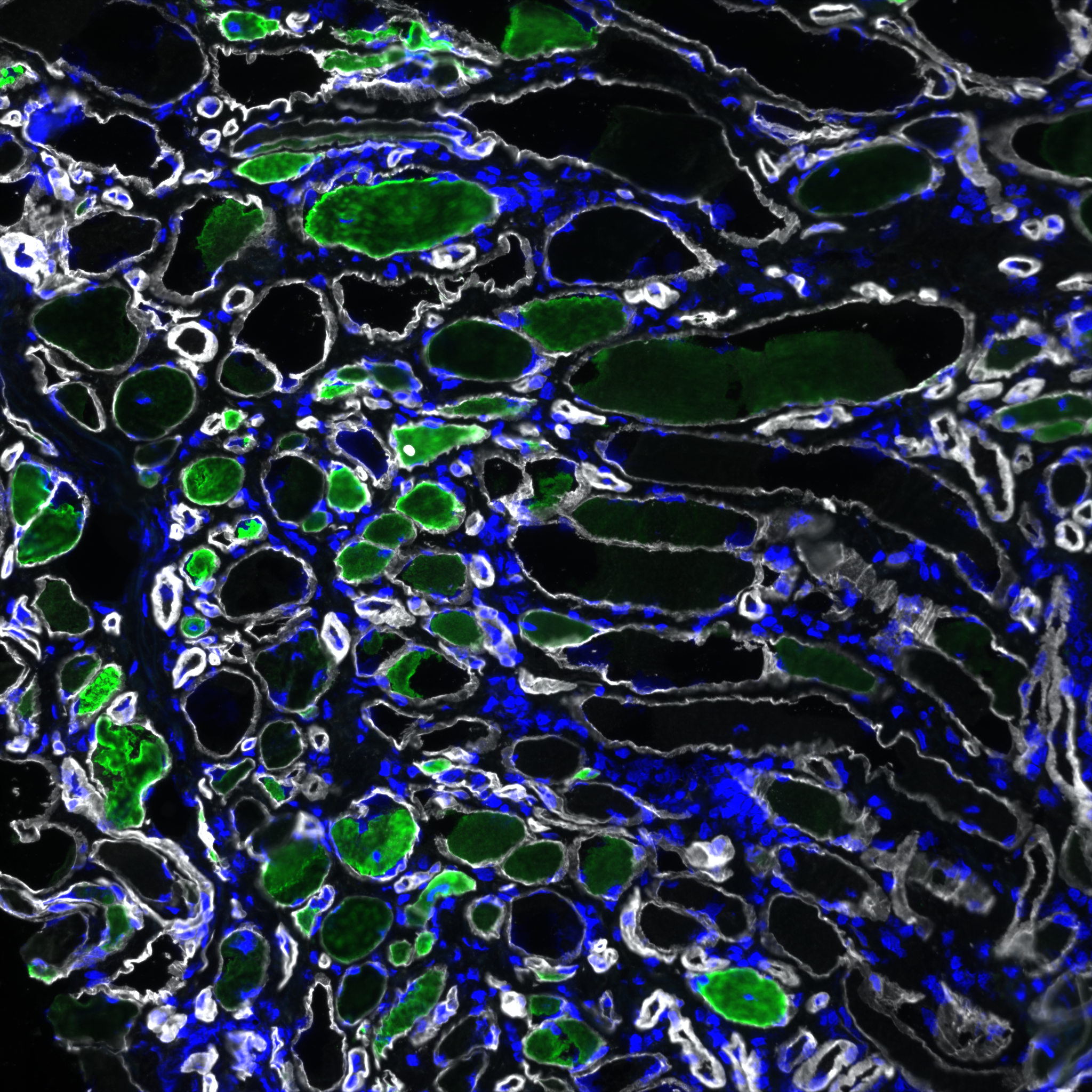

Supplement: Supplementary file 3 — Source data Fig. 1 [file 44319_2026_834_MOESM3_ESM.zip › Figure 1/1B/IBM.tif]

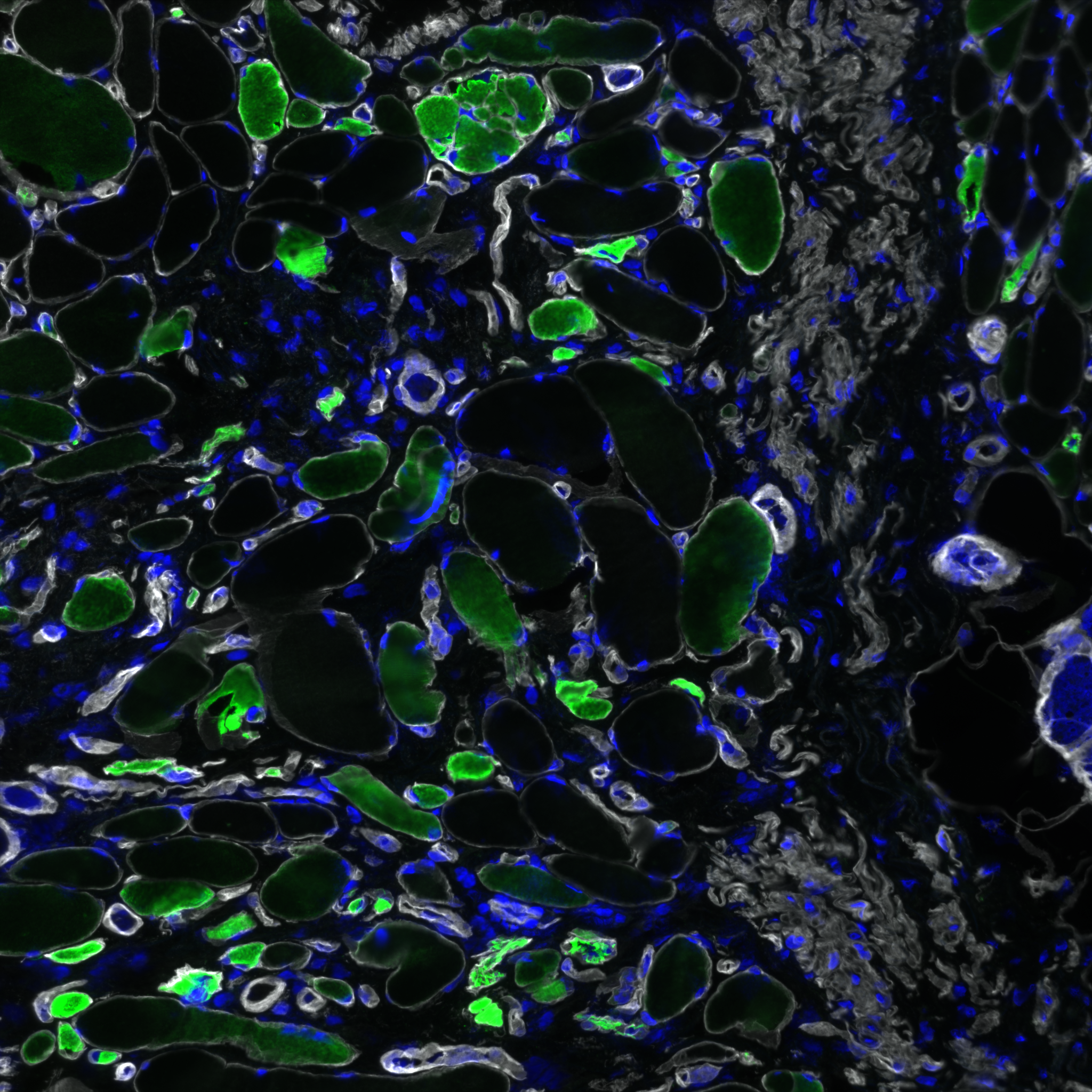

Supplement: Supplementary file 3 — Source data Fig. 1 [file 44319_2026_834_MOESM3_ESM.zip › Figure 1/1B/OPMD.tif]

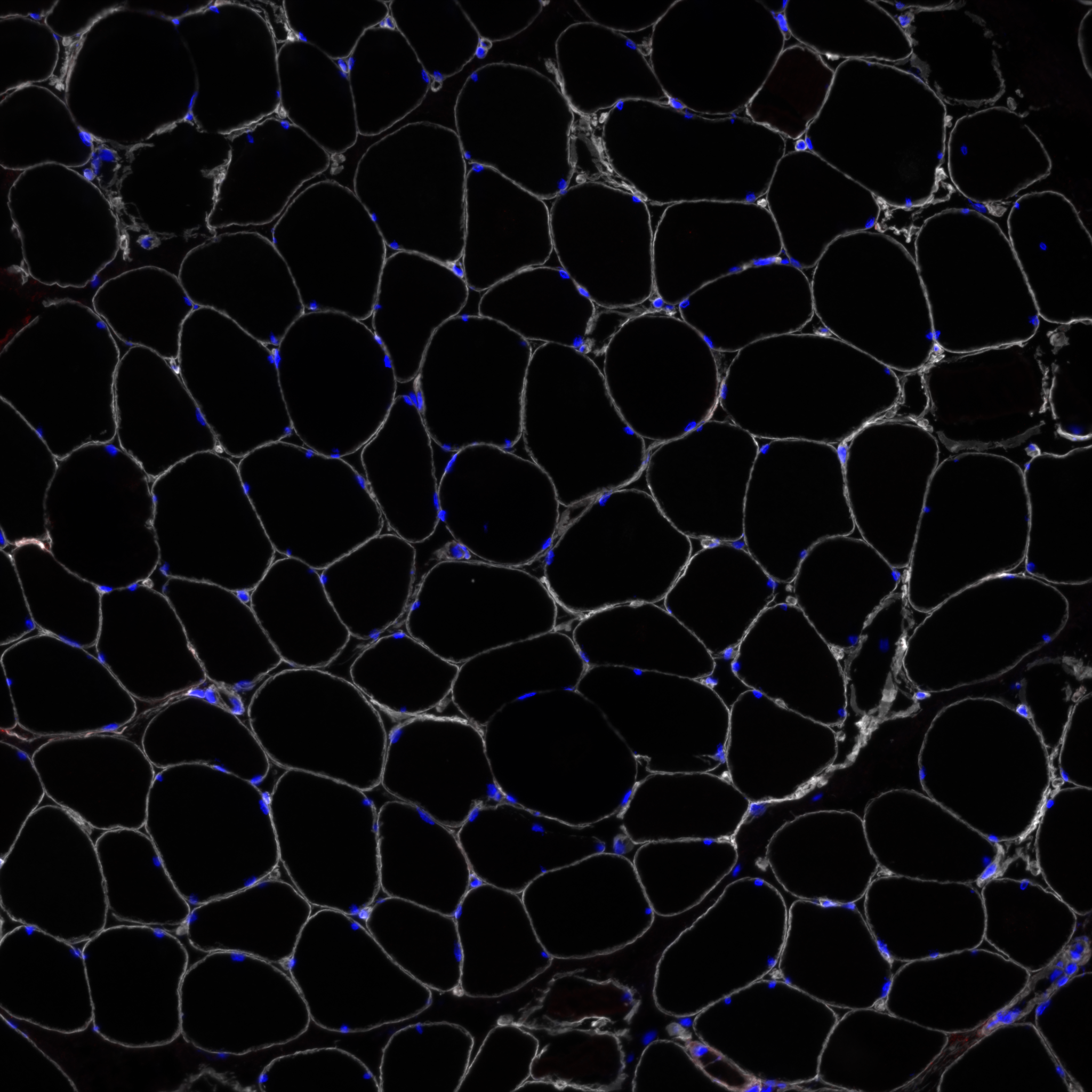

Supplement: Supplementary file 3 — Source data Fig. 1 [file 44319_2026_834_MOESM3_ESM.zip › Figure 1/1C/20230215 27 560 PVCT CD90555 laminin 647 dapi x20 3_c1-3.tif]

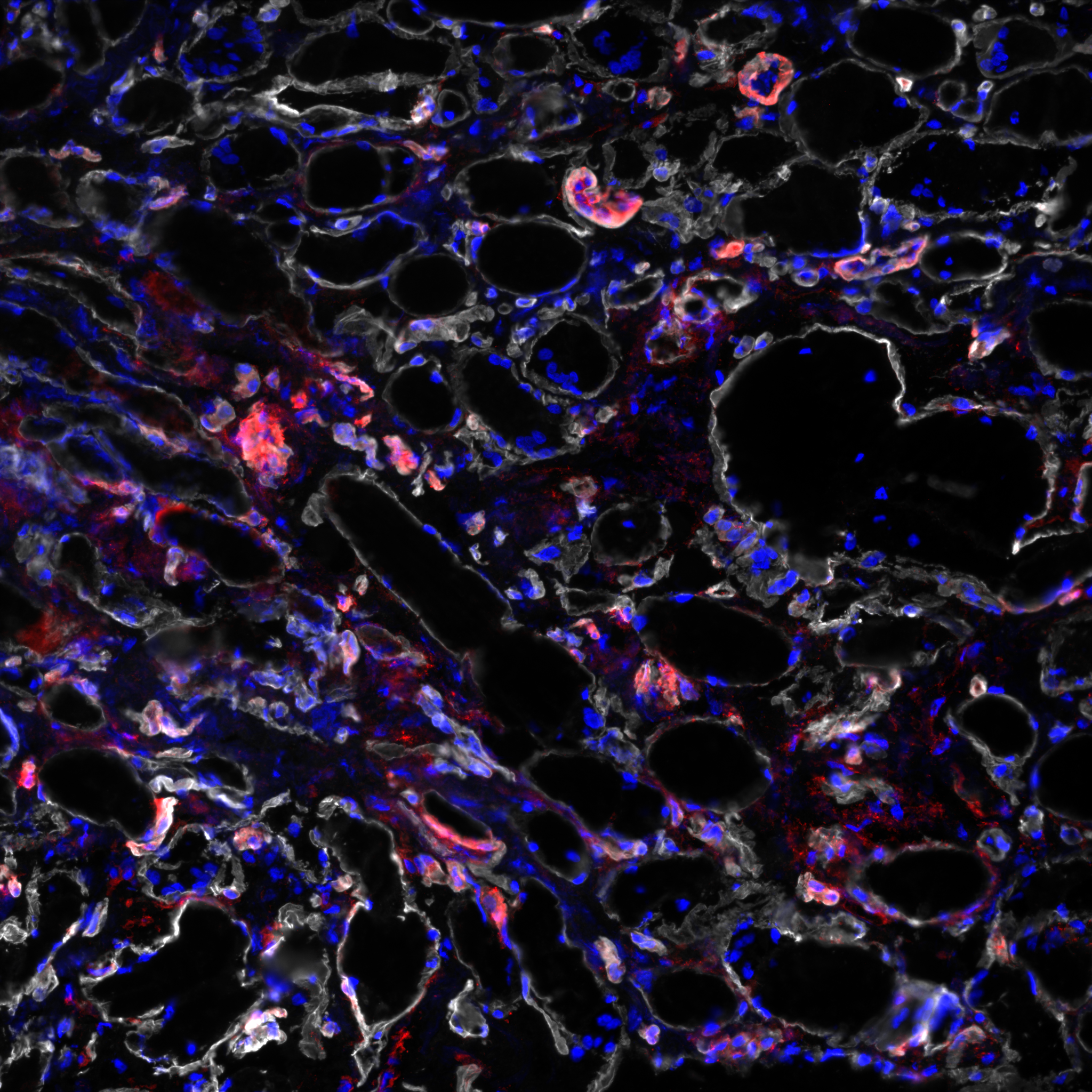

Supplement: Supplementary file 3 — Source data Fig. 1 [file 44319_2026_834_MOESM3_ESM.zip › Figure 1/1C/20230215 ABo1435OP CD90555 laminin 647 dapi x20 3_c1-3.tif]

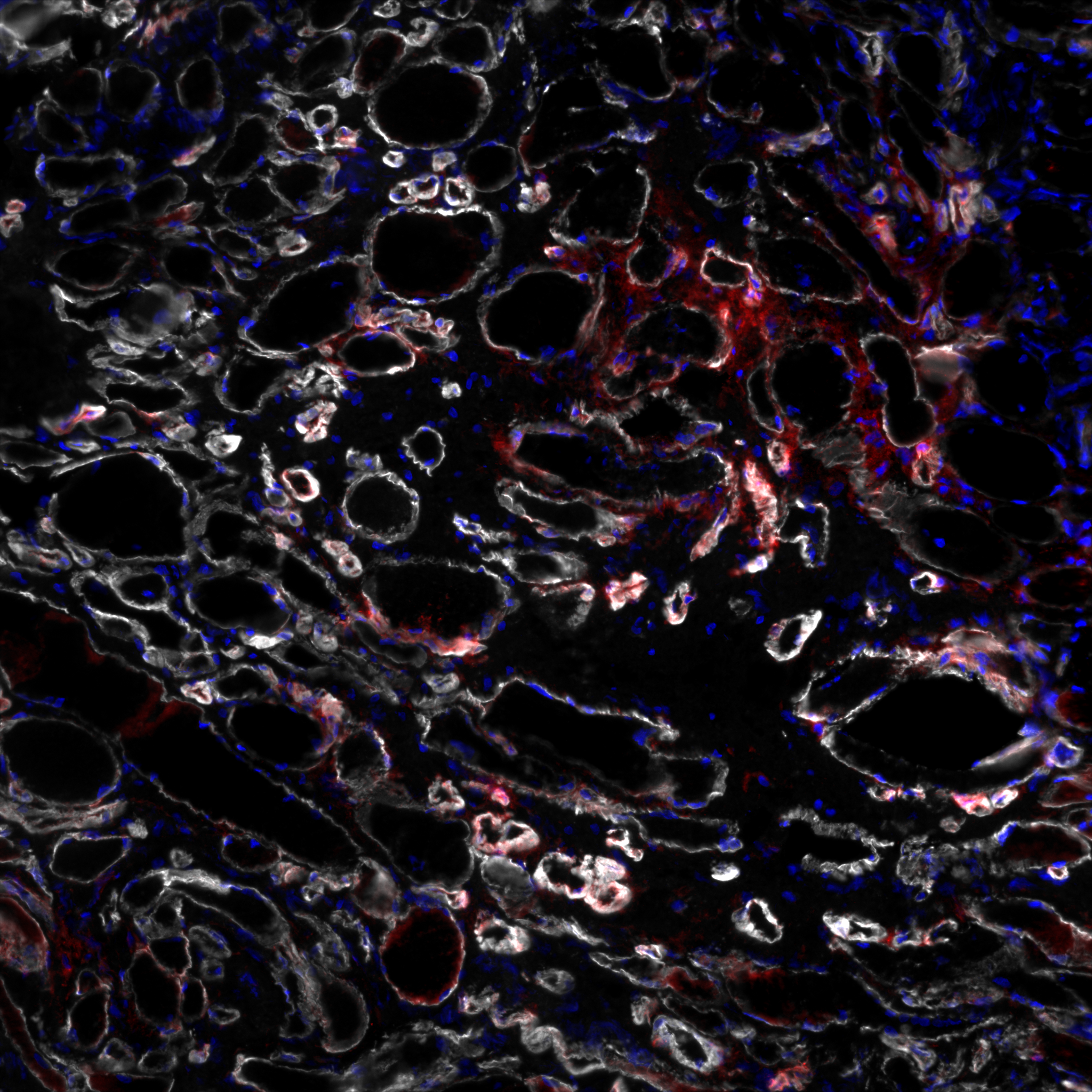

Supplement: Supplementary file 3 — Source data Fig. 1 [file 44319_2026_834_MOESM3_ESM.zip › Figure 1/1C/20230215 EN1370IBM CD90555 laminin 647 dapi x20 2_c1-3.tif]

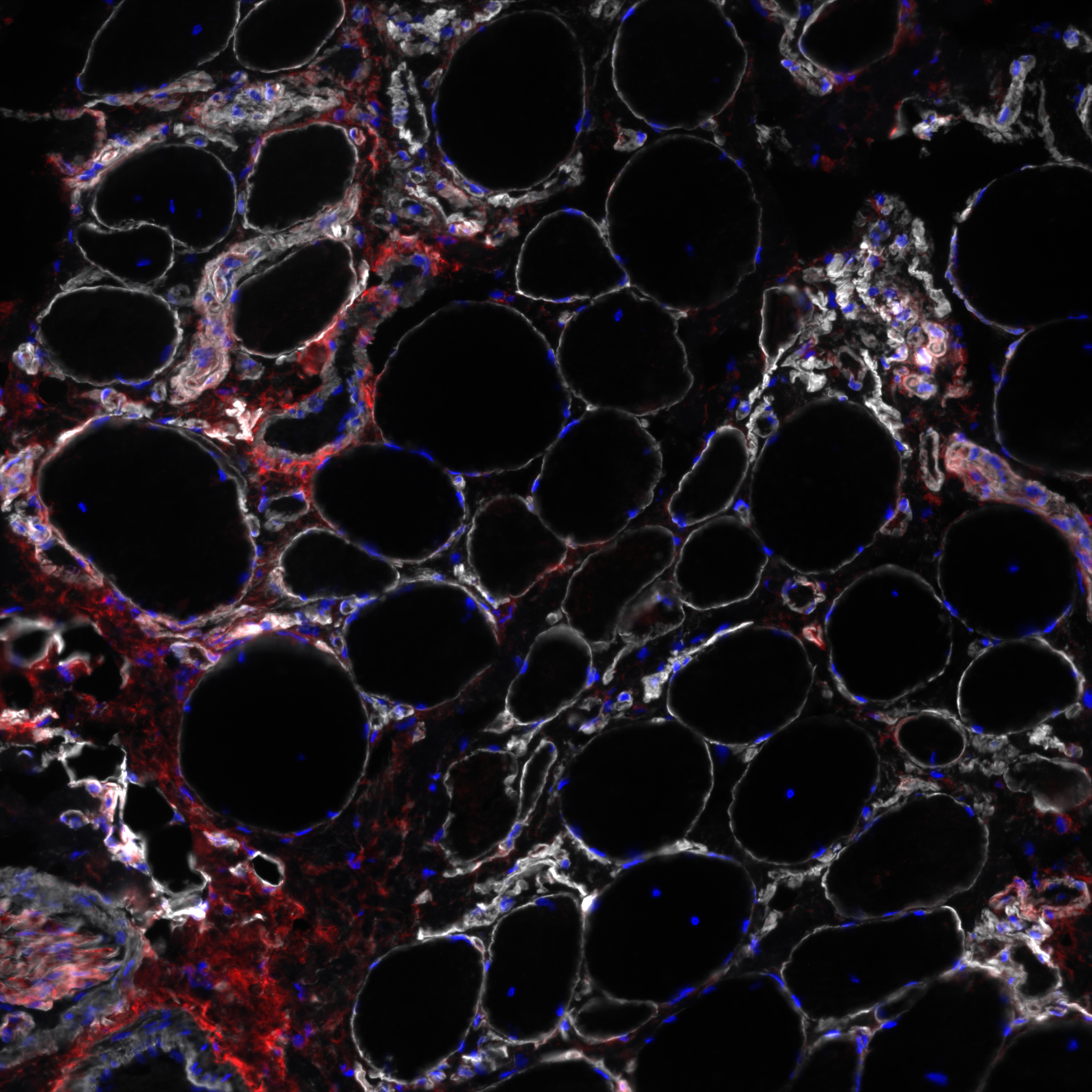

Supplement: Supplementary file 3 — Source data Fig. 1 [file 44319_2026_834_MOESM3_ESM.zip › Figure 1/1C/20230215 EN696DMD CD90555 laminin 647 dapi x20.czi 2_c1-3.tif]

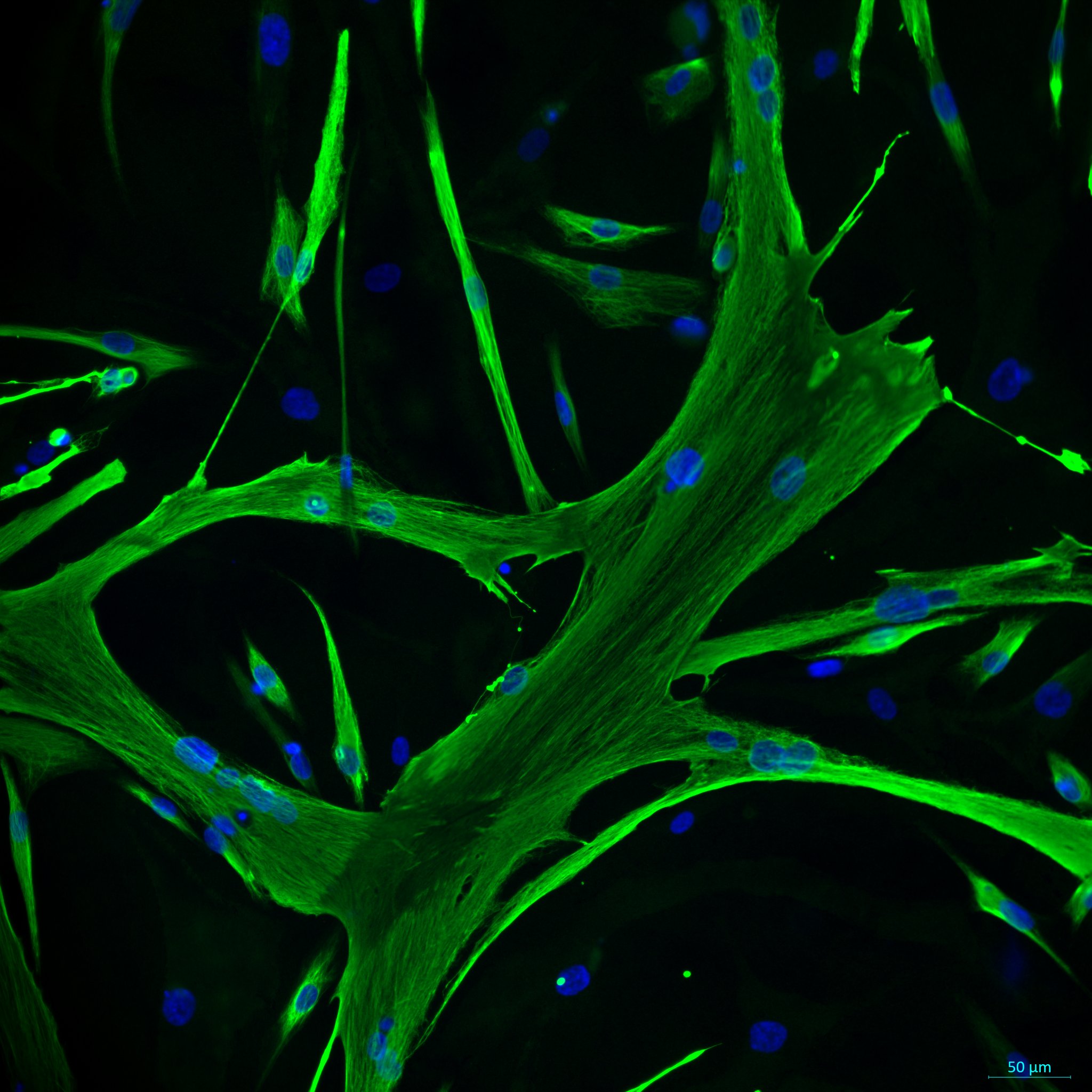

Supplement: Supplementary file 3 — Source data Fig. 1 [file 44319_2026_834_MOESM3_ESM.zip › Figure 1/1F/+CTL FAPs.jpg]

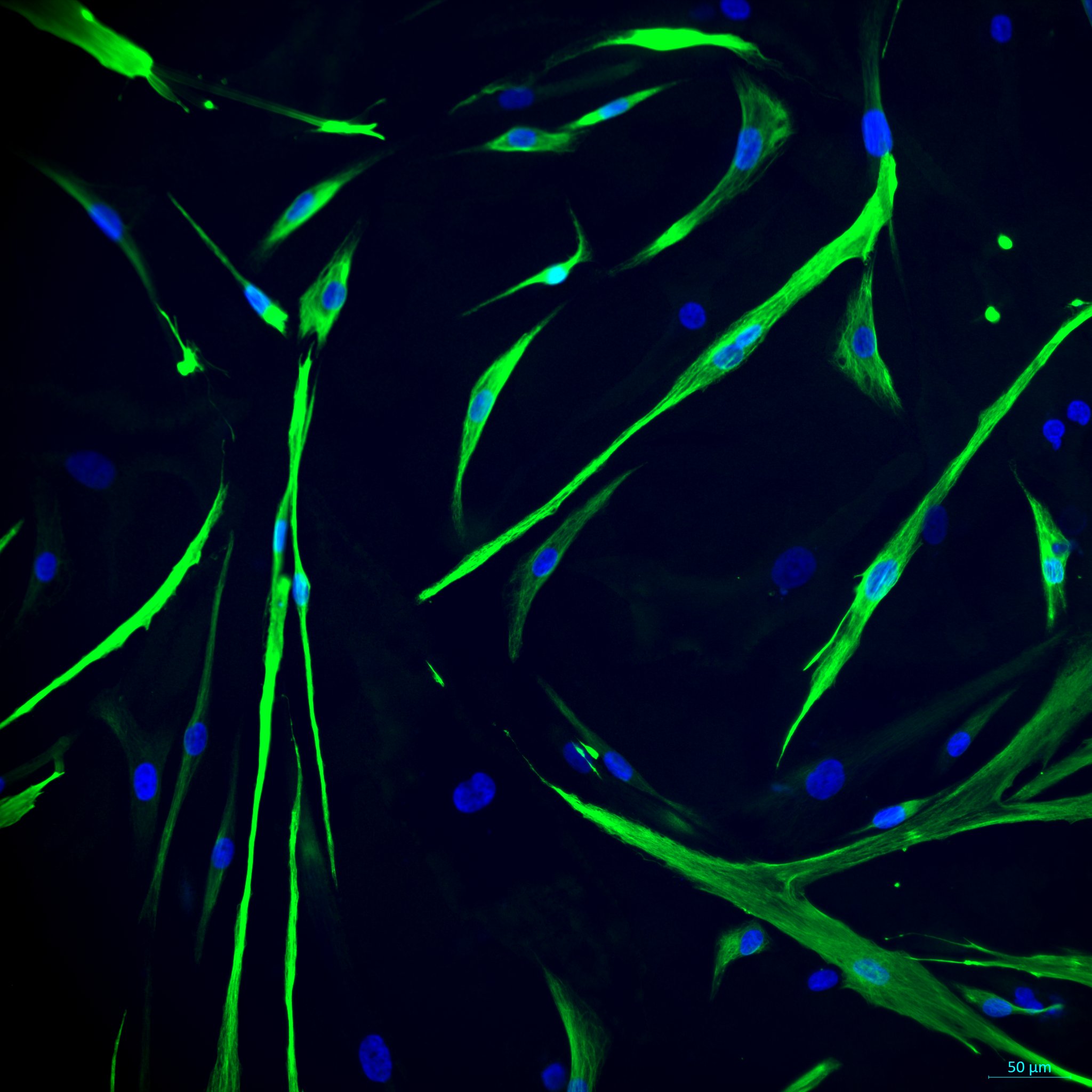

Supplement: Supplementary file 3 — Source data Fig. 1 [file 44319_2026_834_MOESM3_ESM.zip › Figure 1/1F/+DMD FAPs.jpg]

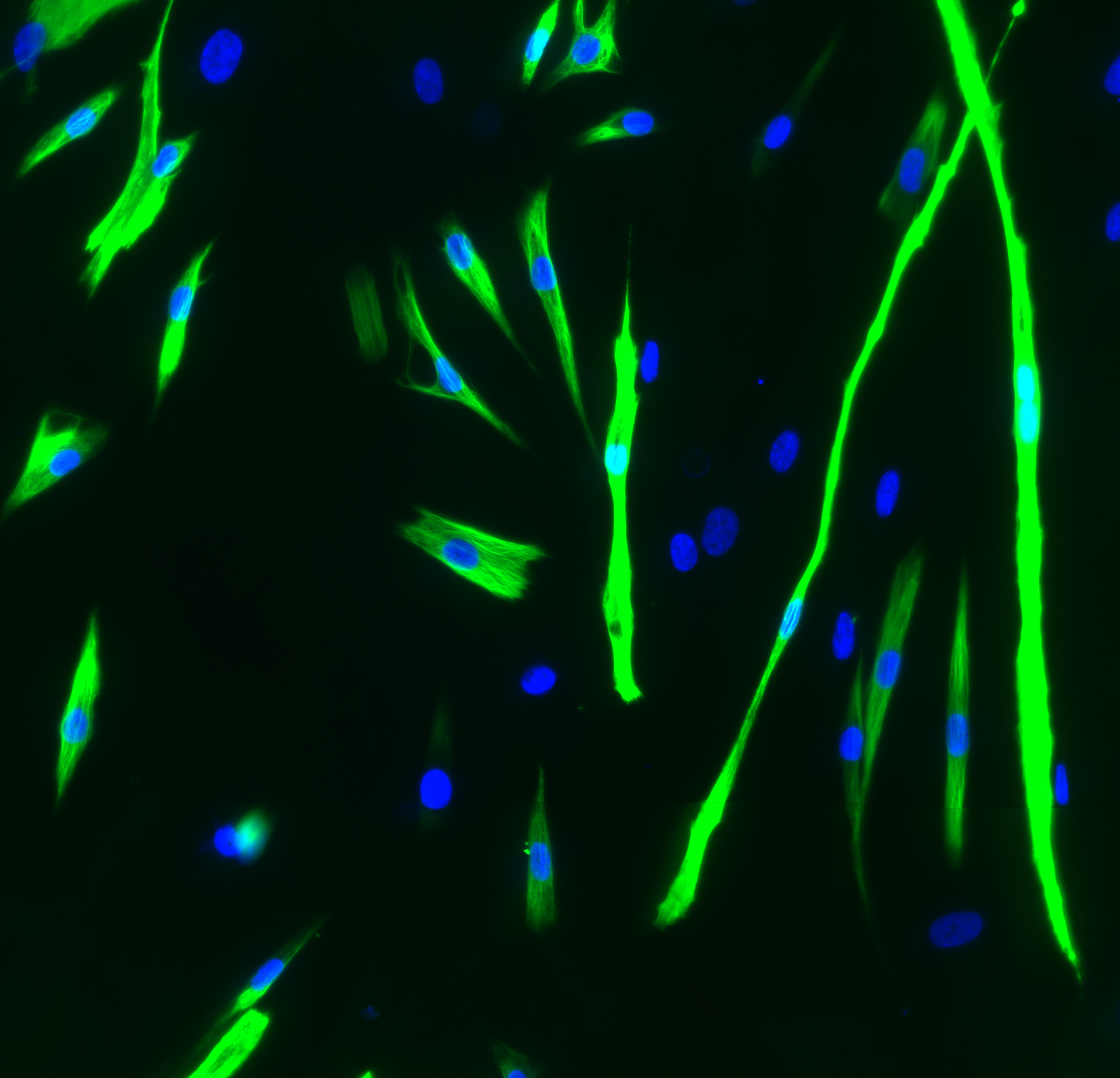

Supplement: Supplementary file 3 — Source data Fig. 1 [file 44319_2026_834_MOESM3_ESM.zip › Figure 1/1F/+IBM FAPs.tif]

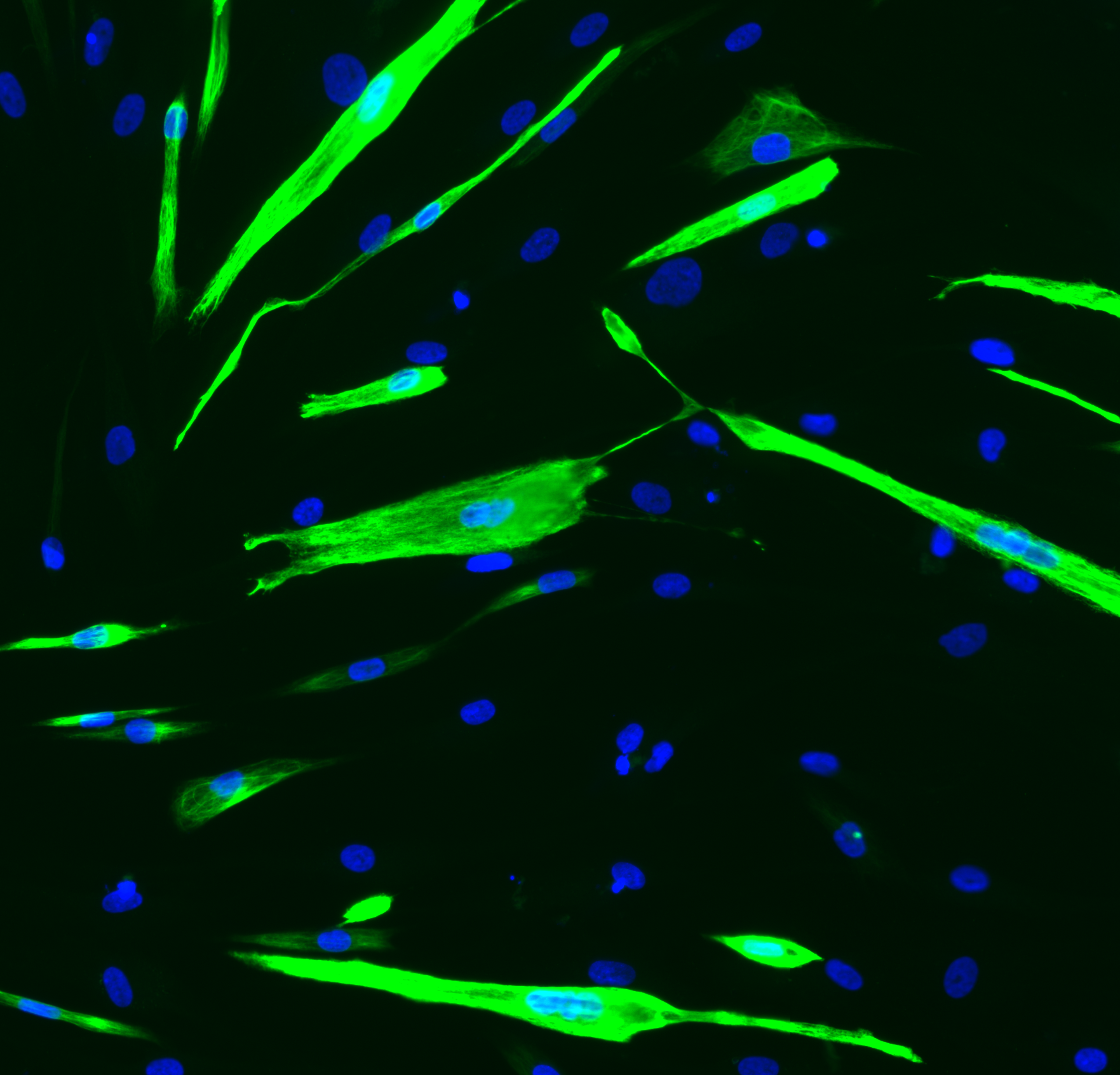

Supplement: Supplementary file 3 — Source data Fig. 1 [file 44319_2026_834_MOESM3_ESM.zip › Figure 1/1F/+OPMD FAPs.tif]

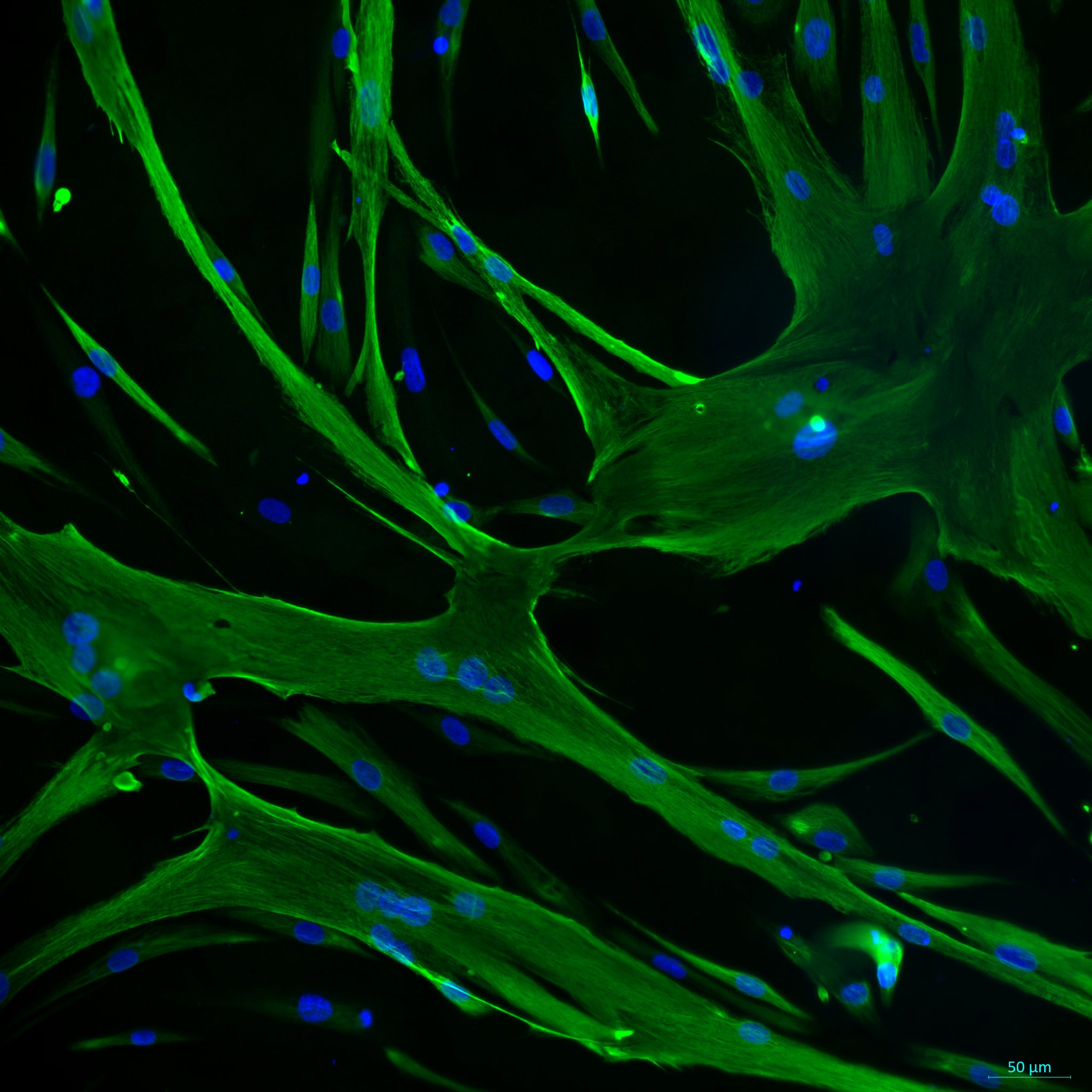

Supplement: Supplementary file 3 — Source data Fig. 1 [file 44319_2026_834_MOESM3_ESM.zip › Figure 1/1F/w:o FAPs.jpg]

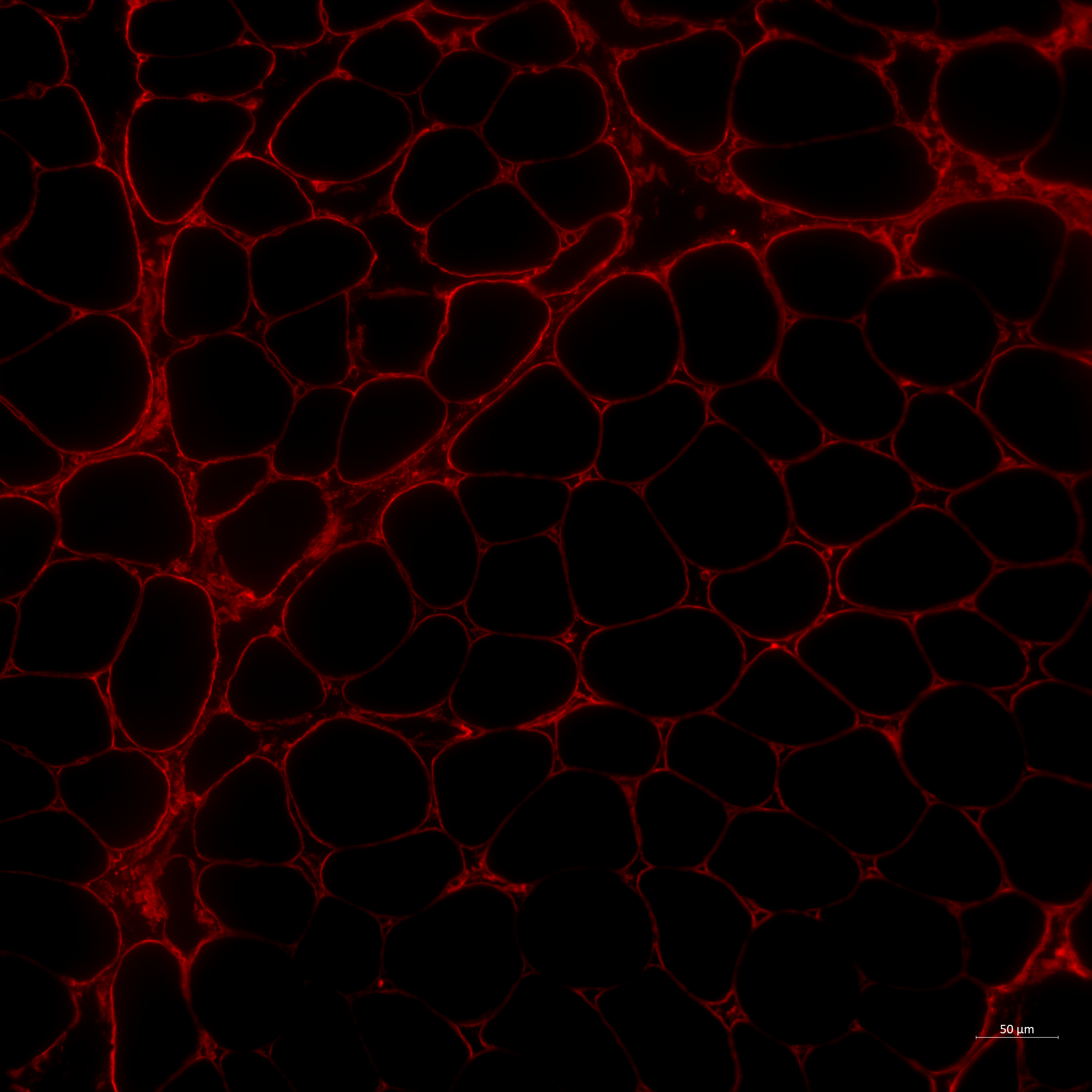

Supplement: Supplementary file 5 — Source data Fig. 3 [file 44319_2026_834_MOESM5_ESM.zip › Figure 3/3D/CTL COLVI.tif]

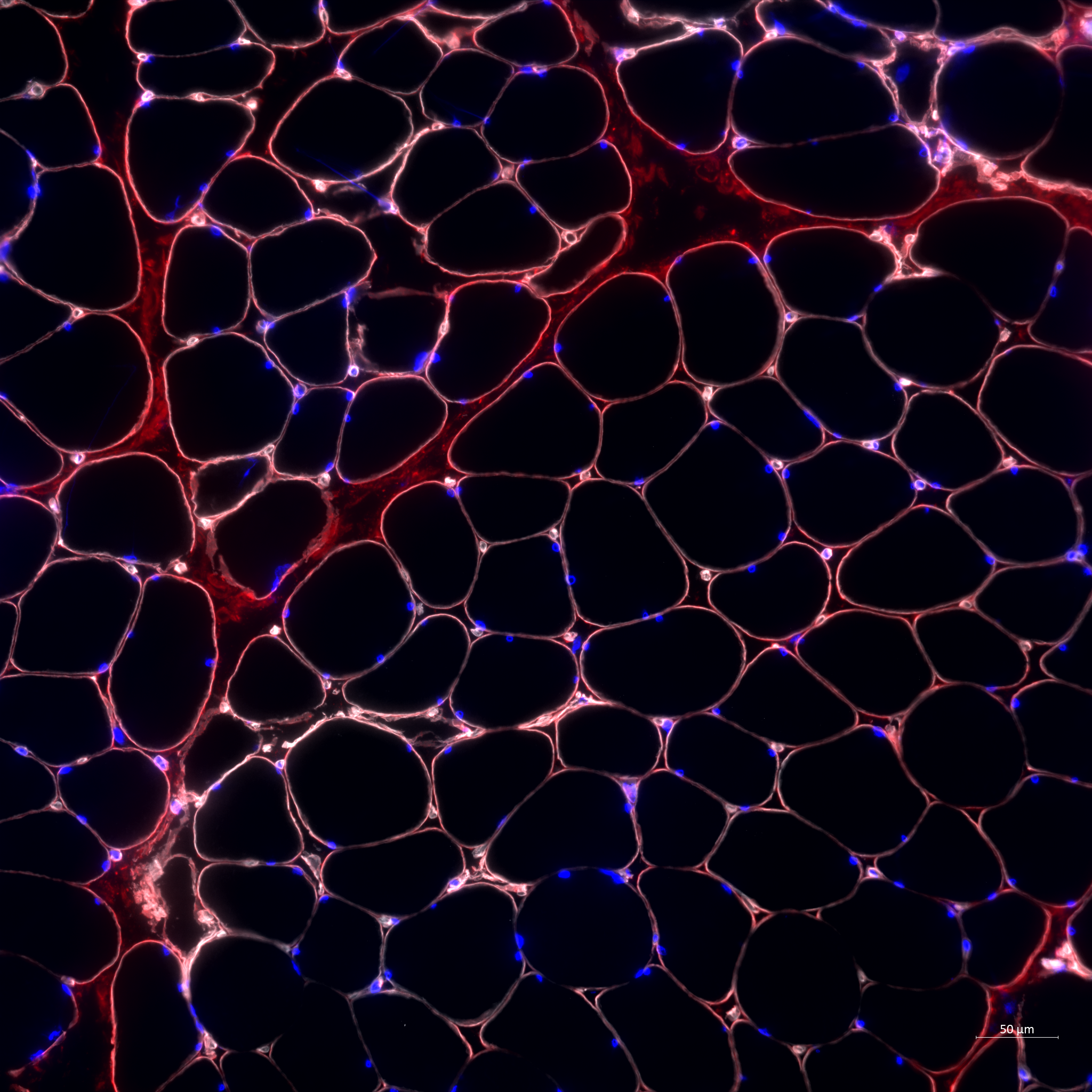

Supplement: Supplementary file 5 — Source data Fig. 3 [file 44319_2026_834_MOESM5_ESM.zip › Figure 3/3D/CTL laminin COLVI.tif]

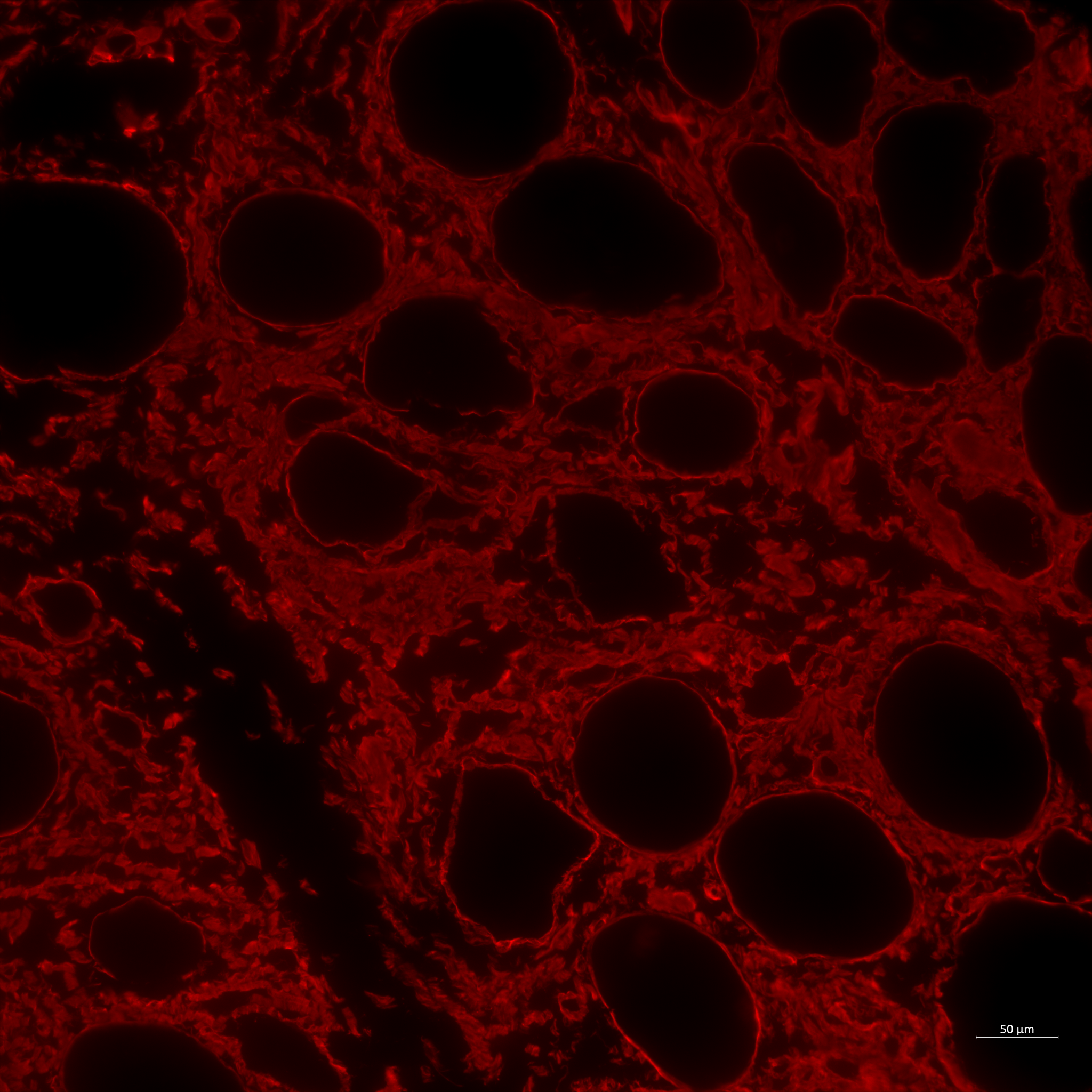

Supplement: Supplementary file 5 — Source data Fig. 3 [file 44319_2026_834_MOESM5_ESM.zip › Figure 3/3D/DMD COLVI.tif]

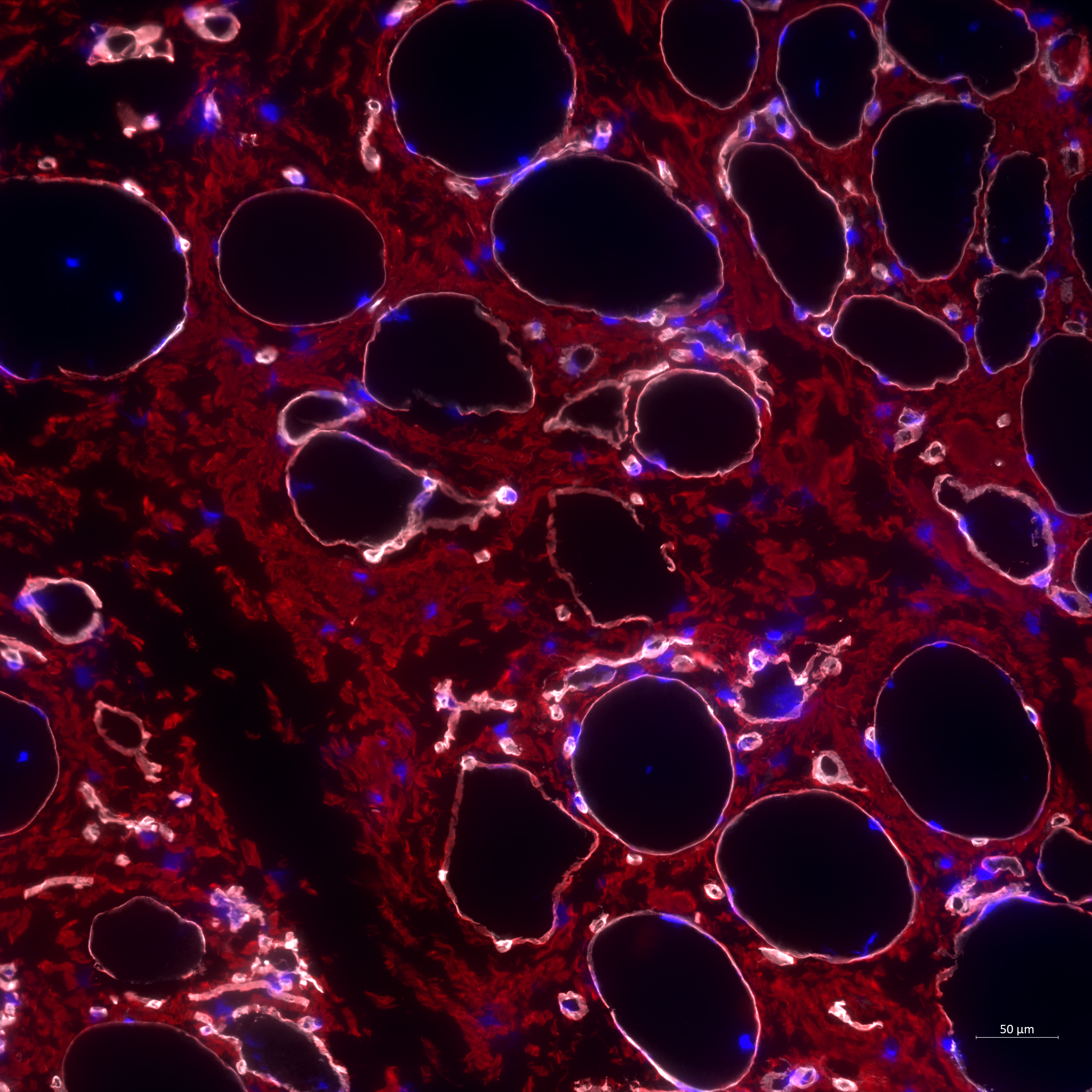

Supplement: Supplementary file 5 — Source data Fig. 3 [file 44319_2026_834_MOESM5_ESM.zip › Figure 3/3D/DMD laminin COLVI.tif]

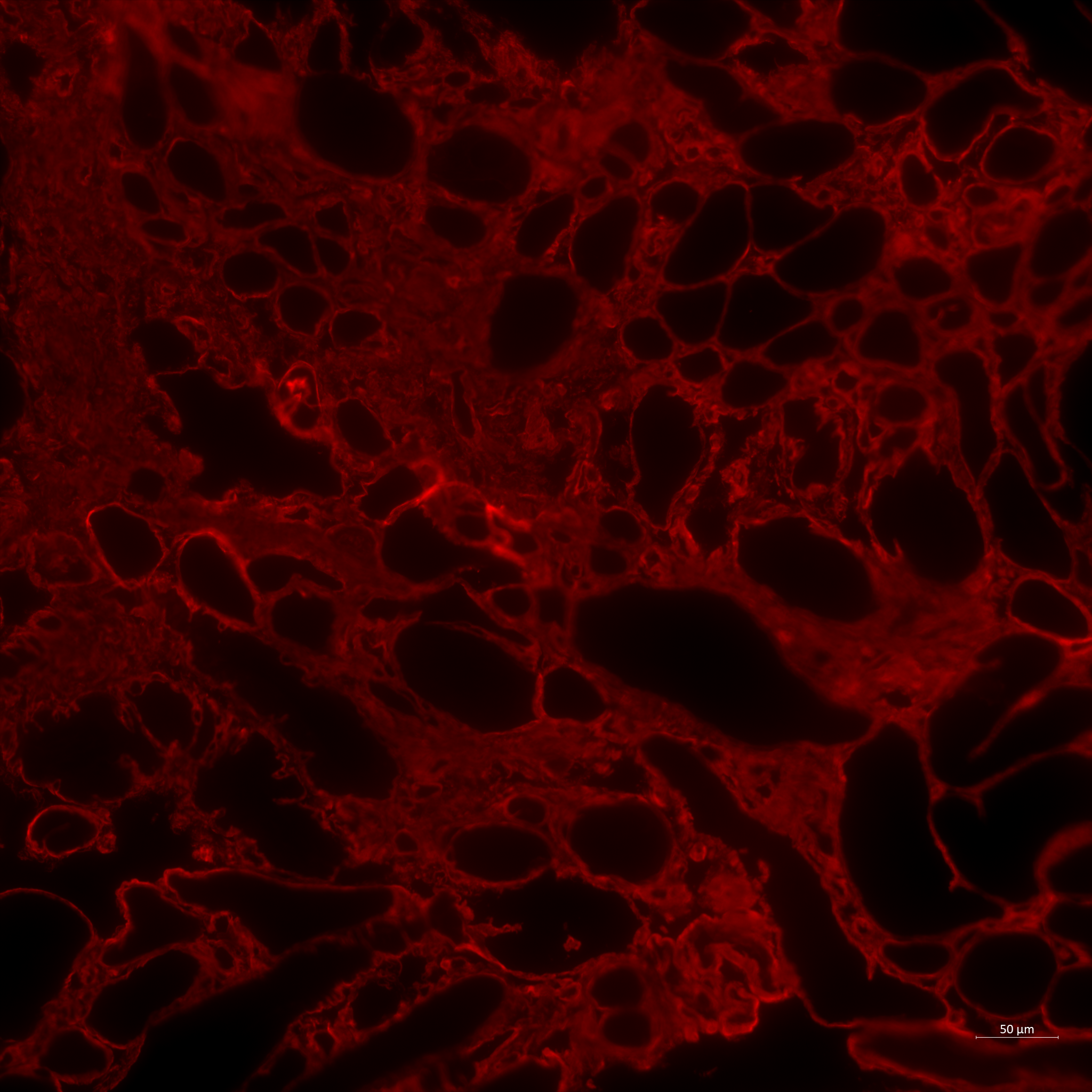

Supplement: Supplementary file 5 — Source data Fig. 3 [file 44319_2026_834_MOESM5_ESM.zip › Figure 3/3D/IBM COLVI.tif]

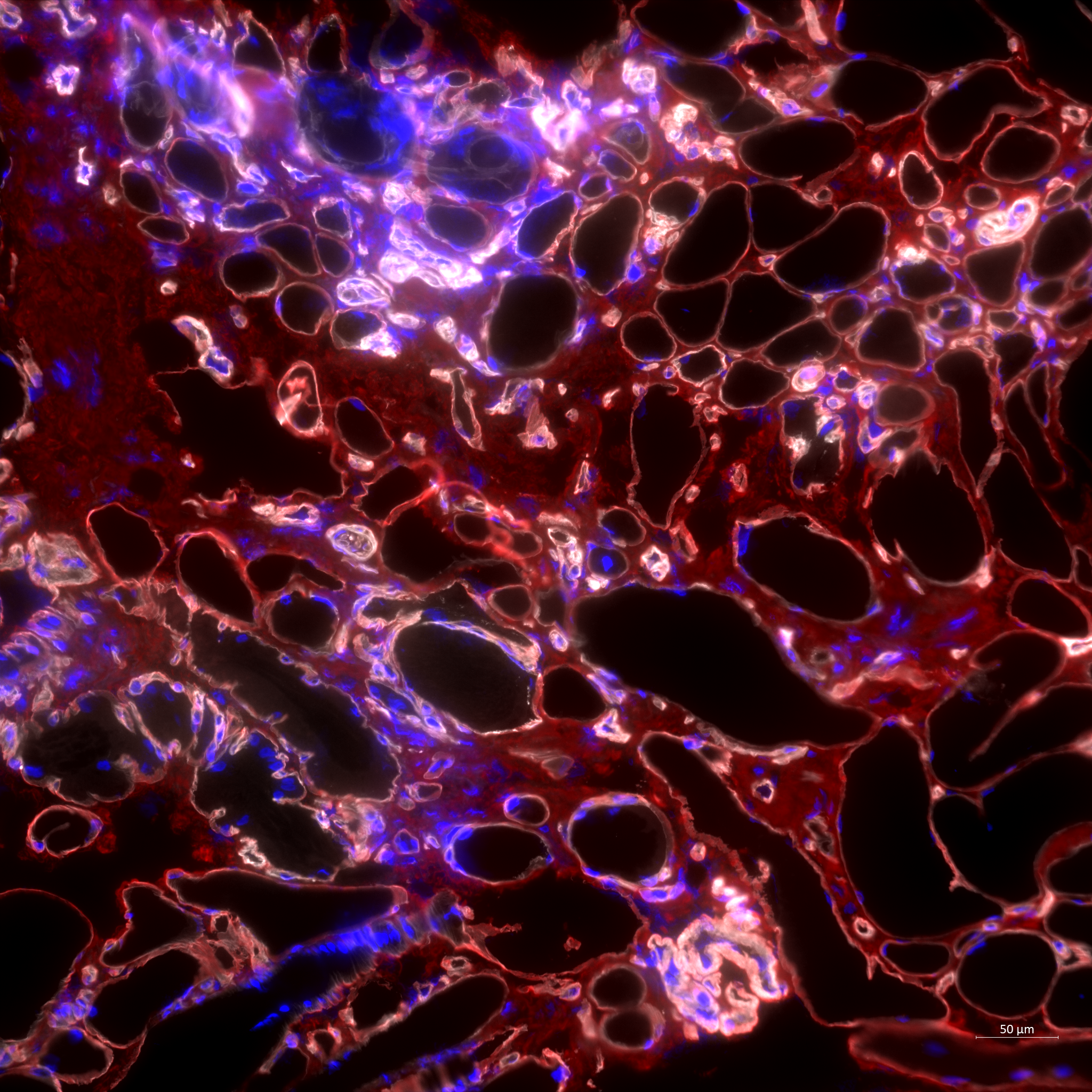

Supplement: Supplementary file 5 — Source data Fig. 3 [file 44319_2026_834_MOESM5_ESM.zip › Figure 3/3D/IBM laminin COLVI.tif]

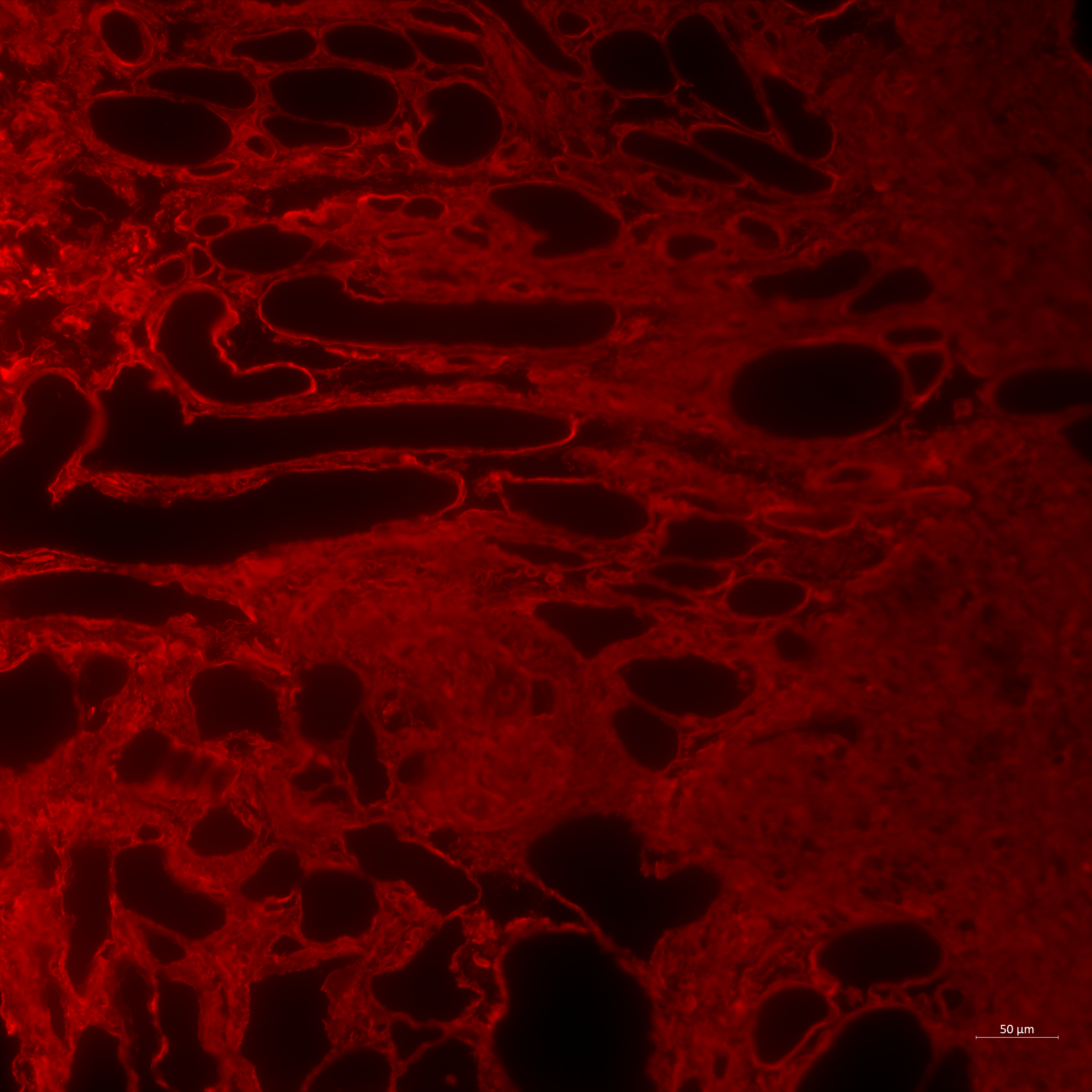

Supplement: Supplementary file 5 — Source data Fig. 3 [file 44319_2026_834_MOESM5_ESM.zip › Figure 3/3D/OPMD COLVI.tif]

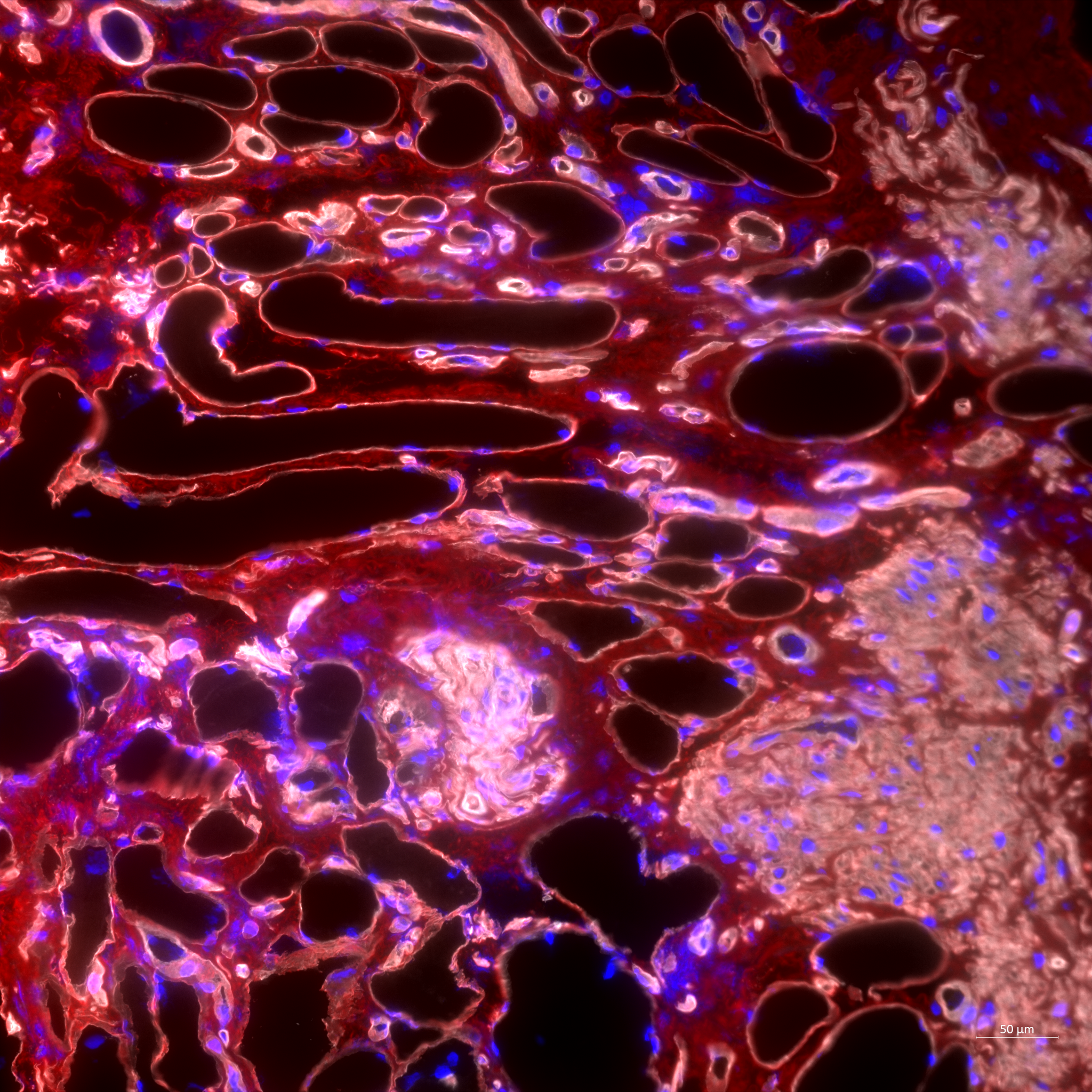

Supplement: Supplementary file 5 — Source data Fig. 3 [file 44319_2026_834_MOESM5_ESM.zip › Figure 3/3D/OPMD laminin COLVI.tif]

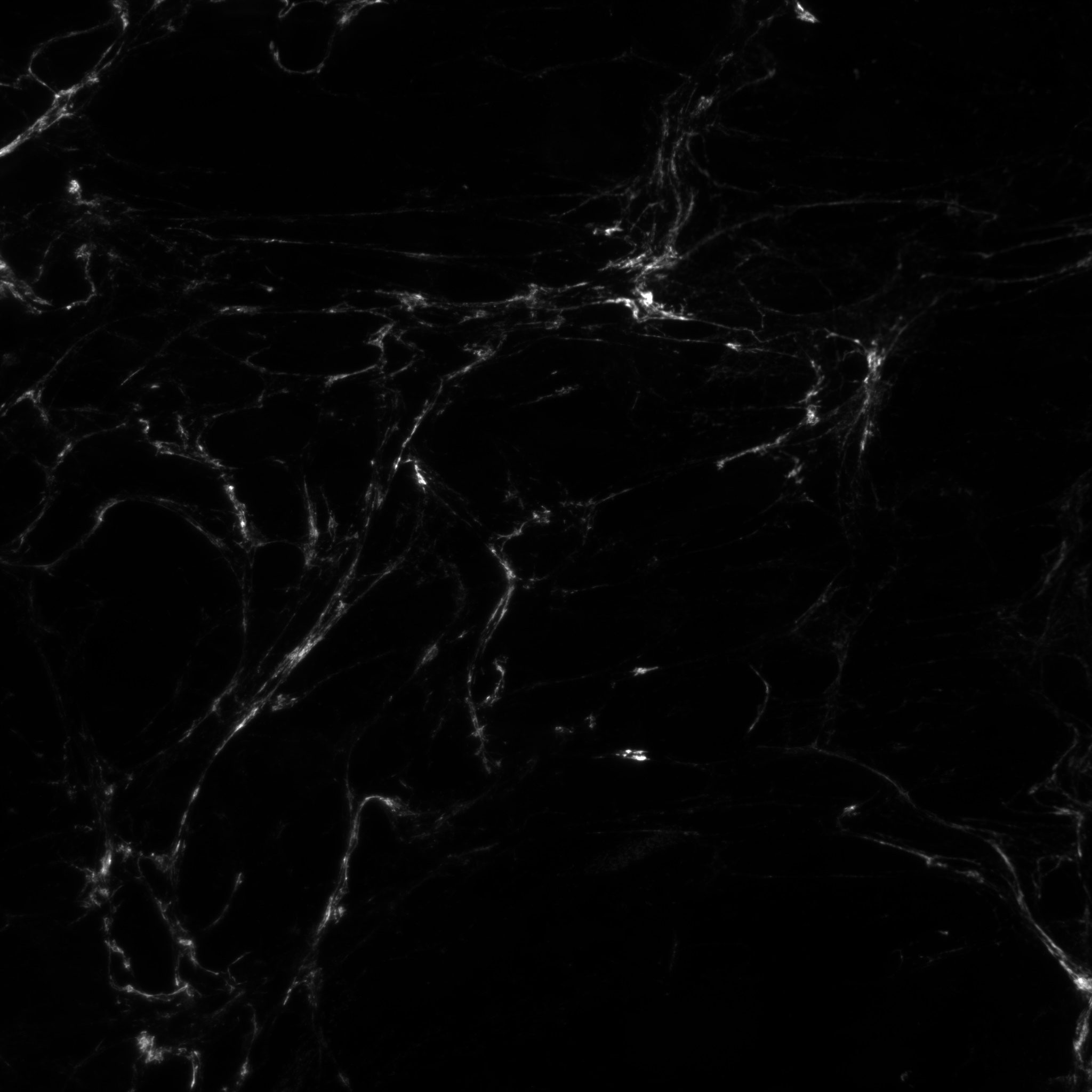

Supplement: Supplementary file 5 — Source data Fig. 3 [file 44319_2026_834_MOESM5_ESM.zip › Figure 3/3F/CTL COL4.jpg]

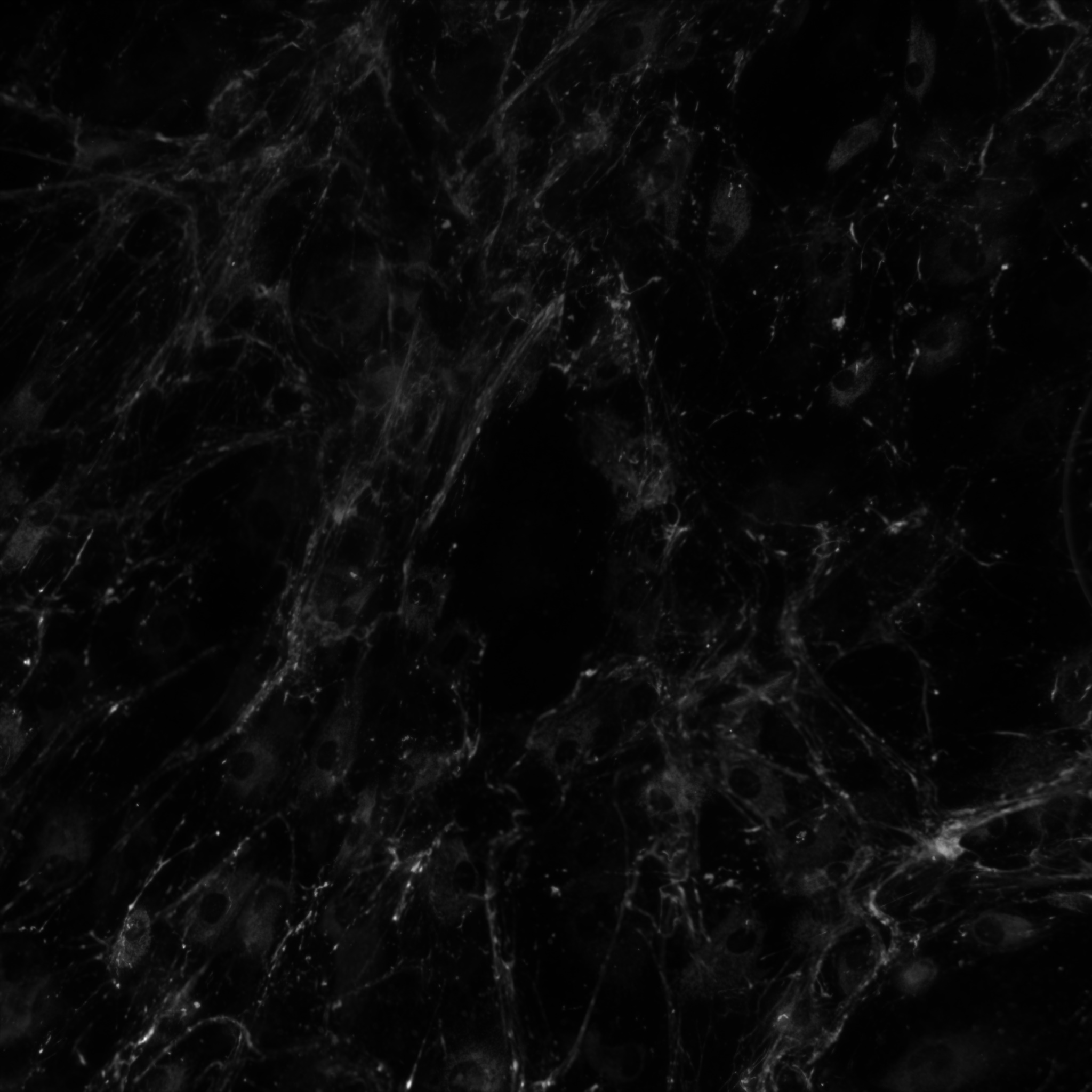

Supplement: Supplementary file 5 — Source data Fig. 3 [file 44319_2026_834_MOESM5_ESM.zip › Figure 3/3F/CTL COL6.jpg]

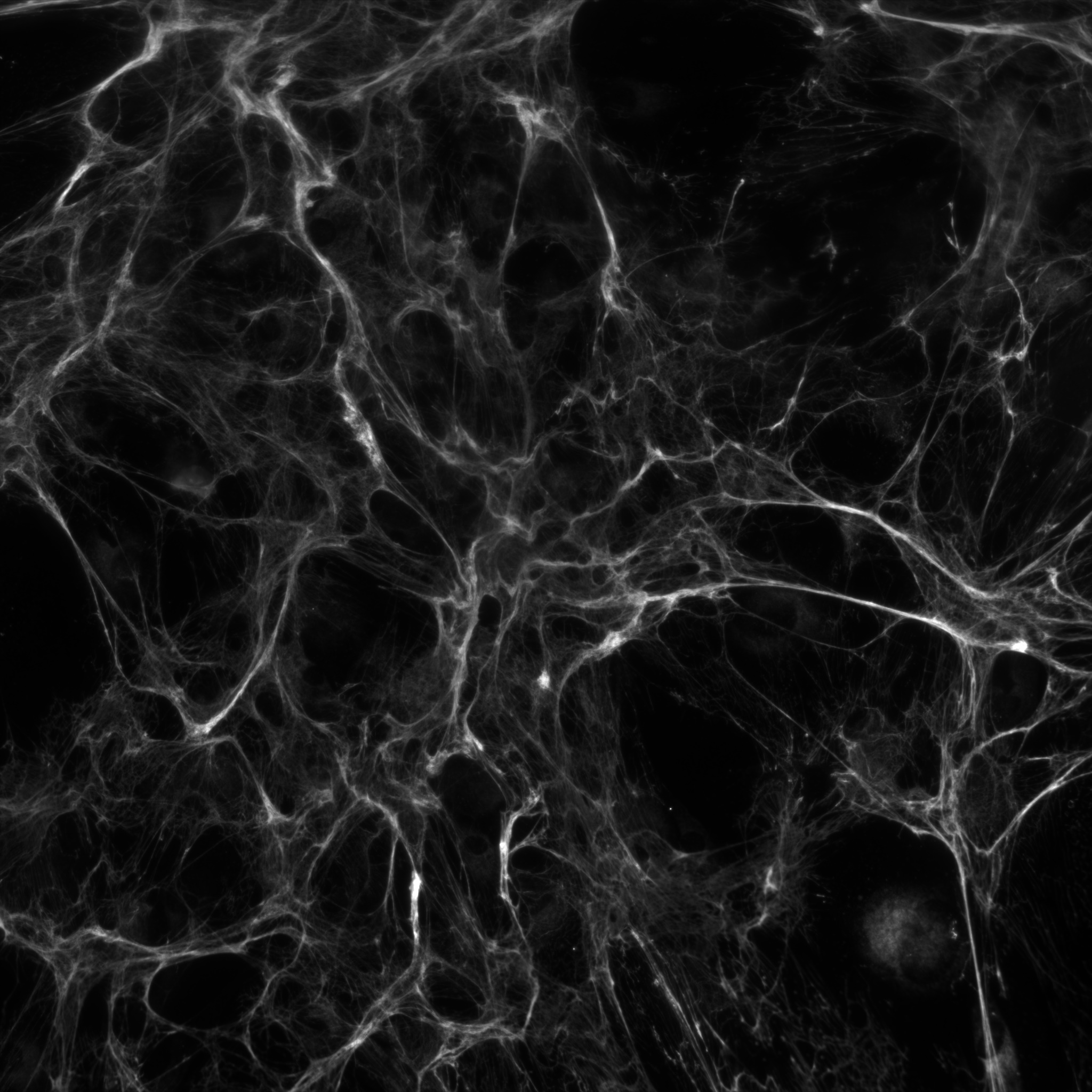

Supplement: Supplementary file 5 — Source data Fig. 3 [file 44319_2026_834_MOESM5_ESM.zip › Figure 3/3F/CTL FN1.jpg]

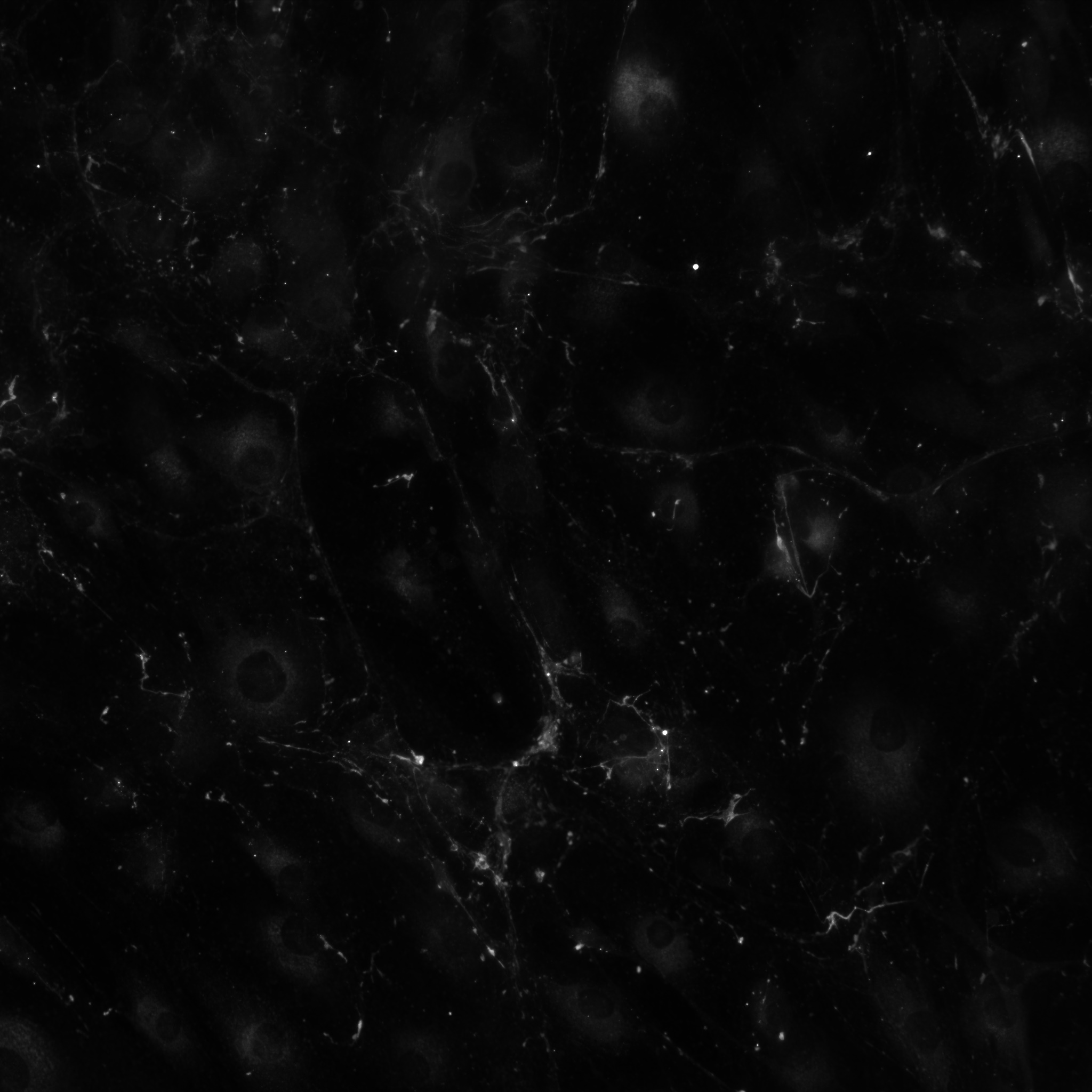

Supplement: Supplementary file 5 — Source data Fig. 3 [file 44319_2026_834_MOESM5_ESM.zip › Figure 3/3F/CTL TNXB.jpg]

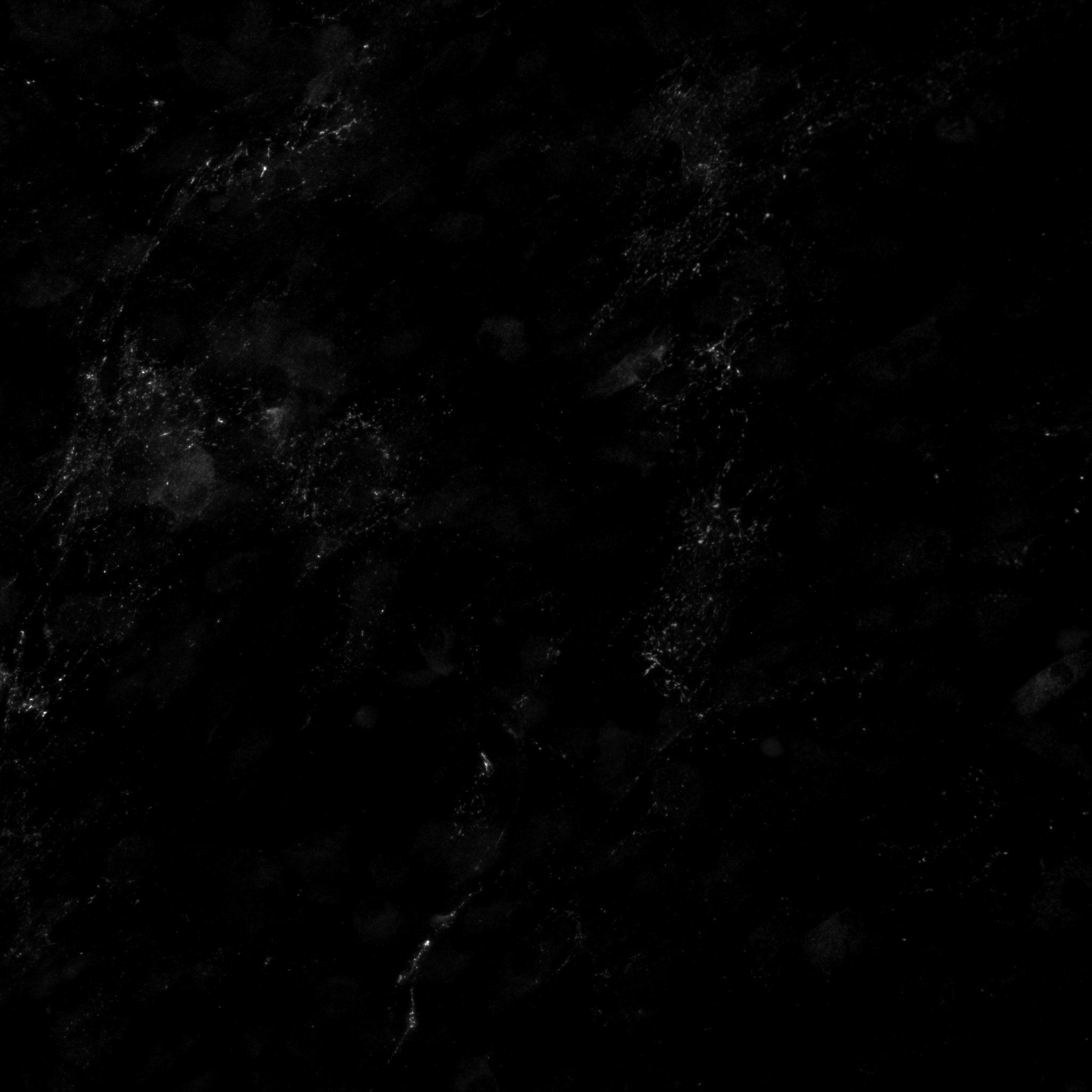

Supplement: Supplementary file 5 — Source data Fig. 3 [file 44319_2026_834_MOESM5_ESM.zip › Figure 3/3F/DMD COL4.jpg]

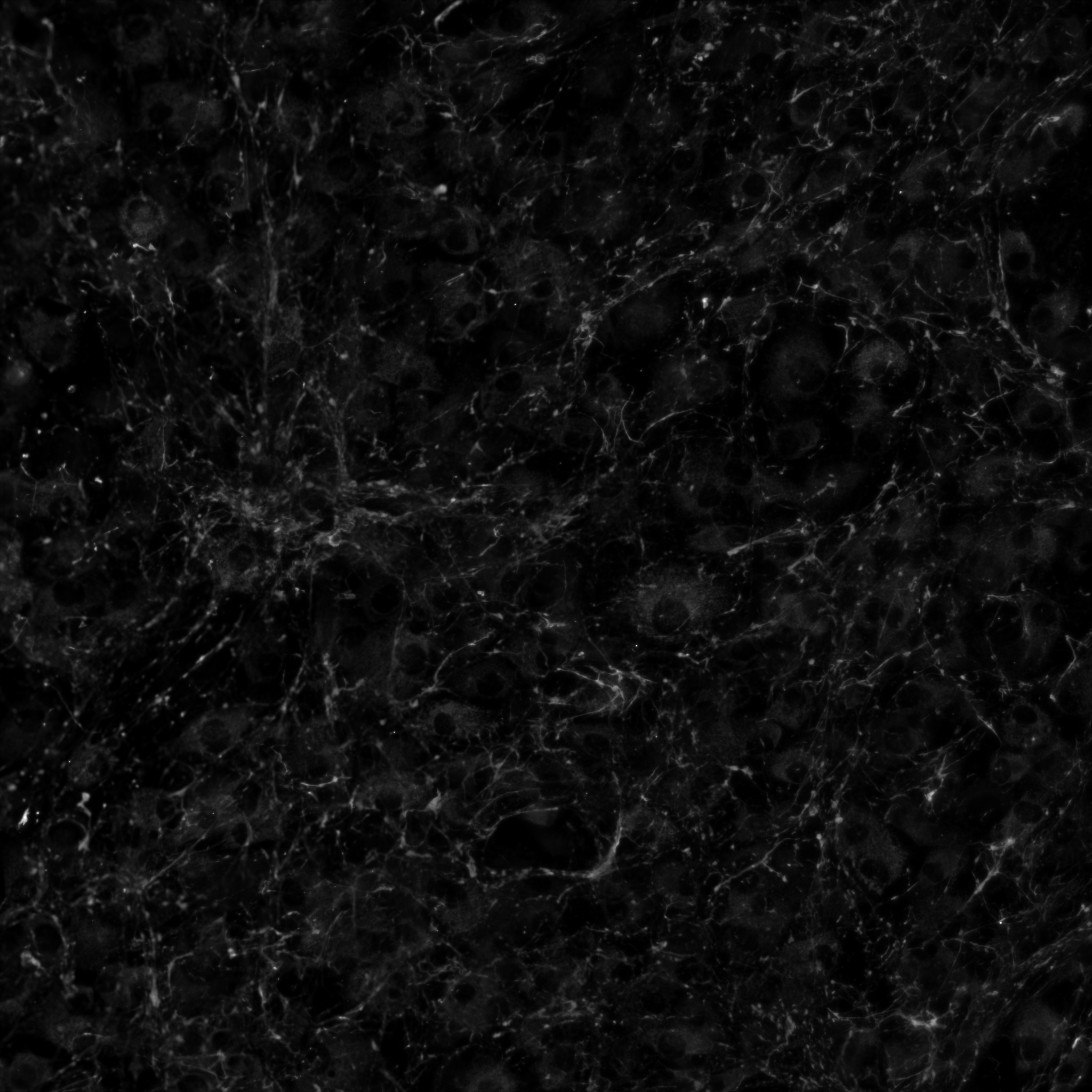

Supplement: Supplementary file 5 — Source data Fig. 3 [file 44319_2026_834_MOESM5_ESM.zip › Figure 3/3F/DMD COL6.jpg]

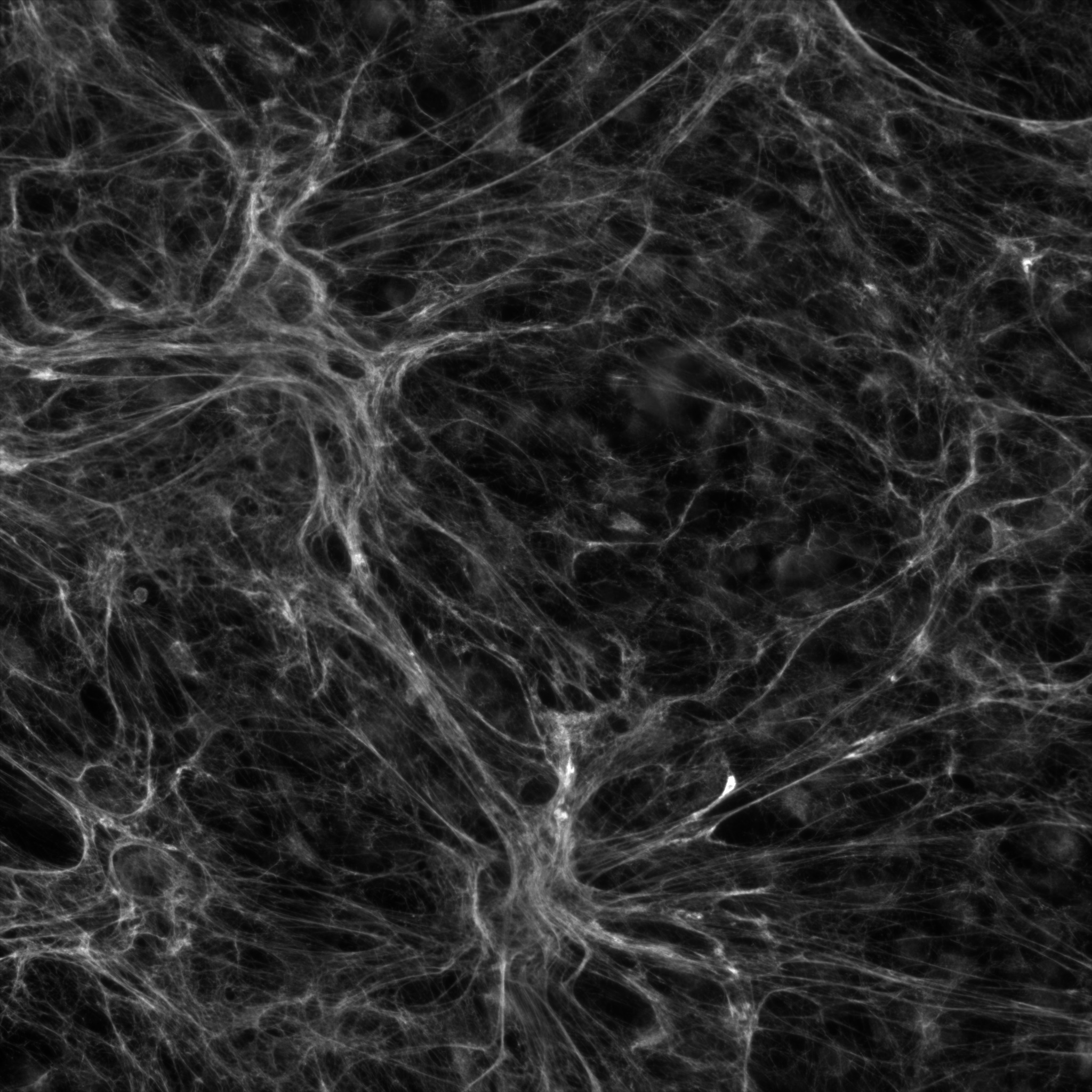

Supplement: Supplementary file 5 — Source data Fig. 3 [file 44319_2026_834_MOESM5_ESM.zip › Figure 3/3F/DMD FN1.jpg]

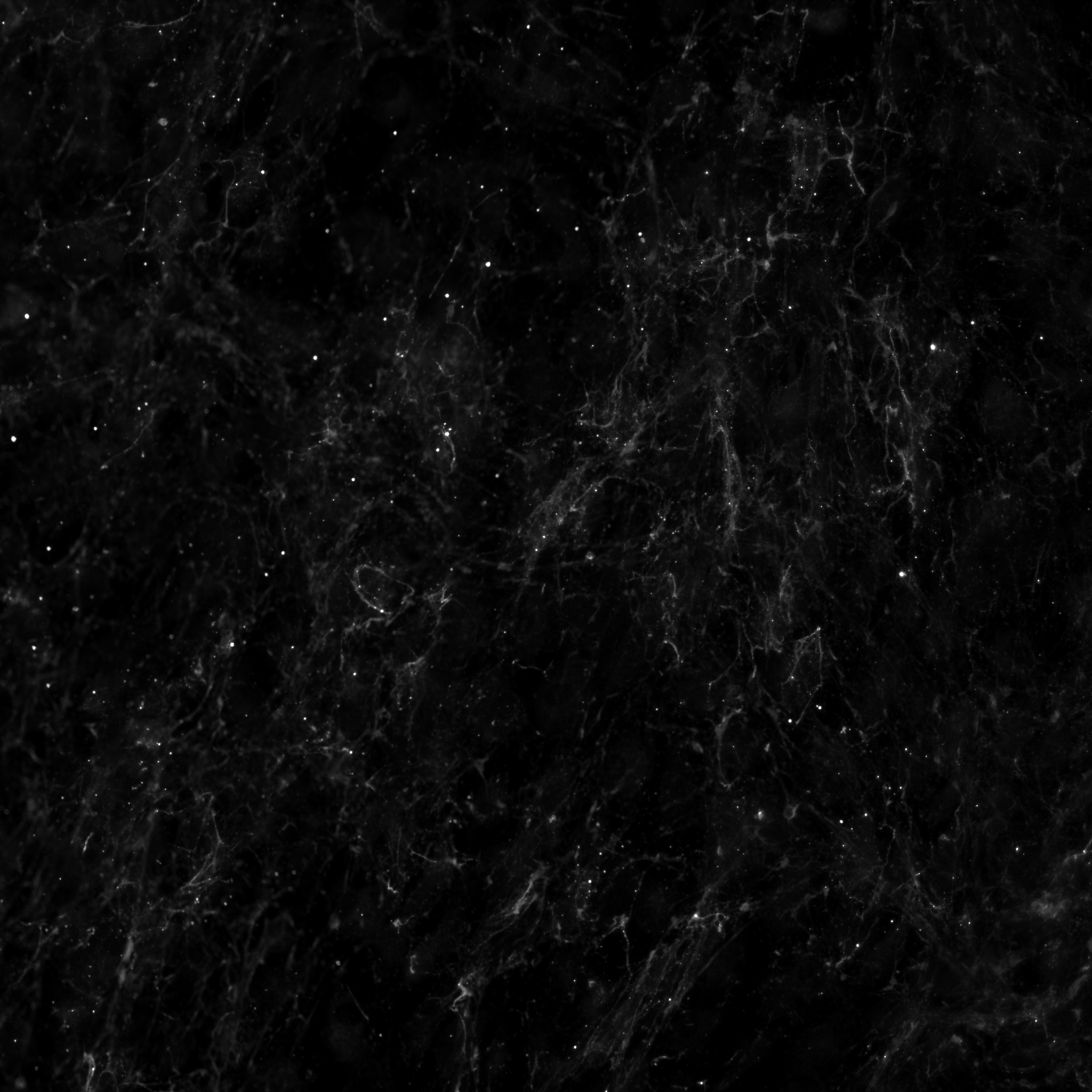

Supplement: Supplementary file 5 — Source data Fig. 3 [file 44319_2026_834_MOESM5_ESM.zip › Figure 3/3F/DMD TNXB.jpg]

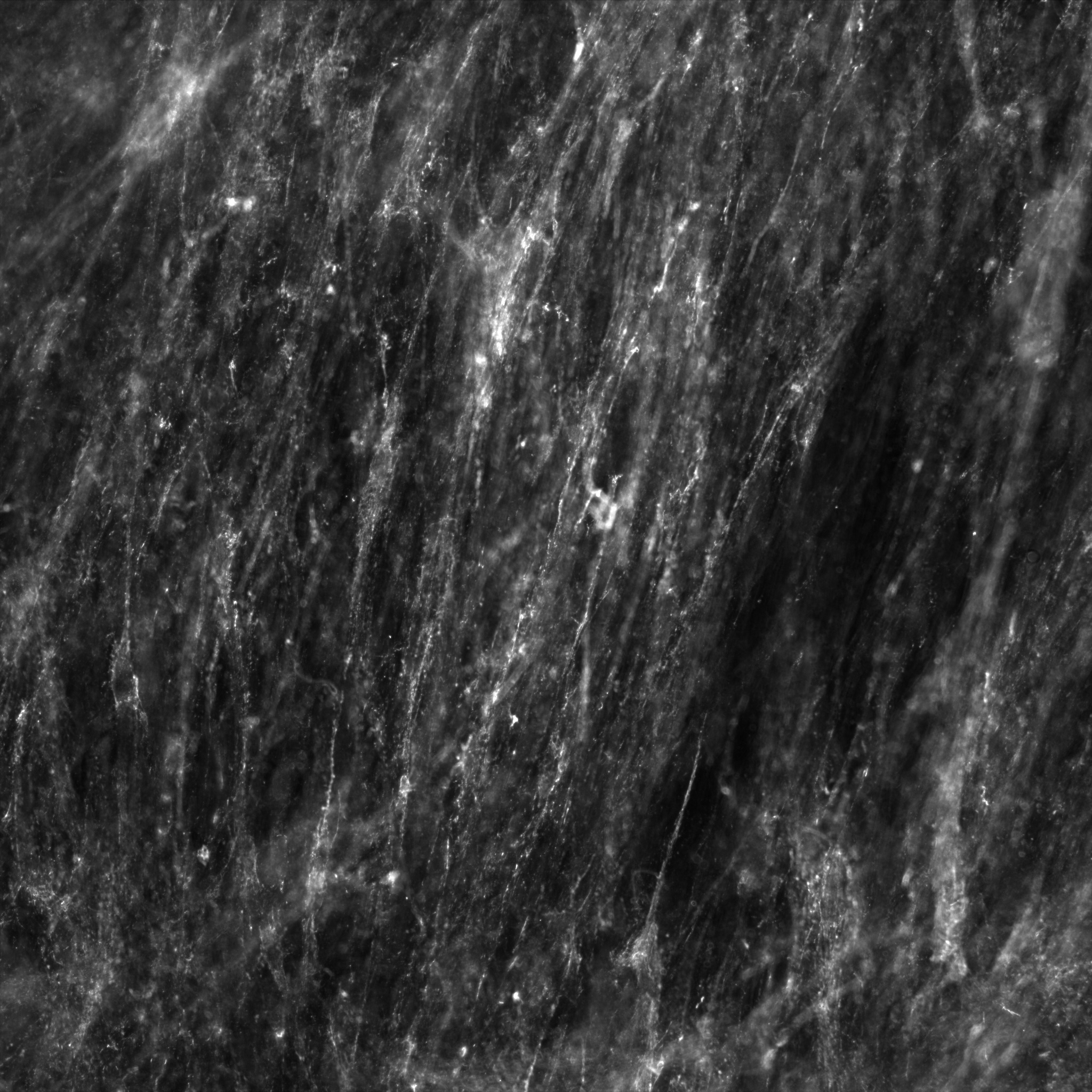

Supplement: Supplementary file 5 — Source data Fig. 3 [file 44319_2026_834_MOESM5_ESM.zip › Figure 3/3F/IBM COL4.jpg]

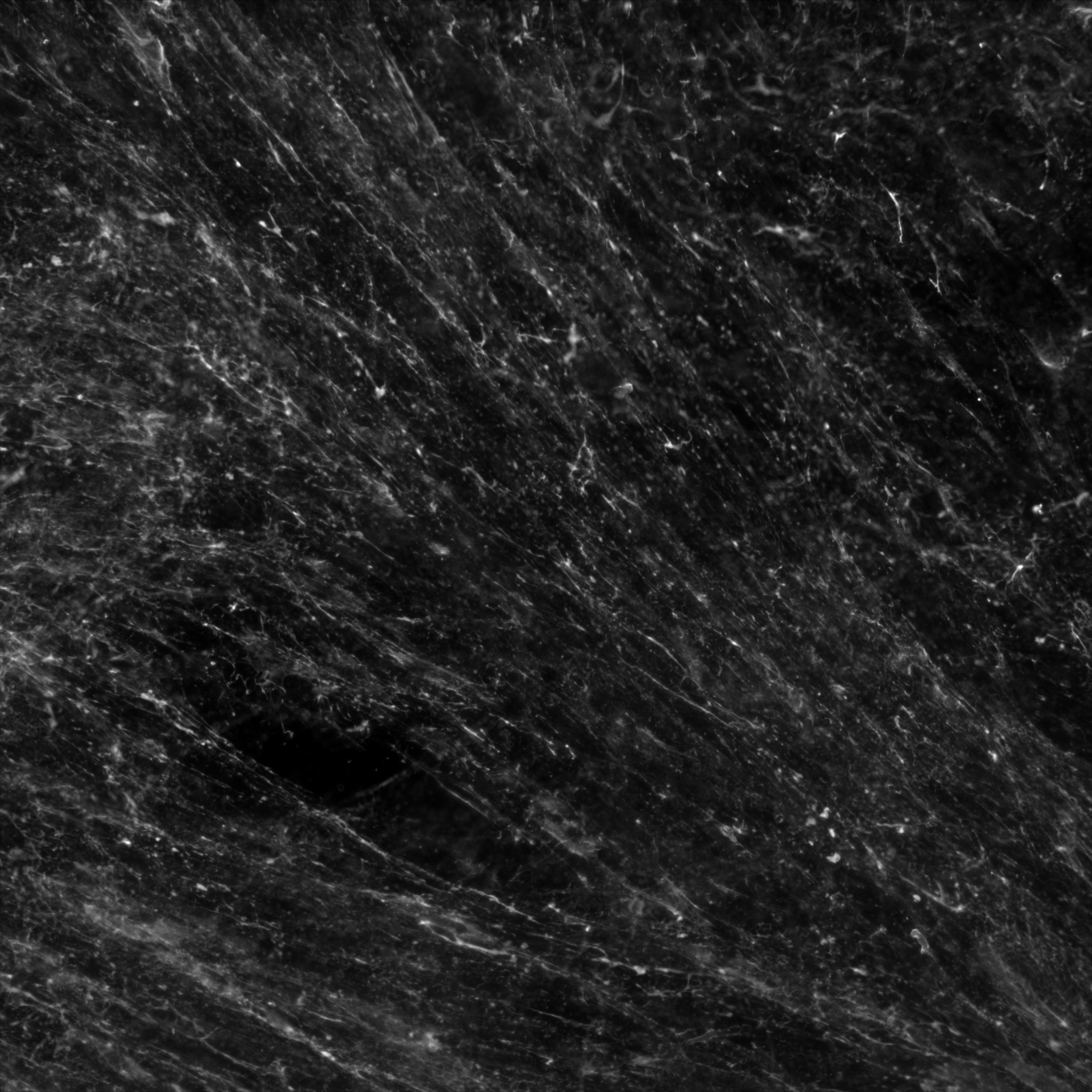

Supplement: Supplementary file 5 — Source data Fig. 3 [file 44319_2026_834_MOESM5_ESM.zip › Figure 3/3F/IBM COL6.jpg]

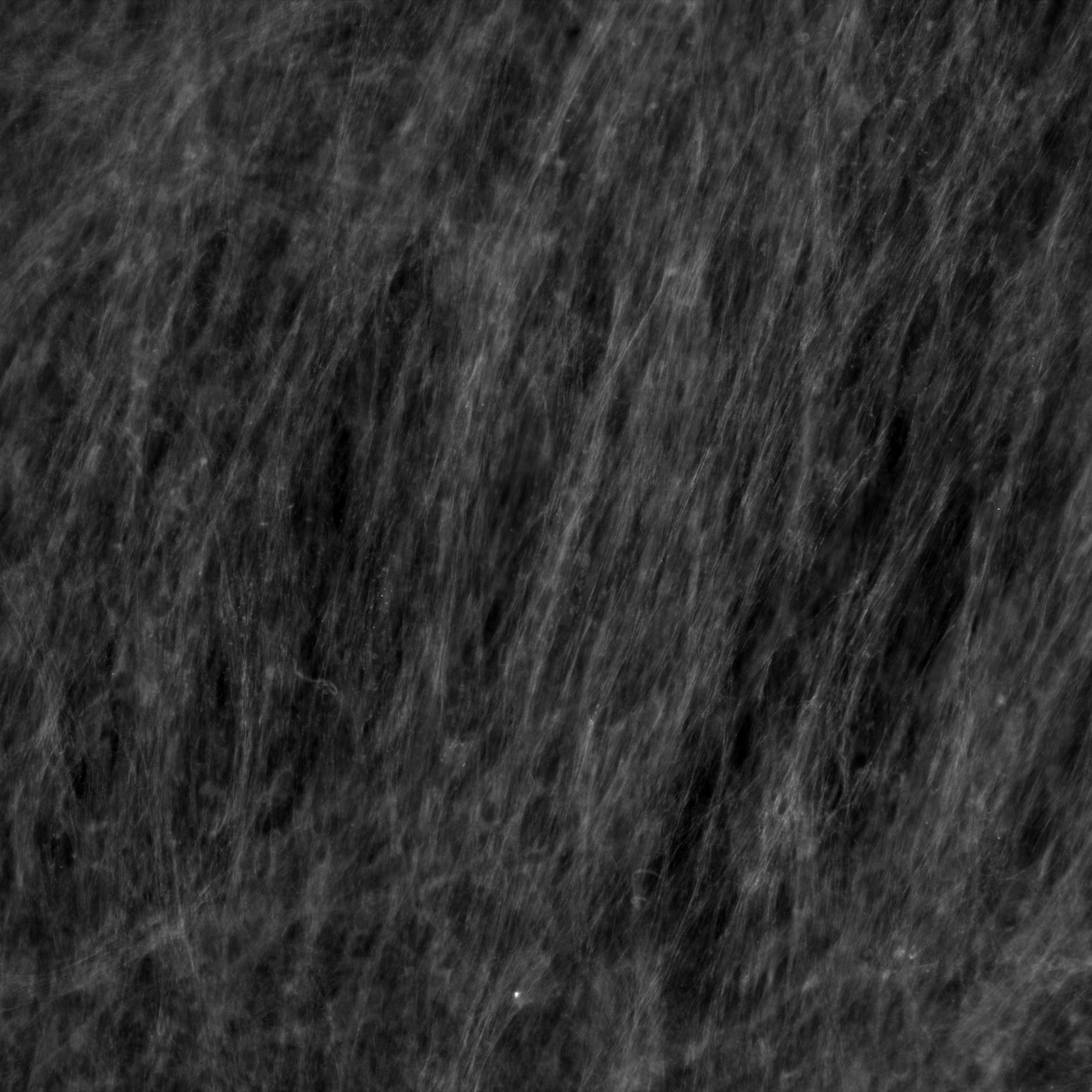

Supplement: Supplementary file 5 — Source data Fig. 3 [file 44319_2026_834_MOESM5_ESM.zip › Figure 3/3F/IBM FN1.jpg]

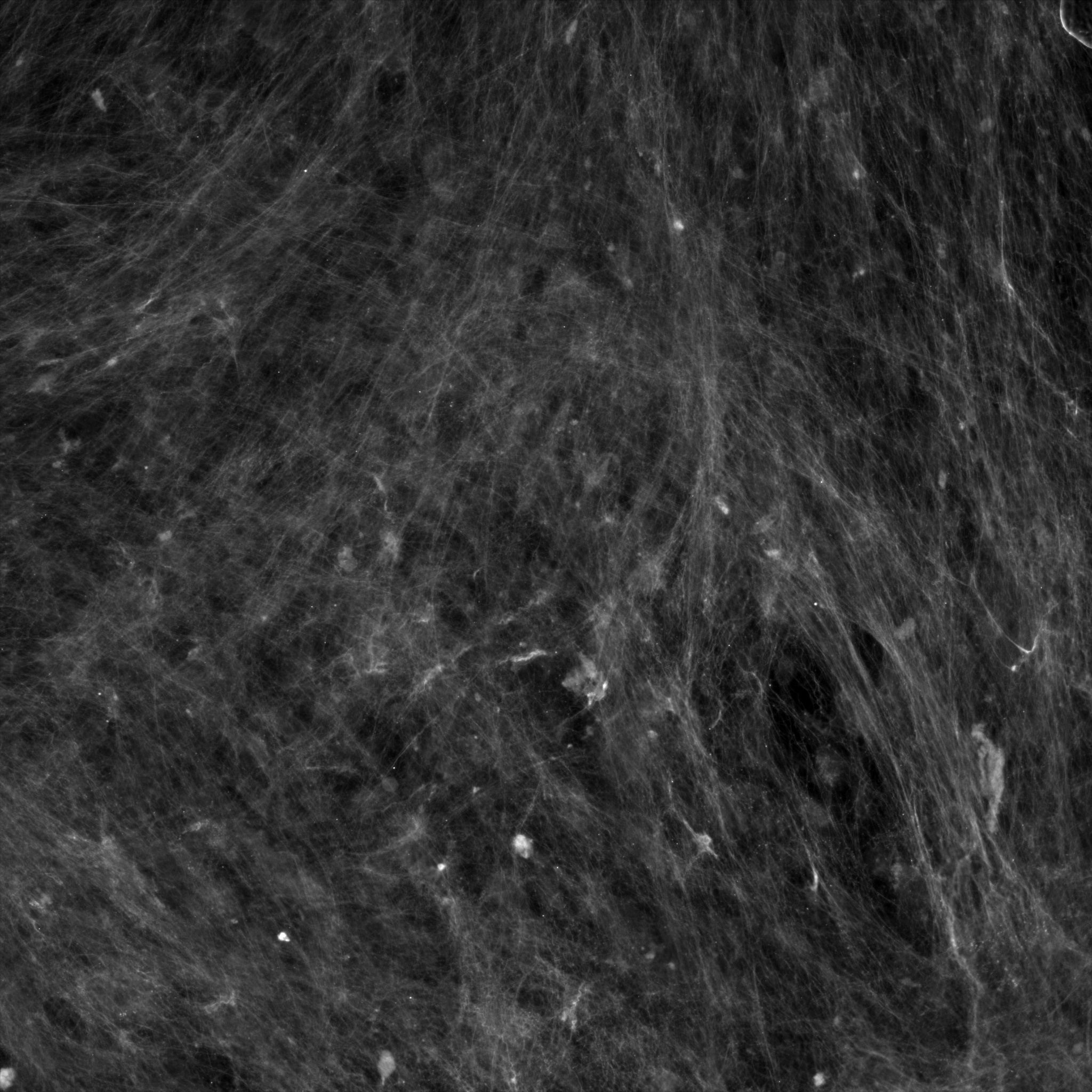

Supplement: Supplementary file 5 — Source data Fig. 3 [file 44319_2026_834_MOESM5_ESM.zip › Figure 3/3F/IBM TNXB.jpg]

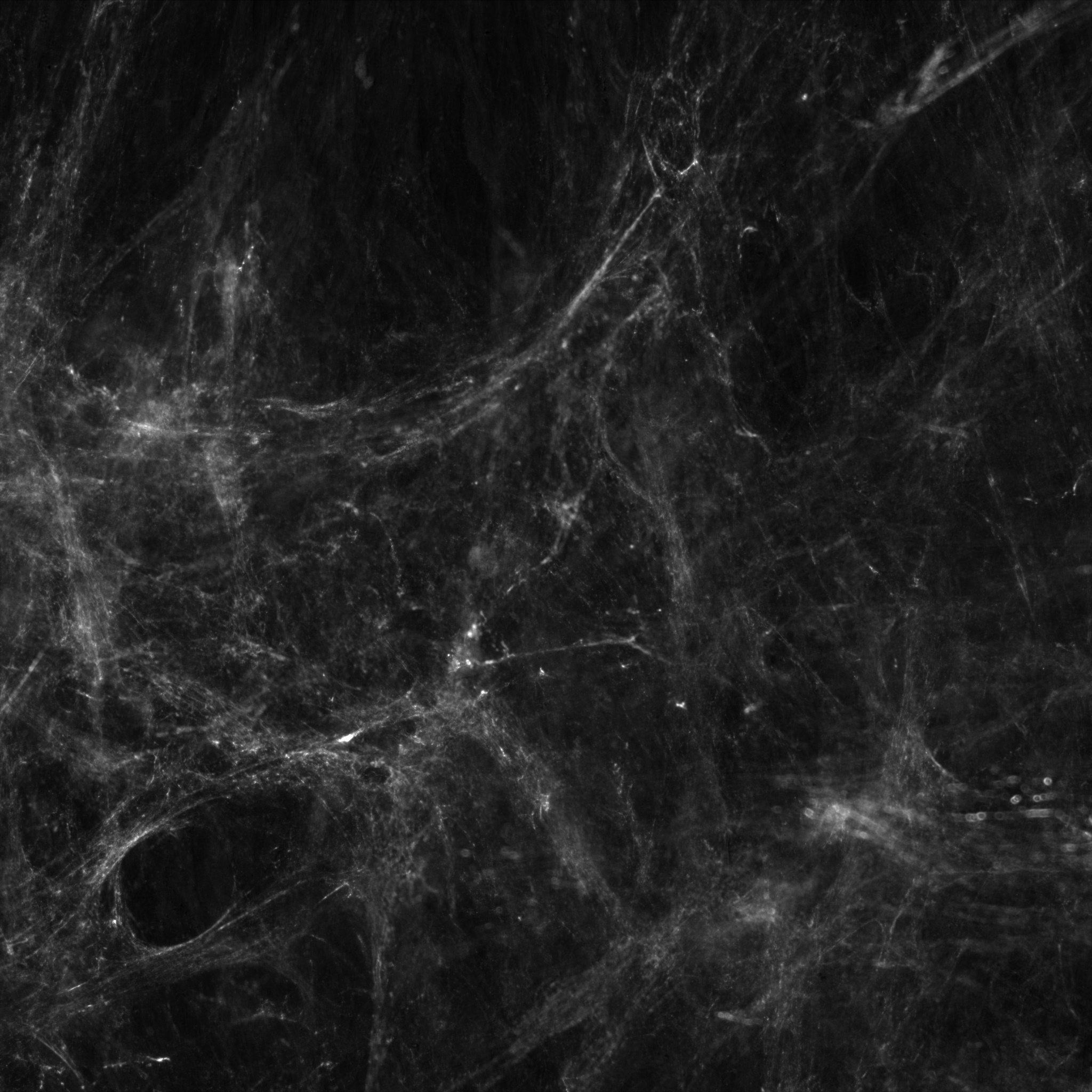

Supplement: Supplementary file 5 — Source data Fig. 3 [file 44319_2026_834_MOESM5_ESM.zip › Figure 3/3F/OPMD COL4.jpg]

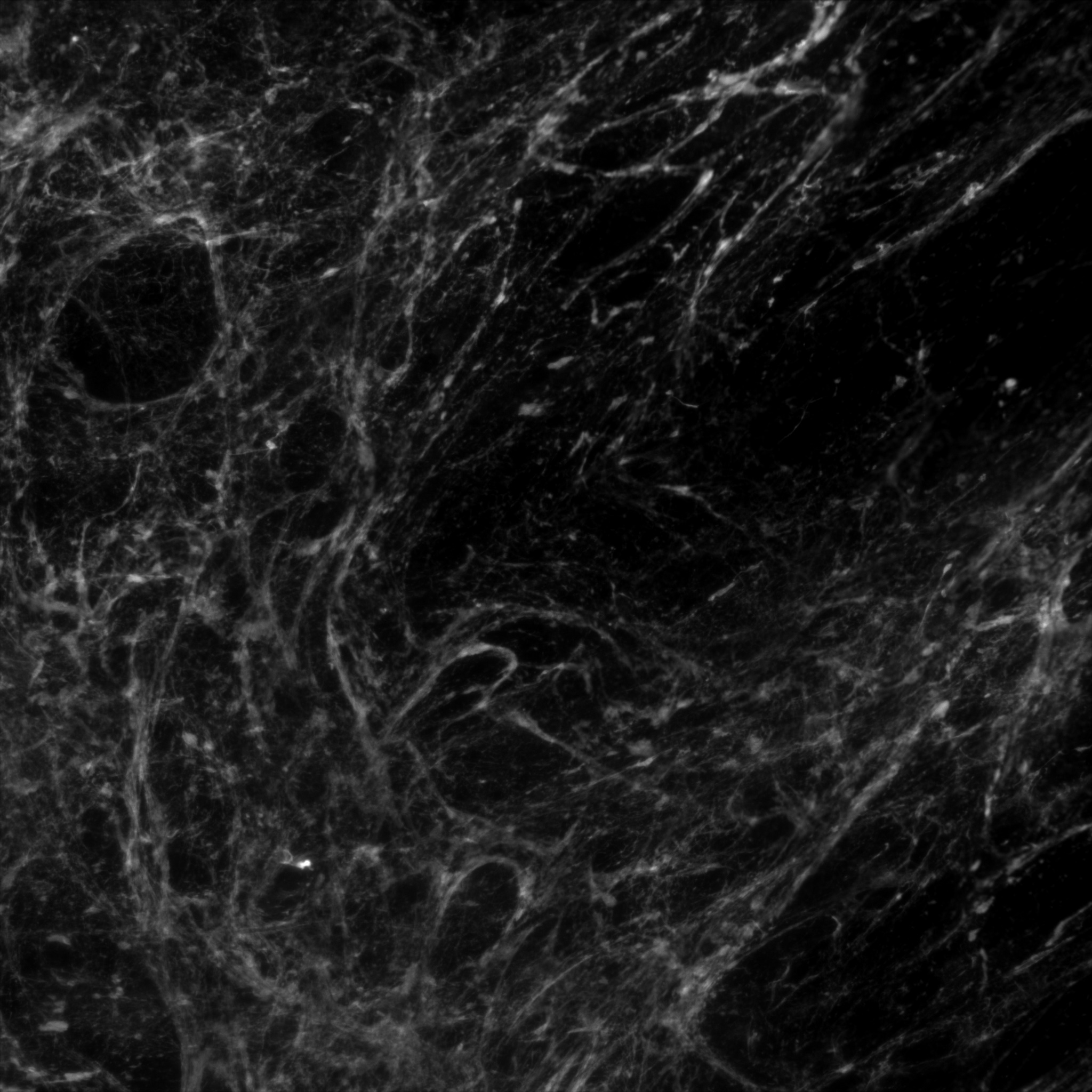

Supplement: Supplementary file 5 — Source data Fig. 3 [file 44319_2026_834_MOESM5_ESM.zip › Figure 3/3F/OPMD COL6.jpg]

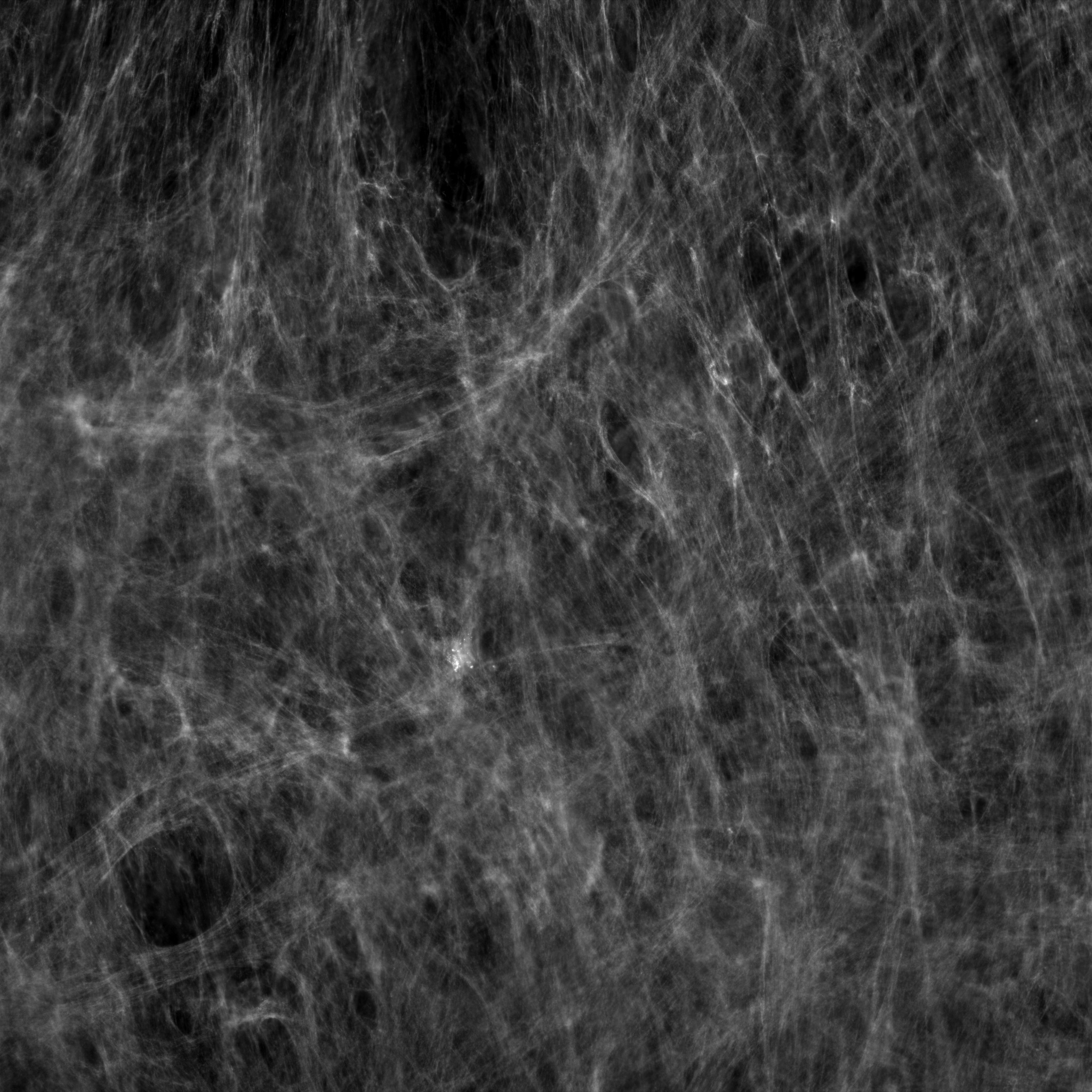

Supplement: Supplementary file 5 — Source data Fig. 3 [file 44319_2026_834_MOESM5_ESM.zip › Figure 3/3F/OPMD FN1 .jpg]

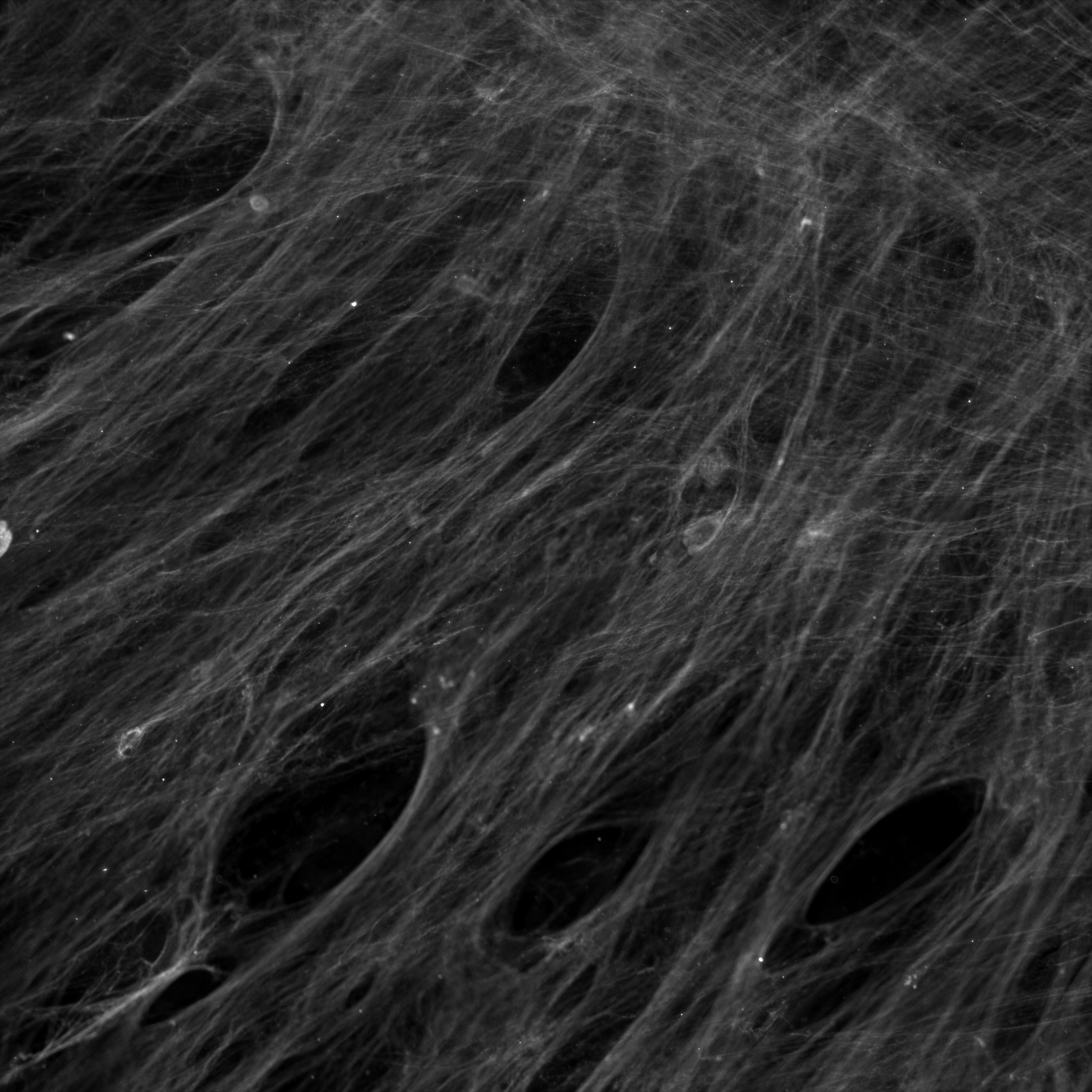

Supplement: Supplementary file 5 — Source data Fig. 3 [file 44319_2026_834_MOESM5_ESM.zip › Figure 3/3F/OPMD TNXB.jpg]

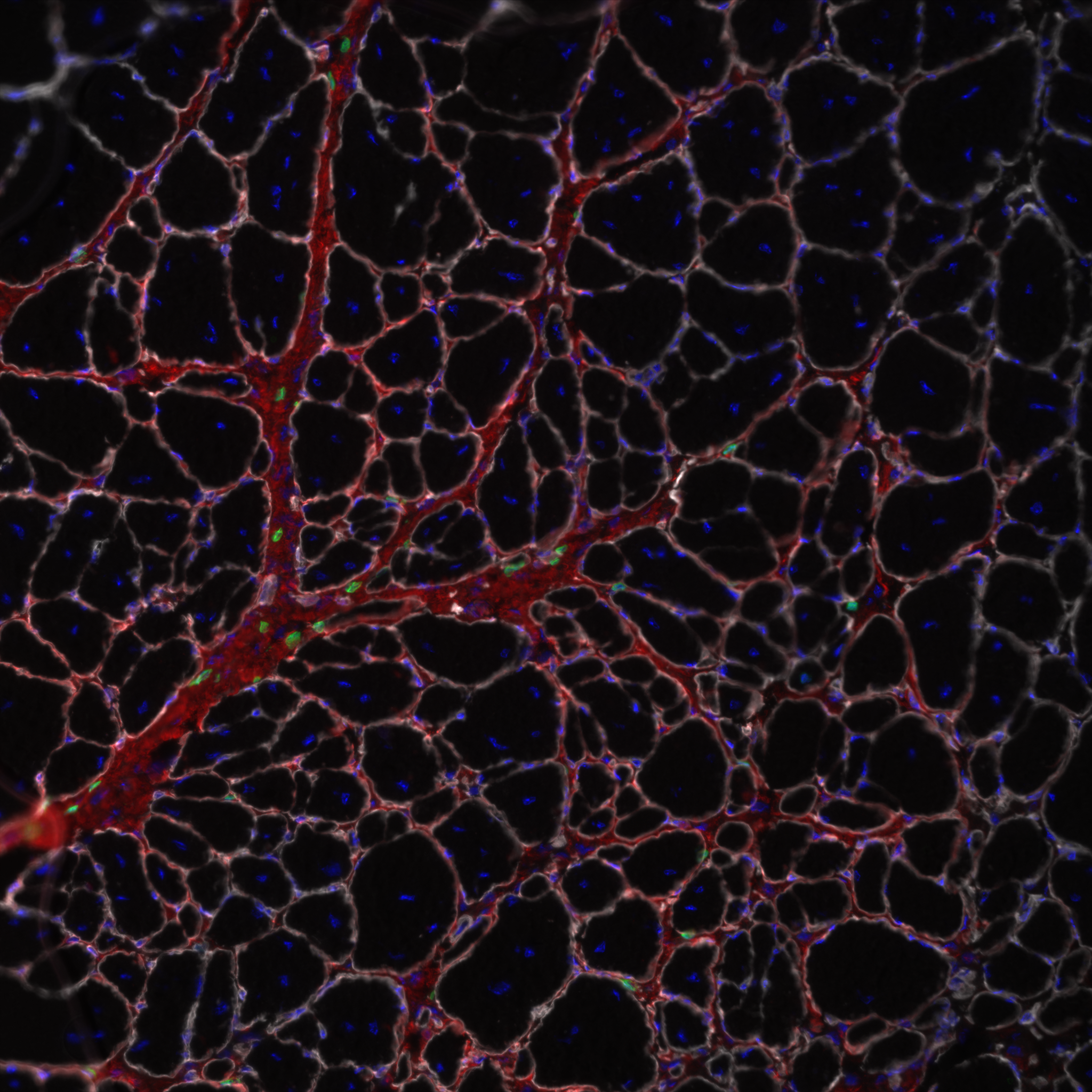

Supplement: Supplementary file 6 — Source data Fig. 4 [file 44319_2026_834_MOESM6_ESM.zip › Figure 4/4B/CTL hCOL6 hlaminAC.tif]

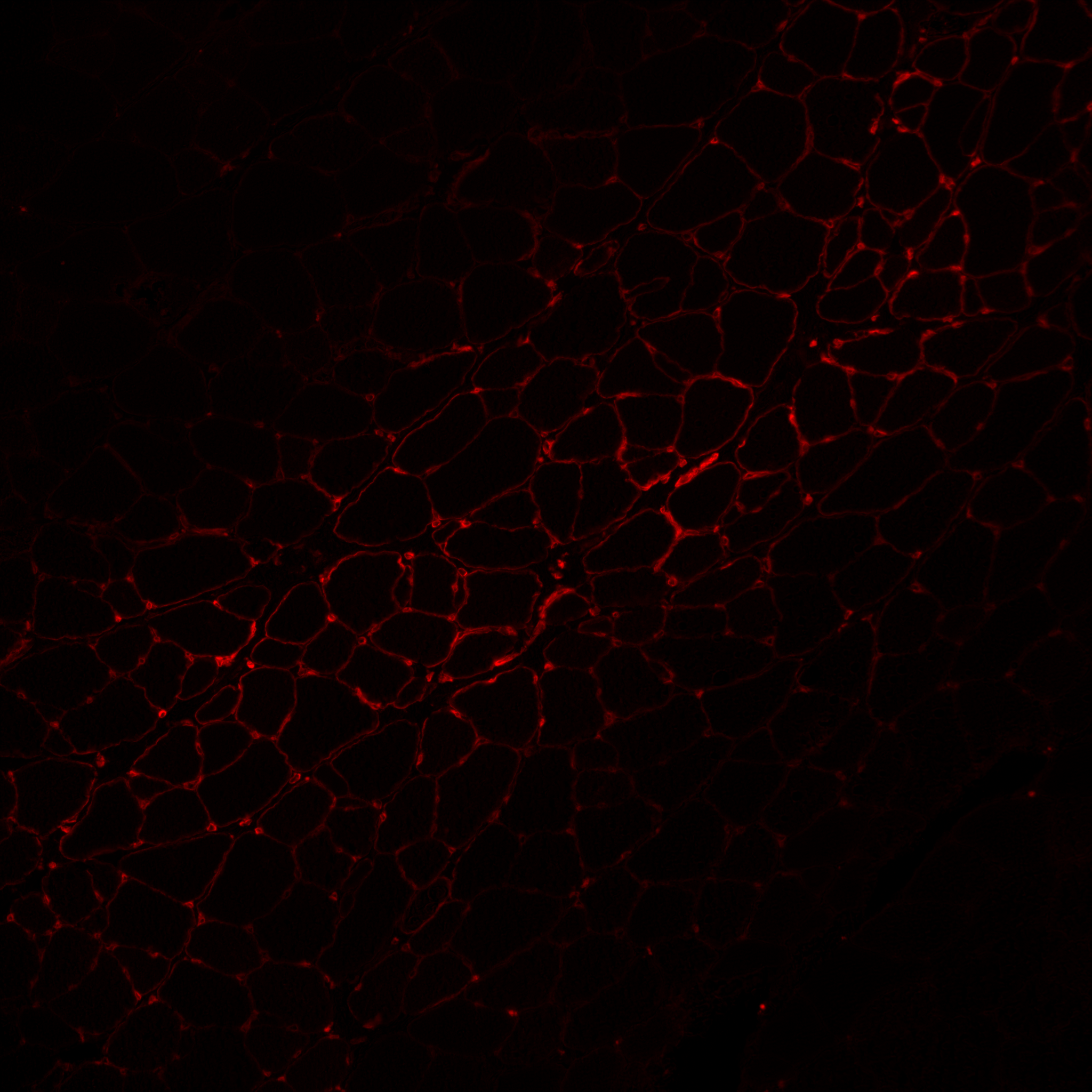

Supplement: Supplementary file 6 — Source data Fig. 4 [file 44319_2026_834_MOESM6_ESM.zip › Figure 4/4B/CTL hLAMC1.tif]

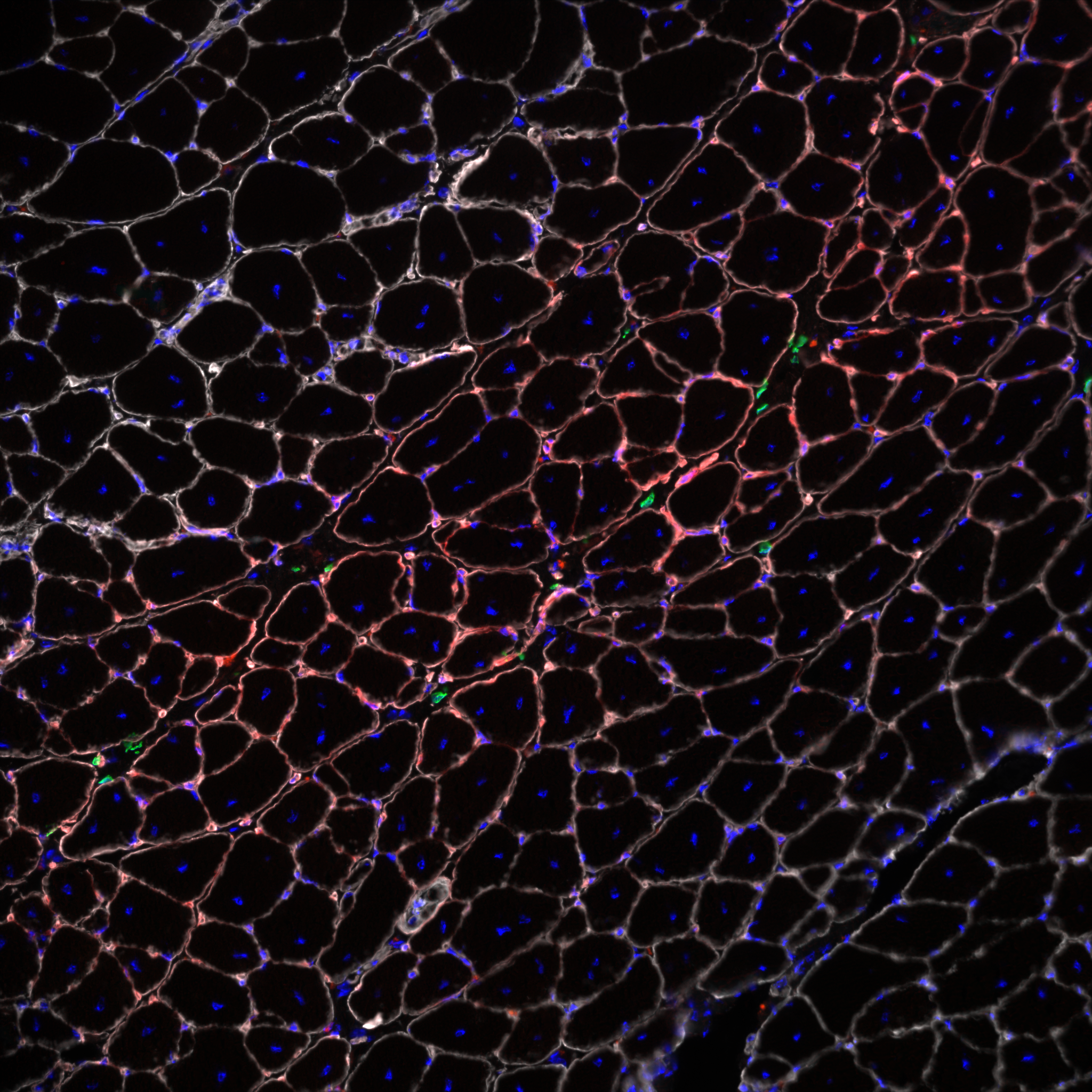

Supplement: Supplementary file 6 — Source data Fig. 4 [file 44319_2026_834_MOESM6_ESM.zip › Figure 4/4B/CTL hlaminAC hLAMC1 555.tif]

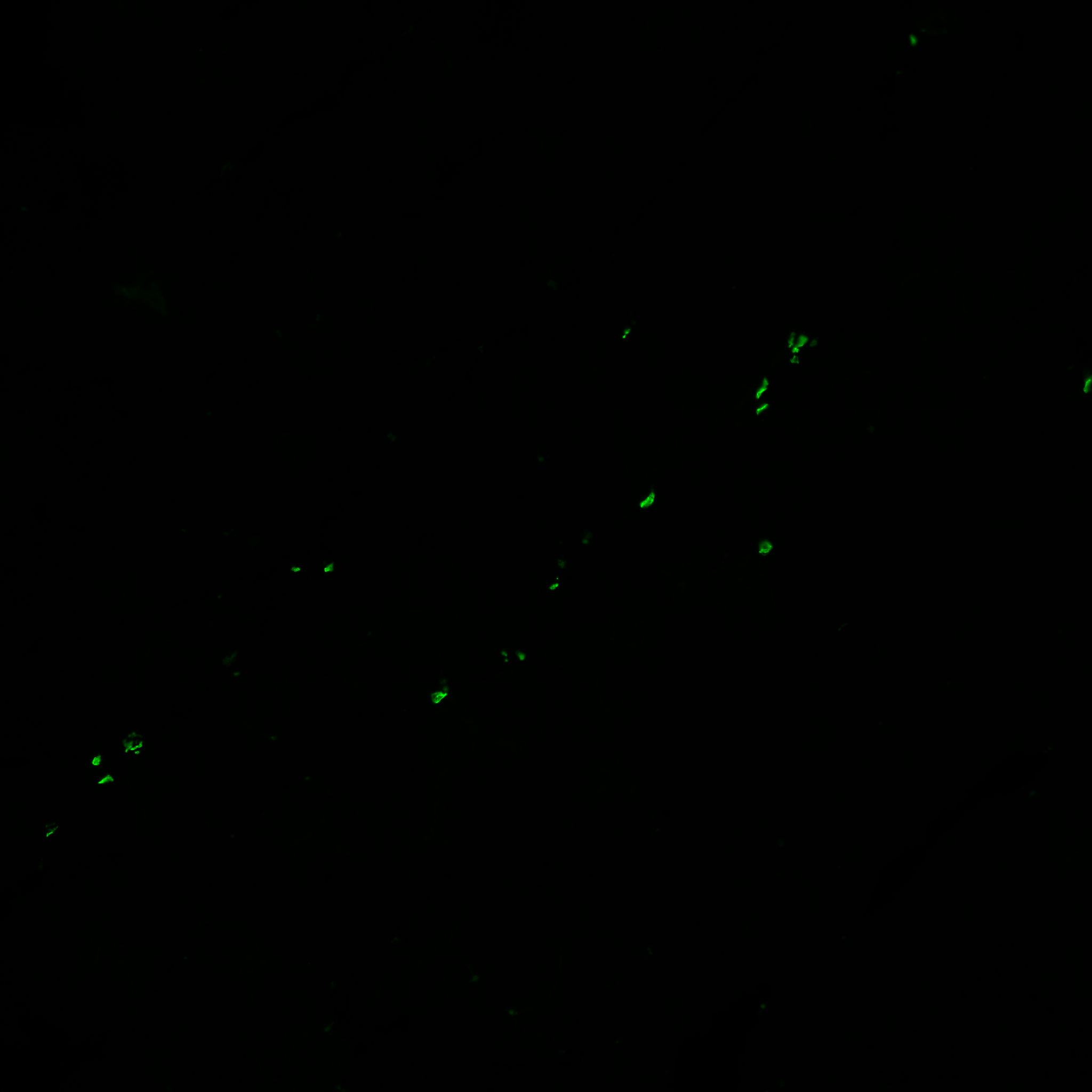

Supplement: Supplementary file 6 — Source data Fig. 4 [file 44319_2026_834_MOESM6_ESM.zip › Figure 4/4B/CTL hlaminAC.tif]

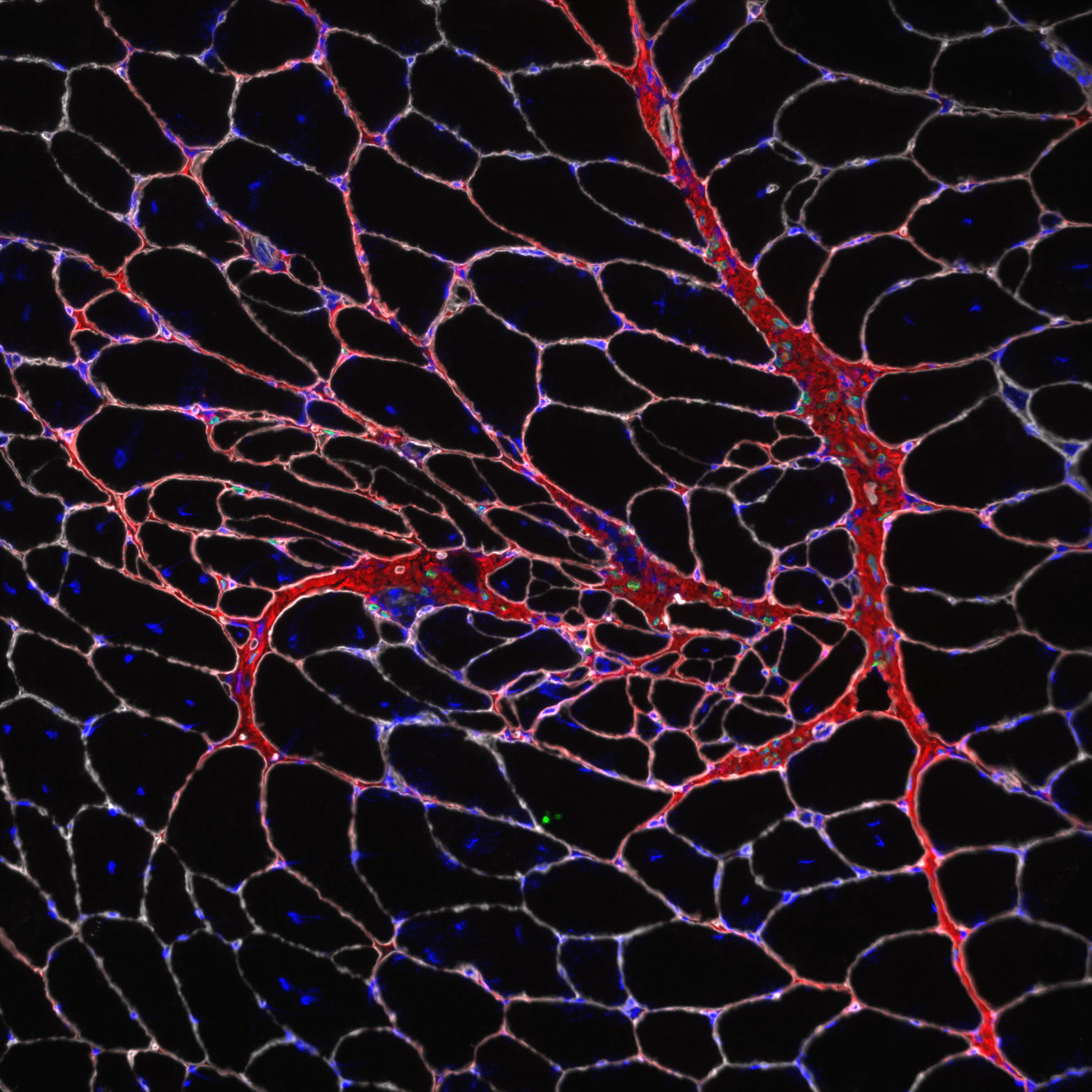

Supplement: Supplementary file 6 — Source data Fig. 4 [file 44319_2026_834_MOESM6_ESM.zip › Figure 4/4B/DMD hCOL6 hlaminAC.tif]

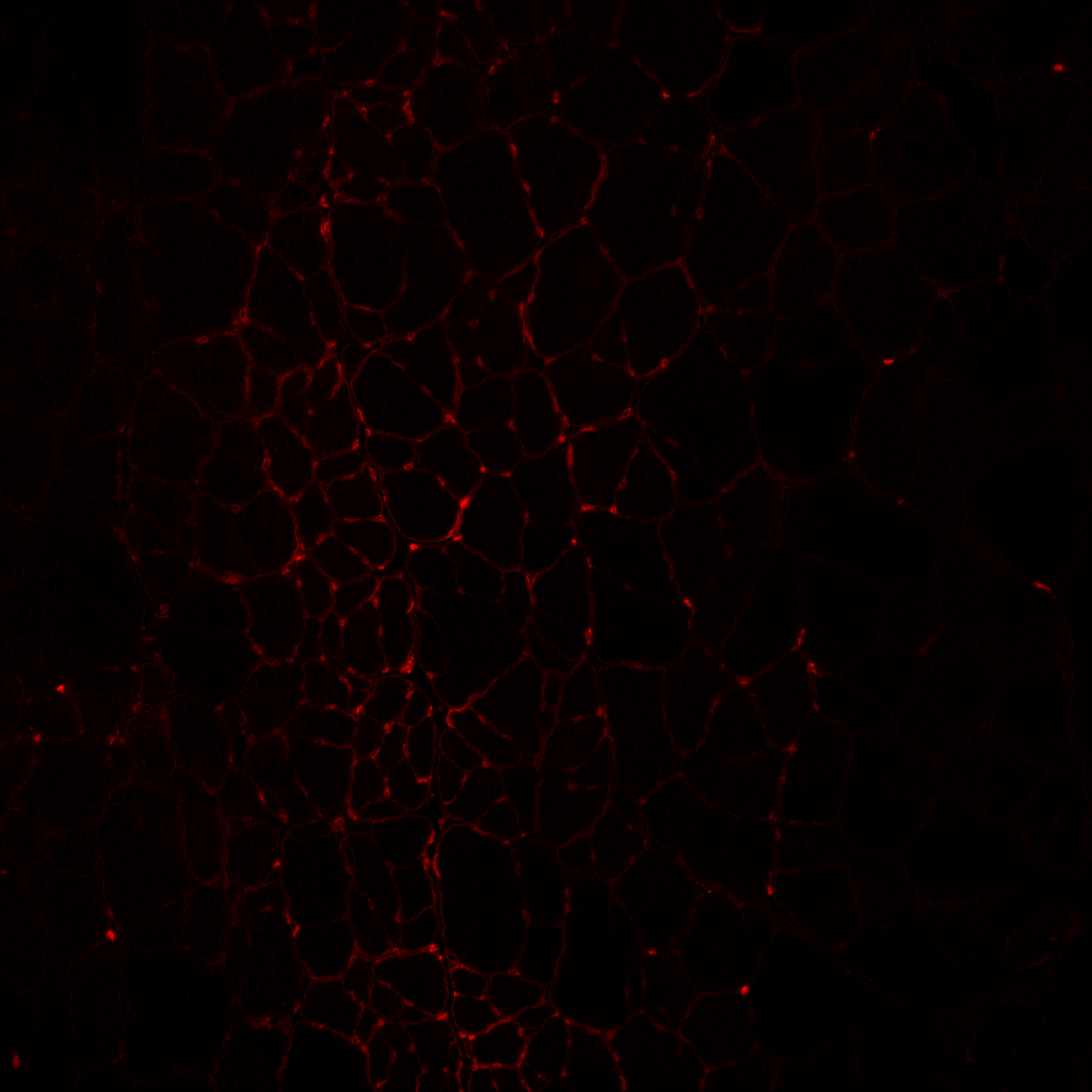

Supplement: Supplementary file 6 — Source data Fig. 4 [file 44319_2026_834_MOESM6_ESM.zip › Figure 4/4B/DMD hLAMC1.tif]

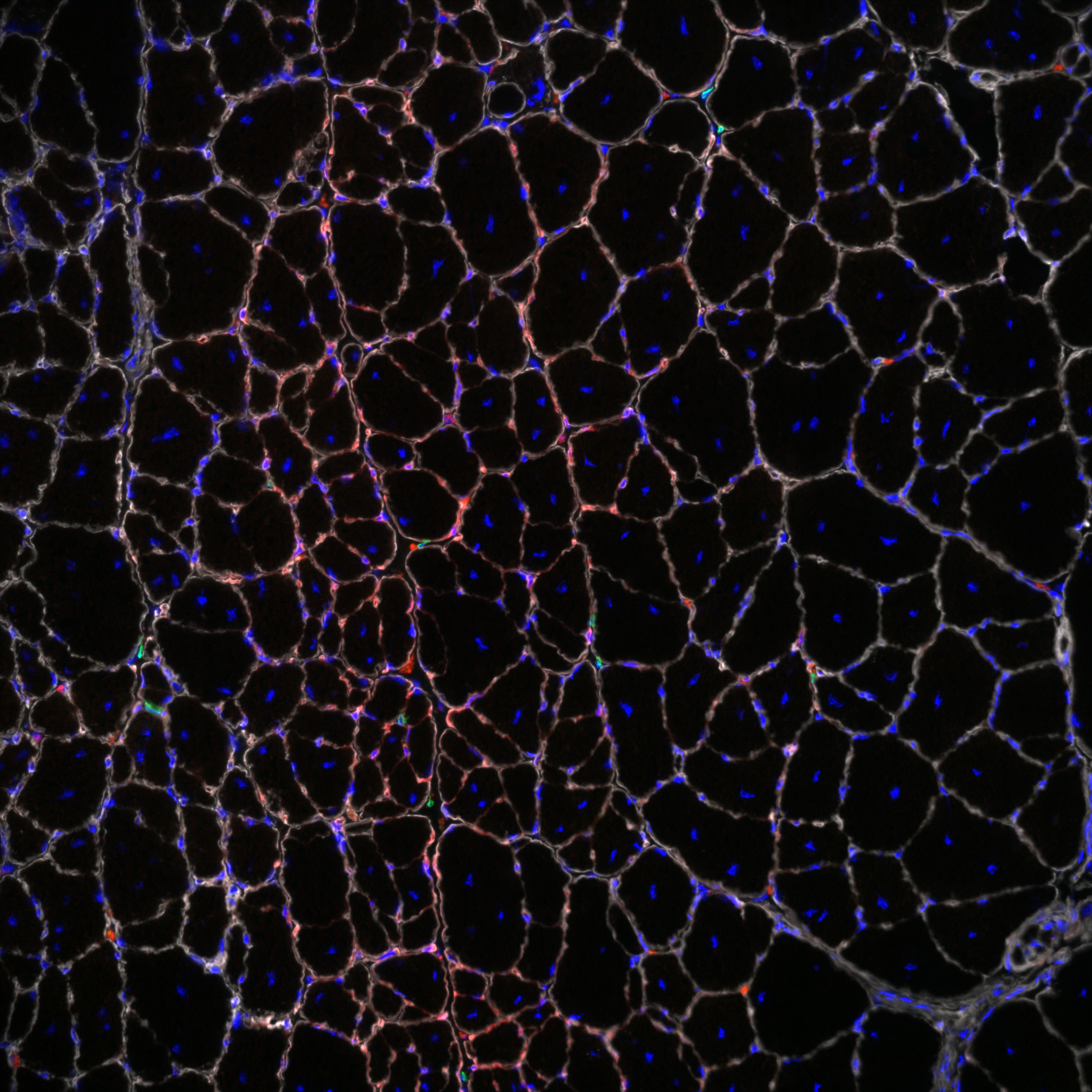

Supplement: Supplementary file 6 — Source data Fig. 4 [file 44319_2026_834_MOESM6_ESM.zip › Figure 4/4B/DMD hlaminAC hLAMC1 .tif]

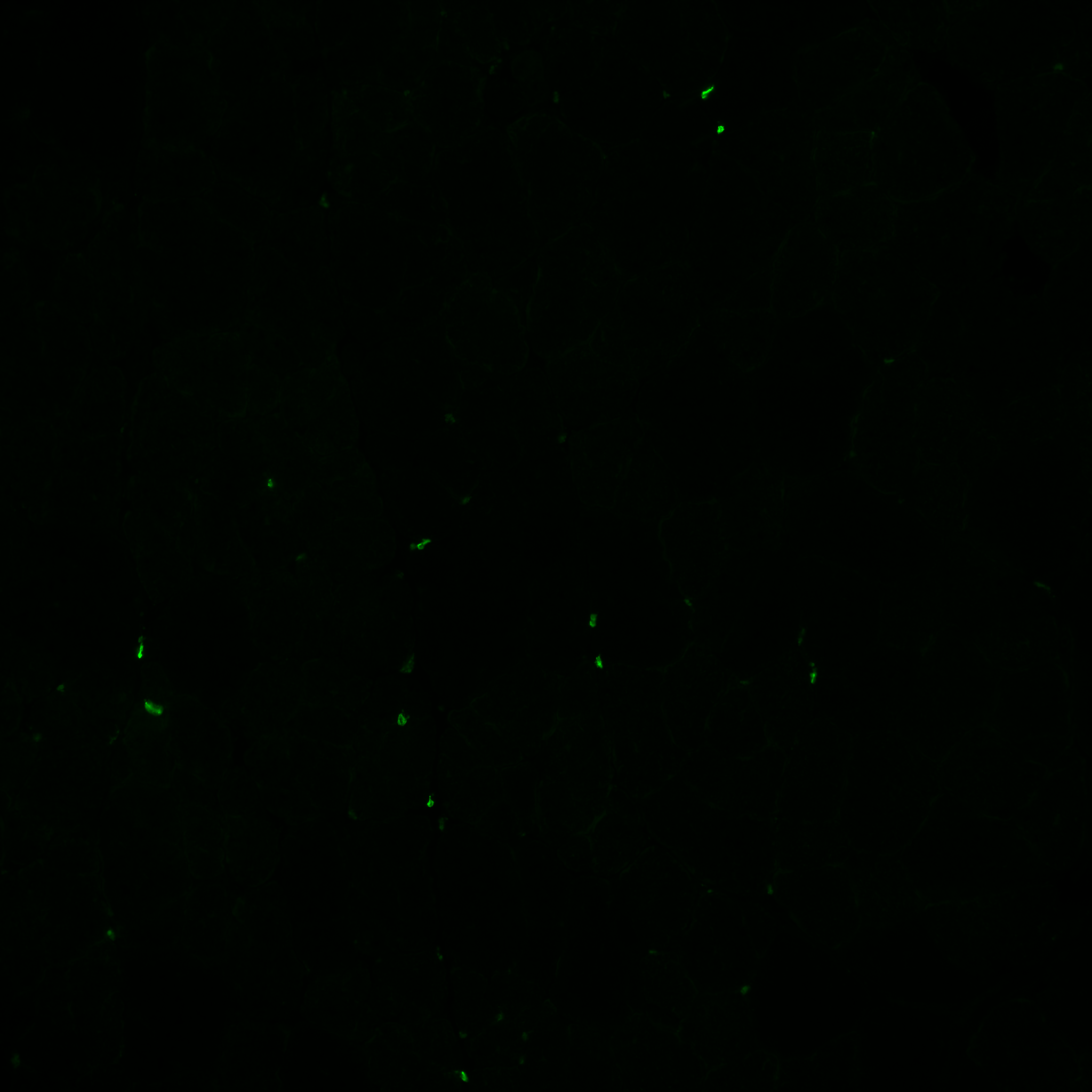

Supplement: Supplementary file 6 — Source data Fig. 4 [file 44319_2026_834_MOESM6_ESM.zip › Figure 4/4B/DMD hlaminAC.tif]

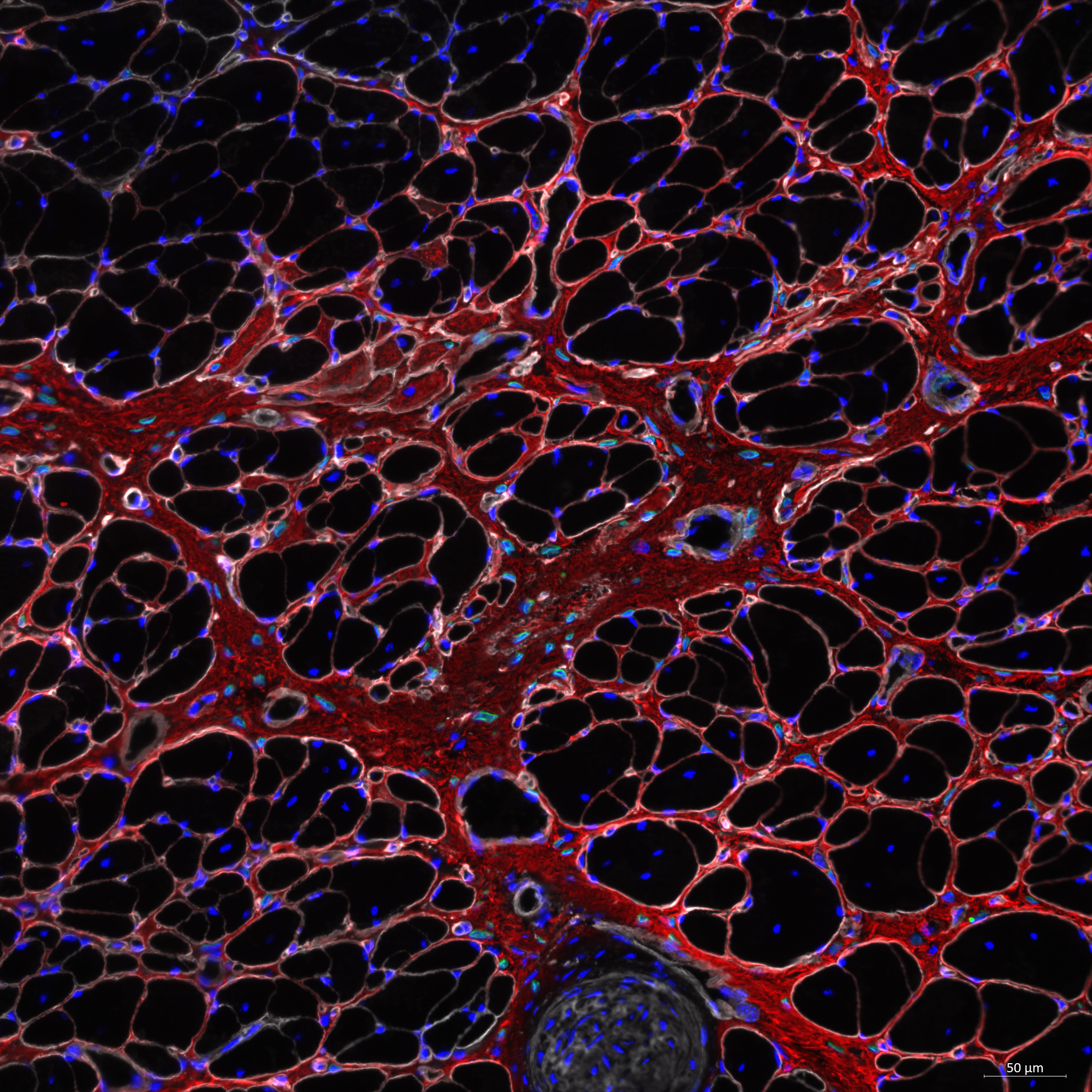

Supplement: Supplementary file 6 — Source data Fig. 4 [file 44319_2026_834_MOESM6_ESM.zip › Figure 4/4B/OPMD hCOL6 hlaminAC.tif]

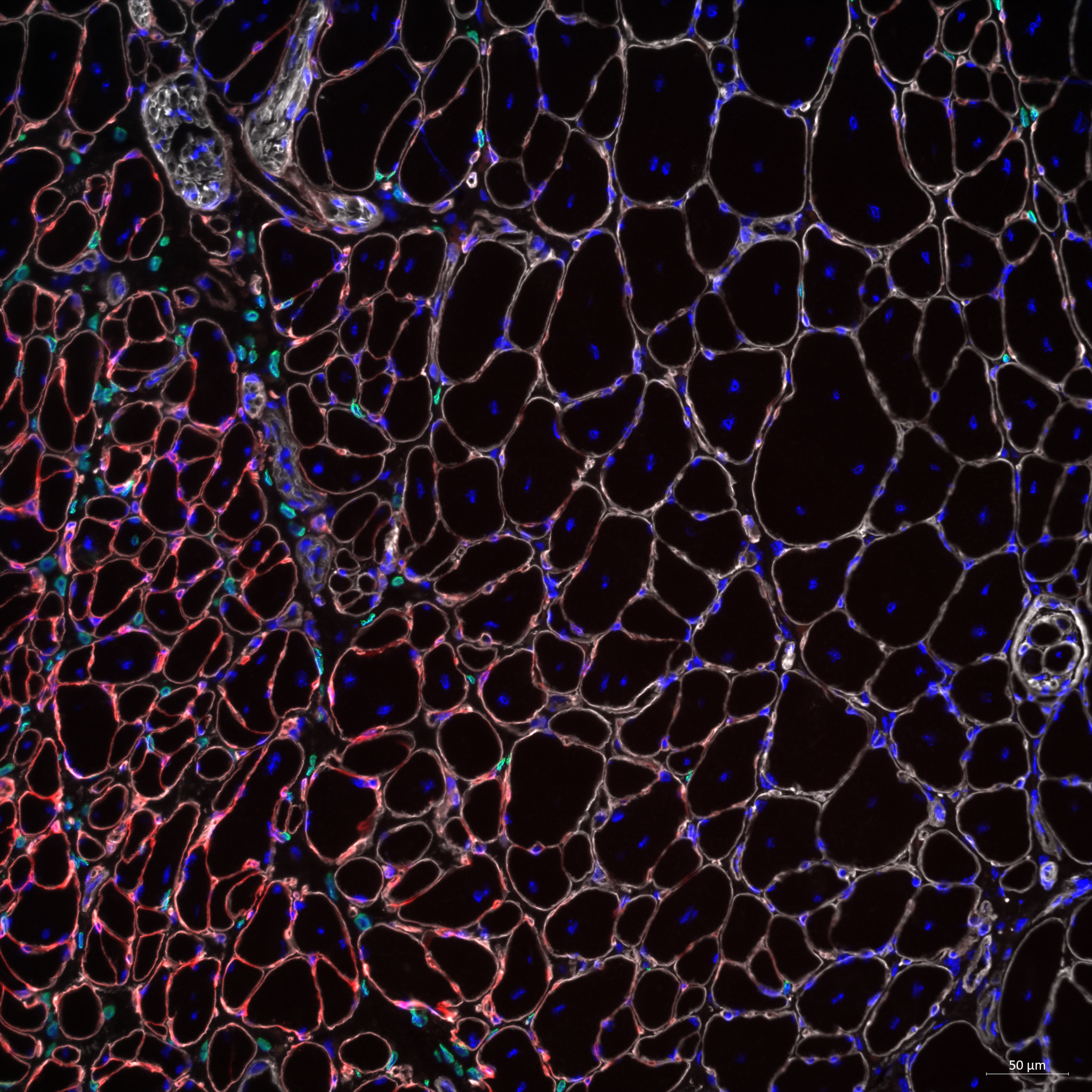

Supplement: Supplementary file 6 — Source data Fig. 4 [file 44319_2026_834_MOESM6_ESM.zip › Figure 4/4B/OPMD hLAMC1 hlaminAC.tif]

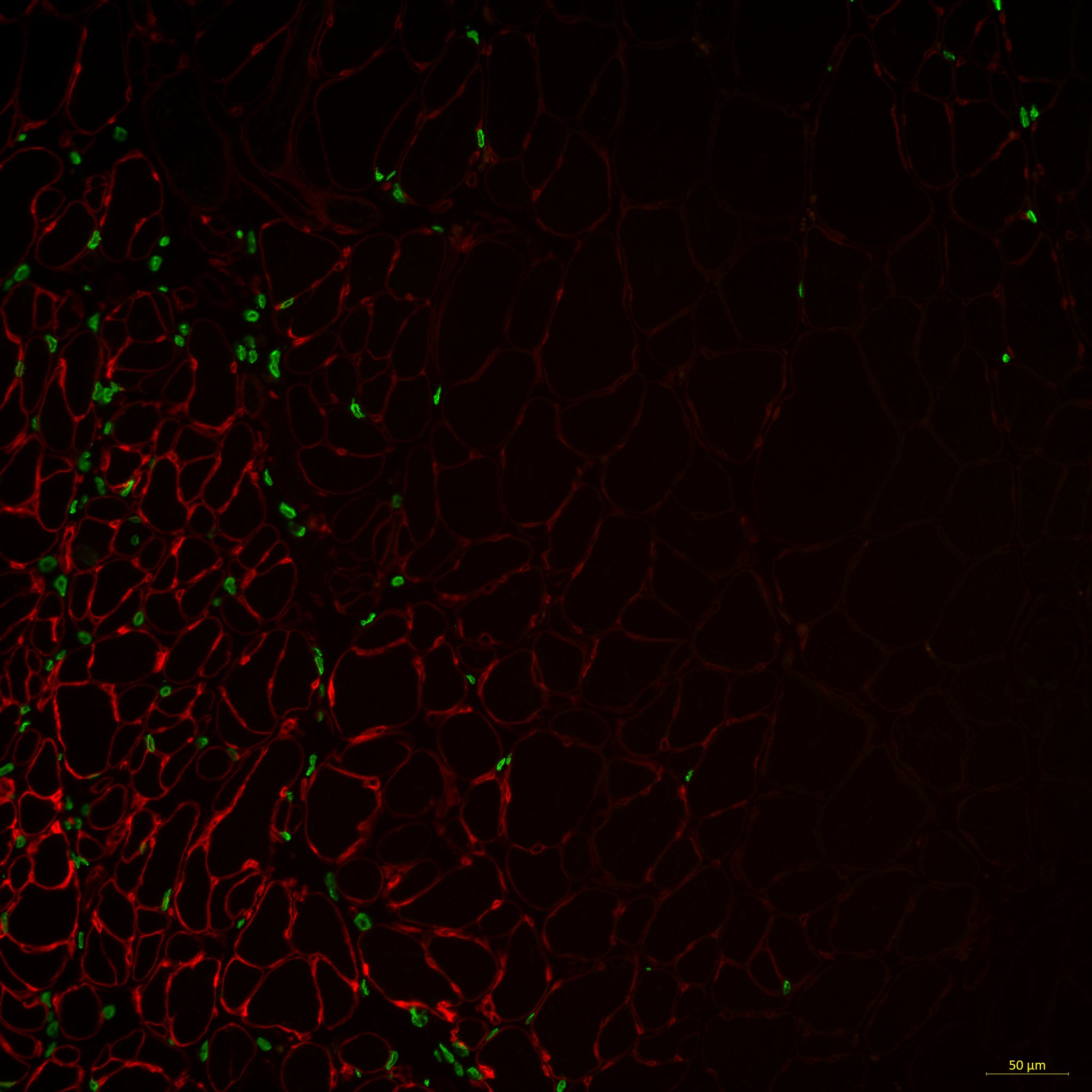

Supplement: Supplementary file 6 — Source data Fig. 4 [file 44319_2026_834_MOESM6_ESM.zip › Figure 4/4B/OPMD hLAMC1.jpg]

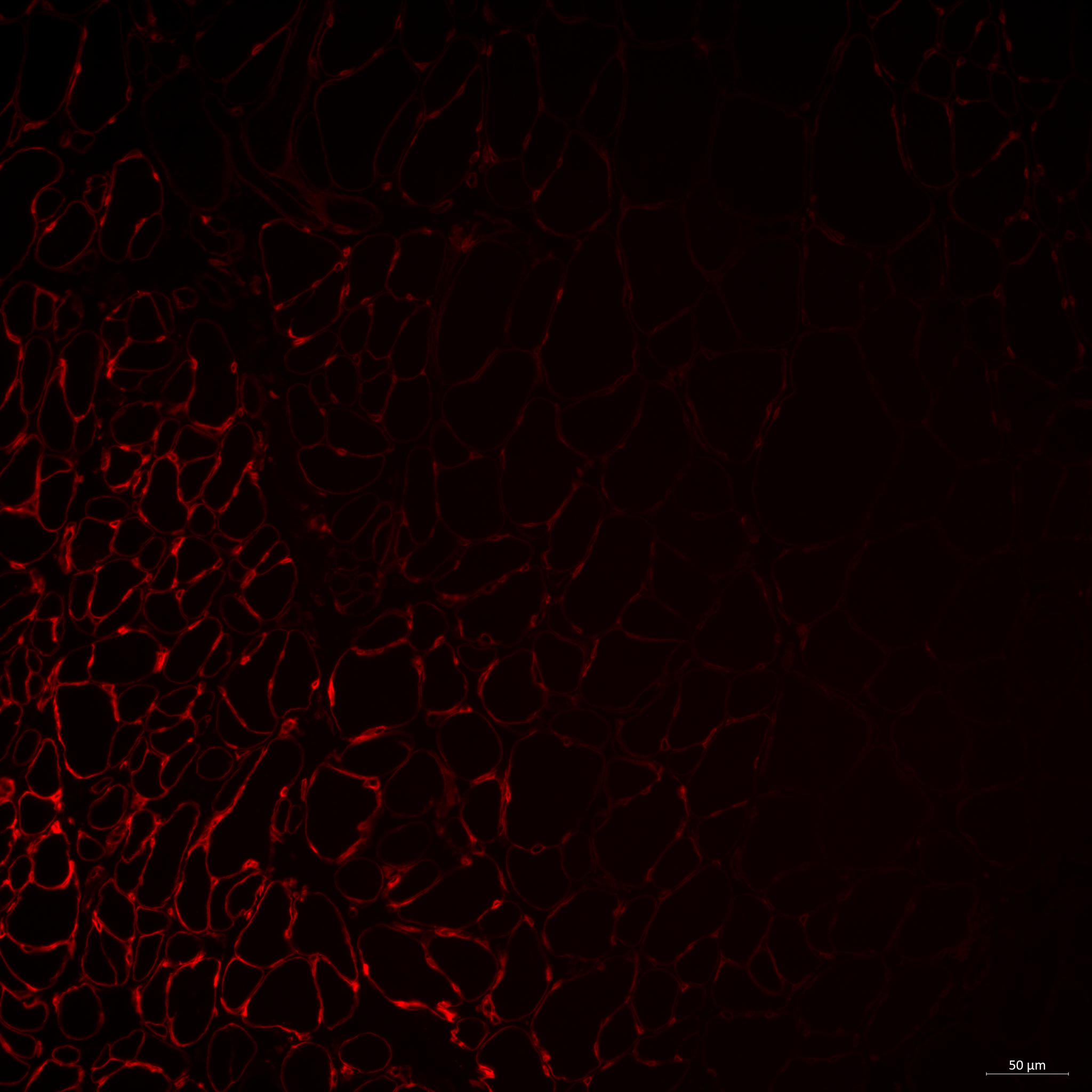

Supplement: Supplementary file 6 — Source data Fig. 4 [file 44319_2026_834_MOESM6_ESM.zip › Figure 4/4B/OPMD hLAMC1.tif]

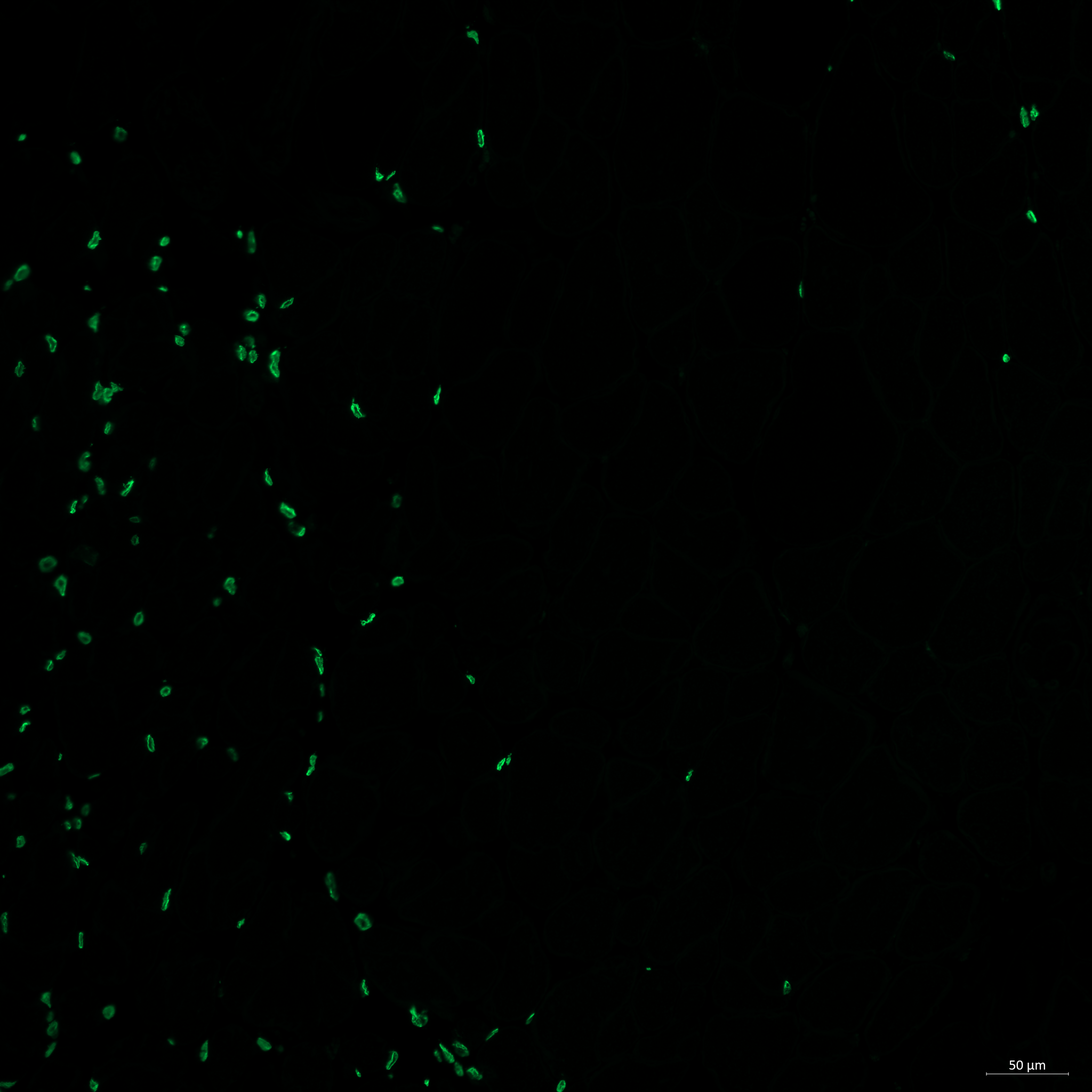

Supplement: Supplementary file 6 — Source data Fig. 4 [file 44319_2026_834_MOESM6_ESM.zip › Figure 4/4B/OPMD hlaminAC.tif]

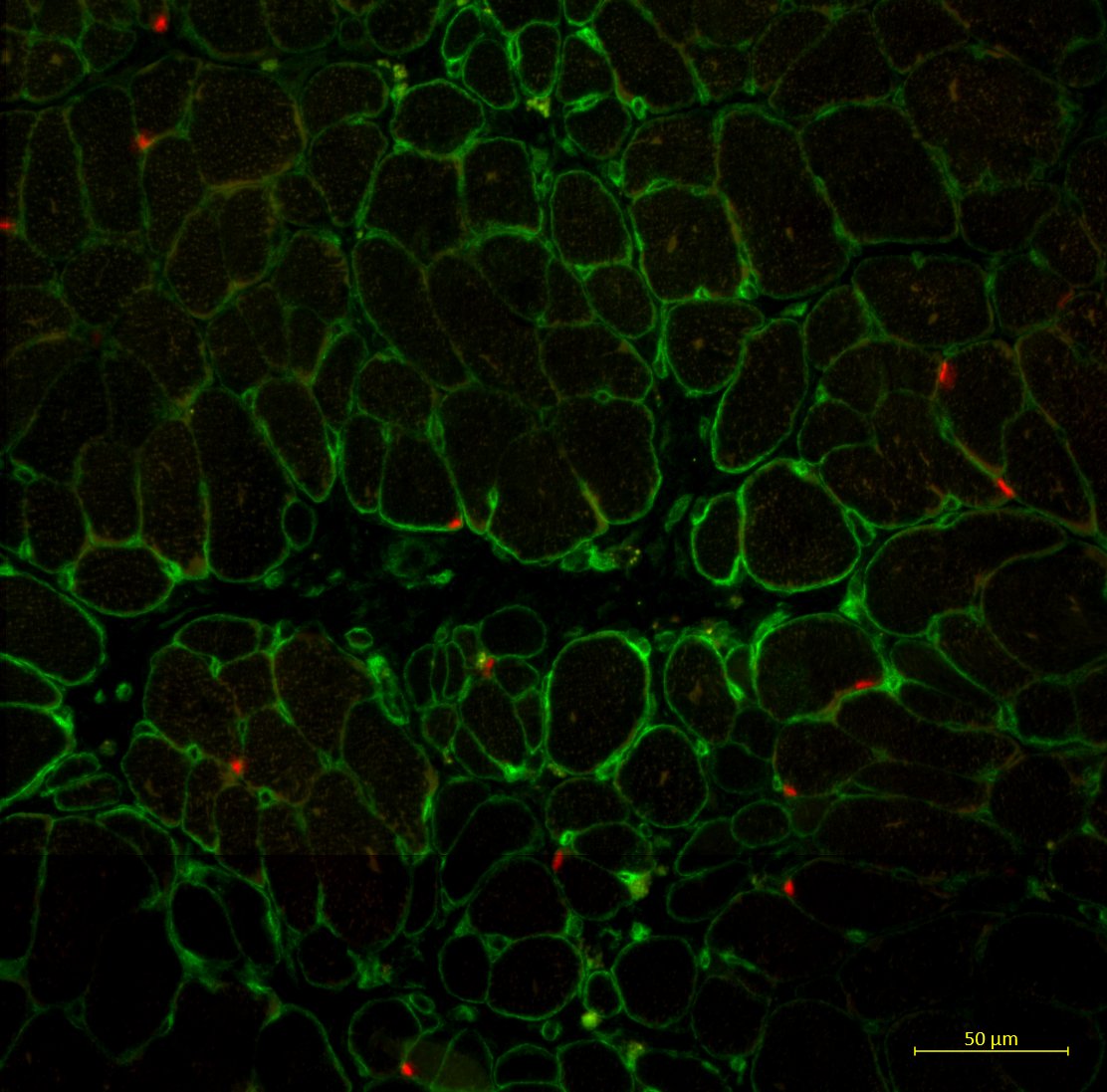

Supplement: Supplementary file 6 — Source data Fig. 4 [file 44319_2026_834_MOESM6_ESM.zip › Figure 4/4D/CTL hLAMC1 pax7.jpg]

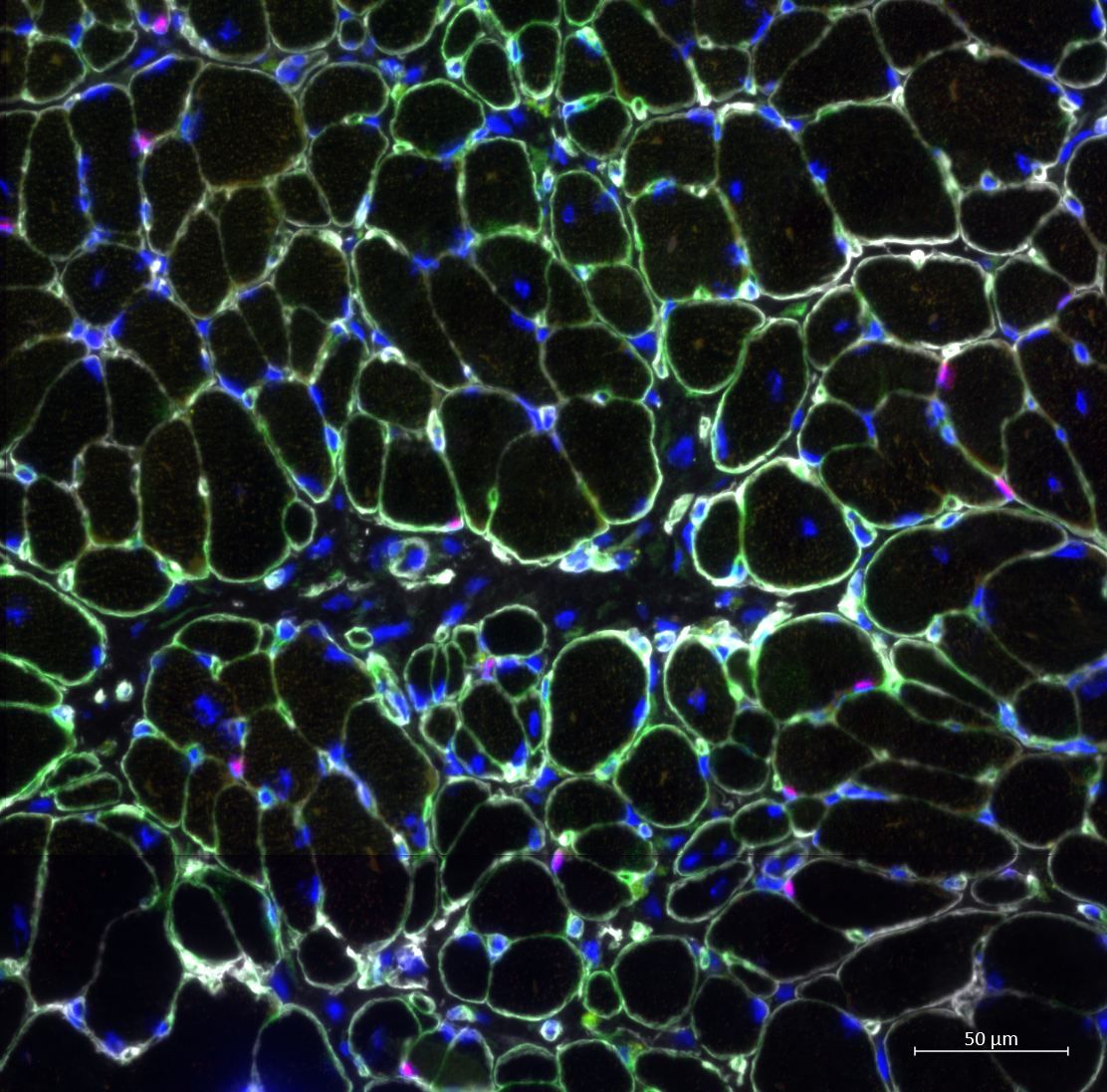

Supplement: Supplementary file 6 — Source data Fig. 4 [file 44319_2026_834_MOESM6_ESM.zip › Figure 4/4D/CTL.tif]

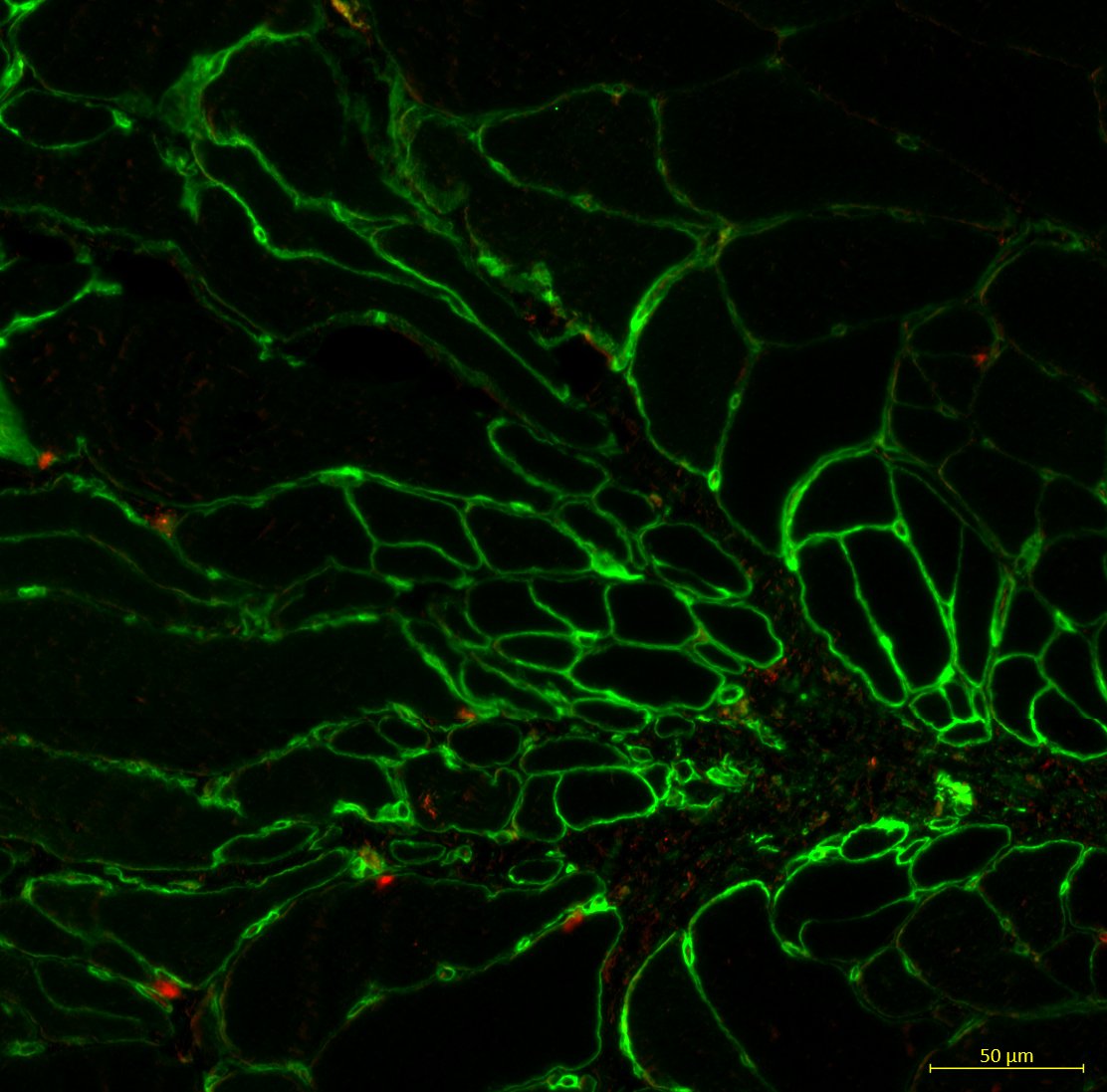

Supplement: Supplementary file 6 — Source data Fig. 4 [file 44319_2026_834_MOESM6_ESM.zip › Figure 4/4D/DMD hLAMC1 pax7.jpg]

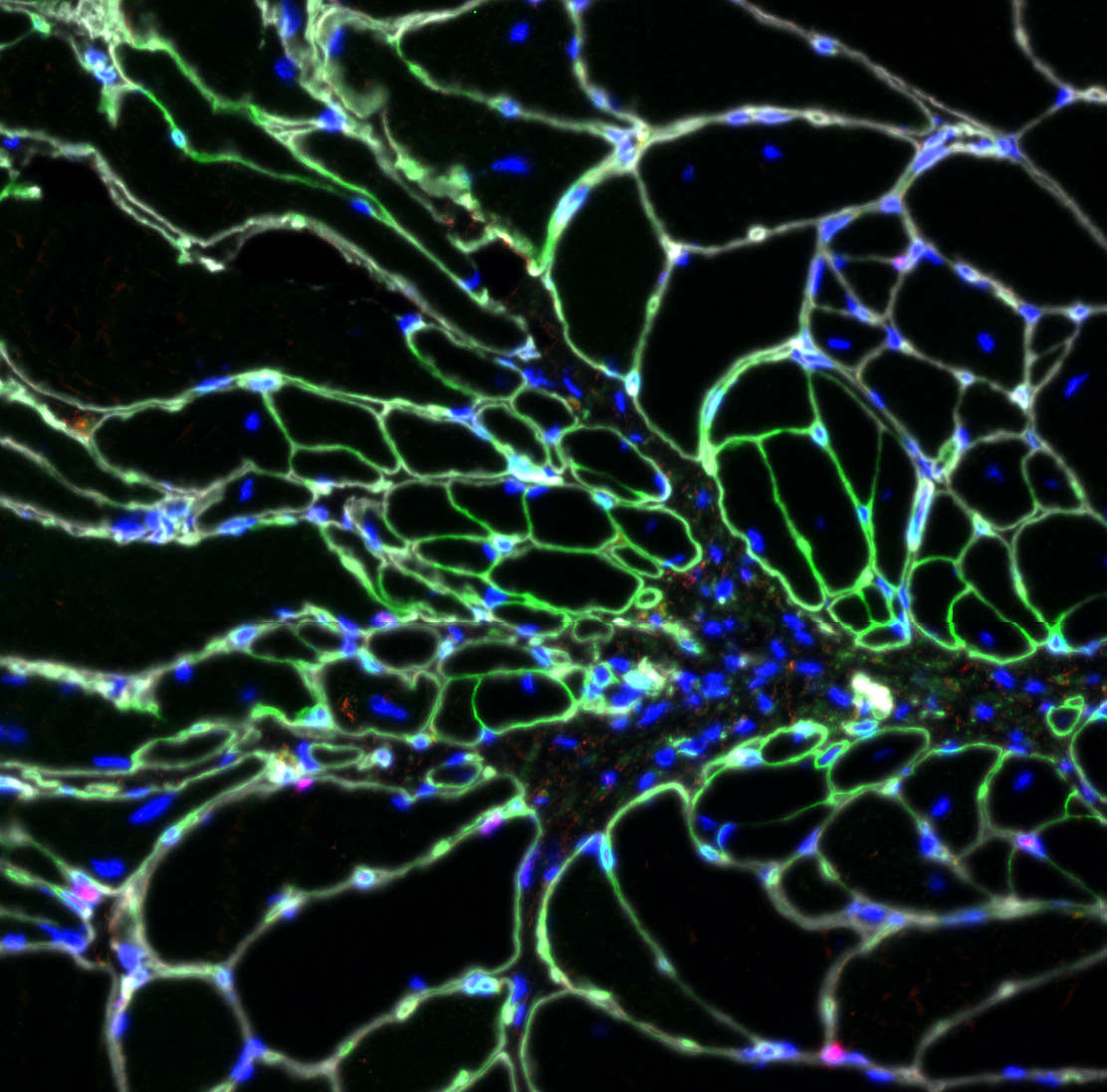

Supplement: Supplementary file 6 — Source data Fig. 4 [file 44319_2026_834_MOESM6_ESM.zip › Figure 4/4D/DMD.tif]

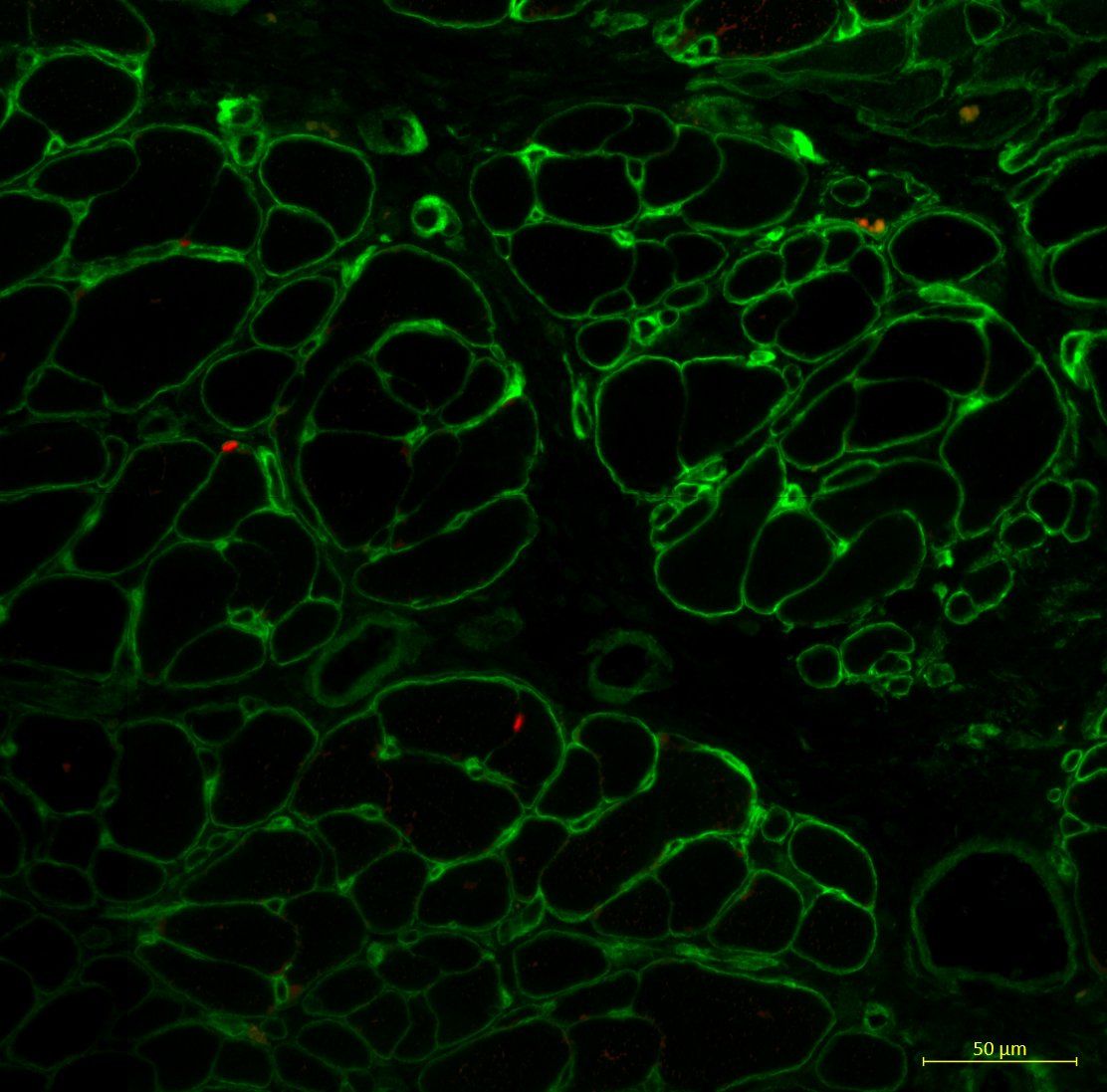

Supplement: Supplementary file 6 — Source data Fig. 4 [file 44319_2026_834_MOESM6_ESM.zip › Figure 4/4D/OPMD hLAMC1 pax7.jpg]

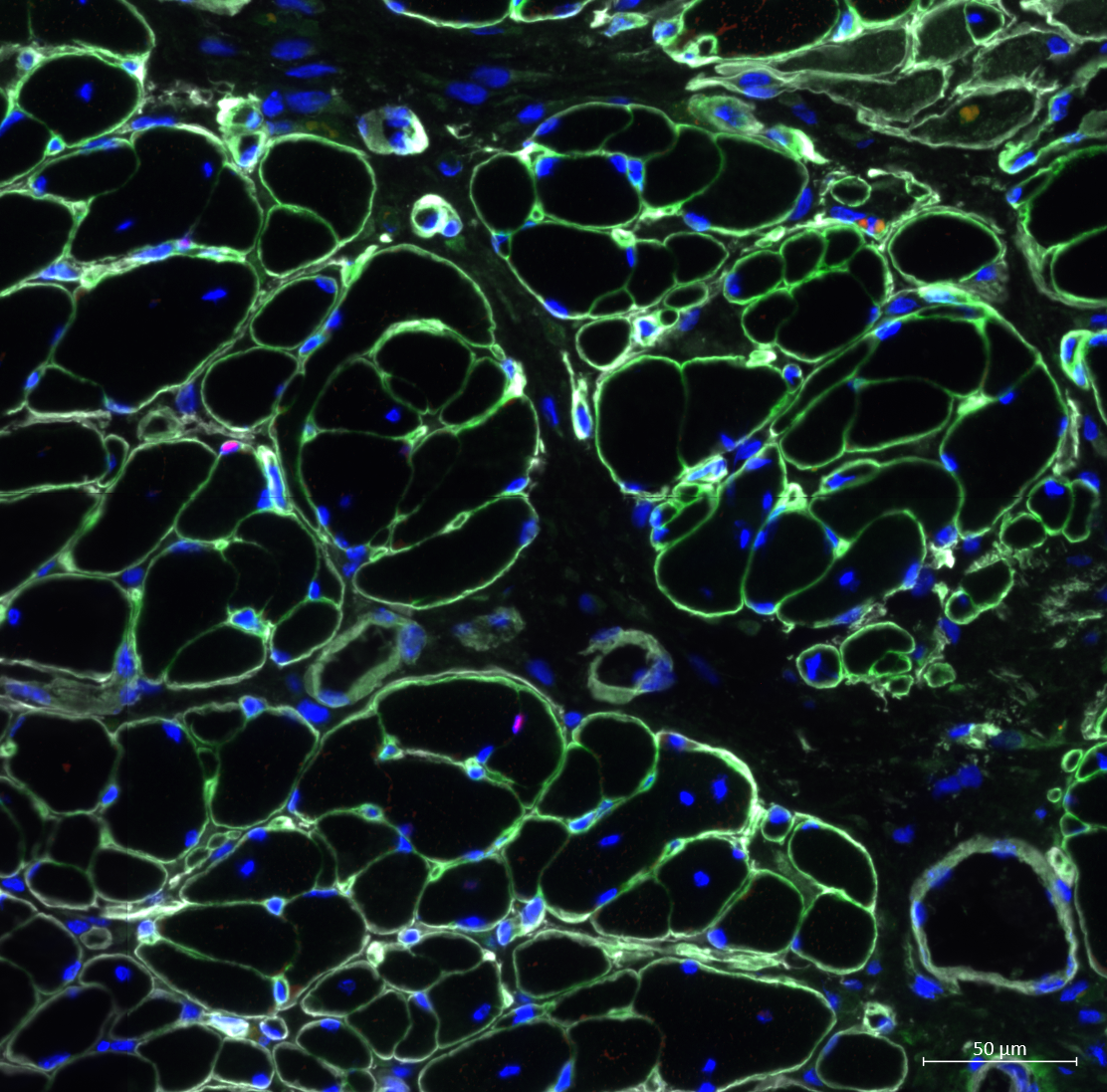

Supplement: Supplementary file 6 — Source data Fig. 4 [file 44319_2026_834_MOESM6_ESM.zip › Figure 4/4D/OPMD.tif]

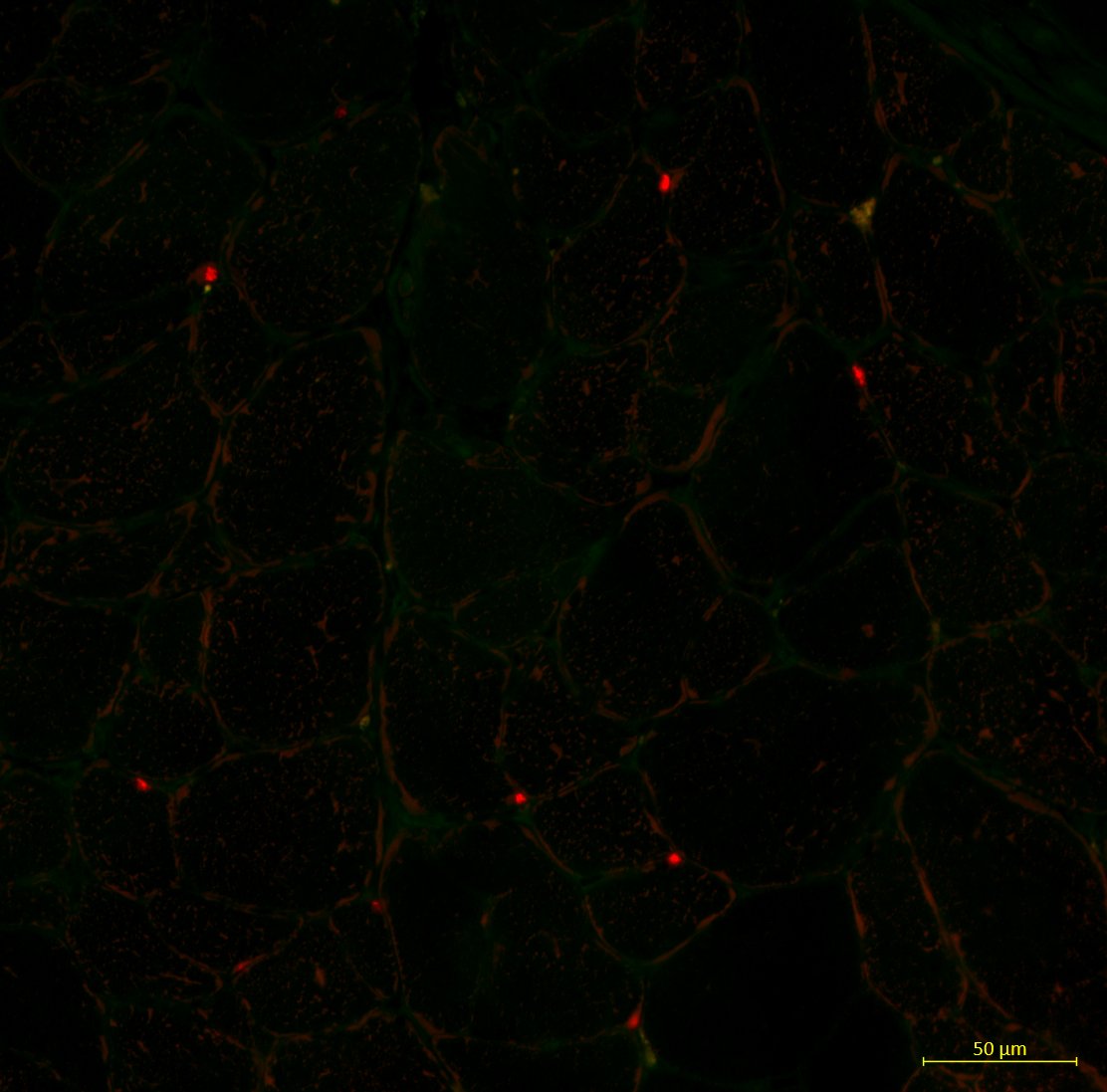

Supplement: Supplementary file 6 — Source data Fig. 4 [file 44319_2026_834_MOESM6_ESM.zip › Figure 4/4D/w:o hLAMC1 pax7.jpg]

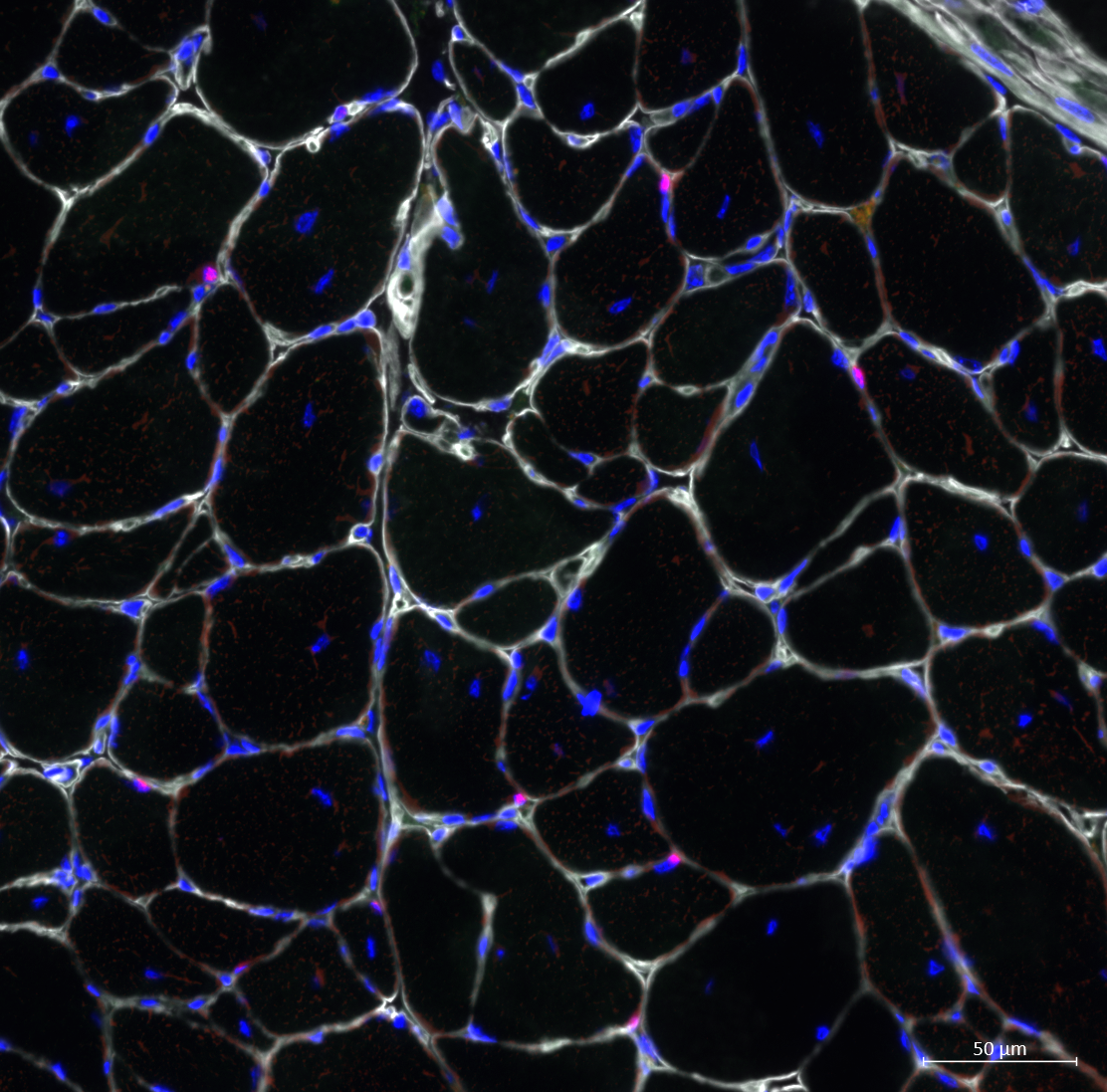

Supplement: Supplementary file 6 — Source data Fig. 4 [file 44319_2026_834_MOESM6_ESM.zip › Figure 4/4D/w:o.tif]

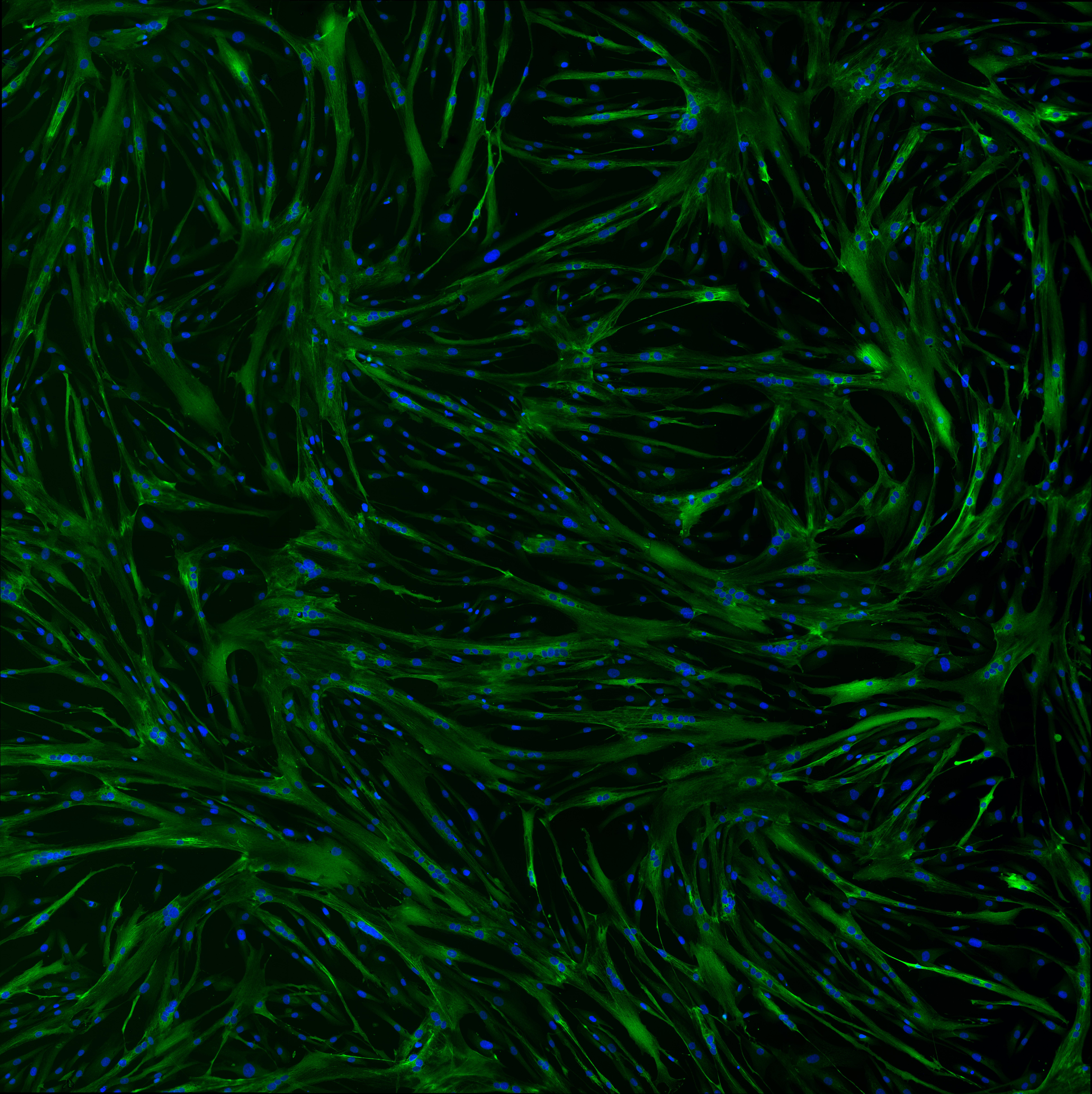

Supplement: Supplementary file 7 — Source data Fig. 5 [file 44319_2026_834_MOESM7_ESM.zip › Figure 5/5C/5 ug.jpg]

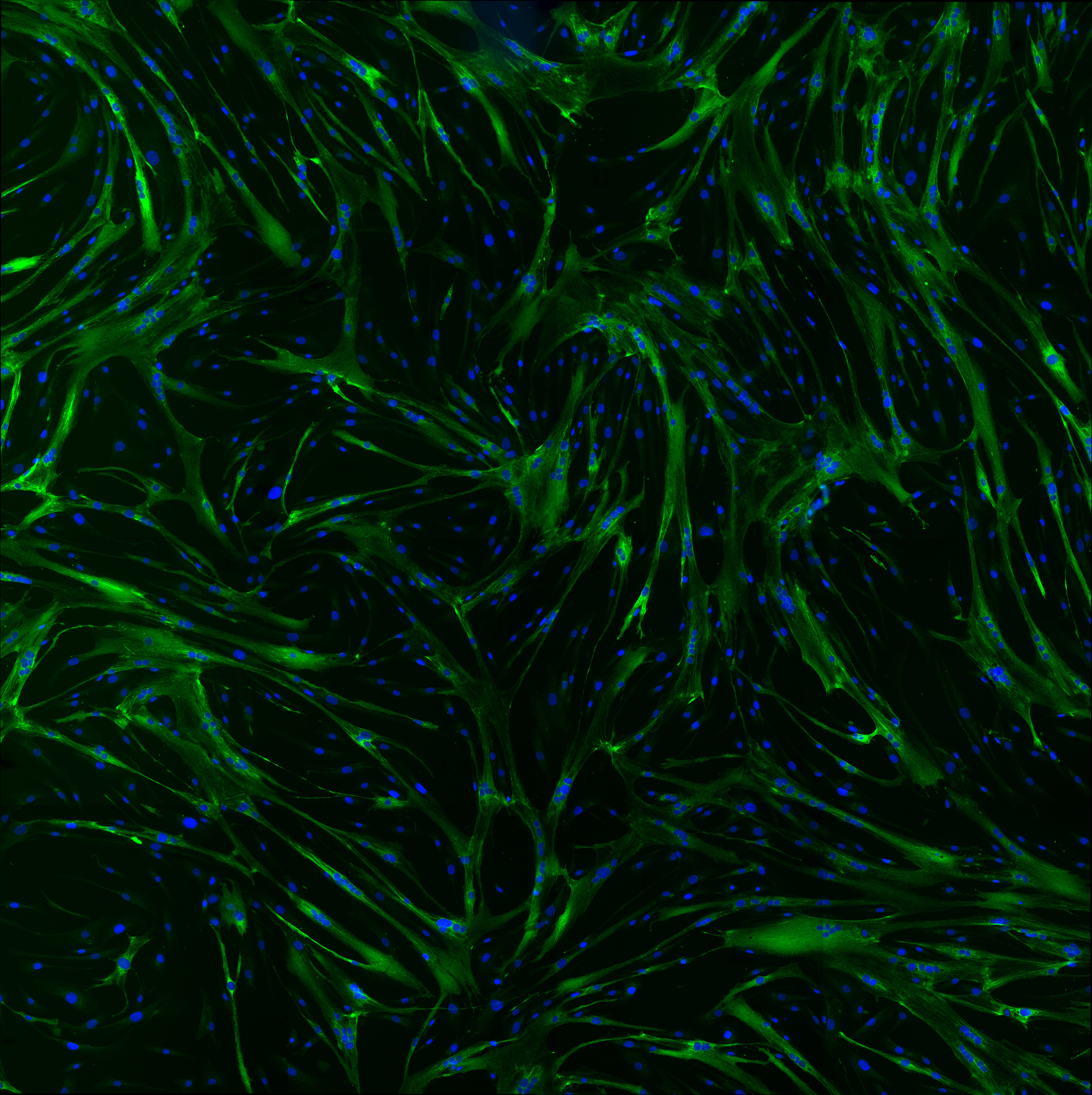

Supplement: Supplementary file 7 — Source data Fig. 5 [file 44319_2026_834_MOESM7_ESM.zip › Figure 5/5C/25ug.jpg]

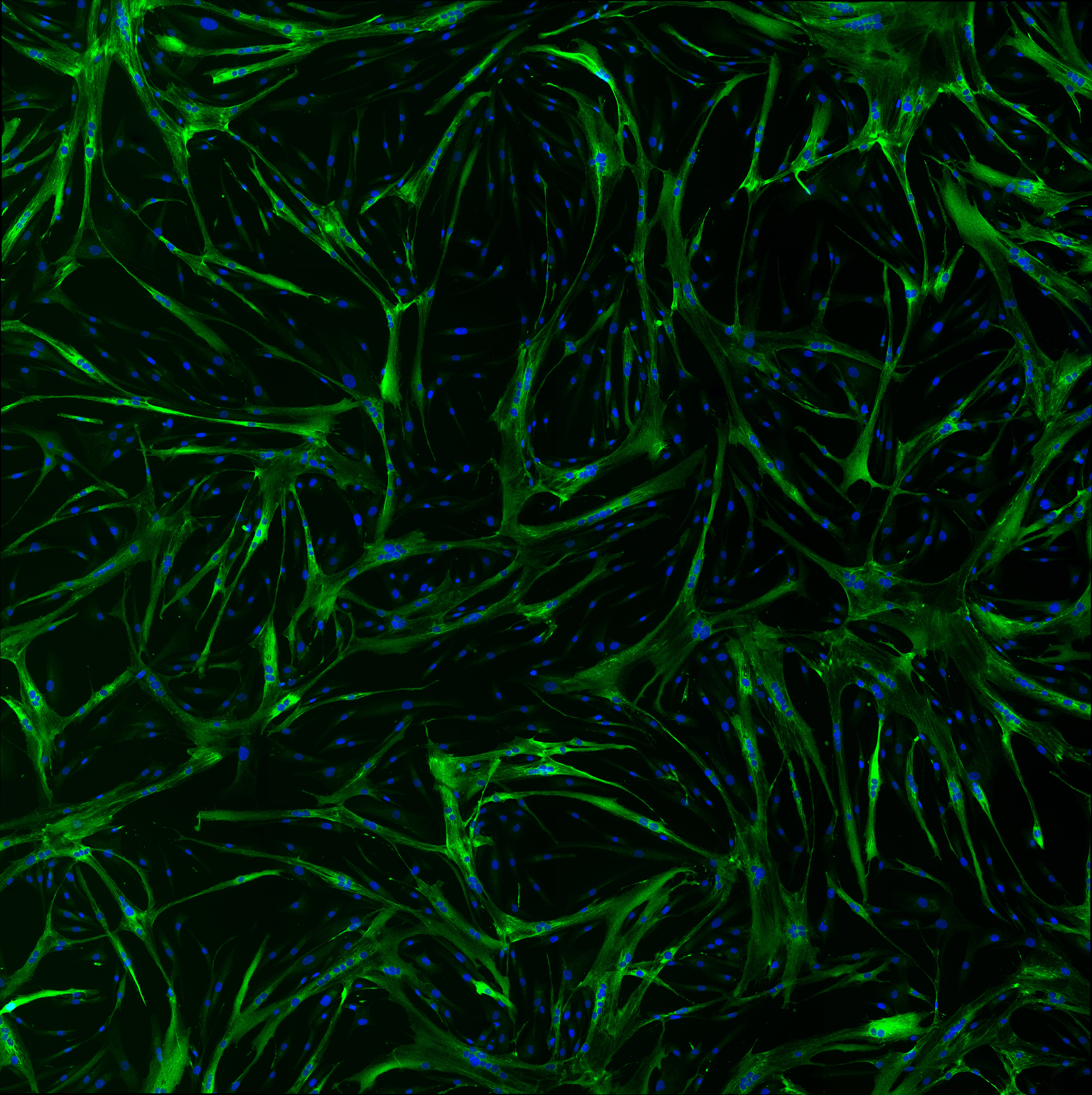

Supplement: Supplementary file 7 — Source data Fig. 5 [file 44319_2026_834_MOESM7_ESM.zip › Figure 5/5C/50ug .jpg]

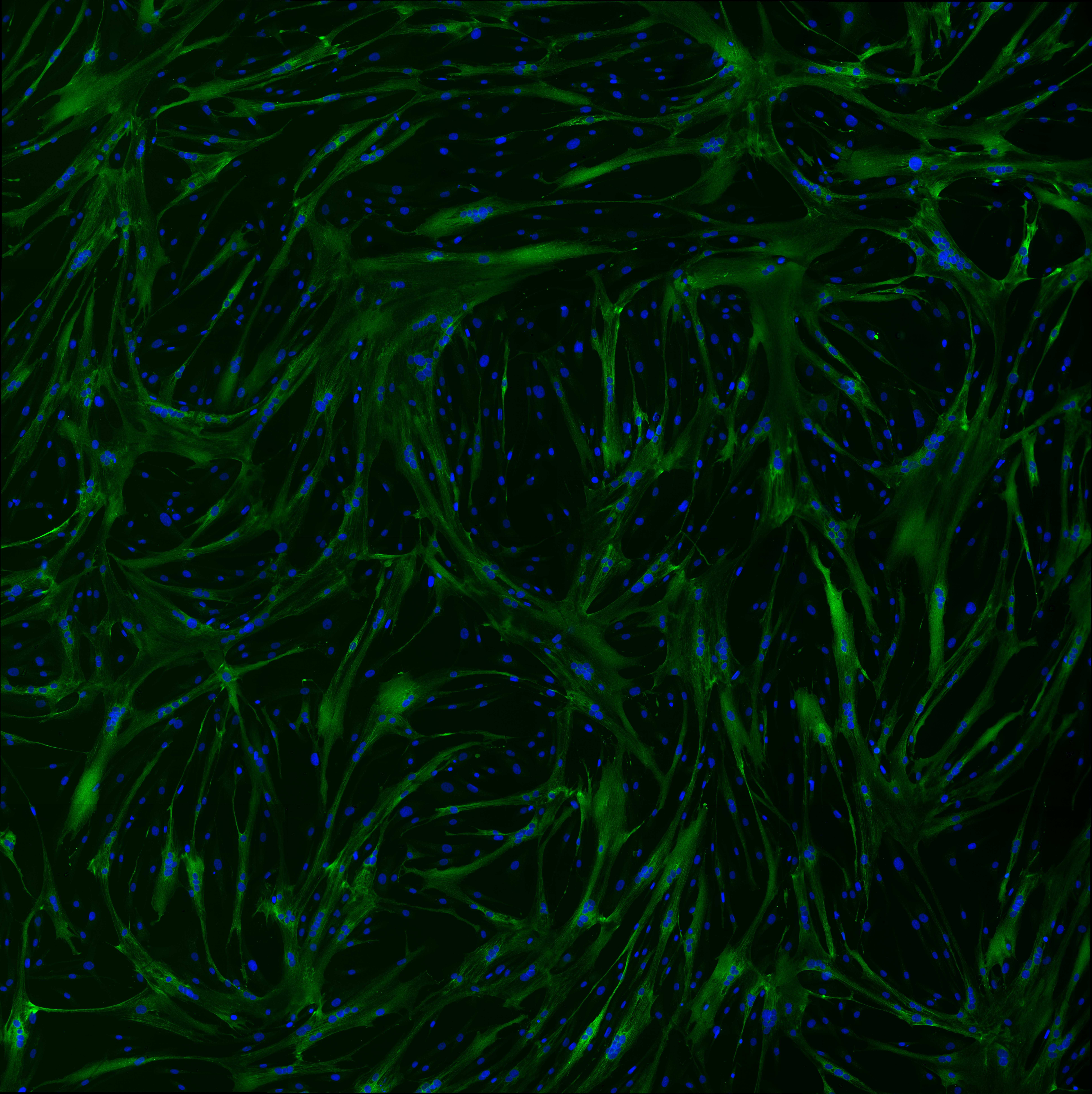

Supplement: Supplementary file 7 — Source data Fig. 5 [file 44319_2026_834_MOESM7_ESM.zip › Figure 5/5C/1,25 ug .jpg]

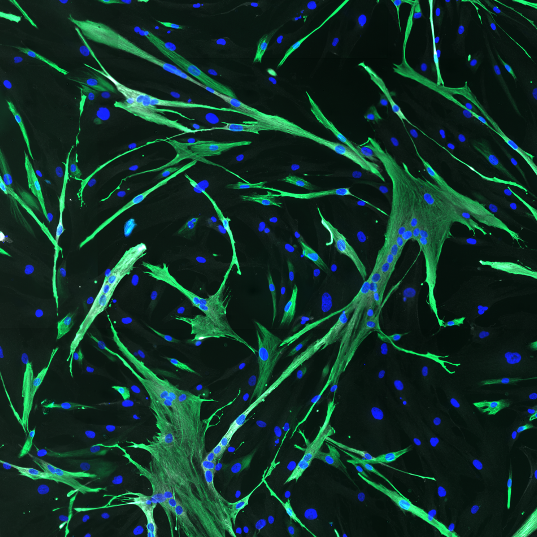

Supplement: Supplementary file 7 — Source data Fig. 5 [file 44319_2026_834_MOESM7_ESM.zip › Figure 5/5F/+FAPs siCOL6.tif]

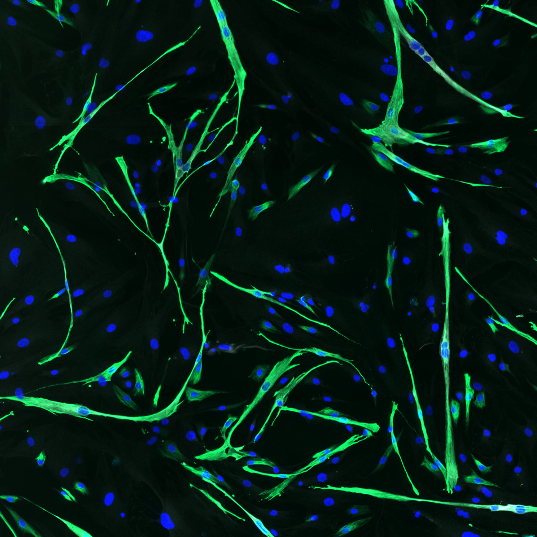

Supplement: Supplementary file 7 — Source data Fig. 5 [file 44319_2026_834_MOESM7_ESM.zip › Figure 5/5F/+FAPs siCTL.tif]

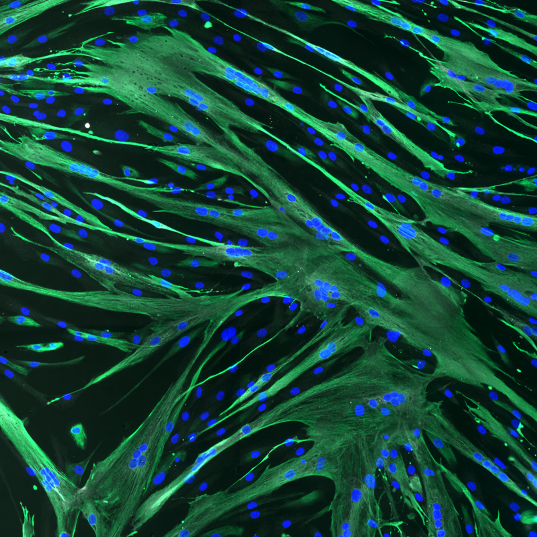

Supplement: Supplementary file 7 — Source data Fig. 5 [file 44319_2026_834_MOESM7_ESM.zip › Figure 5/5F/w:o FAPs.tif]

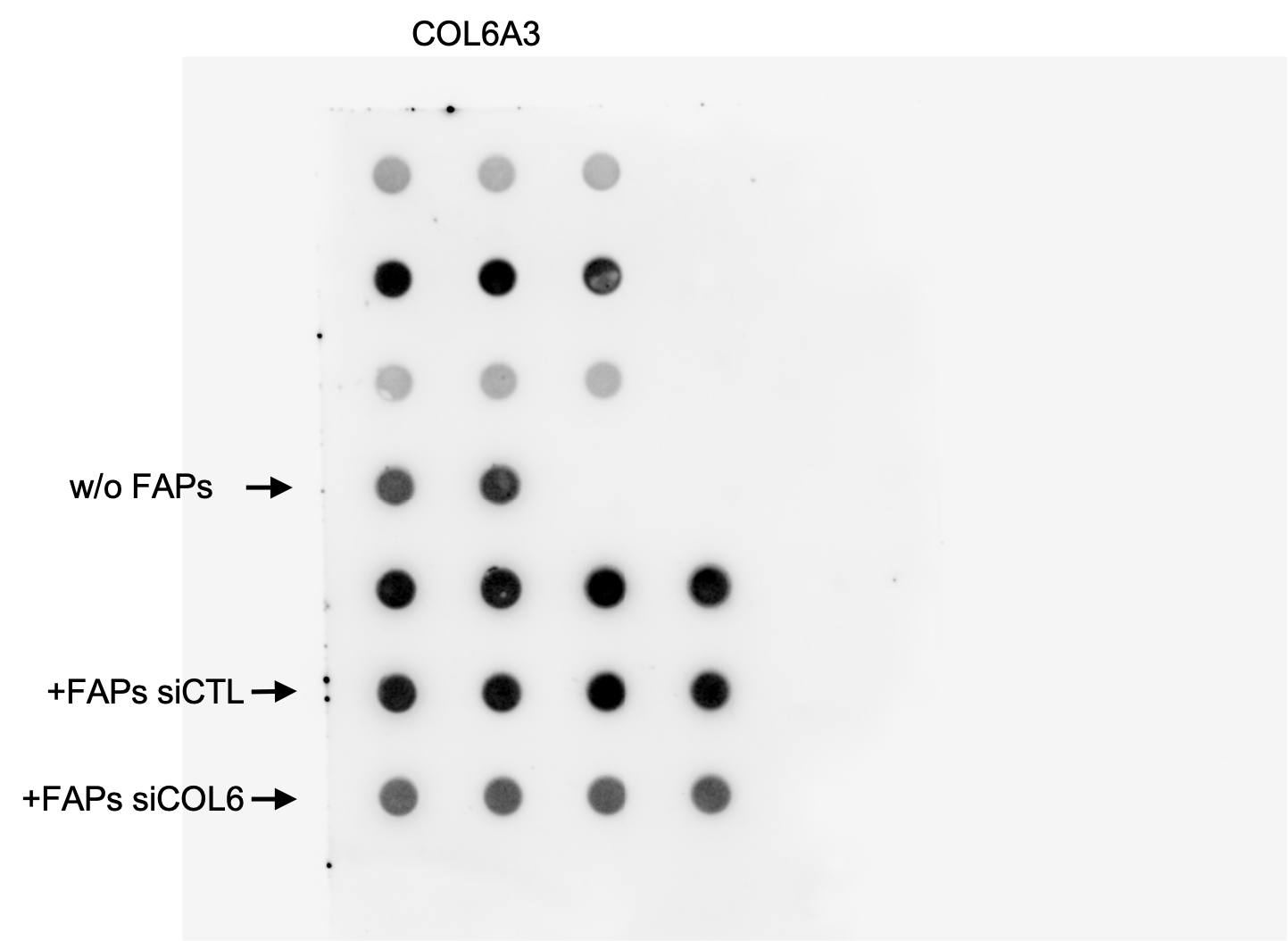

Supplement: Supplementary file 7 — Source data Fig. 5 [file 44319_2026_834_MOESM7_ESM.zip › Figure 5/5E/dot blot col6a3 uncropped.png]

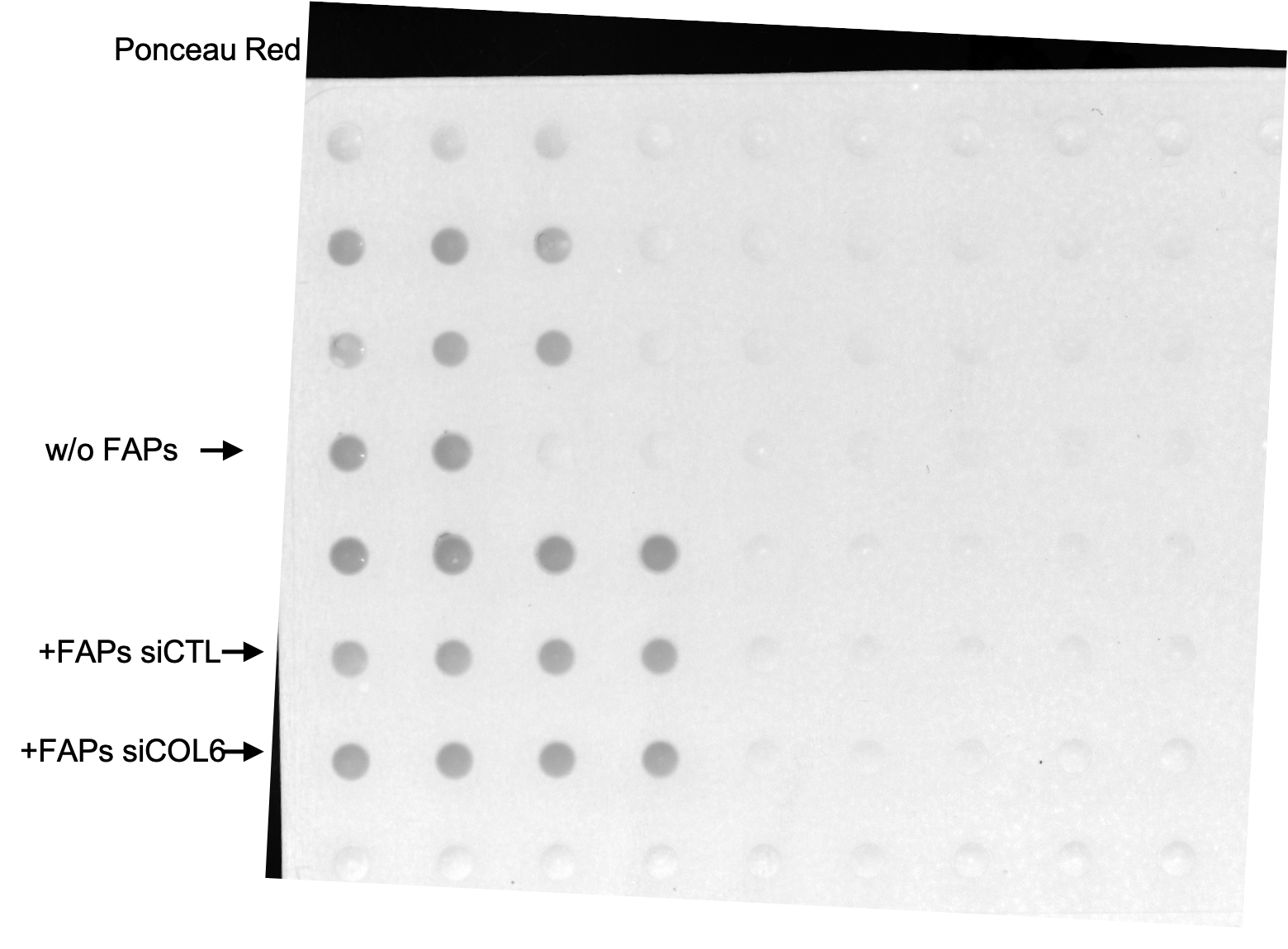

Supplement: Supplementary file 7 — Source data Fig. 5 [file 44319_2026_834_MOESM7_ESM.zip › Figure 5/5E/dot blot ponceau red uncropped.png]
