# Supplementary material for: Collagen VI is a fibrosis-associated signal disrupting muscle regeneration across distinct human myopathies
Source: EMBO Rep. 2026 Jun 19;27(14):4124–40. doi: 10.1038/s44319-026-00834-0 (PMC13400756; doi:10.1038/s44319-026-00834-0)
Supplement: Supplementary file 2 — Peer Review File [file 44319_2026_834_MOESM2_ESM.pdf]

EMBO reports

Collagen VI is a fibrosis-associated signal disrupting muscle regeneration across human myopathies

Laura Muraine, Mona Bensalah, Stephen Gargan, Paul Dowling, Anne Bigot, Valérie Allamand, Jamila Dhiab, Maria Kondili, Sophie Perié, Jean Lacau St-Guily, Gillian Butler-Browne, Vincent Mouly, Kay Ohlendieck, Capucine Trollet, and Elisa Negroni

Corresponding author(s): Elisa Negroni (elisa.negroni@courriel.upmc.fr) , Capucine Trollet (capucine.trollet@sorbonne-universite.fr)

Review Timeline:

Transfer Date:	18th Dec 25
Editorial Decision:	9th Jan 26
Revision Received:	3rd Apr 26
Editorial Decision:	6th May 26
Revision Received:	22nd May 26
Accepted:	3rd Jun 26

Editor: Achim Breiling

Transaction Report: This manuscript was transferred to EMBO Reports following peer review at Review Commons.

(Note: With the exception of the correction of typographical or spelling errors that could be a source of ambiguity, letters and reports are not edited. Depending on transfer agreements, referee reports obtained elsewhere may or may not be included in this compilation. Referee reports are anonymous unless the Referee chooses to sign their reports.)

Revision Plan

Manuscript number: RC-2025-03278
Corresponding author(s): Elisa, Negroni

[The "revision plan" should delineate the revisions that authors intend to carry out in response to the points raised by the referees. It also provides the authors with the opportunity to explain their view of the paper and of the referee reports.

The document is important for the editors of affiliate journals when they make a first decision on the transferred manuscript. It will also be useful to readers of the reprint and help them to obtain a balanced view of the paper.

If you wish to submit a full revision, please use our "Full Revision" template. ***It is important to use the appropriate template to clearly inform the editors of your intentions.****]*

1. General Statements [optional]

This section is optional. Insert here any general statements you wish to make about the goal of the study or about the reviews.

Dear Editors,

We are pleased to resubmit our manuscript entitled "Collagen VI is a conserved pro-fibrotic signal disrupting muscle regeneration across distinct human myopathies" to EMBO Reports, following its evaluation through Review Commons. We are grateful for the reviewers' thoughtful and highly constructive feedback, and we are encouraged by their strong support of the study's significance, design, and mechanistic insights.

We provide a point-by-point answer to all the comments and we are convinced that the revised manuscript taking into account the reviewer comments will further strengthens the clarity and impact of our conclusions. We are confident that these advances will be of strong interest to the readership of EMBO Reports, particularly those focused on tissue regeneration, ECM biology, muscular dystrophies, and translational therapeutics.

2. Description of the planned revisions

Insert here a point-by-point reply that explains what revisions, additional experimentations and analyses are planned to address the points raised by the referees.

Revision Plan

Review COMMONS

*Point-by-point reply that explains what revisions, additional experimentations and analyses are planned to address the points raised by the referees are highlighted in **blue.***

Reviewer #1 (Evidence, reproducibility and clarity (Required)):

The study aims to define important changes in protein expression in three different myopathies: DMD, OPMDI, and IBM. The authors used fibrotic myopathies to investigate their expression profile using proteomics and defined the expression of proteins specifically associated with the extracellular matrix. Many changes were found, which were unique to one of the myopathies; however, one feature present in all three diseases was an expression of collagen VI as a common disease-associated protein. Collagen VI is expressed mainly by FAP, and in a coculture experiment, FAPs isolated from patients suffering from each of the three myopathies caused impaired myogenic differentiation in co-culture experiments. Transplantation of FAPS in immunocompromised mice caused enhanced fibrosis and a loss of PAX7+ myogenic stem cells. Myogenic differentiation was rescued in the co-culture experiment with patient-derived FAPs after siRNA-mediated knockdown of Col VI.

Overall, the manuscript is well written, and the experiments are very well designed and executed. The study provides a novel insight into a common pathogenic mechanism in myopathies.

1. However, I am unable to see a significantly elevated expression of Collagen VI in cultured DMD-derived FAPs, which to me looked like control levels, yet the functional studies suggest that siRNA-mediated knockdown rescues myogenic differentiation in DMD-derived FAPS indistinguishable from OPMDI-derived FAPs. What is your explanation for this? I think it is advisable to perform some other form of quantitative assessment, such as a Western blot to quantify ColVI expression in FAP cultures.

The images in Figure 3F, showing immunostaining of ECM proteins produced by FAPs in vitro, including Collagen VI, were not intended to demonstrate differences in protein secretion between control and pathological FAPs, but rather to confirm that these cells are capable of producing the proteins identified in our proteomic analysis. Furthermore, we are not convinced that a simple quantification of this protein after culturing FAPs alone would be sufficient to demonstrate a more robust production of Collagen VI: co-culturing these cells with myotubes might be required to stimulate FAPs to produce Collagen VI.

2. In the discussion, a lot of the disease-specific alterations in protein expressions are described, which I believe should be all mentioned in the results section. Thus, the discussion needs to be rewritten and should therefore be refocussed on the difficulty to exploit the findings therapeutically, given the requirement for ColVI in muscle tissue.)

Thank you for the comment. We agree and will restructure the manuscript accordingly. We will add more information on disease-specific protein expression changes in the Results section, and we will discuss the therapeutic challenges linked to the essential role of ColVI in muscle tissue.

Revision Plan

Reviewer #1 (Significance (Required)):

The strength of this manuscript is the unifying observation that Col VI seems to be a novel drug target to modulate disease severity and to enhance myogenic regeneration. However, since ColVI is also required, as a loss is also associated with muscle disease, only a small therapeutic window will be present for most patients. Thus, at present, it is unclear how easy the observation could be therapeutically exploited. It will be interesting to see how generally the observation might be? An older report by the Campbell lab also reported that in sarcoglycan-associated limb-girdle muscular dystrophy collagen VI is elevated and that muscle pathology was reduced after crossing γ-sarcoglycan-null mutants with a Col6a2-deficient mouse; however that there was no gain in muscle function. Unfortunately, the authors of the current study did not cite this previous study and did not test whether this previous observation would also apply to mouse models for each of the three human myopathies under study. I am not suggesting performing such a study, as it would essentially be a follow-up study. However, this important work needs to be cited and should also be discussed.

We thank the reviewer for this observation and apologize for not including this very relevant reference in the initial version of our manuscript. We will incorporate it in the revised version and dedicate an extensive section in the Discussion to address this point.

Reviewer #2 (Evidence, reproducibility and clarity (Required)):

Summary
In this study, the authors used liquid chromatography coupled with mass spectrometry to analyse three human myopathies (DMD, Duchenne Muscular Dystrophy; OPMD, Oculopharyngeal Muscular Dystrophy; IBM, Inclusion Body Myositis) to gain greater understanding of fibrosis as it is a major marker of disease progression and obstacle to muscle repair. The proteomics data revealed overlapping and distinct protein signatures between the three human myopathies. Through further approaches involving the transplantation of human fibro-adipogenic progenitor cells (FAPs) from dystrophic muscles in regenerating mouse muscles, the authors showed that FAPs produce ECM proteins, including Collagen VI, and contribute to the muscle ECM remodelling. Dystrophic FAPs are associated with a decline in the number of muscle stem cells and reduced fibre size, and Collagen VI is sufficient and necessary for FAP-mediated decrease in fusion index. Altogether, this manuscript reveals that FAPs are major drivers of the ECM remodelling underpinning fibrosis in human myopathies.

Major comments
1) My major comment relates to some conclusions which misrepresent slightly the complexity of the models and the data obtained.
An example of this is the title of the manuscript: 'Collagen VI is a conserved pro-fibrotic signal disrupting muscle regeneration across distinct human myopathies'. The manuscript showed that Collagen VI is upregulated in the three human myopathies examined but has not showed that it is a pro-fibrotic signal. The article showed that FAPs are pro-fibrotic, and separately showed that Collagen VI affects the fusion index and muscle stem cell numbers in a regenerating

context.
The statement made in the introduction that FAPs are the key ECM producing cells in muscle ignores that other cell types can be major ECM producing cells in different circumstances, including macrophages, endothelial cells and muscle cells. Therefore, the proteomics data from whole human muscles cannot be directly attributed to FAPs only.
Also, the environment of fibrotic dystrophic muscles is distinct from that of regenerating muscles. So, I would encourage the authors to exercise caution in their conclusions as I am not sure that the effect of FAPs in one context translates well in another context.

We thank the reviewer for this comment and for highlighting important conceptual aspects regarding the interpretation of our data. To address the reviewer's pertinent remark concerning the conclusions and the title, we propose revising the current title ("Collagen VI is a conserved pro-fibrotic signal disrupting muscle regeneration across distinct human myopathies") to more accurately reflect the scope of our findings, emphasizing fibrosis-associated remodeling and the contribution of Collagen VI, without implying that Collagen VI alone is sufficient to drive fibrosis.

Following the reviewer's comment, with which we fully agree, we will revise the sentence in the introduction. The revised sentence will acknowledge the contribution of other cell types to ECM production depending on the pathological and temporal context.

We again agree with the reviewer that fibrotic dystrophic muscle differs substantially from regenerating muscle. In our study, we used a regenerative model to mimic muscle regeneration and to investigate how FAPs behave during ongoing regeneration and how they influence the satellite cell niche and myofiber formation. Our aim was not to equate regenerating and chronically fibrotic environments, but rather to explore how fibrosis-associated cues, identified in human myopathies, can impact the regenerative process. The reviewer's insightful comment indeed provides an excellent basis for future investigations aimed at elucidating FAP behavior within a murine fibrotic environment. Such studies would require the use of specific mouse models that recapitulate the genetic background, the extent of muscle fibrosis, and the disease-specific signals of each of the three pathologies studied, combined with an immunodeficient background to allow engraftment of human cells. While beyond the scope of the present work, this represents an important direction for future studies.

2) Experiments carried out in Figs. 1 and 5 raise several questions. In the absence of a clear answer, it would be wise to moderate the interpretation of the findings. FAP from diseased muscles proliferate more rapidly than FAPs from healthy muscles. When co-cultured with muscle cells or transplanted in regenerating mouse muscles, do they invade the co-culture (or muscle) and prevent myoblasts (or muscle cells) to proliferate, interact with each other and fuse? Are FAPs affecting fusion because they affect differentiation or because cells die or stop proliferating? Or do they delay differentiation?

We thank the reviewer for highlighting this point. Fig1 and Fig5 analyse how FAPs influence myoblast fusion an differentiation into myotubes using co-culture experiments. To analyse this we systematically use the fusion index. We have preliminary data obtained from co-culture of myoblasts with control and fibrotic (OPMD and DMD) FAPs that allow us to reply to the concern of the referee.

When FAPs are co-cultured with myoblasts under differentiation conditions (serum-free DMEM), conditioned medium produced by differentiating myoblasts induces an increase in the

proliferative activity of fibrotic FAPs. Importantly, this does not result in a total number of FAPs sufficient to colonize the entire culture dish. This is evident in Fig. 1F, which contains representative images of co-culture experiments showing the presence of FAPs (desmin negative/DAPI-positive cells). Therefore, we can exclude the possibility that FAP overgrowth leads to culture dish invasion and indirectly inhibits myoblast proliferation. Moreover, in our previous study (Bensalah et al., JCSM, 2022, Fig3B), we demonstrated that FAPs isolated from fibrotic muscle are smaller in size, making them less likely to occupy a substantial surface area of the culture dish. Consequently, it is unlikely that fibrotic FAPs impair myoblast–myoblast interactions through steric hindrance. This interpretation is further supported by EdU incorporation analysis in myoblasts during co-culture, which shows that the percentage of proliferating myoblasts does not differ between myoblasts cultured alone and those co-cultured with FAPs (see figure below).

Figure for referee with unpublished data and its description has been removed upon request by the authors.

Regarding the potential death of myoblasts in co-culture due to the presence of FAPs, we did not observe any cell mortality during the course of the co-culture experiments. After 5 days of differentiation, we quantified the same proportion of cells as initially seeded (ie 70% myoblasts and 30% FAPs), either in co-culture with control or DMD FAPs:

Figure for referee with unpublished data and its description has been removed upon request by the authors.

3) Could the authors interpret the distinct signature of the human myopathies in light of the fact that different muscles and patients of different ages were analysed?

Thank you for pointing out this important discussion point. The muscles for each pathology were compared to the same type of muscle from an age-matched, unaffected subject (for example, cricopharyngeal muscle from an OPMD patient compared with cricopharyngeal muscle from a control subject) in order to identify differences between normal and pathological conditions, without introducing potential variability linked to muscle specificity. We believe that the proteins we identified for each condition correspond to those expressed at a very specific stage of the disease. For example, it is possible that the increased protein levels observed in DMD muscle around 14 years of age would differ from those that might be found if the same analysis were performed on muscle from a 20-year-old patient. We will carefully integrate all these elements of discussion in the revised version of our manuscript.

Minor comments

1) In your Introduction, cite reference to support the statement that FAPs are the ECM producing cells in muscles. Report on studies showing the involvement of other cell types in ECM production.

In the final version of our manuscript, we will integrate references to support the statement that FAPs are ECM producing cells within the muscles and showing the involvement of other cell types in ECM production.

2) A brief description of the ECM structure in muscles would be useful (interstitial and basal lamina)

We will integrate a description of the ECM structure in the revised version of our manuscript.

3) It would be useful if the authors could add information about the sex of patients used in the analyses.

We will add information regarding the sex of the patients, as requested by the reviewer, in the revised version of the manuscript.

4) Figure 4: the text mentions that csa was calculated while the figure legend indicates that minimum ferret's analysis was carried out.

We would like to thank the reviewer for pointing this out. The mistake will be corrected in the revised version: we used the minimum ferret’s to carry out the analysis.

5) The experimental design for the muscle regeneration upon FAPs transplantation varies significantly between OPMD and DMD. What is the rationale for this?

Revision Plan

Our lab has performed for years transplantation of human cells following the induction of muscle regeneration in the tibialis anterior of immunodeficient mice, using two different methods to induce muscle degeneration: cryodamage and notexin injection. The final engraftment results have always been comparable. Switching to notexin injection instead of cryodamage, which requires more invasive surgery, and using a single injection instead of three not only simplifies the procedure but also addresses the 3R principle by reducing animal suffering and the number of interventions.

Reviewer #2 (Significance (Required)):

The strength of this study lies in the proteomics analyses carried out on human biopsies, which reveal a disruption in the balanced production of ECM components that may contribute to the high levels of fibrosis observed. The separate demonstration that FAPs synthesise ECM proteins and when co-cultured with myocytes, inhibit their differentiation suggest strongly that they may be essential players in the excessive fibrosis observed in vivo in human myopathies. One limitation of this study was to test the role of FAPs in vivo in a muscle regeneration model, and not in a model of muscular dystrophy, as these two models are significantly different. This study is of interest to scientists with interest in basic or clinical research in fibrosis and/or muscular dystrophies. This reviewer has expertise in muscle regeneration and skeletal muscle stem cell biology.

Reviewer #3 (Evidence, reproducibility and clarity (Required)):

A-Summary
In this study, Muraine et al. perform a comparative proteomic analysis of three distinct human myopathies characterized by fibrosis: Duchenne muscular dystrophy (DMD), oculopharyngeal muscular dystrophy (OPMD), and inclusion body myositis (IBM). Using liquid chromatography coupled to tandem mass spectrometry (LC-MS/MS), they compare fibrotic muscles from patients to those of age-matched healthy controls and identify matrisome alterations specific to each disease. The authors further isolate fibro-adipogenic progenitors (FAPs) from patient biopsies and characterize their secretion of key ECM proteins both in vitro and in vivo. Functional experiments demonstrate that FAP-secreted ECM components, including collagen VI and basal lamina proteins, modulate muscle stem cell behavior and tissue regenerative capacity. Notably, silencing COL6 in patient FAPs restores myogenic fusion, directly linking pathological collagen VI deposition to regeneration failure.
This work advances understanding of ECM remodeling in human muscle diseases and identifies collagen VI as a conserved effector of fibrosis and a potential therapeutic target across genetically and clinically distinct myopathies.

B-Major comments
Overall, the study is very well-designed, and the main conclusions are convincing. The comparative proteomic approach, combined with functional assays using patient-derived FAPs, provides novel and clinically relevant insights into ECM remodeling and its consequences on muscle regeneration. However, several points require clarification or additional data presentation to fully support the claims and improve reproducibility.

Figure 1A:
A global view of the muscle section is needed to visually confirm that approximately 60% of the

tissue is fibrotic and 40% contains myocytes, as claimed. In its current form, the quantification does not appear to match the representative images shown.

We propose to include the following images in the supplementary data to better illustrate the Sirius Red staining in larger fields of the muscle sections. If the reviewer and the editor consider it useful, we can also provide new images that are more closely representative of the Sirius Red quantification shown in Fig. 1A.

Figure for referee with unpublished data and its description has been removed upon request by the authors.

Figure 1C:
Higher magnification is required, as individual FAPs are not discernible, only red signal is visible.

We can provide the following images at higher magnification in the supplementary data to better discriminate FAPs stained by CD90 antibody, within the muscle.

Figure for referee with unpublished data and its description has been removed upon request by the authors.

Given the highly fibrotic context described, I strongly recommend including co-staining for CD45 and CD31 in addition to CD90. Fibrosis is known to induce phenotypic plasticity and atypical marker expression, including CD90 upregulation in endothelial and immune cells. Co-labeling with CD45 and CD31 is therefore important to exclude immune and endothelial contamination and confirm that CD90 indeed identifies FAPs. The scale of the graph should also be adjusted, as FAPs appear absent from control samples.

We agree with the reviewer that the dystrophic environment can induce atypical marker expression. When performing the CD90 staining, we also applied a laminin antibody in order to precisely discriminate the muscle architecture. This staining allowed us to clearly identify the endothelial cells in the vessels, counterstained with laminin, and thereby exclude endothelial cells from our CD90 quantification. We will also repeat the CD90 staining using a CD45 antibody to exclude potential immune cells and refine our quantification. We propose to provide these images as supplementary data in the revised version of our manuscript. We will adjust the scale of the graphs in fig1C as requested by the reviewer, and a copy of the new graph is provided below.

Figure for referee with unpublished data and its description has been removed upon request by the authors.

Figure 1E:
In murine models, FAPs are well-established regulators of myogenesis, supporting myoblast differentiation and fusion. It is therefore surprising that, in this study, human FAPs do not promote myotube formation. Although direct evidence for pro-myogenic effects of human FAPs is less extensive than in mice, this discrepancy should be discussed since it is to me a very interesting finding. Possible explanations might include differences in activation state, culture conditions, or communication with MuSCs. Clarifying this point would strengthen interpretation and contextualization within the current understanding of human versus murine FAP biology.

We agree with the referee that this divergent behaviour between human and murine FAPs deserves further discussion. We will address this point in the discussion section, by considering possible explanations, already suggested by the reviewer, which may include differences in activation state (homeostasis/regenerating conditions vs fibrotic condition, culture conditions, different ratio between MuSCs and FAPs).

Figure 3F: Quantification relative to controls is needed to support the visual data and conclusions.

As mentioned for Reveiwer #2, the images in Figure 3F, showing immunostaining of ECM proteins produced by FAPs in vitro, including Collagen VI, were not intended to demonstrate differences in protein secretion between control and pathological FAPs, but rather to confirm that these cells are capable of producing the proteins identified in our proteomic analysis. Furthermore, we believe that simply quantifying this protein in FAPs cultured alone may not adequately reflect their capacity to produce Collagen VI: co-culture with myotubes might be required to elicit a more robust production.

Figures 4 and 5: It is unclear why IBM and/or DMD FAPs are not included in these analyses. Given that the study title emphasizes comparison across DMD, OPMD, and IBM, inclusion (or at least an explanation for exclusion) of IBM and/or DMD samples is required.

We thank the reviewer for this comment. We would like to clarify that the comparative analysis across DMD, OPMD, and IBM FAPs constitutes the core of the study and is presented throughout the manuscript.

The *in vivo* and siRNA-based mechanistic experiments shown in Figures 4 and 5 were designed as proof-of-principle experiments to demonstrate the functional role of COL6, rather than to systematically re-test all three pathologies. Performing these experiments in all three conditions was not technically feasible, as they are particularly demanding in terms of material, time, and in vivo manipulation.

We therefore chose for the *in vivo* part, two conditions that are sufficiently distinct—DMD and OPMD. OPMD and IBM FAPs, both isolated from the cricopharyngeal muscle of aged patients, are largely overlapping. By choosing more distinct conditions, we aimed to demonstrate that the results can be consistently reproduced. We selected a single pathology as a representative example for the siRNA to illustrate the mechanism. Based on the strong similarities observed across the phenotype of DMD, OPMD, and IBM FAPs in our comparative analyses, we propose that this COL6-dependent mechanism is likely shared among the three diseases, although we acknowledge that this was not experimentally tested in all cases. We will clarify this rationale in the revised manuscript.

Similarly, since LAMC1 is consistently upregulated across diseases, the rationale for focusing primarily on collagen VI should be better justified, or the contribution of LAMC1 should be discussed or data included.

In the revised version, we will provide the rationale that motivated our choice of focusing on collagen VI. We are of course aware that collagen VI is not the only molecule that may play a role; however, we decided to start with this molecule because all three chains forming the complete complex were found among the increased and shared proteins. LAMC1, although possibly pivotal in the fibrotic process, represents only one subunit of the much larger laminin family.

C-Minor comments:

The authors of reference 15 need to be cited in the text : "To further delineate the cellular

sources of ECM proteins, we analyzed a publicly available human single-cell RNA sequencing (scRNA-seq) dataset from 15"

We will quote the authors of reference 15 in the text of the revised manuscript

D-Are the suggested experiments realistic in terms of time and resources? Yes, the proposed additions are feasible within a reasonable timeframe.

E-Are the data and methods presented in a way that allows reproduction? Yes, methods are clearly described, and data presentation is generally transparent.

F-Are experiments adequately replicated and is statistical analysis adequate? Yes, the replication and statistical approaches are appropriate.

Reviewer #3 (Significance (Required)):

General assessment
This study provides a comprehensive proteomic and functional analysis of extracellular matrix (ECM) remodeling in three human myopathies characterized by fibrosis: DMD, OPMD, and IBM. Its main strength lies in its integrative design, combining human patient samples, quantitative proteomics, and FAP-derived ECM functional assays, to dissect disease-specific and shared fibrotic mechanisms. The identification of collagen VI as a common and functionally relevant effector of fibrosis across distinct genetic contexts represents a valuable contribution to the field. The use of primary human FAPs derived directly from patient biopsies also adds a high degree of translational relevance.
The main limitations are related to the incomplete inclusion of all disease groups in some analyses. Addressing these issues would further strengthen the reproducibility and generality of the conclusions.

Advance
To my knowledge, this is the first study to perform a comparative ECM proteomic profiling across multiple human myopathies while functionally linking FAP-derived collagen VI to defective myogenic regeneration. Previous work has implicated collagen VI accumulation in muscular dystrophies and in aging-related fibrosis, but its identification here as a shared effector across genetically distinct myopathies broadens its conceptual significance. The demonstration that silencing COL6 in human patient FAPs restores myotube fusion capacity provides direct mechanistic insight and suggests potential therapeutic avenues aimed at modulating FAP-ECM interactions. Thus, the study advances understanding of fibrosis not only in a disease-specific manner but also at a systems level, establishing a unifying ECM signature across fibrotic muscle disorders.

Audience
The study will primarily interest researchers in the fields of muscle biology, fibrosis, extracellular matrix biology, and stromal cell function, as well as those focusing on muscle regeneration and neuromuscular diseases. Its integrative human-based approach will also attract a translational and clinical audience, especially those exploring anti-fibrotic strategies or biomarker discovery. Beyond neuromuscular pathology, the identification of collagen VI as a conserved driver of fibrosis may be relevant to other fibrotic conditions where FAP-like cells contribute to ECM remodeling.

Revision Plan

Field of expertise: Muscle regeneration, mesenchymal stromal cells, adipose biology, connective tissue, cell migration.

Areas outside my primary expertise: Advanced LC-MS/MS analytical pipelines and proteomic normalization methods.

3. Description of the revisions that have already been incorporated in the transferred manuscript

Please insert a point-by-point reply describing the revisions that were already carried out and included in the transferred manuscript. If no revisions have been carried out yet, please leave this section empty.

4. Description of analyses that authors prefer not to carry out

Please include a point-by-point response explaining why some of the requested data or additional analyses might not be necessary or cannot be provided within the scope of a revision. This can be due to time or resource limitations or in case of disagreement about the necessity of such additional data given the scope of the study. Please leave empty if not applicable.

1st Editorial Decision 9th Jan 2026

Dear Dr. Negroni,

Thank you for transferring your manuscript from Review Commons to EMBO reports. I now went through the manuscript, the referee reports from Review Commons (attached again below) and your revision plan.

Judging from your revision plan, it seems that you will be able to adequately address the concerns of the referees during a major revision. As the evaluation of the referees is overall positive and they consider the manuscript as very interesting, I invite you to revise your manuscript accordingly with the understanding that all referee concerns must be addressed in the revised manuscript and/or in a final detailed point-by-point response, as indicated in your revision plan.

Revised manuscripts should be submitted within three months of a request for revision. Please contact me to discuss the revision (also by video chat) if you have further questions or comments regarding the revision, or should you need additional time.

Acceptance of your manuscript will depend on a positive outcome of another round of review at EMBO reports, using the same referees.

When submitting your revised manuscript, please also carefully review the instructions that follow below.

PLEASE NOTE THAT upon resubmission revised manuscripts are subjected to an initial quality control prior to exposition to re-review. Upon failure in the initial quality control, the manuscripts are sent back to the authors, which may lead to delays. Frequent reasons for such a failure are the lack of the data availability section (please see below) and the presence of statistics based on n=2 (the authors are then asked to present scatter plots or provide more data points).

When submitting your revised manuscript, we will require:

1) a .docx formatted version of the final manuscript text (including legends for main figures, EV figures and tables), but without the figures included. Please make sure that changes are highlighted to be clearly visible. Figure legends should be compiled at the end of the manuscript text.

2) individual production quality figure files as .eps, .tif, .jpg (one file per figure), of main figures and EV figures. Please upload these as separate, individual files upon re-submission. Please make sure that all figure panels are called out separately and sequentially in the manuscript text

The Expanded View format, which will be displayed in the main HTML of the paper in a collapsible format, has replaced the Supplementary information. You can submit up to 5 images as Expanded View. Please follow the nomenclature Figure EV1, Figure EV2 etc. The figure legend for these should be included in the main manuscript document file in a section called Expanded View Figure Legends after the main Figure Legends section. Additional Supplementary material should be supplied as a single pdf file labeled Appendix. The Appendix should have page numbers and needs to include a table of content on the first page (with page numbers) and legends for all content. Please follow the nomenclature Appendix Figure Sx, Appendix Table Sx etc. throughout the text, and also label the figures and tables according to this nomenclature.

For more details please refer to our guide to authors:
https://link.springer.com/journal/44319/submission-guidelines#cms-Revised-submissions

Please consult our guide for figure preparation:
https://media.springernature.com/original/springer-cms/rest/v1/content/27825798/data/v1

3) a .docx formatted letter INCLUDING the reviewers' reports and your detailed point-by-point responses to their comments. As part of the EMBO Press transparent editorial process, the point-by-point response is part of the Review Process File (RPF), which will be published alongside your paper.

4) a complete author checklist, which you can download from our author guidelines (https://link.springer.com/journal/44319/submission-guidelines#cms-Revised-submissions). Please insert page numbers in the checklist to indicate where the requested information can be found in the manuscript. The completed author checklist will also be part of the RPF.

Please also follow our guidelines for the use of living organisms, and the respective reporting guidelines:
https://link.springer.com/partners/embo-press/editorial-policies#Animal%20and%20human%20studies

5) that primary datasets produced in this study (e.g. RNA-seq, ChIP-seq and array data) are deposited in an appropriate public database. This is now mandatory (like the COI statement). If no primary datasets have been deposited in any database, please

state this in this section (e.g. 'No primary datasets have been generated and deposited').

See also: https://link.springer.com/partners/embo-press/editorial-policies#Data%20deposition

Please remember to provide a reviewer password if the datasets are not yet public.

The accession numbers and database should be listed in a formal "Data Availability " section (placed after Materials & Methods) that follows the model below. Please note that the Data Availability Section is restricted to new primary data that are part of this study.

Data availability

The datasets produced in this study are available in the following databases:

- RNA-Seq data: Gene Expression Omnibus GSE46843 (https://www.ncbi.nlm.nih.gov/geo/query/acc.cgi?acc=GSE46843)
- [data type]: [name of the resource] [accession number/identifier/doi] ([URL or identifiers.org/DATABASE:ACCESSION])

*** Note - All links should resolve to a page where the data can be accessed. ***

Moreover, I have these editorial requests:

6) We now request the publication of original source data with the aim of making primary data more accessible and transparent to the reader. You will receive a separate email with instructions for providing source data with your revised manuscript, including information how to upload and organize the files.

7) Our journal encourages inclusion of *data citations in the reference list* to directly cite datasets that were re-used and obtained from public databases. Data citations in the article text are distinct from normal bibliographical citations and should directly link to the database records from which the data can be accessed. In the main text, data citations are formatted as follows: "Data ref: Smith et al, 2001" or "Data ref: NCBI Sequence Read Archive PRJNA342805, 2017". In the Reference list, data citations must be labeled with "[DATASET]". A data reference must provide the database name, accession number/identifiers and a resolvable link to the landing page from which the data can be accessed at the end of the reference. Further instructions are available at: https://link.springer.com/journal/44319/submission-guidelines#cms-Reference-guidelines

8) Regarding data quantification and statistics, please make sure that the number "n" for how many independent experiments were performed, their nature (biological versus technical replicates), the bars and error bars (e.g. SEM, SD) and the test used to calculate p-values is indicated in the respective figure legends (also for EV and Appendix figures). Please also check that all the p-values are explained in the legend, and that these fit to those shown in the figure. Please provide statistical testing where applicable. Please avoid the phrase 'independent experiment', but clearly state if these were biological or technical replicates. Please also indicate (e.g. with n.s.) if testing was performed, but the differences are not significant. In case n=2, please show the data as separate datapoints without error bars and statistics. See also:
https://link.springer.com/journal/44319/submission-guidelines#cms-Figure-and-data-presentation

If n<5, please show single datapoints for diagrams.

9) Please add scale bars of similar style and thickness to microscopic images, using clearly visible black or white bars (depending on the background). Please place these in the lower right corner of the images themselves. Please do not write on or near the bars in the image but define the size in the respective figure legend.

10) Please also note our reference format:
https://link.springer.com/journal/44319/submission-guidelines#cms-Reference-guidelines

11) We updated our journal's competing interests policy in January 2022 and request authors to consider both actual and perceived competing interests. Please name this section 'Disclosure and Competing Interests Statement' and put it after the Acknowledgements section.
See: https://link.springer.com/partners/embo-press/editorial-policies#Competing%20interest%20disclosures

12) We now use CRediT to specify the contributions of each author in the journal submission system. CRediT replaces the author contribution section. Please use the free text box to provide more detailed descriptions and do NOT provide your final manuscript text file with an author contributions section. See also our guide to authors (section 'Author contributions'):
https://link.springer.com/journal/44319/submission-guidelines#cms-Revised-submissions

13) All Materials and Methods need to be described in the main text using our 'Structured Methods' format, which is required for all research articles. According to this format, the Methods section should include a Reagents and Tools Table (listing key reagents, experimental models, software, and relevant equipment and including their sources and relevant identifiers), uploaded

as separate file, and a Methods section in which we encourage the authors to describe their methods using a step-by-step protocol format with bullet points, to facilitate the adoption of the methodologies across labs. More information on how to adhere to this format as well as downloadable templates (.doc) for the Reagents and Tools Table can be found in our author guidelines (section 'Structured Methods'):

https://link.springer.com/journal/44319/submission-guidelines#cms-Manuscript-organisation-and-formatting

Please add all primer and antibody information directly to the Reagents and Tools table.

14) Please add up to 5 keywords to the manuscript and order the sections like this, using these names:
Title page - Abstract (not more than 175 words) - Keywords - Introduction - Results - Discussion - Methods - Data availability section - Acknowledgements (please include here also the funding information) - Disclosure and Competing Interests Statement - References - Figure legends - Expanded View Figure legends

15) Please make sure that all the funding information is also entered into the online submission system and that it is complete and similar to the one in the acknowledgement section of the manuscript text file.

We would also welcome the submission of cover suggestions, or motifs to be used by our Graphics Illustrator in designing a cover.

As part of the EMBO publication's Transparent Editorial Process, EMBO reports publishes online a Review Process File (RPF) to accompany accepted manuscripts. This File will be published in conjunction with your paper and will include the referee reports, your point-by-point response and all pertinent correspondence relating to the manuscript.

You are able to opt out of this by letting the editorial office know (emboreports@embo.org). If you do opt out, the Review Process File link will point to the following statement: "No Review Process File is available with this article, as the authors have chosen not to make the review process public in this case."

I look forward to seeing a revised form of your manuscript when it is ready.

Kind regards,

Achim

Achim Breiling
Senior Editor
EMBO Reports

Referee #1:

The study aims to define important changes in protein expression in three different myopathies: DMD, OPMDI, and IBM. The authors used fibrotic myopathies to investigate their expression profile using proteomics and defined the expression of proteins specifically associated with the extracellular matrix. Many changes were found, which were unique to one of the myopathies; however, one feature present in all three diseases was an expression of collagen VI as a common disease-associated protein. Collagen VI is expressed mainly by FAP, and in a coculture experiment, FAPs isolated from patients suffering from each of the three myopathies caused impaired myogenic differentiation in co-culture experiments. Transplantation of FAPS in immunocompromised mice caused enhanced fibrosis and a loss of PAX7+ myogenic stem cells. Myogenic differentiation was rescued in the co-culture experiment with patient-derived FAPs after siRNA-mediated knockdown of Col VI.

Overall, the manuscript is well written, and the experiments are very well designed and executed. The study provides a novel insight into a common pathogenic mechanism in myopathies.

1. However, I am unable to see a significantly elevated expression of Collagen VI in cultured DMD-derived FAPs, which to me looked like control levels, yet the functional studies suggest that siRNA-mediated knockdown rescues myogenic differentiation in DMD-derived FAPS indistinguishable from OPMDI-derived FAPs. What is your explanation for this? I think it is advisable to perform some other form of quantitative assessment, such as a Western blot to quantify ColVI expression in FAP cultures.
2. In the discussion, a lot of the disease-specific alterations in protein expressions are described, which I believe should be all mentioned in the results section. Thus, the discussion needs to be rewritten and should therefore be refocussed on the difficulty to exploit the findings therapeutically, given the requirement for ColVI in muscle tissue.

Significance:
The strength of this manuscript is the unifying observation that Col VI seems to be a novel drug target to modulate disease severity and to enhance myogenic regeneration.

However, since ColVI is also required, as a loss is also associated with muscle disease, only a small therapeutic window will be present for most patients. Thus, at present, it is unclear how easy the observation could be therapeutically exploited. It will be interesting to see how generally the observation might be? An older report by the Campbell lab also reported that in sarcoglycan-associated limb-girdle muscular dystrophy collagen VI is elevated and that muscle pathology was reduced after crossing γ-sarcoglycan-null mutants with a Col6a2-deficient mouse; however that there was no gain in muscle function.

Unfortunately, the authors of the current study did not cite this previous study and did not test whether this previous observation would also apply to mouse models for each of the three human myopathies under study. I am not suggesting performing such a study, as it would essentially be a follow-up study. However, this important work needs to be cited and should also be discussed.

Referee #2:

In this study, the authors used liquid chromatography coupled with mass spectrometry to analyse three human myopathies (DMD, Duchenne Muscular Dystrophy; OPMD, Oculopharyngeal Muscular Dystrophy; IBM, Inclusion Body Myositis) to gain greater understanding of fibrosis as it is a major marker of disease progression and obstacle to muscle repair. The proteomics data revealed overlapping and distinct protein signatures between the three human myopathies. Through further approaches involving the transplantation of human fibro-adipogenic progenitor cells (FAPs) from dystrophic muscles in regenerating mouse muscles, the authors showed that FAPs produce ECM proteins, including Collagen VI, and contribute to the muscle ECM remodelling. Dystrophic FAPs are associated with a decline in the number of muscle stem cells and reduced fibre size, and Collagen VI is sufficient and necessary for FAP-mediated decrease in fusion index. Altogether, this manuscript reveals that FAPs are major drivers of the ECM remodelling underpinning fibrosis in human myopathies.

Major comments

1. My major comment relates to some conclusions which misrepresent slightly the complexity of the models and the data obtained.
An example of this is the title of the manuscript: 'Collagen VI is a conserved pro-fibrotic signal disrupting muscle regeneration across distinct human myopathies'. The manuscript showed that Collagen VI is upregulated in the three human myopathies examined but has not showed that it is a pro-fibrotic signal. The article showed that FAPs are pro-fibrotic, and separately showed that Collagen VI affects the fusion index and muscle stem cell numbers in a regenerating context.
The statement made in the introduction that FAPs are the key ECM producing cells in muscle ignores that other cell types can be major ECM producing cells in different circumstances, including macrophages, endothelial cells and muscle cells. Therefore, the proteomics data from whole human muscles cannot be directly attributed to FAPs only.
Also, the environment of fibrotic dystrophic muscles is distinct from that of regenerating muscles. So, I would encourage the authors to exercise caution in their conclusions as I am not sure that the effect of FAPs in one context translates well in another context.
2. Experiments carried out in Figs. 1 and 5 raise several questions. In the absence of a clear answer, it would be wise to moderate the interpretation of the findings. FAP from diseased muscles proliferate more rapidly than FAPs from healthy muscles. When co-cultured with muscle cells or transplanted in regenerating mouse muscles, do they invade the co-culture (or muscle) and prevent myoblasts (or muscle cells) to proliferate, interact with each other and fuse? Are FAPs affecting fusion because they affect differentiation or because cells die or stop proliferating? Or do they delay differentiation?
3. Could the authors interpret the distinct signature of the human myopathies in light of the fact that different muscles and patients of different ages were analysed?

Minor comments

1. In your Introduction, cite reference to support the statement that FAPs are the ECM producing cells in muscles. Report on studies showing the involvement of other cell types in ECM production.
2. A brief description of the ECM structure in muscles would be useful (interstitial and basal lamina)
3. It would be useful if the authors could add information about the sex of patients used in the analyses.
4. Figure 4: the text mentions that csa was calculated while the figure legend indicates that minimum ferret's analysis was carried out.
5. The experimental design for the muscle regeneration upon FAPs transplantation varies significantly between OPMD and DMD. What is the rationale for this?

Significance:
The strength of this study lies in the proteomics analyses carried out on human biopsies, which reveal a disruption in the balanced production of ECM components that may contribute to the high levels of fibrosis observed. The separate demonstration that FAPs synthesise ECM proteins and when co-cultured with myocytes, inhibit their differentiation suggest

strongly that they may be essential players in the excessive fibrosis observed in vivo in human myopathies. One limitation of this study was to test the role of FAPs in vivo in a muscle regeneration model, and not in a model of muscular dystrophy, as these two models are significantly different.

This study is of interest to scientists with interest in basic or clinical research in fibrosis and/or muscular dystrophies.

Referee #3:

In this study, Muraine et al. perform a comparative proteomic analysis of three distinct human myopathies characterized by fibrosis: Duchenne muscular dystrophy (DMD), oculopharyngeal muscular dystrophy (OPMD), and inclusion body myositis (IBM). Using liquid chromatography coupled to tandem mass spectrometry (LC-MS/MS), they compare fibrotic muscles from patients to those of age-matched healthy controls and identify matrisome alterations specific to each disease. The authors further isolate fibro-adipogenic progenitors (FAPs) from patient biopsies and characterize their secretion of key ECM proteins both in vitro and in vivo. Functional experiments demonstrate that FAP-secreted ECM components, including collagen VI and basal lamina proteins, modulate muscle stem cell behavior and tissue regenerative capacity. Notably, silencing COL6 in patient FAPs restores myogenic fusion, directly linking pathological collagen VI deposition to regeneration failure.
This work advances understanding of ECM remodeling in human muscle diseases and identifies collagen VI as a conserved effector of fibrosis and a potential therapeutic target across genetically and clinically distinct myopathies.

Major comments

Overall, the study is very well-designed, and the main conclusions are convincing. The comparative proteomic approach, combined with functional assays using patient-derived FAPs, provides novel and clinically relevant insights into ECM remodeling and its consequences on muscle regeneration. However, several points require clarification or additional data presentation to fully support the claims and improve reproducibility.

Figure 1A:

A global view of the muscle section is needed to visually confirm that approximately 60% of the tissue is fibrotic and 40% contains myocytes, as claimed. In its current form, the quantification does not appear to match the representative images shown.

Figure 1C:

Higher magnification is required, as individual FAPs are not discernible, only red signal is visible. Given the highly fibrotic context described, I strongly recommend including co-staining for CD45 and CD31 in addition to CD90. Fibrosis is known to induce phenotypic plasticity and atypical marker expression, including CD90 upregulation in endothelial and immune cells. Co-labeling with CD45 and CD31 is therefore important to exclude immune and endothelial contamination and confirm that CD90 indeed identifies FAPs. The scale of the graph should also be adjusted, as FAPs appear absent from control samples.

Figure 1E:

In murine models, FAPs are well-established regulators of myogenesis, supporting myoblast differentiation and fusion. It is therefore surprising that, in this study, human FAPs do not promote myotube formation. Although direct evidence for pro-myogenic effects of human FAPs is less extensive than in mice, this discrepancy should be discussed since it is to me a very interesting finding. Possible explanations might include differences in activation state, culture conditions, or communication with MuSCs. Clarifying this point would strengthen interpretation and contextualization within the current understanding of human versus murine FAP biology.

Figure 3F: Quantification relative to controls is needed to support the visual data and conclusions.

Figures 4 and 5: It is unclear why IBM and/or DMD FAPs are not included in these analyses. Given that the study title emphasizes comparison across DMD, OPMD, and IBM, inclusion (or at least an explanation for exclusion) of IBM and/or DMD samples is required.
Similarly, since LAMC1 is consistently upregulated across diseases, the rationale for focusing primarily on collagen VI should be better justified, or the contribution of LAMC1 should be discussed or data included.

Minor comments:

The authors of reference 15 need to be cited in the text : "To further delineate the cellular sources of ECM proteins, we analyzed a publicly available human single-cell RNA sequencing (scRNA-seq) dataset from 15"

Are the suggested experiments realistic in terms of time and resources? Yes, the proposed additions are feasible within a reasonable timeframe.

Are the data and methods presented in a way that allows reproduction? Yes, methods are clearly described, and data presentation is generally transparent.

Are experiments adequately replicated and is statistical analysis adequate? Yes, the replication and statistical approaches are appropriate.

Significance:

General assessment

This study provides a comprehensive proteomic and functional analysis of extracellular matrix (ECM) remodeling in three human myopathies characterized by fibrosis: DMD, OPMD, and IBM. Its main strength lies in its integrative design, combining human patient samples, quantitative proteomics, and FAP-derived ECM functional assays, to dissect disease-specific and shared fibrotic mechanisms. The identification of collagen VI as a common and functionally relevant effector of fibrosis across distinct genetic contexts represents a valuable contribution to the field. The use of primary human FAPs derived directly from patient biopsies also adds a high degree of translational relevance.

The main limitations are related to the incomplete inclusion of all disease groups in some analyses. Addressing these issues would further strengthen the reproducibility and generality of the conclusions.

Advance

To my knowledge, this is the first study to perform a comparative ECM proteomic profiling across multiple human myopathies while functionally linking FAP-derived collagen VI to defective myogenic regeneration. Previous work has implicated collagen VI accumulation in muscular dystrophies and in aging-related fibrosis, but its identification here as a shared effector across genetically distinct myopathies broadens its conceptual significance. The demonstration that silencing COL6 in human patient FAPs restores myotube fusion capacity provides direct mechanistic insight and suggests potential therapeutic avenues aimed at modulating FAP-ECM interactions. Thus, the study advances understanding of fibrosis not only in a disease-specific manner but also at a systems level, establishing a unifying ECM signature across fibrotic muscle disorders.

Audience

The study will primarily interest researchers in the fields of muscle biology, fibrosis, extracellular matrix biology, and stromal cell function, as well as those focusing on muscle regeneration and neuromuscular diseases. Its integrative human-based approach will also attract a translational and clinical audience, especially those exploring anti-fibrotic strategies or biomarker discovery. Beyond neuromuscular pathology, the identification of collagen VI as a conserved driver of fibrosis may be relevant to other fibrotic conditions where FAP-like cells contribute to ECM remodeling.

We would like to thank the reviewers for their time and expertise in offering their constructive criticisms on our manuscript. We provide below a point-by-point answer to all the comments and we are convinced that the revised manuscript taking into account the reviewer comments will further strengthens the clarity and impact of our conclusions.

Compared with the previous version submitted, we made changes throughout the manuscript to align to the EMBO Reports editorial requirements, including Figure 1G for which we have performed a new experiment of co-culture using IBM FAPs to increase the number of samples.

Reviewer #1 (Evidence, reproducibility and clarity (Required)):

The study aims to define important changes in protein expression in three different myopathies: DMD, OPMDI, and IBM. The authors used fibrotic myopathies to investigate their expression profile using proteomics and defined the expression of proteins specifically associated with the extracellular matrix. Many changes were found, which were unique to one of the myopathies; however, one feature present in all three diseases was an expression of collagen VI as a common disease-associated protein. Collagen VI is expressed mainly by FAP, and in a coculture experiment, FAPs isolated from patients suffering from each of the three myopathies caused impaired myogenic differentiation in co-culture experiments. Transplantation of FAPS in immunocompromised mice caused enhanced fibrosis and a loss of PAX7+ myogenic stem cells. Myogenic differentiation was rescued in the co-culture experiment with patient-derived FAPs after siRNA-mediated knockdown of Col VI.

Overall, the manuscript is well written, and the experiments are very well designed and executed. The study provides a novel insight into a common pathogenic mechanism in myopathies.

1. However, I am unable to see a significantly elevated expression of Collagen VI in cultured DMD-derived FAPs, which to me looked like control levels, yet the functional studies suggest that siRNA-mediated knockdown rescues myogenic differentiation in DMD-derived FAPS indistinguishable from OPMDI-derived FAPs. What is your explanation for this? I think it is advisable to perform some other form of quantitative assessment, such as a Western blot to quantify ColVI expression in FAP cultures.

The images in Figure 3F, showing immunostaining of ECM proteins produced by FAPs in vitro (including Collagen VI), were not intended to demonstrate quantitative differences in protein secretion between control and pathological FAPs. Rather, their purpose was to confirm that these cells are capable of producing the ECM components identified in our proteomic analysis.
Importantly, FAPs in these experiments were not cultured under the same conditions as those used for the co-culture assays. Specifically, Figure 3F corresponds to FAPs maintained for 10 days in growth medium, whereas co-culture experiments in presence of myoblasts were performed under differentiation conditions (5 days in serum-deprived medium).
For this reason, we do not believe that quantifying Collagen VI levels in FAPs monocultures alone would be sufficient to capture disease-relevant differences. Instead, we hypothesize that interactions with myotubes/muscle fibers are required to stimulate Collagen VI

production by FAPs. This is supported by our data in a regenerative context (Figure 5A), where human FAPs derived from OPMD and DMD patients exhibit increased Collagen VI expression compared to controls

2. In the discussion, a lot of the disease-specific alterations in protein expressions are described, which I believe should be all mentioned in the results section. Thus, the discussion needs to be rewritten and should therefore be refocussed on the difficulty to exploit the findings therapeutically, given the requirement for ColVI in muscle tissue.)

Thank you for the comment. We agree and have restructured the manuscript accordingly. We added more information on disease-specific protein expression changes in the Results section, and discussed the therapeutic challenges linked to the essential role of ColVI in muscle tissue.

Reviewer #1 (Significance (Required)):

The strength of this manuscript is the unifying observation that Col VI seems to be a novel drug target to modulate disease severity and to enhance myogenic regeneration. However, since ColVI is also required, as a loss is also associated with muscle disease, only a small therapeutic window will be present for most patients. Thus, at present, it is unclear how easy the observation could be therapeutically exploited. It will be interesting to see how generally the observation might be? An older report by the Campbell lab also reported that in sarcoglycan- associated limb-girdle muscular dystrophy collagen VI is elevated and that muscle pathology was reduced after crossing γ-sarcoglycan-null mutants with a Col6a2-deficient mouse; however that there was no gain in muscle function. Unfortunately, the authors of the current study did not cite this previous study and did not test whether this previous observation would also apply to mouse models for each of the three human myopathies under study. I am not suggesting performing such a study, as it would essentially be a follow-up study. However, this important work needs to be cited and should also be discussed.

We thank the reviewer for this observation and apologize for not including this very relevant reference in the initial version of our manuscript. We have incorporated it in the revised version and dedicated a section in the Discussion to address this point, as follows: “These findings raise important considerations regarding the therapeutic targeting of collagen VI. Notably, previous work in γ-sarcoglycan-deficient mice showed that reducing collagen VI ameliorates muscle pathology without improving muscle function (De Greef *et al*, 2016) Together with our data, this suggests that modulating collagen VI levels may not readily translate into functional benefit. Importantly, our identification of collagen VI as a shared alteration across the three myopathies studied here supports its relevance as a common disease-associated ECM component. However, therapeutic strategies will likely require precise tuning rather than simple inhibition, and their efficacy may still depend on disease context and stage”.

Reviewer #2 (Evidence, reproducibility and clarity (Required)):

Summary

In this study, the authors used liquid chromatography coupled with mass spectrometry to analyse three human myopathies (DMD, Duchenne Muscular Dystrophy; OPMD, Oculopharyngeal Muscular Dystrophy; IBM, Inclusion Body Myositis) to gain greater understanding of fibrosis as it is a major marker of disease progression and obstacle to muscle repair. The proteomics data revealed overlapping and distinct protein signatures between the three human myopathies. Through further approaches involving the transplantation of human fibro-adipogenic progenitor cells (FAPs) from dystrophic muscles in regenerating mouse muscles, the authors showed that FAPs produce ECM proteins, including Collagen VI, and contribute to the muscle ECM remodelling. Dystrophic FAPs are associated with a decline in the number of muscle stem cells and reduced fibre size, and Collagen VI is sufficient and necessary for FAP-mediated decrease in fusion index. Altogether, this manuscript reveals that FAPs are major drivers of the ECM remodelling underpinning fibrosis in human myopathies.

Major comments

1) My major comment relates to some conclusions which misrepresent slightly the complexity of the models and the data obtained.
An example of this is the title of the manuscript: 'Collagen VI is a conserved pro-fibrotic signal disrupting muscle regeneration across distinct human myopathies'. The manuscript showed that Collagen VI is upregulated in the three human myopathies examined but has not showed that it is a pro-fibrotic signal. The article showed that FAPs are pro-fibrotic, and separately showed that Collagen VI affects the fusion index and muscle stem cell numbers in a regenerating context.
The statement made in the introduction that FAPs are the key ECM producing cells in muscle ignores that other cell types can be major ECM producing cells in different circumstances, including macrophages, endothelial cells and muscle cells. Therefore, the proteomics data from whole human muscles cannot be directly attributed to FAPs only.
Also, the environment of fibrotic dystrophic muscles is distinct from that of regenerating muscles. So, I would encourage the authors to exercise caution in their conclusions as I am not sure that the effect of FAPs in one context translates well in another context.

We thank the reviewer for this comment and for highlighting important conceptual aspects regarding the interpretation of our data. To address the reviewer's pertinent remark concerning the conclusions and the title, we propose revising the current title ("Collagen VI is a conserved pro-fibrotic signal disrupting muscle regeneration across distinct human myopathies") to "Collagen VI is a fibrosis-associated signal disrupting muscle regeneration across distinct human myopathies" to more accurately reflect the scope of our findings, emphasizing fibrosis-associated remodeling and the contribution of Collagen VI, without implying that Collagen VI alone is sufficient to drive fibrosis.

Following the reviewer's comment, with which we fully agree, we have revised the sentence in the introduction (*"ECM proteins are secreted by a variety of cells within muscle; muscle stem cells (MuSCs), macrophages, endothelial cells, fibroadipogenic progenitors (FAPs) (Baghdadi et al, 2018; Schüler et al, 2022; Gratchev et al, 2001; Schnoor et al, 2008; Urciuolo et al, 2013). Among those, FAPs are the most prominent source of ECM proteins during homeostasis and regeneration (Zou et al, 2008; Uezumi et al, 2011; Chapman et al, 2017; Archile-Contreras et al, 2010;* Kühl *et al*, 1984; Gatchalian *et al*, 1989*)"* to acknowledge the contribution of other cell types to ECM production as well as added a sentence in the

discussion (*"FAPs are often considered as the major producers of ECM components in skeletal muscle although they are not the only ECM-secreting cells within muscle (Zou et al, 2008; Uezumi et al, 2011). During regeneration, other cell types like MuSCs, macrophages or endothelial cells have been shown to secrete ECM components (Baghdadi et al, 2018; Schüler et al, 2022; Gratchev et al, 2001; Schnoor et al, 2008; Urciuolo et al, 2013)")*

As the reviewer pointed out, we did not mean to assert that the proteomics data from whole human muscles could be directly attributed to FAPs only, but rather that the proteins found dysregulated could indeed be secreted by FAPs in vitro and could therefore act in the communication defect observed in our co culture experiments.

We again agree with the reviewer that fibrotic dystrophic muscle differs substantially from regenerating muscle. In our study, we used a regenerative model to mimic muscle regeneration and to investigate how FAPs behave during ongoing regeneration and how they influence the satellite cell niche and myofiber formation. Our aim was not to treat as equivalent regenerating and chronically fibrotic environments, but rather to explore how fibrosis-associated cues, identified in human myopathies, can impact the regenerative process. The reviewer's insightful comment indeed provides an excellent basis for future investigations aimed at elucidating FAP behavior within a murine fibrotic environment. Such studies would require the use of specific mouse models that recapitulate the genetic background, the extent of muscle fibrosis, and the disease-specific signals of each of the three pathologies studied, combined with an immunodeficient background to allow engraftment of human cells. While beyond the scope of the present work, this represents an important direction for future studies.

2) Experiments carried out in Figs. 1 and 5 raise several questions. In the absence of a clear answer, it would be wise to moderate the interpretation of the findings. FAP from diseased muscles proliferate more rapidly than FAPs from healthy muscles. When co-cultured with muscle cells or transplanted in regenerating mouse muscles, do they invade the co-culture (or muscle) and prevent myoblasts (or muscle cells) to proliferate, interact with each other and fuse? Are FAPs affecting fusion because they affect differentiation or because cells die or stop proliferating? Or do they delay differentiation?

We thank the reviewer for raising this important point. Figures 1 and 5 were designed to assess how FAPs influence myoblast fusion and differentiation using co-culture assays, with fusion index as a quantitative readout.
We performed additional analyses to clarify the mechanisms underlying the observed effects.
First, although fibrotic FAPs display increased proliferative activity in growth medium, this does not lead to overgrowth or invasion of the co-culture. As shown in Fig. 1F, FAPs (desmin-negative/DAPI-positive cells) remain a minority population and do not colonize the culture dish. Moreover, based on our previous study (Bensalah et al., JCSM, 2022, Fig. 3B), fibrotic FAPs are smaller in size, further arguing against a physical barrier impairing myoblast–myoblast interactions.
Second, EdU incorporation assays indicate that myoblast proliferation is not affected by the presence of FAPs, excluding the possibility that reduced fusion results from impaired myoblast proliferation (see figure below).

Figure for referee with unpublished data and its description has been removed upon request by the authors.

Third, we did not observe obvious signs of increased cell death in co-culture conditions (see figure below), suggesting that the reduced fusion is not due to loss of myoblast viability.
Taken together, these results indicate that fibrotic FAPs do not impair myoblast fusion by outcompeting them, inhibiting their proliferation, or inducing cell death. Instead, our data support the idea that FAPs interfere with the myogenic program itself, likely by affecting the differentiation process and/or delaying the transition from proliferation to fusion. The primary readout used throughout this study is the fusion index of myoblasts differentiating into myotubes. To address the reviewer's comment, we have carefully revised the manuscript to ensure consistent terminology, referring only to increases or decreases in the fusion index and avoiding confusion with related concepts such as delays in fusion and delay in differentiation, (that could be one possibility).

Figure for referee with unpublished data and its description has been removed upon request by the authors.

3) Could the authors interpret the distinct signature of the human myopathies in light of the fact that different muscles and patients of different ages were analysed?

Thank you for pointing out this important discussion point. The muscles for each pathology were compared to the same type of muscle from an age-matched, unaffected subject (for example, cricopharyngeal muscle from an OPMD patient compared with cricopharyngeal muscle from a control subject) in order to identify differences between normal and pathological conditions, without introducing potential variability linked to muscle specificity. We believe that the proteins we identified for each condition correspond to those expressed at a very specific stage of the disease. For example, it is possible that the increased protein levels observed in DMD muscle around 14 years of age would differ from those that might be found if the same analysis were performed on muscle from a 20-year-old patient. We have carefully integrated all these elements of discussion in the revised version of our manuscript in the discussion session ("To minimize muscle-specific variability, each condition was compared to the corresponding muscle type from age-matched unaffected individuals, enabling the identification of robust disease-associated proteomic changes within fibrotic environment.").

Minor comments

1) In your Introduction, cite reference to support the statement that FAPs are the ECM producing cells in muscles. Report on studies showing the involvement of other cell types in ECM production.

We have now integrated in the introduction references to support the statement that FAPs are ECM producing cells within the muscles and acknowledge the involvement of other cell types in ECM production, as follows: *ECM proteins are secreted by a variety of cells within muscle: muscle stem cells (MuSCs), macrophages, endothelial cells, and fibroadipogenic progenitors (FAPs) (Baghdadi et al, 2018; Schüler et al, 2022; Gratchev et al, 2001; Schnoor et al, 2008; Urciuolo et al, 2013).*

2) A brief description of the ECM structure in muscles would be useful (interstitial and basal lamina)

We added a brief description of the ECM structure in the introduction of the manuscript to present basal lamina and interstitial ECM, as follows: "In skeletal muscle, the ECM surrounding each muscle fiber is defined as the endomysium. This connective tissue can be divided into two distinct yet interconnected compartments: the basal lamina and the interstitial ECM. the basal lamina is directly linked to the sarcolemma of each fiber surrounds sublaminar MuSCs. It is connected, through a collagen VI network, to a reticular scaffold of interstitial ECM rich in collagen I, and III (Sanes et al 2003). The endomysium is highly deformable and its composition undergoes dynamic remodeling during regeneration process".

3) It would be useful if the authors could add information about the sex of patients used in the analyses.

We added information regarding the sex of the patients in the material and methods section

4) Figure 4: the text mentions that csa was calculated while the figure legend indicates that minimum ferret's analysis was carried out.

We would like to thank the reviewer for pointing this out. The mistake has been corrected in the text: minimal feret's diameter was determined to carry out the analysis.

5) The experimental design for the muscle regeneration upon FAPs transplantation varies significantly between OPMD and DMD. What is the rationale for this?

We thank the reviewer for this comment and would like to clarify a potential misunderstanding. In our study, OPMD and DMD conditions are not directly compared to each other, but each is analyzed independently relative to its respective control condition. Therefore, differences in the experimental design between these models do not affect the interpretation of our results.

Our laboratory has extensive experience with human cell transplantation following muscle regeneration in the tibialis anterior of immunodeficient mice, using two different injury models, cryodamage and notexin injection. Based on our previous work, these approaches yield comparable engraftment outcomes, supporting their validity as alternative methods to induce muscle regeneration. The use of notexin injection instead of cryodamage in certain experiments reflects a methodological optimization. Notexin induces a homogeneous muscle injury while avoiding invasive surgery. In addition, using a single injection instead of multiple interventions simplifies the procedure and aligns with the the 3R principle by reducing animal suffering and the number of manipulations.
Taken together, although different injury models were used, each experimental condition is internally controlled, and this does not impact the conclusions of the study.

Reviewer #2 (Significance (Required)):

The strength of this study lies in the proteomics analyses carried out on human biopsies, which reveal a disruption in the balanced production of ECM components that may contribute to the high levels of fibrosis observed. The separate demonstration that FAPs synthesise ECM proteins and when co-cultured with myocytes, inhibit their differentiation suggest strongly that they may be essential players in the excessive fibrosis observed in vivo in human myopathies. One limitation of this study was to test the role of FAPs in vivo in a muscle regeneration model, and not in a model of muscular dystrophy, as these two models are significantly different.
This study is of interest to scientists with interest in basic or clinical research in fibrosis and/or muscular dystrophies. This reviewer has expertise in muscle regeneration and skeletal muscle stem cell biology.

Reviewer #3 (Evidence, reproducibility and clarity (Required)):

A-Summary
In this study, Muraine et al. perform a comparative proteomic analysis of three distinct human myopathies characterized by fibrosis: Duchenne muscular dystrophy (DMD), oculopharyngeal muscular dystrophy (OPMD), and inclusion body myositis (IBM). Using liquid chromatography coupled to tandem mass spectrometry (LC-MS/MS), they compare fibrotic muscles from patients to those of age-matched healthy controls and identify

matrisome alterations specific to each disease. The authors further isolate fibro-adipogenic progenitors (FAPs) from patient biopsies and characterize their secretion of key ECM proteins both in vitro and in vivo. Functional experiments demonstrate that FAP-secreted ECM components, including collagen VI and basal lamina proteins, modulate muscle stem cell behavior and tissue regenerative capacity. Notably, silencing COL6 in patient FAPs restores myogenic fusion, directly linking pathological collagen VI deposition to regeneration failure.

This work advances understanding of ECM remodeling in human muscle diseases and identifies collagen VI as a conserved effector of fibrosis and a potential therapeutic target across genetically and clinically distinct myopathies.

B-Major comments

Overall, the study is very well-designed, and the main conclusions are convincing. The comparative proteomic approach, combined with functional assays using patient-derived FAPs, provides novel and clinically relevant insights into ECM remodeling and its consequences on muscle regeneration. However, several points require clarification or additional data presentation to fully support the claims and improve reproducibility.

Figure 1A:

A global view of the muscle section is needed to visually confirm that approximately 60% of the tissue is fibrotic and 40% contains myocytes, as claimed. In its current form, the quantification does not appear to match the representative images shown.

We thank the reviewer for this comment. The image shown in Fig. 1A corresponds to a representative field and does not capture the entire muscle section, which may explain the perceived discrepancy with the quantification.

To address this point, we have included additional low-magnification images (Appendix Fig S1), showing the full muscle section in the supplementary data, in order to provide a global view of the Sirius Red staining and better illustrate the relative proportions of fibrotic and myofiber areas.

Figure for referee with unpublished data and its description has been removed upon request by the authors.

Figure 1C:

Higher magnification is required, as individual FAPs are not discernible, only red signal is visible.

To address this point, we have included additional higher-magnification images in the supplementary data (Appendix Fig S2) to better discriminate FAPs stained with CD90 antibody, within the muscle.

Figure for referee with unpublished data and its description has been removed upon request by the authors.

Given the highly fibrotic context described, I strongly recommend including co-staining for CD45 and CD31 in addition to CD90. Fibrosis is known to induce phenotypic plasticity and atypical marker expression, including CD90 upregulation in endothelial and immune cells. Co-labeling with CD45 and CD31 is therefore important to exclude immune and endothelial contamination and confirm that CD90 indeed identifies FAPs. The scale of the graph should also be adjusted, as FAPs appear absent from control samples.

We agree with the reviewer that the dystrophic environment can induce atypical marker expression. We chose to use CD90 staining as PDGFRa antibodies did not perform well in fibrotic human biopsies. When performing the CD90 staining, we also applied a laminin antibody in order to precisely discriminate the muscle architecture. This staining allowed us to clearly identify the endothelial cells around vessels, counterstained with laminin, and thereby to exclude them from our CD90 quantification.
We have repeated the CD90 staining using a CD45 antibody to exclude potential immune cells and could not find any CD90+ cells positive for CD45 in any of the pathologies, as illustrated below in a IBM biopsy.
If the reviewer finds it relevant, we can provide these images as supplementary data in the revised version of our manuscript.

Figure for referee with unpublished data and its description has been removed upon request by the authors.

Immunostaining of an IBM human skeletal muscle biopsy showing CD90 (red) CD45 (green) and Hoechst stainings. Scale: 20μm

We have also adjusted the scale of the graphs in fig1C as requested by the reviewer, and a copy of the new graph is provided below.

Figure for referee with unpublished data and its description has been removed upon request by the authors.

Figure 1E:
In murine models, FAPs are well-established regulators of myogenesis, supporting myoblast differentiation and fusion. It is therefore surprising that, in this study, human FAPs do not promote myotube formation. Although direct evidence for pro-myogenic effects of human FAPs is less extensive than in mice, this discrepancy should be discussed since it is to me a very interesting finding. Possible explanations might include differences in activation state, culture conditions, or communication with MuSCs. Clarifying this point would strengthen interpretation and contextualization within the current understanding of human versus murine FAP biology.

We agree with the referee that this divergent behavior between human and murine FAPs deserves further discussion. We have addressed this point in the discussion section, by considering possible explanations, already suggested by the reviewer, which may include differences in activation state (homeostasis/regenerating conditions vs fibrotic condition, culture conditions, different ratio between MuSCs and FAPs during co-culture). The following paragraph has been added in the discussion section: “Our results show a deleterious impact from disease-derived FAPs on MuSCs, revealed by a decrease of myoblasts fusion index *in vitro*, while no effect is visible in the presence of control FAPs. FAPs are essential to muscle

and their absence leads to altered regeneration in mouse (Wosczyna *et al*, 2019). In mice, FAPs have been shown to promote myogenesis (Joe *et al*, 2010; Yu *et al*, 2024). However, some studies do not observe a positive effect of FAPs on myoblasts differentiation and fusion (Mázala *et al*, 2023). Interestingly, we did not observe a positive effect on myoblasts fusion when co cultured with FAPs isolated from control muscles. These discrepancies between studies could be explained by species differences, variations in culture conditions including the medium used, the duration of differentiation, the ratio of each cell types in co culture or the activation state of the FAPs."

Figure 3F: Quantification relative to controls is needed to support the visual data and conclusions.

As mentioned for Reviewer #2, the images in Figure 3F, showing immunostaining of ECM proteins produced by FAPs in vitro, including Collagen VI, were not intended to demonstrate differences in protein secretion between control and pathological FAPs, but rather to confirm that these cells are capable of producing the proteins identified in our proteomic analysis. Furthermore, we believe that simply quantifying this protein in FAPs cultured alone may not adequately reflect their capacity to produce Collagen VI: co-culture with myotubes might be required to elicit a more robust production.

Figures 4 and 5: It is unclear why IBM and/or DMD FAPs are not included in these analyses. Given that the study title emphasizes comparison across DMD, OPMD, and IBM, inclusion (or at least an explanation for exclusion) of IBM and/or DMD samples is required.

We thank the reviewer for this comment. We would like to clarify that the comparative analysis across DMD, OPMD, and IBM FAPs constitutes the core of the study and is presented throughout the manuscript.
The *in vivo* and siRNA-based mechanistic experiments shown in Figures 4 and 5 were designed as proof-of-principle experiments to demonstrate the functional role of COL6, rather than to systematically re-test all three pathologies. Performing these experiments in all three conditions was not technically feasible, as they are particularly demanding in terms of material, time, and in vivo manipulation.
We therefore chose for the *in vivo* part, two conditions that are sufficiently distinct—DMD and OPMD. OPMD and IBM FAPs, both isolated from the cricopharyngeal muscle of aged patients, are largely overlapping. By choosing more distinct conditions, we aimed to demonstrate that the results can be consistently reproduced. We selected a single pathology as a representative example for the siRNA to illustrate the mechanism. Based on the strong similarities observed across the phenotype of DMD, OPMD, and IBM FAPs in our comparative analyses, we propose that this COL6-dependent mechanism is likely shared among the three diseases, although we acknowledge that this was not experimentally tested in all cases. The following sentence has been added: "As FAPs isolated from the three pathologies present similar in vitro characteristic, we decided as a proof of principle to use FAPs from OPMD patients").

Similarly, since LAMC1 is consistently upregulated across diseases, the rationale for focusing primarily on collagen VI should be better justified, or the contribution of LAMC1 should be discussed or data included.

We provided the rationale that motivated our choice of focusing on collagen VI in the discussion. We are of course aware that collagen VI is not the only molecule that may play a role; however, we decided to focus our functional study on this molecule because all three chains forming the complete collagen VI protein were found among the increased and shared proteins. LAMC1represents only one subunit of the heterotrimeric protein. However, LAMC1 and HSPG2 are possibly pivotal in the fibrotic process and interesting candidates and should be investigated in future studies.

C-Minor comments:
The authors of reference 15 need to be cited in the text : "To further delineate the cellular sources of ECM proteins, we analyzed a publicly available human single-cell RNA sequencing (scRNA-seq) dataset from 15"

We thank the reviewer for pointing that out and cited the reference in the text as a data citation following editorial recommendations.

D-Are the suggested experiments realistic in terms of time and resources? Yes, the proposed additions are feasible within a reasonable timeframe.

E-Are the data and methods presented in a way that allows reproduction? Yes, methods are clearly described, and data presentation is generally transparent.

F-Are experiments adequately replicated and is statistical analysis adequate? Yes, the replication and statistical approaches are appropriate.

Reviewer #3 (Significance (Required)):

General assessment
This study provides a comprehensive proteomic and functional analysis of extracellular matrix (ECM) remodeling in three human myopathies characterized by fibrosis: DMD, OPMD, and IBM. Its main strength lies in its integrative design, combining human patient samples, quantitative proteomics, and FAP-derived ECM functional assays, to dissect disease-specific and shared fibrotic mechanisms. The identification of collagen VI as a common and functionally relevant effector of fibrosis across distinct genetic contexts represents a valuable contribution to the field. The use of primary human FAPs derived directly from patient biopsies also adds a high degree of translational relevance.
The main limitations are related to the incomplete inclusion of all disease groups in some analyses. Addressing these issues would further strengthen the reproducibility and generality of the conclusions.

Advance
To my knowledge, this is the first study to perform a comparative ECM proteomic profiling across multiple human myopathies while functionally linking FAP-derived collagen VI to defective myogenic regeneration. Previous work has implicated collagen VI accumulation in muscular dystrophies and in aging-related fibrosis, but its identification here as a shared effector across genetically distinct myopathies broadens its conceptual significance. The

demonstration that silencing COL6 in human patient FAPs restores myotube fusion capacity provides direct mechanistic insight and suggests potential therapeutic avenues aimed at modulating FAP-ECM interactions. Thus, the study advances understanding of fibrosis not only in a disease-specific manner but also at a systems level, establishing a unifying ECM signature across fibrotic muscle disorders.

Audience

The study will primarily interest researchers in the fields of muscle biology, fibrosis, extracellular matrix biology, and stromal cell function, as well as those focusing on muscle regeneration and neuromuscular diseases. Its integrative human-based approach will also attract a translational and clinical audience, especially those exploring anti-fibrotic strategies or biomarker discovery. Beyond neuromuscular pathology, the identification of collagen VI as a conserved driver of fibrosis may be relevant to other fibrotic conditions where FAP-like cells contribute to ECM remodeling.

Field of expertise: Muscle regeneration, mesenchymal stromal cells, adipose biology, connective tissue, cell migration.

Areas outside my primary expertise: Advanced LC-MS/MS analytical pipelines and proteomic normalization methods.

Dear Dr. Negroni,

Thank you for the submission of your revised manuscript to our editorial offices. I have now received the reports from the three referees that were asked to re-evaluate the study, you will find below. As you will see, the referees now fully support the publication of the study in EMBO reports.

Before we can proceed with formal acceptance, I have the editorial requests below I ask you to address in a final revised manuscript. Please also provide a final p-b-p-response to the editorial requests.

Editorial requests:

- Please provide the abstract written in present tense throughout.

- Please make sure the final manuscript text file is ordered like this, using only these names:
Title page - Abstract - Keywords - Introduction - Results - Discussion - Methods - Data availability section - Acknowledgements - Disclosure and Competing Interests Statement - References - Figure legends

- In order to render the presentation more comprehensive, I suggest moving all the tables presently in the main manuscript text to the Appendix. Please do that, using the nomenclature 'Appendix Table Sx', starting with table S1. Please do only use full numberings. There can't be e.g. tables 4A or 4B. The numbering of Appendix figures and tables is independent. I.e., you should start with Appendix Table S1, then Appendix Table S2 and so on. Please move all the tables presently in the manuscript text files and the corresponding legends to the Appendix (and remove them from the final manuscript text file), and combine these with the tables already present there. Finally, please update all callouts.

- Please add the manuscript title to the Appendix cover page (Appendix for "...").

- Please add the antibody and primer information (presently Appendix Tables S4 and S5) directly to the Reagents and Tools Table and remove these from the Appendix. Please update their callouts ('see Reagents & Tools table') and also remove the instructions section from the Reagents & Tools Table.

- Please check again that the number "n" for how many independent experiments were performed, their nature (biological versus technical replicates), the bars and error bars (e.g. SEM, SD) and the test used to calculate p-values is indicated in the respective figure legends (main and EV figures). Please also check that all the p-values are explained in the legend, and that this fit to those shown in the figure. Please provide statistical testing where applicable. Please avoid the phrase 'independent experiment' but clearly state if these were biological or technical replicates. Please also indicate (e.g. with n.s.) if testing was performed, but the differences are not significant. In case n=2, please show the data as separate datapoints without error bars and statistics. See also:

https://link.springer.com/journal/44319/submission-guidelines#cms-Figure-and-data-presentation

If n<5, please show single datapoints for diagrams. Moreover:
- Please note that the exact p values are not provided in the legends of figures 1A, B, C, D, G; 4B, C; 5A, C, E, F
- Please indicate the statistical test used for data analysis in the legends of figures 2B, 5A, C, E, F
- Please note that information related to n is missing in the legends of figures 5B, E
- Please note that the error bars are not defined in the legends of figures 5A, C, E, F

- The data availability section is restricted to information on externally deposited datasets. Please remove any other text from this section.

- Please list both corresponding authors in the author checklist header.

- Please add a full citation for the protocol.io mentioned on page 24 and include it in the references.

- Please add scale bars of similar style and thickness to all microscopic images, using clearly visible black or white bars (depending on the background). Please place these in the lower right corner of the images themselves. Please do not write on or near the bars in the image but define the size in the respective figure legend. Presently, many scale bars are rather thin. Please improve.

- We noted several reuses of images between panel 1A and Appendix Figure S2, which seems to be intentional. Please clearly indicate in the respective figure legends that images have been reused or show enlargements/subsets of panels shown in the respective other panel.

Moreover, it seems Figure 5E has been edited or is a composite. Please provide source data for this figure and explain how it was obtained/created.

In addition, I would need from you uploaded separately:
- a short, two-sentence summary of the manuscript (not more than 35 words).
- two to four short (!) bullet points highlighting the key findings of your study (two lines each).
- a schematic summary figure as separate file that provides a sketch of the major findings (not a data image) in jpeg or tiff format (with the exact width of 550 pixels and the exact height of 300 pixels) that can be used as a visual synopsis on our website.

I look forward to seeing the further revised version of your manuscript when it is ready. Please let me know if you have questions regarding the revision.

Best,

Achim Breiling
Senior Editor
EMBO Reports

Referee #1:

The manuscript has been previously reviewed by me for EMBO Journal and has now been resubmitted after revision to EMBO Reports. The authors have satisfactorily addressed my comments and introduced the requested changes to the manuscript.

Referee #2:

I would like to thank the authors for their thorough and constructive point-by-point response and for the significant efforts made to revise the manuscript.
Overall, the authors have addressed the major concerns raised in the initial review in a satisfactory manner. The addition of supplementary data, particularly the inclusion of low- and high-magnification images (Appendix Figures S1 and S2), substantially improves the clarity and interpretability of the histological and cellular analyses. The clarification regarding CD90 specificity, including additional CD45 co-staining, is appropriate and strengthens confidence in the identification of FAP populations.

Importantly, the authors have taken care to moderate some of their initial claims, especially regarding the role of collagen VI, and have refined the title and discussion accordingly. These revisions better reflect the complexity of ECM remodeling and avoid overinterpretation. The expanded discussion on the differences between human and murine FAP behavior, as well as the limitations related to the use of regeneration models versus chronic disease contexts, is particularly welcome and improves the overall conceptual framing of the study.The additional explanations provided regarding experimental design choices-such as the selective use of certain disease conditions in mechanistic experiments are reasonable and adequately justified given the technical constraints. While the inclusion of all disease groups in every assay would further strengthen the generality of the conclusions, I agree that the current data provide a solid proof-of-principle.

Overall, the manuscript is now clearer, more balanced, and methodologically sound. The study provides important and novel insights into ECM remodeling across human myopathies and highlights collagen VI as a shared and functionally relevant component of the fibrotic environment.

In its revised form, I consider the manuscript suitable for publication.

Referee #3:

This manuscript has now been revised by the authors who have taken into account my recommendations and comments and produced a detailed study accompanied by a thorough analysis; I am therefore happy to accept the manuscript for publication.

All editorial requests have been addressed by the authors.

Dr. Elisa Negroni
Sorbonne Université, INSERM, Association Institut de Myologie,
Centre de Recherche en Myologie, UMRS974
47 bd de l'Hôpital
Paris 75013
France

Dear Dr. Negroni,

I am very pleased to accept your manuscript for publication in the next available issue of EMBO reports. Thank you for your contribution to our journal.

Your manuscript will be processed for publication by EMBO Press. It will be copy edited and you will receive page proofs prior to publication. Please note that you will be contacted by Springer Nature Author Services to complete licensing and payment information.

You may qualify for financial assistance for your publication charges - either via a Springer Nature fully open access agreement or an EMBO initiative. Check your eligibility: https://link.springer.com/journal/44319/how-to-publish-with-us

Should you be planning a Press Release on your article, please notify the production team via this form in order to coordinate publication and release dates: https://forms.cloud.microsoft/e/eH4A8JVP6g

If you have any questions, please do not hesitate to contact the Editorial Office. Thank you for your contribution to EMBO Reports.

Yours sincerely,

Achim Breiling
Senior Editor
EMBO Reports

>>> Please note that it is EMBO Reports policy for the transcript of the editorial process (containing referee reports and your response letter) to be published as an online supplement to each paper. If you do NOT want this, you will need to inform the Editorial Office via email immediately. More information is available here: https://link.springer.com/partners/embo-press/editorial-policies#Peer%20review

EMBO Press Author Checklist

Corresponding Author Name: Elisa Negroni, Capucine Trollet	[The EMBO Journal - Author Guidelines](#)<br>[EMBO Reports - Author Guidelines](#)<br>[Molecular Systems Biology - Author Guidelines](#)<br>[EMBO Molecular Medicine - Author Guidelines](#)
Journal Submitted to: EMBO Reports	
Manuscript Number: EMBOR-2025-63478V1-T	

Reporting Checklist for Life Science Articles (updated January
This checklist is adapted from Materials Design Analysis Reporting (MDAR) Checklist for Authors. MDAR establishes a minimum set of requirements in transparent reporting in the life sciences (see Statement of Task: 10.31222/osf.io/9sm4x). Please follow the journal's guidelines in preparing your
Please note that a copy of this checklist will be published alongside your article.

Abridged guidelines for figures
1. Data
The data shown in figures should satisfy the following conditions:
- the data were obtained and processed according to the field's best practice and are presented to reflect the results of the experiments in an accurate and unbiased manner.
- ideally, figure panels should include only measurements that are directly comparable to each other and obtained with the same assay.
- plots include clearly labeled error bars for independent experiments and sample sizes. Unless justified, error bars should not be shown for technical
- if n<5, the individual data points from each experiment should be plotted. Any statistical test employed should be justified.
- Source Data should be included to report the data underlying figures according to the guidelines set out in the authorship guidelines on Data

2. Captions
Each figure caption should contain the following information, for each panel where they are relevant:
- a specification of the experimental system investigated (eg cell line, species name).
- the assay(s) and method(s) used to carry out the reported observations and measurements.
- an explicit mention of the biological and chemical entity(ies) that are being measured.
- an explicit mention of the biological and chemical entity(ies) that are altered/varied/perturbed in a controlled manner.
- the exact sample size (n) for each experimental group/condition, given as a number, not a range;
- a description of the sample collection allowing the reader to understand whether the samples represent technical or biological replicates (including how many animals, litters, cultures, etc.).
- a statement of how many times the experiment shown was independently replicated in the laboratory.
- definitions of statistical methods and measures:
  - common tests, such as t-test (please specify whether paired vs. unpaired), simple χ2 tests, Wilcoxon and Mann-Whitney tests, can be unambiguously identified by name only, but more complex techniques should be described in the methods section;
  - are tests one-sided or two-sided?
  - are there adjustments for multiple comparisons?
  - exact statistical test results, e.g., P values = x but not P values < x;
  - definition of 'center values' as median or average;
  - definition of error bars as s.d. or s.e.m.

Please complete ALL of the questions below.
Select "Not Applicable" only when the requested information is not relevant for your study.

Materials

Newly Created Materials	Information included in the manuscript?	In which section is the information available? (Reagents and Tools Table, Materials and Methods, Figures, Data Availability Section)
New materials and reagents need to be available; do any restrictions apply?	Yes	Reagents and Tools Table

Antibodies	Information included in the manuscript?	In which section is the information available? (Reagents and Tools Table, Materials and Methods, Figures, Data Availability Section)
For **antibodies** provide the following information:<br>- Commercial antibodies: RRID (if possible) or supplier name, catalogue number and or/clone number<br>- Non-commercial: RRID or citation	Yes	Reagents and Tools Table

DNA and RNA sequences	Information included in the manuscript?	In which section is the information available? (Reagents and Tools Table, Materials and Methods, Figures, Data Availability Section)
**Short novel DNA or RNA including primers, probes**: provide the sequences.	Yes	Reagents and Tools Table

Cell materials	Information included in the manuscript?	In which section is the information available? (Reagents and Tools Table, Materials and Methods, Figures, Data Availability Section)
**Cell lines:** Provide species information, strain. Provide accession number in repository **OR** supplier name, catalog number, clone number, and/**OR** RRID.	Not Applicable	
**Primary cultures:** Provide species, strain, sex of origin, genetic modification status.	Yes	Material and Methods
Report if the cell lines were recently **authenticated** (e.g., by STR profiling) and tested for mycoplasma contamination.	Yes	Material and Methods

Experimental animals	Information included in the manuscript?	In which section is the information available? (Reagents and Tools Table, Materials and Methods, Figures, Data Availability Section)
**Laboratory animals or Model organisms:** Provide species, strain, sex, age, genetic modification status. Provide accession number in repository **OR** supplier name, catalog number, clone number, **OR** RRID.	Yes	Material and methods
**Animal observed in or captured from the field:** Provide species, sex, and age where possible.	Not Applicable	
Please detail **housing and husbandry conditions**.	Yes	Material and methods

Plants and microbes	Information included in the manuscript?	In which section is the information available? (Reagents and Tools Table, Materials and Methods, Figures, Data Availability Section)
**Plants:** provide species and strain, ecotype and cultivar where relevant, unique accession number if available, and source (including location for collected wild specimens).	Not Applicable	
**Microbes:** provide species and strain, unique accession number if available, and source.	Not Applicable	

Human research participants	Information included in the manuscript?	In which section is the information available? (Reagents and Tools Table, Materials and Methods, Figures, Data Availability Section)
If collected and within the bounds of privacy constraints report on age, sex and gender or ethnicity for all study participants.	Yes	Materials and Methods

Core facilities	Information included in the manuscript?	In which section is the information available? (Reagents and Tools Table, Materials and Methods, Figures, Data Availability Section)
If your work benefited from core facilities, was their service mentioned in the acknowledgments section?	Yes	Acknowledgments

Design

Study protocol	Information included in the manuscript?	In which section is the information available? (Reagents and Tools Table, Materials and Methods, Figures, Data Availability Section)
If study protocol has been **pre-registered, provide DOI in the manuscript**. For clinical trials, provide the trial registration number **OR** cite DOI.	Not Applicable	
Report the **clinical trial registration number** (at ClinicalTrials.gov or equivalent), where applicable.	Not Applicable	

Laboratory protocol	Information included in the manuscript?	In which section is the information available? (Reagents and Tools Table, Materials and Methods, Figures, Data Availability Section)
Provide DOI OR other citation details if **external detailed step-by-step protocols** are available.	Not Applicable	

Experimental study design and statistics	Information included in the manuscript?	In which section is the information available? (Reagents and Tools Table, Materials and Methods, Figures, Data Availability Section)
Include a statement about **sample size** estimate even if no statistical methods were used.	Yes	Material and Methods
Were any steps taken to minimize the effects of subjective bias when allocating animals/samples to treatment (e.g. **randomization procedure**)? If yes, have they been described?	Yes	Material and Methods
Include a statement about **blinding** even if no blinding was done.	Yes	Material and Methods
Describe **inclusion/exclusion criteria** if samples or animals were excluded from the analysis. Were the criteria pre-established?<br><br>If sample or data points were omitted from analysis, report if this was due to attrition or intentional exclusion and provide justification.	Yes	Material and Methods
For every figure, are **statistical tests** justified as appropriate? Do the data meet the assumptions of the tests (e.g., normal distribution)? Describe any methods used to assess it. Is there an estimate of variation within each group of data? Is the variance similar between the groups that are being statistically compared?	Yes	Material and Methods, Figures

Sample definition and in-laboratory replication	Information included in the manuscript?	In which section is the information available? (Reagents and Tools Table, Materials and Methods, Figures, Data Availability Section)
In the figure legends: state number of times the experiment was **replicated** in laboratory.	Yes	Figure legends
In the figure legends: define whether data describe **technical or biological replicates**.	Yes	Figure legends

Ethics

Ethics	Information included in the manuscript?	In which section is the information available? (Reagents and Tools Table, Materials and Methods, Figures, Data Availability Section)
Studies involving **human participants**: State details of **authority granting ethics approval** (IRB or equivalent committee(s), provide reference number for approval.	Yes	
Studies involving **human participants**: Include a statement confirming that **informed consent** was obtained from all subjects and that the experiments conformed to the principles set out in the WMA Declaration of Helsinki and the Department of Health and Human Services Belmont Report.	Yes	Material and methods
Studies involving **human participants:** For publication of **patient photos**, include a statement confirming that consent to publish was obtained.	Not Applicable	
Studies involving experimental **animals**: State details of **authority granting ethics approval** (IRB or equivalent committee(s), provide reference number for approval. Include a statement of compliance with ethical regulations.	Yes	Material and methods
Studies involving **specimen and field samples:** State if relevant **permits** obtained, provide details of authority approving study; if none were required, explain why.	Not Applicable	

Dual Use Research of Concern (DURC)	Information included in the manuscript?	In which section is the information available? (Reagents and Tools Table, Materials and Methods, Figures, Data Availability Section)
Could your study fall under dual use research restrictions? Please check biosecurity documents and list of **select agents and toxins** (CDC): https://www.selectagents.gov/sat/list.htm	Not Applicable	
If you used a select agent, is the security level of the lab appropriate and reported in the manuscript?	Not Applicable	
If a study is subject to dual use research of concern regulations, is the name of the **authority granting approval and reference number** for the regulatory approval provided in the manuscript?	Not Applicable	

Reporting

The MDAR framework recommends adoption of discipline-specific guidelines, established and endorsed through community initiatives. Journals have their own policy about requiring specific guidelines and recommendations to complement MDAR.

Adherence to community standards	Information included in the manuscript?	In which section is the information available? (Reagents and Tools Table, Materials and Methods, Figures, Data Availability Section)
State if relevant guidelines or checklists (e.g., **ICMJE, MIBBI, ARRIVE, PRISMA**) have been followed or provided.	Not Applicable	
For **tumor marker prognostic studies,** we recommend that you follow the **REMARK** reporting guidelines (see link list at top right). See author guidelines, under 'Reporting Guidelines'. Please confirm you have followed these guidelines.	Not Applicable	
For **phase II and III randomized controlled trials**, please refer to the **CONSORT** flow diagram (see link list at top right) and submit the CONSORT checklist (see link list at top right) with your submission. See author guidelines, under 'Reporting Guidelines'. Please confirm you have submitted this list.	Not Applicable	

Data Availability

Data availability	Information included in the manuscript?	In which section is the information available? (Reagents and Tools Table, Materials and Methods, Figures, Data Availability Section)
Have **primary datasets** been deposited according to the journal's guidelines (see 'Data Deposition' section) and the respective accession numbers provided in the Data Availability Section?	Yes	Data Availability Section
Were **human clinical and genomic datasets** deposited in a public access-controlled repository in accordance to ethical obligations to the patients and to the applicable consent agreement?	Not Applicable	
Are **computational models** that are central and integral to a study available without restrictions in a machine-readable form? Were the relevant accession numbers or links provided?	Not Applicable	
If publicly available data were reused, provide the respective **data citations in the reference list**.	Yes	Reference list
